# Studies towards the Enantioselective Synthesis of Cryptowolinol via Pd^0^-Catalyzed C(sp^3^)–H Arylation/Parallel Kinetic Resolution

**DOI:** 10.1021/acs.orglett.4c00386

**Published:** 2024-04-03

**Authors:** Takeru Miyakoshi, Domenic Kronenberg, Sota Tamaki, Rafael Lombardi, Olivier Baudoin

**Affiliations:** University of Basel, Department of Chemistry, St. Johanns-Ring 19, CH-4056 Basel, Switzerland

## Abstract

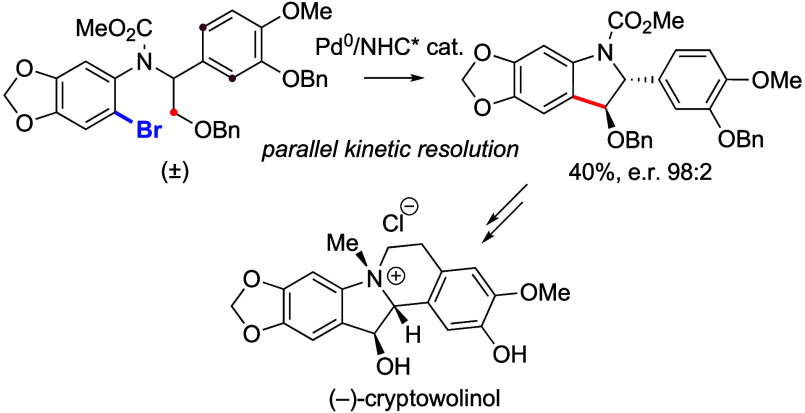

We report a model study towards the enantioselective synthesis of the dibenzopyrrocoline alkaloid (−)-cryptowolinol. The key step involves a challenging enantioselective Pd^0^-catalyzed C(sp^3^)–H arylation performed with a chiral NHC ligand, which proceeds via parallel kinetic resolution (PKR). A very efficient PKR process was achieved on a deoxygenated model substrate and was successfully transposed to a potential intermediate en route to (−)-cryptowolinol.

Among the abundant isoquinoline alkaloids,^1^ very few, such as cryptowoline (**1**) and cryptaustoline, possess the dibenzopyrrocoline framework (Scheme 1a).^2,3^ (−)-Cryptowolinol **2**, isolated together with **1** from the New Caledonian lauraceae *Cryptocarya phyllostemon*,^4^ displays an additional hydroxy group at C_12_, which makes its stereoselective synthesis challenging. In addition, doubts can be raised regarding the absolute configuration of both (−)-**1** and (−)-**2**, as the absolute configuration of (−)-cryptausoline, which differs from **1** only by its four free phenols, was reassigned to its enantiomer via total synthesis by Meyers and co-workers.^5^ Motivated by these problems and our interest in this field, we embarked on the enantioselective synthesis of cryptowolinol using Pd-catalyzed C–H activation as the key step.^6^ Our retrosynthetic analysis is depicted in Scheme 1a. We hypothesized that **2** could arise from protected arylindoline **3** (PG = protecting group) via Friedel–Crafts-type construction of the six-membered ring and methylation of the tertiary amine. In turn, enantioenriched **3** could arise from racemic aryl bromide **4** via enantioselective Pd^0^-catalyzed C(sp^3^)–H arylation occurring via parallel kinetic resolution (PKR).^7,8^ Indeed, Kündig and co-workers reported that racemic aryl bromides **7** containing two different alkyl groups undergo efficient PKR in the presence of a Pd/chiral NHC catalyst to generate an equimolar mixture of enantioenriched regioisomers **8** and **9**.^9^ We wondered if compound **10**, which models intermediate **4** and possesses a phenyl substituent instead of one of the alkyl groups in **7**, could undergo such a PKR process to furnish scalemic indoline **11** and dibenzophenanthridine **12**. An efficient PKR requires that the two substituents react at comparable rates with the catalyst. In the case of **10**, this is very challenging as C(sp^2^)–H bonds are usually much more reactive than secondary C(sp^3^)–H bonds,^10,11^ and therefore, **10** might rather undergo exclusive C(sp^2^)–H arylation to give racemic **12**.^12^ This work reports our model studies of the PKR of aryl bromides **10** and its application to the more substituted intermediate **4** en route to (−)-cryptowolinol.

**Scheme 1:**
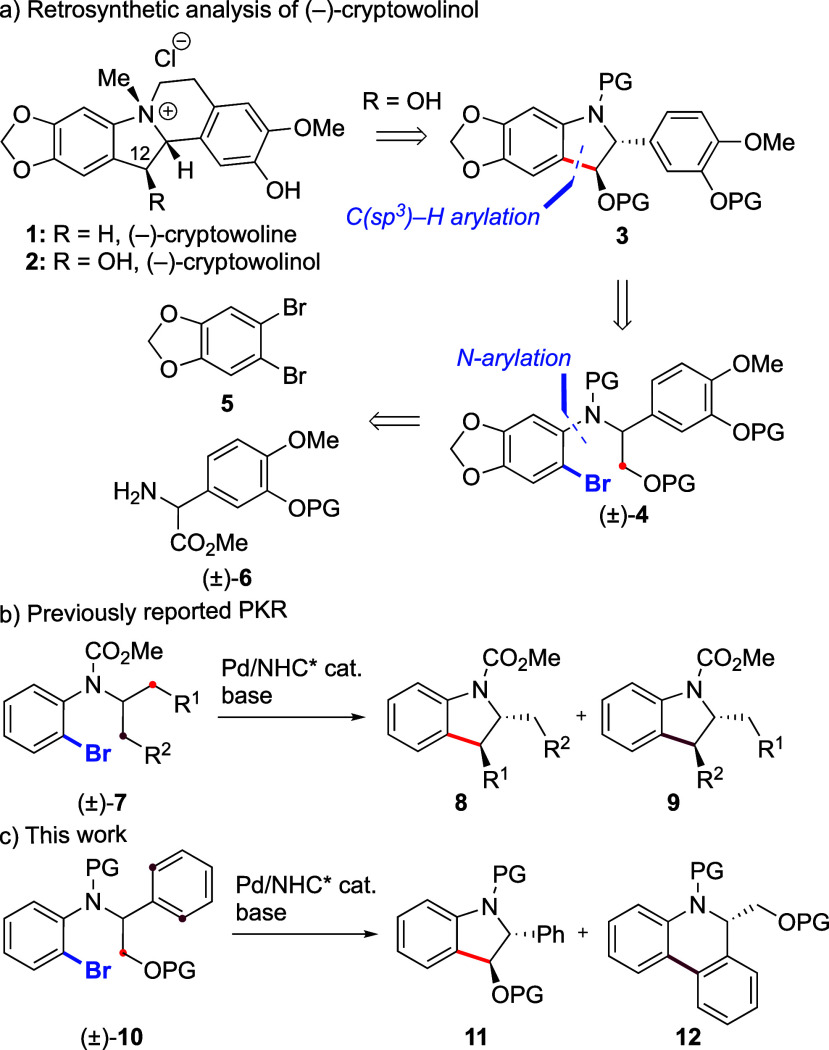
Retrosynthetic Analysis of (−)-Cryptowolinol

First, we investigated the effect of protecting groups on the primary alcohol (Y) and aniline (Z) on the PKR of racemic model substrates **10** (Table 1). Substrates bearing various silyl groups on the primary alcohol were first considered (entries 1–4). We employed conditions that were recently found to be optimal for the enantioselective C(sp^3^)–H arylation of secondary C–H bonds on linear alkyl chains,^13^ using IBioxtBu^14^ as the chiral ligand and [Pd(π-allyl)Cl]_2_ as the Pd source. Unfortunately, this resulted in the formation of C(sp^2^)–H arylation products **12a**–**d** in low to moderate yields and enantioselectivities, along with variable amounts of indole **13**, which was speculated to arise from elimination of the desired product. Given that **12** possesses a benzylic stereocenter, we probed its propensity to racemize by heating it under the reaction conditions but did not observe any significant racemization. Substrate **10e** bearing an ethyl instead of methyl carbamate was tested but did not furnish better results (entry 5). A pivaloyl protecting group was also introduced on the primary alcohol but did not yield the desired indoline product **11f** either (entry 6). In contrast, **11** was formed in low yield alongside **12** when ether protecting groups ^*t*^Bu, Bn, PMB, and MOM were employed (entries 7–10, respectively). In particular, the Bn, PMB, and MOM groups provided indolines **11h**–**j**, respectively, in excellent enantioselectivities (entries 8–10, respectively), which indicates that C(sp^3^)–H arylation is favored by a lower steric hindrance on the protected primary alcohol. It is noteworthy that the achiral IBioxMe_4_ ligand^15^ provided a higher proportion of dibenzophenanthridine product **12h**, which further illustrates the substrate innate preference for C(sp^2^)–H arylation with this family of ligands (entry 11; compare it with entry 8). Recrystallization of co-product **12j** afforded a highly enantiomerically enriched material (er 98.5:1.5), which could be analyzed by X-ray crystallography. This allowed determination of the absolute configuration of **12j** (as *R*) and by extension of indoline **11j** (*S* at the same carbon atom) and other products in this study. Surprisingly, using the aryl iodide (**10k**) instead of the bromide (**10h**) led to the exclusive formation of racemic C(sp^2^)–H arylation product **12k** (entry 12), which might reveal a change in the reaction mechanism. When COCF_3_ was used as an N-protecting group, multiple compounds were observed, with an only 10% yield of product **12l** observed by ^1^H NMR (entry 13), hence giving limited options to protect the nitrogen atom in the synthesis of cryptowolinol. As shown in entry 8, the C(sp^2^)–H arylation product is favored over the C(sp^3^)–H arylation product with IBioxtBu as the ligand, which leads to a low er for **12** and a high er for **11**. This corresponds to a high selectivity factor (*s*) for **11** (275) and a low *s* for **12** (6.7). To achieve a more efficient PKR, a slower C(sp^2^)–H arylation/faster C(sp^3^)–H arylation must be achieved so that both reactions occur at comparable rates. Tuning the substituents of the IBiox ligand did not lead to any improvement (entry 14). In contrast, chiral NHC ligands **L**^**1**^ and **L**^**2**^ employed by Kündig and co-workers for substrates **7** (see Scheme 1)^9,16^ provided a much more efficient PKR process (entries 15 and 16, respectively). In particular, (*R*,*R*)-**L**^**2**^ containing *o*-tolyl substituents provided a more equal rate of C(sp^3^)–H and C(sp^2^)–H arylation products **11h** and **12h**, thus translating into better er and *s* values for both products (entry 16). As these two products can be separated by standard column chromatography, the desired indoline **11h** was isolated in close-to-ideal yield (47%) and enantioselectivity (>99% ee). It is noteworthy that indoline **11h** was isolated as a single *trans* diastereoisomer in line with previous studies,^9^ which presumably results from minimization of repulsion between substituents at the C(sp^3^)–H activation transition state.

**Table 1:**
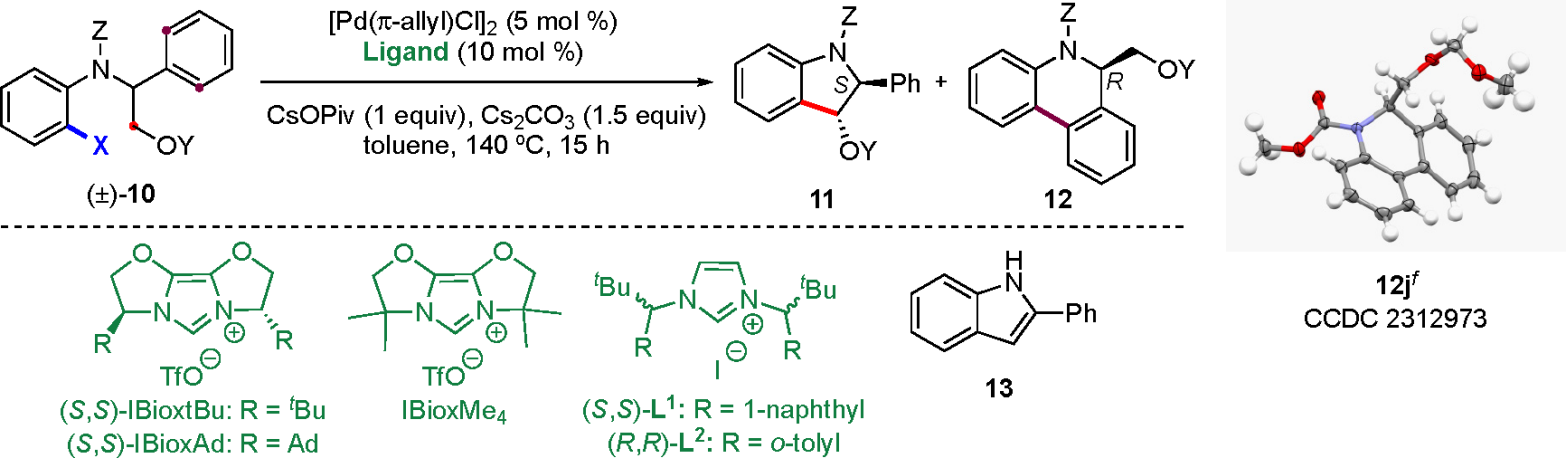
Assessment of Various Substrates in the Parallel Kinetic Resolution^a^

entry	substrate	X	Y	Z	ligand	yield of **11** (%)^b^	er of **11**^c^	*s* of **11**^d^	yield of **12** (%)^b^	er of **12**^c^	*s* of **12**^d^
1	**10a**	Br	TBS	CO_2_Me	(*S,S*)-IBioxtBu	–			22	78:22	
2	**10b**	Br	TES	CO_2_Me	(*S*,*S*)-IBioxtBu	–			14	68:32	
3	**10c**	Br	TBDPS	CO_2_Me	(*S*,*S*)-IBioxtBu	–			0		
4	**10d**	Br	TIPS	CO_2_Me	(*S*,*S*)-IBioxtBu	–			43	84:16	
5	**10e**	Br	TIPS	CO_2_Et	(*S*,*S*)-IBioxtBu	–			32	72:28	
6	**10f**	Br	Piv	CO_2_Me	(*S*,*S*)-IBioxtBu	–			60	77:23	
7	**10g**	Br	^*t*^Bu	CO_2_Me	(*S*,*S*)-IBioxtBu	trace			69	59:41	
8	**10h**	Br	Bn	CO_2_Me	(*S*,*S*)-IBioxtBu	22	99.5:0.5	275	62	69:31	6.7
9	**10i**	Br	PMB	CO_2_Me	(*S*,*S*)-IBioxtBu	14	99.4:0.6		59	64:36	
10	**10j**	Br	MOM	CO_2_Me	(*S*,*S*)-IBioxtBu	27	99.6:0.4		38	69:31	
11	**10h**	Br	Bn	CO_2_Me	IBioxMe_4_	12^e^	50:50		88^e^	50:50	
12	**10k**	I	Bn	CO_2_Me	(*S*,*S*)-IBioxtBu	–			89	50:50	
13	**10l**	Br	Bn	COCF_3_	(*S*,*S*)-IBioxtBu	–			10^e^		
14	**10h**	Br	Bn	CO_2_Me	(*S*,*S*)-IBioxAd	21	99:1	136	60	69:31	5.9
15	**10h**	Br	Bn	CO_2_Me	(*S*,*S*)-**L**^**1**^	35	0.2:99.8	1083	47	84:16	14.3
16	**10h**	Br	Bn	CO_2_Me	(*R*,*R*)-**L**^**2**^	47	99.8:0.2	1268	52	8:92	33

a Reaction conditions: **10** (0.1 mmol), [Pd(π-allyl)Cl]_2_ (5 mol %), ligand (10 mol %), CsOPiv (1 equiv), Cs_2_CO_3_ (1.5 equiv), 4 Å MS, toluene, 140 °C, 15 h. TBS = *tert-*butyldimethylsilyl. TES = triethylsilyl. TBDPS = *tert-*butyldiphenylsilyl. TIPS = triisopropylsilyl. Piv = pivaloyl. Bn = benzyl. PMB = *p*-methoxybenzyl. MOM = methoxymethyl.

b Yield of the isolated product.

c er values were determined by HPLC using a chiral stationary phase.

d Selectivity factors calculated from the equation *s* = ln[1 – *c*(1 + ee_Pr_)]/ln[1 – *c*(1 – ee_Pr_)] = *k*_rel_ = *k*_fast_/*k*_slow_, where *c* is the conversion determined by ^1^H NMR and Pr is the product.

e Determined by ^1^H NMR using trichloroethylene as the internal standard.

f Thermal ellipsoids shown at 50% probability. The absolute configurations of the other products were assigned in analogy to **12j**.

Following up on this model study, we embarked on the synthesis of advanced tetraoxygenated intermediate **3** toward (−)-cryptowolinol (Scheme 2). Commercially available benzaldehyde **14** first underwent Strecker and Pinner reactions to generate aminoester **6**. Reduction of the ester and protection as TBS ether were followed by Buchwald–Hartwig N-arylation with dibromide **5**, leading to intermediate **16** in excellent yield. Protecting group exchange to install the required benzyl group, followed by aniline protection with methyl chloroformate, furnished compound **4** in 22.7% overall yield over eight steps.

**Scheme 2:**
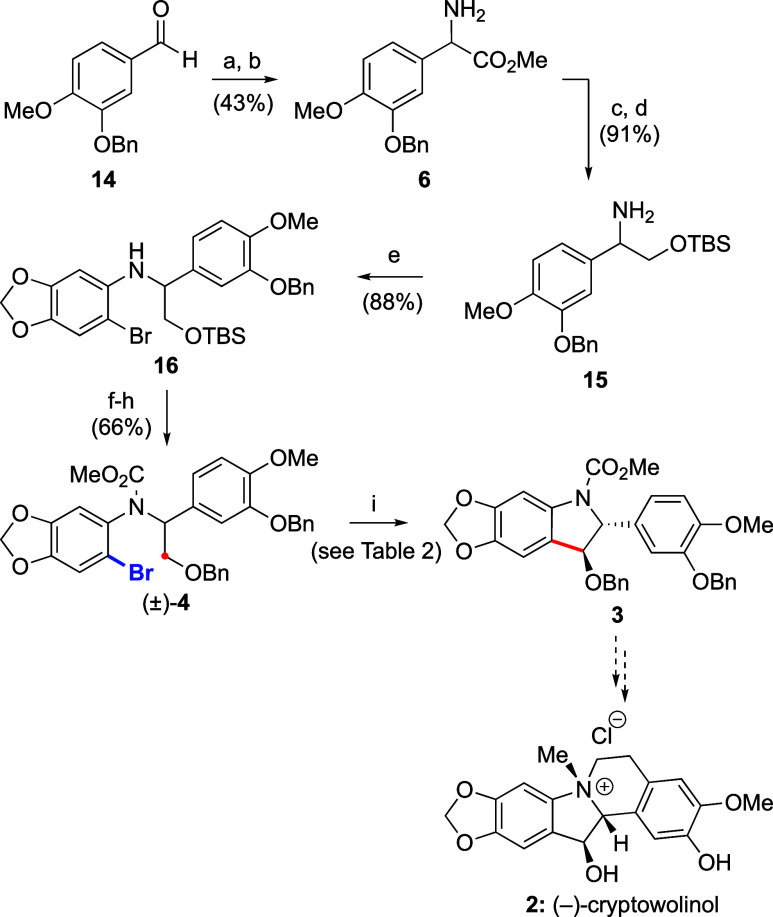
Enantioselective Synthesis of Advanced Intermediate **3** Reaction conditions: (a) LiHMDS (1.1 equiv), THF, −40 to 25 °C, then acetone cyanohydrin (2 equiv), 25 °C; (b) 3 M HCl/MeOH, 25 °C; (c) NaBH_4_ (3 equiv), EtOH, 0 °C; (d) TBSCl (1.1 equiv), Et_3_N (2.2 equiv), DMAP (10 mol %), CH_2_Cl_2_, 0 °C; (e) **15** (1.0 equiv), 5,6-dibromo-1,3-benzodioxole **5** (1.05 equiv), Pd_2_dba_3_ (4 mol %), (±)-BINAP (8 mol %), NaOtBu (1.5 equiv), toluene, 80 °C; (f) TBAF (2 equiv), THF, 0 to 25 °C; (g) NaH (2 equiv), BnBr (1.5 equiv), THF, 0 to 25 °C; (h) ClCO_2_Me, neat, reflux; (i) see Table 2.

Application of the conditions optimized for substrate **10** using (*R*,*R*)-**L**^**2**^ as the ligand (see Table 1, entry 16) to the more oxygenated substrate **4** provided lower yields of both C(sp^3^)–H and C(sp^2^)–H arylation products **3** and **17** (Table 2, entry 1). Indeed, the corresponding protodebrominated product was formed in 22% yield, which affected the efficiency of the PKR process. Analogous ligands **L**^**1**^ and **L**^**3**^ were tested on this substrate but did not furnish better results (entries 2 and 3, respectively). Nevertheless, scaling up the reaction with enantiomeric ligand (*S*,*S*)-**L**^**2**^ (entry 4) allowed the isolation of indoline **3** in satisfying yield (107 mg, 40%) and enantioselectivity (er 98:2). Unfortunately, despite significant experimentation, all attempts to cleave the methyl carbamate in **3** led to the elimination of the benzyloxy group and the formation of the corresponding indole. Future investigations will be devoted to the introduction of a more labile N-protecting group to complete the synthesis of (−)-cryptowolinol and confirm or reassign its absolute configuration.

**Table 2:**
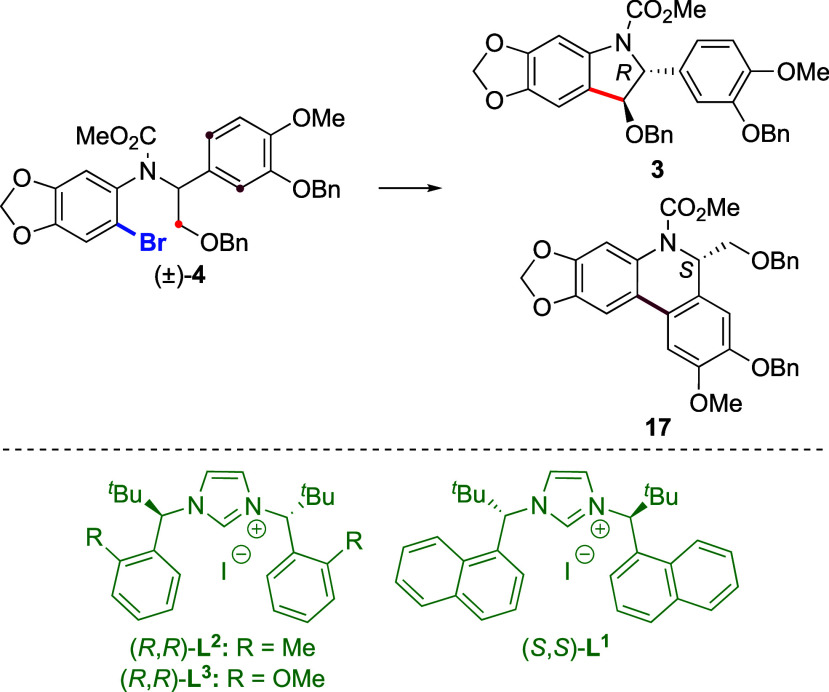
PKR of Aryl Bromide **4**^a^

entry	ligand	yield of **3** (%)^b^	er of **3**^c^	yield of **17** (%)^b^	er of **17**^c^
1	(*R*,*R*)-**L**^**2**^	34	0.5:99.5	35	40:60
2	(*S*,*S*)-**L**^**1**^	15	98.5:1.5	17	73:27
3	(*R*,*R*)-**L**^**3**^	16	4:96	6	34:66
4^d^	(*S*,*S*)-**L**^**2**^	40	98:2	–e	

a Reaction conditions: **4** (0.1 mmol), [Pd(π-allyl)Cl]_2_ (5 mol %), ligand (10 mol %), CsOPiv (1 equiv), Cs_2_CO_3_ (1.5 equiv), 4 Å MS, toluene, 140 °C, 15 h.

b Yield of the isolated product.

c er values were determined by HPLC using a chiral stationary phase.

d Run on a 0.5 mmol scale.

e This product could not be isolated in pure form on this scale. Absolute configurations were assigned on the basis of the X-ray crystal structure of **12j**.

In conclusion, we performed a model study of the enantioselective synthesis of the rare dibenzopyrrocoline alkaloid (−)-cryptowolinol. The key step involves a challenging enantioselective Pd^0^-catalyzed C(sp^3^)–H arylation proceeding via parallel kinetic resolution. A very efficient PKR was achieved on a deoxygenated model substrate and was successfully transposed to a potential intermediate en route to (−)-cryptowolinol.

## Data Availability

The data underlying this study are available in the published article and its Supporting Information.

## Supporting Information Available

Procedural and spectral data (PDF)

## References

[ref1] Bentley K. W. β-Phenylethylamines and the Isoquinoline Alkaloids. Nat. Prod. Rep. 2006, 23, 444–463. 10.1039/B509523A. 16741588

[ref2] Elliott I. W. Dibenzopyrrocoline Alkaloids. Alkaloids 1987, 31, 101–116. 10.1016/S0099-9598(08)60259-X.

[ref3] aEwing J.; Hughes G. K.; Ritchie E.; Taylor W. C. An Alkaloid Related to Dehydrolaudanosoline. Nature 1952, 169, 618–619. 10.1038/169618b0.

[ref4] aLeboeuf M.; Cavé A.; Ranaivo A.; Moskowitz H. Cryptowolinol et Cryptowolidine, Nouveaux Alcaloïdes de Type Dibenzopyrrocoline. Can. J. Chem. 1989, 67, 947–952. 10.1139/v89-145.

[ref5] Meyers A. I.; Sielecki T. M. Total Synthesis of the Dibenzopyrrocoline Alkaloid (*S*)-(+)-Cryptaustoline. Revision of Absolute Configuration Due to an Unusual Inversion in Stereochemistry. J. Am. Chem. Soc. 1991, 113, 2789–2790. 10.1021/ja00007a084.

[ref6] aYamaguchi J.; Yamaguchi A. D.; Itami K. C–H Bond Functionalization: Emerging Synthetic Tools for Natural Products and Pharmaceuticals. Angew. Chem., Int. Ed. 2012, 51, 8960–9009. 10.1002/anie.201201666. 22887739

[ref7] aEames J. Parallel Kinetic Resolutions. Angew. Chem., Int. Ed. 2000, 39, 885–888. 10.1002/(SICI)1521-3773(20000303)39:5<885::AID-ANIE885>3.0.CO;2-2. 10760881

[ref8] aNewton C. G.; Wang S.-G.; Oliveira C. C.; Cramer N. Catalytic Enantioselective Transformations Involving C–H Bond Cleavage by Transition-Metal Complexes. Chem. Rev. 2017, 117, 8908–8976. 10.1021/acs.chemrev.6b00692. 28212007

[ref9] aKatayev D.; Nakanishi M.; Bürgi T.; Kündig E. P. Asymmetric C(sp^3^)–H/C(Ar) Coupling Reactions. Highly *Enantio*-enriched Indolines via Regiodivergent Reaction of a Racemic Mixture. Chem. Sci. 2012, 3, 1422–1425. 10.1039/c2sc20111a.

[ref10] Baudoin O. Ring Construction by Palladium(0)-Catalyzed C(sp^3^)–H Activation. Acc. Chem. Res. 2017, 50, 1114–1123. 10.1021/acs.accounts.7b00099. 28375627

[ref11] Wheatley M.; Zuccarello M.; Tsitopoulou M.; Macgregor S. A.; Baudoin O. Effect of α-Substitution on the Reactivity of C(sp^3^)–H Bonds in Pd^0^-Catalyzed C–H Arylation. ACS Catal. 2023, 13, 12563–12570. 10.1021/acscatal.3c03806. 37822862 PMC10563019

[ref12] aYang S.-Y.; Han W.-Y.; Zhang D.-L.; Zhou X.-J.; Bai M.; Cui B.-D.; Wan N.-W.; Yuan W.-C.; Chen Y.-Z. Synthesis of Phenanthridines through Palladium-Catalyzed Cascade Reaction of 2-Halo-*N*-Ms-arylamines with Benzyl Halides/Sulfonates. Eur. J. Org. Chem. 2017, 2017, 996–1003. 10.1002/ejoc.201601608.

[ref13] Melot R.; Zuccarello M.; Cavalli D.; Niggli N.; Devereux M.; Bürgi T.; Baudoin O. Palladium(0)-Catalyzed Enantioselective Intramolecular Arylation of Enantiotopic Secondary C–H Bonds. Angew. Chem., Int. Ed. 2021, 60, 7245–7250. 10.1002/anie.202014605. 33325596

[ref14] Glorius F.; Altenhoff G.; Goddard R.; Lehmann C. Oxazolines as Chiral Building Blocks for Imidazolium Salts and N-Heterocyclic Carbene Ligands. Chem. Commun. 2002, 2704–2705. 10.1039/b208045a. 12510308

[ref15] Altenhoff G.; Goddard R.; Lehmann C. W.; Glorius F. Sterically Demanding, Bioxazoline-Derived N-Heterocyclic Carbene Ligands with Restricted Flexibility for Catalysis. J. Am. Chem. Soc. 2004, 126, 15195–15201. 10.1021/ja045349r. 15548016

[ref16] Kündig E. P.; Seidel T. M.; Jia Y.-x.; Bernardinelli G. Bulky Chiral Carbene Ligands and Their Application in the Palladium-Catalyzed Asymmetric Intramolecular α-Arylation of Amides. Angew. Chem., Int. Ed. 2007, 46, 8484–8487. 10.1002/anie.200703408. 17912728

