# Supplementary material for: Studies towards the Enantioselective Synthesis of Cryptowolinol via Pd0-Catalyzed C(sp3)–H Arylation/Parallel Kinetic Resolution
Source: Org Lett. 2024 Apr 3;26(15):2923–7. doi: 10.1021/acs.orglett.4c00386 (PMC11040710; doi:10.1021/acs.orglett.4c00386)

Studies Towards the Enantioselective Synthesis of Cryptowolinol via Pd^0-Catalyzed $C(sp^3)$–H Arylation/Parallel Kinetic Resolution

Takeru Miyakoshi, Domenic Kronenberg, Sota Tamaki, Rafael Lombardi,

and Olivier Baudoin*

University of Basel, Department of Chemistry, St. Johanns-Ring 19, CH-4056 Basel, Switzerland

** E-mail: olivier.baudoin@unibas.ch*

Table of Contents

General Information 3

General Procedures 4

Optimization of Chiral Ligands for Parallel Kinetic Resolution 5

Calculated Reaction Profiles 6

Synthesis of Model Substrates for Optimization of Parallel Kinetic Resolution 8

C–H Activation Products of Model Substrates via Parallel Kinetic Resolution 22

Synthetic Procedures and Characterization Data 30

PKR for Enantioselective Core Synthesis of Cryptowolinol 38

Scaled up PKR for Enantioselective Core Synthesis of Cryptowolinol 40

Crystallographic data 41

References 44

NMR Spectrum Data of Model Substrates 45

NMR and HPLC Spectrum Data of C–H Products in Kinetic Resolution 60

NMR and HPLC Spectrum Data in the Core Construction of Cryptowolinol 86

General Information

Experimental Procedures, Reagents, and Glassware: All reactions involving air-sensitive materials were carried out in pre-dried glassware under an argon atmosphere by using Schlenk techniques employing double-line argon-vacuum lines and working in an argon-filled glove box. Chemicals were used as obtained from the suppliers (Sigma Aldrich, Acros Organics, Fluorochem, TCI) unless otherwise stated. Anhydrous THF, DMF, and CH_2Cl_2 were purchased from Acros Organics or Sigma Aldrich. The solvents were degassed with a flow of argon for 20 minutes. For reactions requiring heating, a metal heating block was used for catalytic C–H activation unless mentioned, otherwise an oil bath was used.

Chromatography: Analytical thin-layer chromatography (TLC) was performed using pre-coated Merck silica gel 60 F254 plates (0.25 mm). Visualization of the developed chromatogram was performed by UV absorbance (254 nm) or TLC stains ($KMnO_4$ and Phosphomolybdic acid). Flash chromatography was performed using Silicycle SiliaFlash P60 (230 – 400 mesh) with the indicated eluent system.

NMR Spectroscopy: Proton nuclear magnetic resonance (^{1}H NMR) data were acquired at 400 MHz on a Bruker Advance 400 spectrometer or at 500 MHz on a Bruker Advance 500 spectrometer. Chemical shifts (δ) are reported in parts per million (ppm) relative to residual chloroform (s, 7.26 ppm) or dichlormethane (t, 5.32 ppm). Proton decoupled Carbon-13 nuclear magnetic resonance (^{13}C{^{1}H} NMR) data were acquired at 101 MHz on a Bruker Advance 400 spectrometer or at 126 MHz on a Bruker Advance 500 spectrometer. Chemical shifts are reported in ppm relative to residual chloroform (77.16 ppm) or dichloromethane (p, 53.84 ppm). Proton decoupled Fluorine-19 nuclear magnetic resonance (^{19}F{^{1}H} NMR) were acquired at 376 MHz on a Bruker Advance 400 spectrometer. Splitting patterns are designated as s, singlet; d, doublet; t, triplet; q, quartet; p, pentet; hept, heptet; dd, doublet of doublets; dt, doublet of triplets; ddd, doublet of doublets of doublets; tt, triplet of triplets; tq, triplet of quartets; qt, quartet of triplets; m, multiplet. All NMR data were recorded at 298 K.

Infrared Spectroscopy: Infrared (IR) data were recorded on an ATR Varian Scimitar 800. Absorbance frequencies are reported in reciprocal centimetres (cm^{-1}).

Mass Spectrometry: HRMS measurements were performed on a Bruker maXis 4G QTOF ESI. High resolution mass are given in m/z.

Melting points: were measured on a Büchi B-565 and are uncorrected.

X-ray crystallography: was performed by Dr. A. Prescimone (University of Basel).

General Procedures

Synthesis of Secondary Amines (general procedure A)[1]

A mixture of primary amine (1 equiv.), styrene oxide (3 equiv.), silica gel (10% w/w) in toluene (0.3 M) was heated to 70 ºC overnight. The solvent was evaporated *in vacuo*, and the residue was purified by chromatography on silica gel using cyclohexane/AcOEt as a solvent to afford the pure compounds.

Carbamation of Secondary Amines (general procedure B)[2]

A mixture of *N*-alkyl-*o*-bromoarylamine in chloroformate (2-3 mL/mmol) was heated under reflux overnight. The mixture was concentrated *in vacuo*. The crude material was purified by flash column chromatography using cyclohexane/AcOEt mixture as an eluent.

C–H Activation with NHCs (general procedure C)

Substrate (0.1 mmol, 1 equiv.) was weighted in a 10 mL tube. $[Pd(\pi\text{-allyl})Cl)]_2$ (1.88 mg, 0.005 mmol, 5 mol%), NHCs (0.01 mmol, 10 mol%), CsOPiv (23.4 mg, 0.1 mmol, 1 equiv.), Cs_2CO_3 (48.9 mg, 0.15 mmol, 1.5 equiv.), and 4Å MS (25 mg) were weighted in a glovebox. The tube was closed with a septum, taken out of the glovebox, and solvent (1 mL) was added. The reaction was stirred in a heating block preheated at 140 °C for 15 h. The reaction was cooled to room temperature, filtered through a pad of *Celite*, washed with AcOEt and concentrated *in vacuo*. The crude mixture was analyzed by GC-MS, and purified by chromatography on silica gel or preparative thin-layer chromatography providing the desired product.

Racemic materials were obtained following the same procedure, using $IBioxMe_4$·HOTf or (*rac*)-NHC as a ligand.

Optimization of Chiral Ligands for Parallel Kinetic Resolution

Table 1. Ligand screening for PKR of racemic **10h**

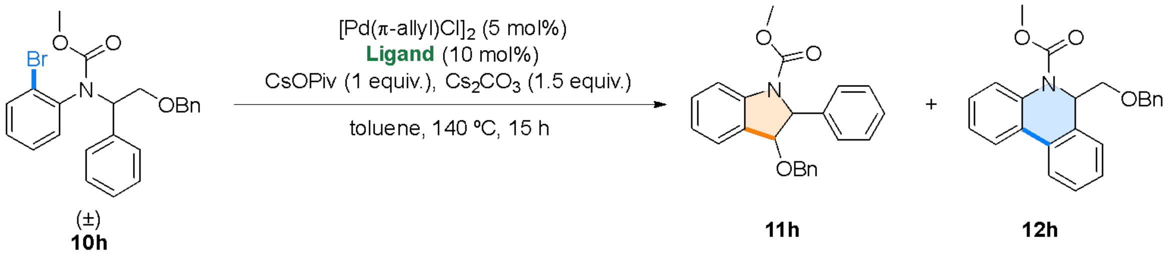

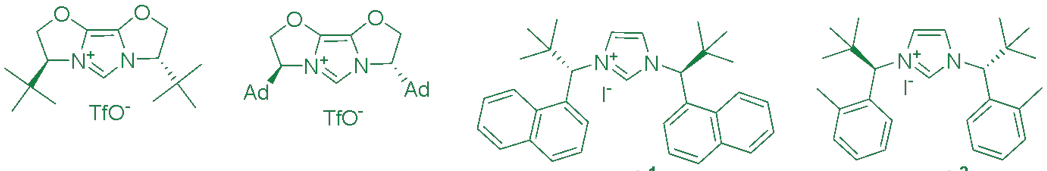

(*S*,*S*)-IBiox*t*Bu (*S*,*S*)-IBioxAd (*S*,*S*)-**L1** (*R*,*R*)-**L2**

Entry	NHC	NMR yield of 2.9/2.10[b]	e.r. of 11/e.r. of 12
1	IBioxtBu	71% (62%)[c]/ 25% (22%)[c]	99.5:0.5/31:69
2	IBioxAd	70% (60%)[c]/ 25% (21%)[c]	99:1/31:69
3	**L1**	56%(47%)[c]/ 42% (35%)[c]	0.2:99.8/84:16
4	**L2**	51% (52%)[c] 45% (47%)[c]	99.8:0.2/8:92

[a]**2.8** (0.1 mmol) was engaged. [b]Determined by ^{1}H NMR using trichloroethylene as internal standard. [c]Isolated yield.

Calculated Reaction Profiles

The following equation was employed to obtain each number.

$$s = \mathrm{In}[1\text{-}c(1\text{+}ee\mathrm{Pr})]/\mathrm{In}[1\text{-}c(1\text{-}ee\mathrm{Pr})] = k_{\mathrm{rel}} = k_{\mathrm{fast}}/k_{\mathrm{slow}}$$

Conversion (c) observed by ^{1}H NMR was introduced in the equation above.

The absolute configuration was assigned according to the absolute configuration of product **12j** which was determined by X-ray crystallography.

Entry 1 (IBioxtBu)

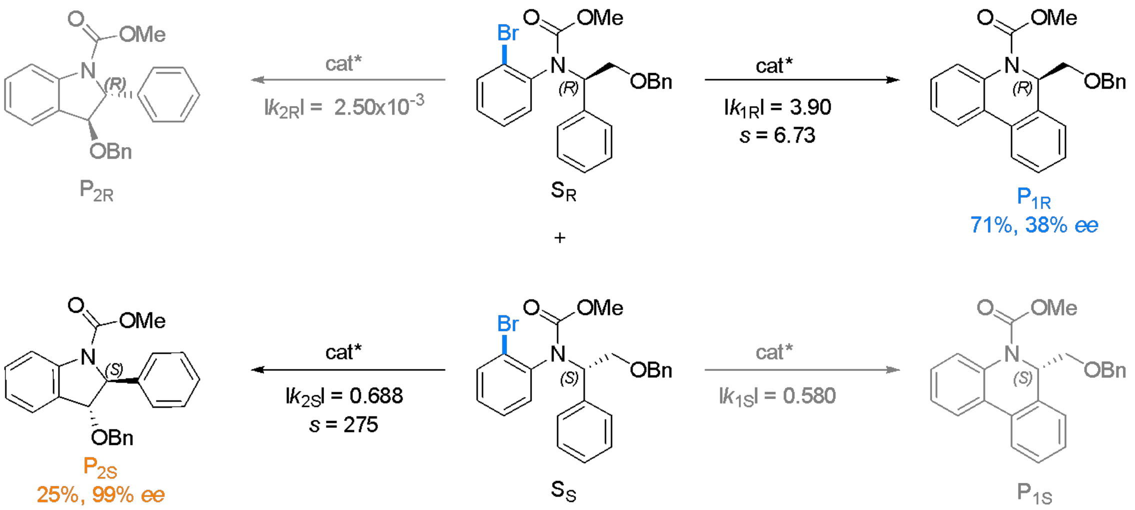

Entry 2 (IBioxAd)

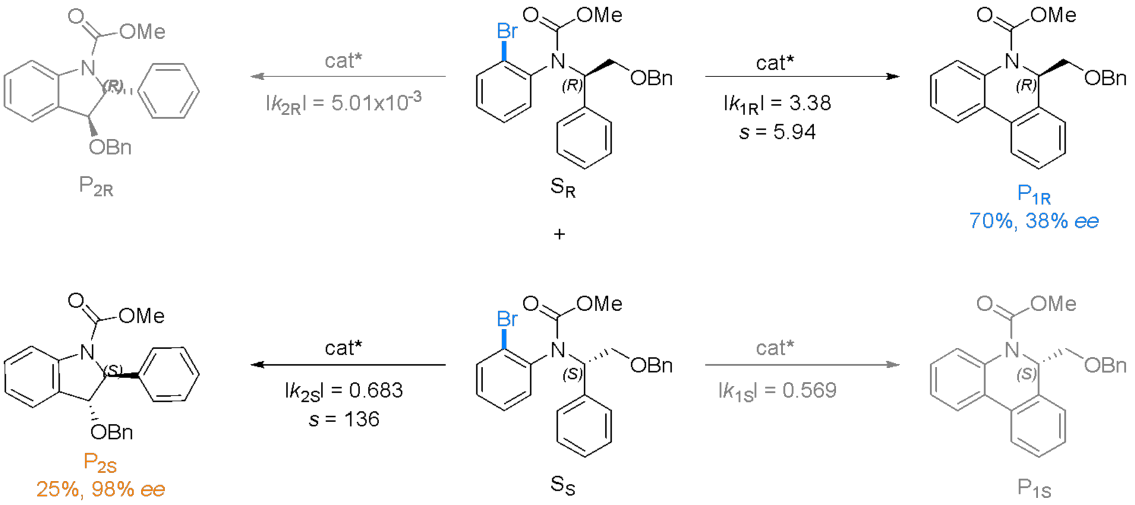

Entry 3 (L[1])

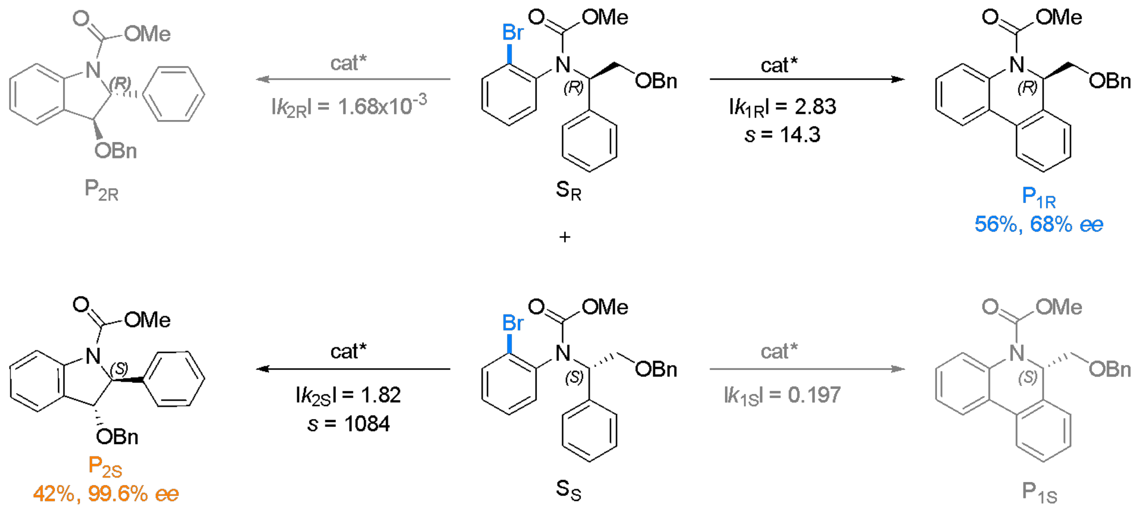

Entry 4 (L[2])

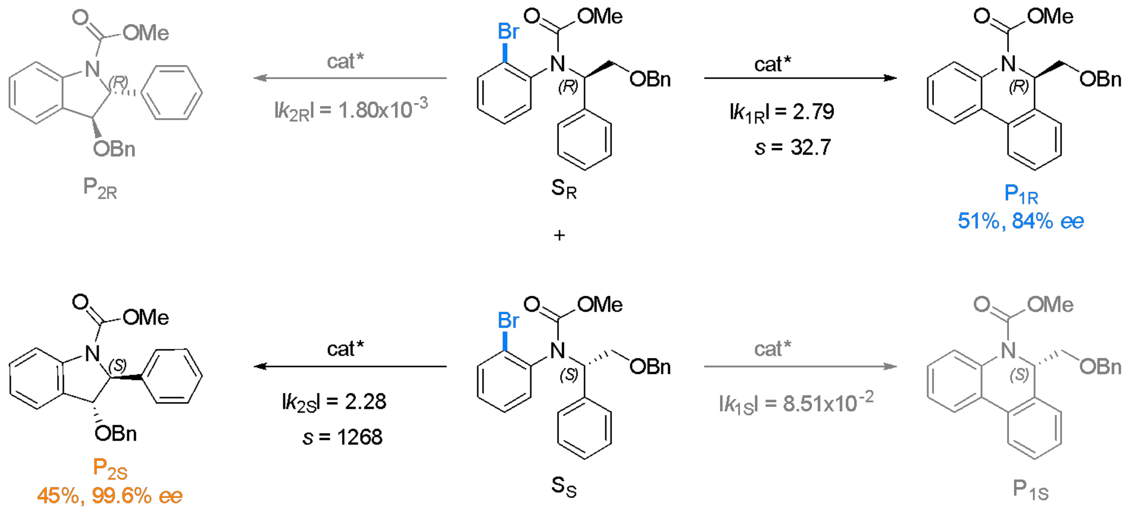

Synthesis of Model Substrates for Optimization of Parallel Kinetic Resolution

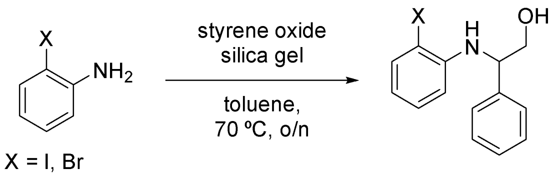

2-((2-bromophenyl)amino)-2-phenylethan-1-ol (SI-1):

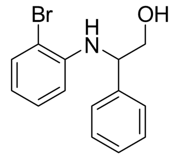

Following general procedure A, a mixture of 2-bromoaniline (2.58 g, 15 mmol, 1 equiv.), styrene oxide (5.2 mL, 45 mmol, 3 equiv.), silica gel (10% w/w) in toluene (45 mL, 0.3 M) was heated to 70 ºC overnight. The solvent was evaporated *in vacuo,* and the residue was purified by chromatography on silica gel using cyclohexane/AcOEt as a solvent to afford 2-((2-bromophenyl)amino)-2-phenylethan-1-ol as a yellow solid (3.48 g, 11.9 mmol, 79%).

^{1}H NMR (400 MHz, Chloroform-*d*) δ 7.43 (dd, *J* = 7.9, 1.5 Hz, 1H), 7.39 – 7.34 (m, 4H), 7.32 – 7.27 (m, 1H), 7.00 (ddd, *J* = 8.5, 7.3, 1.5 Hz, 1H), 6.54 ("t"d, *J* = 7.6, 1.5 Hz, 1H), 6.42 (dd, *J* = 8.2, 1.5 Hz, 1H), 5.18 (s, 1H), 4.57 (dd, *J* = 6.8, 4.2 Hz, 1H), 4.06 – 3.92 (m, 1H), 3.84 (dd, *J* = 11.3, 6.7 Hz, 1H), 1.72 (s, 1H).

^{13}C{^{1}H} NMR (126 MHz, Chloroform-*d*) δ 144.1, 139.6, 132.5, 129.1, 128.5, 127.9, 126.8, 118.5, 113.1, 110.5, 67.5, 59.95.

HRMS (ESI): Calcd for $C_{14}H_{15}BrNO$ $[M+H]^+$: 292.0332, found: 292.0331.

IR (neat): ν (cm^{-1}) 3317, 2934, 1595, 1505, 1452, 1323, 1176, 1063, 1012, 933, 854, 740, 698, 673.

Rf 0.18 (Cyclohexane:AcOEt = 7:1)

Melting point: 75 ºC

2-((2-iodophenyl)amino)-2-phenylethan-1-ol (SI-2):

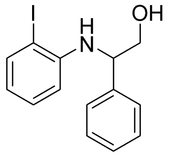

Chemical Formula: $C_{14}H_{14}INO$
Molecular Weight: 339.18

Following general procedure A, a mixture of 2-iodoaniline (2.63 g, 12 mmol, 1 equiv.), styrene oxide (4.1 mL, 36 mmol, 3 equiv.), silica gel (10% w/w) in toluene (40 mL, 0.3 M) was heated to 70 ºC overnight. The solvent was evaporated *in vacuo,* and the residue was purified by chromatography on silica gel using cyclohexane/AcOEt as a solvent to afford 2-((2-iodophenyl)amino)-2-phenylethan-1-ol as a dark solid (2.98 g, 8.73 mmol, 73%).

^{1}H NMR (400 MHz, Chloroform-*d*) δ 7.67 (dd, *J* = 7.8, 1.5 Hz, 1H), 7.35 (d, *J* = 3.8 Hz, 4H), 7.32 – 7.26 (m, 1H), 7.03 (ddd, *J* = 8.3, 7.3, 1.5 Hz, 1H), 6.42 ("t"d, *J* = 7.5, 1.5 Hz, 1H), 6.36 (dd, *J* = 8.2, 1.5

Hz, 1H), 5.07 (s, 1H), 4.57 (dd, *J* = 6.6, 4.2 Hz, 1H), 4.00 (dd, *J* = 11.1, 4.2 Hz, 1H), 3.84 (dd, *J* = 11.1, 6.6 Hz, 1H), 1.73 (s, 1H).

^{13}C{^{1}H} NMR (126 MHz, Chloroform-*d*) δ 146.3, 139.5, 139.1, 129.4, 129.1, 127.9, 126.8, 119.4, 112.4, 86.3, 67.5, 60.3.

HRMS (ESI): Calcd for $C_{14}H_{15}INO$ $[M+H]^+$: 340.0193, found: 340.0197.

IR (neat): ν (cm^{-1}) 3360, 1584, 1503, 1448, 1304, 1172, 1058, 1006, 740, 643.

Rf 0.22 (Cyclohexane:AcOEt = 7:1)

Melting point: 69 ºC

Methyl (2-bromophenyl)(2-((*tert*-butyldimethylsilyl)oxy)-1-phenylethyl)carbamate (10a):

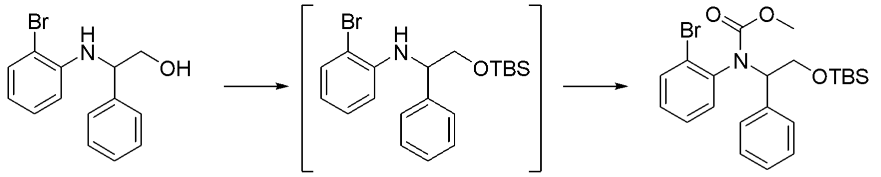

10a
Chemical Formula: $C_{22}H_{30}BrNO_3Si$
Molecular Weight: 464.48

To a solution of 2-((2-bromophenyl)amino)-2-phenylethan-1-ol (584 mg, 2 mmol, 1 equiv.) in DMF (7 mL, 0.3 M) was added imidazole (272 mg, 4 mmol, 2 equiv.), DMAP (24 mg, 0.2 mmol, 10 mol%) and TBSCl (452 mg, 3 mmol, 1.5 equiv.) at 0 ºC and stirred at room temperature overnight. H_2O was added and extracted with Et_2O, washed with brine, dried over Na_2SO_4 anhydrous, filtered and concentrated *in vacuo*. The residue was purified by silica gel column chromatography using cyclohexane/ CH_2Cl_2 (94:6, Rf= 0.35) as a solvent to afford the pure compound 2-bromo-*N*-(2-((*tert*-butyldimethylsilyl)oxy)-1-phenylethyl)aniline as a yellow oil (1.31g, 2.89 mmol, 98%).

Following general procedure B, a mixture of 2-bromo-*N*-(2-((*tert*-butyldimethylsilyl)oxy)-1-phenylethyl)aniline (737 mg, 1.81 mmol, 1 equiv.) in methyl chloroformate (6 mL) was heated under reflux overnight. The mixture was concentrated *in vacuo*. The crude material was purified by flash column chromatography using cyclohexane/AcOEt as a solvent to afford the pure compound Methyl (2-bromophenyl)(2-((*tert*-butyldimethylsilyl)oxy)-1-phenylethyl)carbamate **10a** as a colorless oil (780 mg, 1.68 mmol, 93%).

^{1}**H NMR** (400 MHz, Chloroform-*d*) δ 7.58 (dd, *J* = 7.8, 1.6 Hz, 0.4H), 7.49 – 7.41 (m, 1.2H), 7.40 – 7.01 (m, 7H), 7.00 – 6.94 (m, 0.4H), 5.46 (“t”, *J* = 7.5 Hz, 0.6H), 5.23 (“t”, *J* = 7.1 Hz, 0.4H), 4.39 (br, 0.4H), 4.13 (dd, *J* = 10.9, 8.6 Hz, 0.6H), 4.03 (br, 0.6H), 3.85 (dd, *J* = 10.4, 7.2 Hz, 0.4H), 3.64 (s, 3H), 0.90 (s, 5.4H), 0.79 (s, 3.6H), 0.09 (s, 1.8H), 0.07 (s, 1.8H), -0.01 (s, 1.2H), -0.04 (s, 1.2H).

^{13}**C{^{1}H} NMR** (101 MHz, Chloroform-*d*) δ 156.1, 155.6, 140.2, 138.7, 138.1, 136.6, 133.3, 133.2, 131.3, 129.2, 129.1, 128.9, 128.7, 128.2, 128.1, 128.1, 127.9, 127.8, 127.7, 126.7, 125.5, 65.8, 63.7, 63.2, 62.5, 53.2, 53.2, 25.9, 25.9, 18.3, 18.3, -5.2, -5.3, -5.4, -5.4.

HRMS (ESI): Calcd for $C_{22}H_{31}BrNO_3Si$ $[M+H]^+$: 464.1251, found: 464.1244.

IR (neat): ν (cm^{-1}) 2953, 2856, 1711, 1442, 1391, 1314, 1256, 1193, 1108.

Rf 0.16 (Cyclohexane:AcOEt = 60:1)

Methyl (2-bromophenyl)(1-phenyl-2-((triethylsilyl)oxy)ethyl)carbamate (10b):

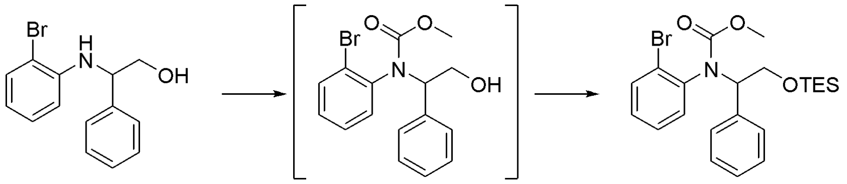

10b
Chemical Formula: $C_{22}H_{30}BrNO_3Si$
Molecular Weight: 464.48

Following general procedure B, a mixture of 2-((2-bromophenyl)amino)-2-phenylethan-1-ol (1.75 g, 6 mmol, 1 equiv.) in methyl chloroformate (18 mL) was heated under reflux overnight. The mixture was concentrated *in vacuo*. The crude material was purified by flash column chromatography using cyclohexane/AcOEt (2:1, Rf= 0.25) as a solvent to afford the pure compound methyl (2-bromophenyl)(2-hydroxy-1-phenylethyl)carbamate as a white solid (1.94 g, 5.54 mmol, 92%).

To a solution of methyl (2-bromophenyl)(2-hydroxy-1-phenylethyl)carbamate (350 mg, 1 mmol, 1 equiv.) in CH_2Cl_2 (3 mL) was added 2,6-lutidine (291 µL, 2.5 mmol, 2.5 equiv.) and TESOTf (452 µL, 2 mmol, 2 equiv.) at 0 ºC and stirred at room temperature overnight. NH_4Cl aq was added at 0 ºC and extracted with CH_2Cl_2. The organic phases were washed with brine, dried, filtered and concentrated *in vacuo*. The crude material was purified by flash column chromatography using cyclohexane/AcOEt as a solvent to afford the pure compound Methyl (2-bromophenyl)(1-phenyl-2-((triethylsilyl)oxy)ethyl)carbamate **10b** as a colorless oil (435 mg, 0.94 mmol, 94%).

^{1}H NMR (400 MHz, Chloroform-*d*) δ 7.58 (dd, *J* = 7.8, 1.7 Hz, 0.4H), 7.50 – 7.40 (m, 1.2H), 7.41 – 7.01 (m, 7H), 6.99 – 6.91 (m, 0.4H), 5.43 ("t", *J* = 7.3 Hz, 0.6H), 5.24 ("t", *J* = 7.2 Hz, 0.4H), 4.39 (br, 0.4H), 4.15 (dd, *J* = 10.8, 8.2 Hz, 0.6H), 4.08 (br, 0.6H), 3.86 (dd, *J* = 10.4, 7.3 Hz, 0.4H), 3.65 (s, 3H), 0.95 (t, *J* = 7.9 Hz, 5H), 0.87 (t, *J* = 7.9 Hz, 4H), 0.63 (q, *J* = 8.1 Hz, 3.4H), 0.53 (q, *J* = 7.7 Hz, 2.6H).

^{13}C{^{1}H} NMR (101 MHz, Chloroform-*d*) δ 156.0, 155.4, 140.1, 138.6, 138.1, 136.6, 133.2, 133.1, 131.2, 131.2, 129.1, 128.9, 128.8, 128.6, 128.1, 128.0, 128.0, 127.97, 127.9, 127.8, 127.7, 127.6, 126.5, 125.5, 65.7, 63.8, 62.95, 62.1, 53.1, 53.0, 6.8, 6.7, 4.5, 4.3.

HRMS (ESI): Calcd for $C_{22}H_{31}BrNO_3Si$ $[M+H]^+$: 464.1251, found: 464.1250.

IR (neat): ν (cm^{-1}) 2953, 2877, 1711, 1586, 1442, 1391, 1314, 1193, 1108, 1009.

Rf 0.15 (Cyclohexane:AcOEt = 40:1)

Methyl (2-bromophenyl)(2-((*tert*-butyldiphenylsilyl)oxy)-1-phenylethyl)carbamate (10c):

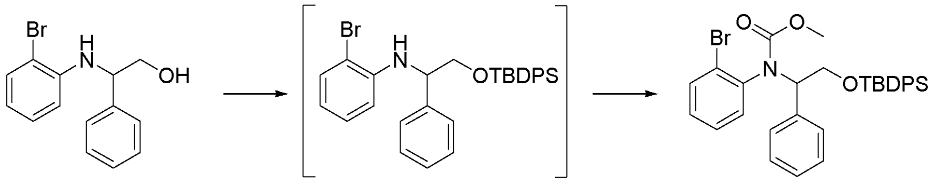

10c
Chemical Formula: $C_{32}H_{34}BrNO_3Si$
Molecular Weight: 588.62

To a solution of 2-((2-bromophenyl)amino)-2-phenylethan-1-ol (584 mg, 2 mmol, 1 equiv.) in DMF (7 mL, 0.3 M) was added imidazole (272 mg, 4 mmol, 2 equiv.) and TBDPSCl (778 μL, 3 mmol, 1.5 equiv.) at 0 ºC and stirred at room temperature overnight. H_2O was added and extracted with Et_2O, washed with brine, dried over Na_2SO_4 anhydrous, filtered and concentrated *in vacuo*. The residue was purified by silica gel column chromatography using cyclohexane/CH_2Cl_2 as solvent to afford the pure compound 2-bromo-*N*-(2-((*tert*-butyldiphenylsilyl)oxy)-1-phenylethyl)aniline as a yellow oil (982 mg, 1.85 mmol, 93%).

Following general procedure B, a mixture of 2-bromo-*N*-(2-((*tert*-butyldiphenylsilyl)oxy)-1-phenylethyl)aniline (982 mg, 1.85 mmol, 1 equiv.) in methyl chloroformate (6 mL) was heated under reflux overnight. The mixture was concentrated *in vacuo*. The crude material was purified by flash column chromatography using cyclohexane/AcOEt as a solvent to afford the pure compound Methyl (2-bromophenyl)(2-((*tert*-butyldiphenylsilyl)oxy)-1-phenylethyl)carbamate **10c** as a colorless oil (1.03 g, 1.75 mmol, 95%).

^{1}H NMR (400 MHz, Chloroform-*d*) δ 7.71 (d"t", *J* = 6.5, 1.6 Hz, 1.2H), 7.65 (d"t", *J* = 6.6, 1.6 Hz, 1.2H), 7.59 – 7.06 (m, 15H), 6.98 (d, *J* = 7.4 Hz, 0.4H), 6.91 (d, *J* = 7.5 Hz, 1.2H), 5.63 (br, 0.6H), 5.32 (t, *J* = 6.9 Hz, 0.4H), 4.34 (m, 0.4H), 4.18 (dd, *J* = 10.9, 9.1 Hz, 0.6H), 4.02 (dd, *J* = 10.4, 5.8 Hz, 0.6H), 3.88 (dd, *J* = 10.6, 7.1 Hz, 0.4H), 3.65 (s, 1.8H), 3.63 (s, 1.2H), 1.07 (s, 6H), 0.93 (s, 3H).

^{13}C{^{1}H} NMR (101 MHz, Chloroform-*d*) δ 156.0, 155.4, 139.7, 138.5, 137.8, 136.0, 135.7, 135.6, 135.6, 135.5, 133.5, 133.3, 133.2, 133.1, 133.0, 131.4, 131.1, 129.8, 129.7, 129.6, 129.1, 128.9, 128.8, 128.7, 128.1, 127.98, 127.8, 127.8, 127.7, 127.7, 127.6, 127.6, 127.6, 126.7, 125.5, 77.4, 65.3, 63.7, 63.2, 53.0, 52.99, 26.9, 26.7, 19.2, 19.1.

HRMS (ESI): Calcd for $C_{32}H_{35}BrNO_3Si$ $[M+H]^+$: 588.1564, found: 588.1556.

IR (neat): ν (cm^{-1}) 3069, 2953, 2858, 1710, 1441, 1315, 1109.

Rf 0.28 (Cyclohexane:AcOEt = 40:1)

Methyl (2-bromophenyl)(1-phenyl-2-((triisopropylsilyl)oxy)ethyl)carbamate (10d):

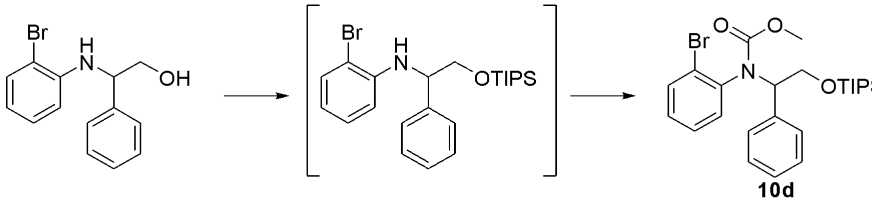

10d
Chemical Formula: $C_{25}H_{36}BrNO_3Si$
Molecular Weight: 506.56

To a solution of 2-((2-bromophenyl)amino)-2-phenylethan-1-ol (934 mg, 3.2 mmol, 1 equiv.) in DMF (11 mL, 0.3 M) was added imidazole (327 mg, 4.8 mmol, 1.5 equiv.), DMAP (39 mg, 0.32 mmol, 10 mol%) and TIPSCl (324 µL, 4.8 mmol, 1.3 equiv.) at 0 ºC and stirred at room temperature overnight. H_2O was added and extracted with Et_2O, washed with brine, dried over Na_2SO_4 anhydrous, filtered and concentrated *in vacuo*. The residue was purified by silica gel column chromatography using cyclohexane/ CH_2Cl_2 (94:6, Rf= 0.35) as a solvent to afford the pure compound 2-bromo-*N*-(1-phenyl-2-((triisopropylsilyl)oxy)ethyl)aniline as a yellow oil (1.03 g, 2.3 mmol, 72%).

Following general procedure B, a mixture of 2-bromo-*N*-(1-phenyl-2-((triisopropylsilyl)oxy)ethyl)aniline (1.31 g, 2.92 mmol, 1 equiv.) in methyl chloroformate (9 mL) was heated under reflux overnight. The mixture was concentrated *in vacuo*. The crude material was purified by flash column chromatography using cyclohexane/AcOEt as a solvent to afford the pure compound Methyl (2-bromophenyl)(1-phenyl-2-((triisopropylsilyl)oxy)ethyl)carbamate **10d** as a colorless oil (1.26 g, 2.5 mmol, 86%).

^{1}H NMR (400 MHz, Chloroform-*d*) δ 7.58 (dd, *J* = 7.7, 1.8 Hz, 0.4H), 7.51 (ddd, *J* = 8.1, 4.0, 1.6 Hz, 0.6H), 7.46 (dd, *J* = 8.0, 1.5 Hz, 0.6H), 7.41 – 7.27 (m, 2.4H), 7.25 – 6.88 (m, 5H), 5.54 (“t”, *J* = 7.4 Hz, 0.6H), 5.28 (“t”, *J* = 7.3 Hz, 0.4H), 4.50 (br, 0.4H), 4.20 (dd, *J* = 10.7, 8.8 Hz, 0.6H), 4.11 (“t”, *J* = 8.9 Hz, 0.6H), 3.91 (dd, *J* = 10.1, 7.7 Hz, 0.4H), 3.63 (s, 3H), 1.25 – 1.01 (m, 14H), 0.98 (m, 7H).

^{13}C{^{1}H} NMR (101 MHz, Chloroform-*d*) δ 156.1, 155.5, 140.0, 138.7, 137.9, 136.6, 133.3, 133.2, 131.3, 129.1, 129.0, 128.7, 128.4, 128.1, 128.1, 128.1, 127.9, 127.8, 127.7, 126.8, 125.5, 65.7, 63.7, 63.4, 62.7, 53.2, 53.1, 18.1, 18.1, 18.0, 17.99, 12.1, 12.0.

HRMS (ESI): Calcd for $C_{25}H_{37}BrNO_3Si$ $[M+H]^+$: 506.1721, found: 506.1717.

IR (neat): ν (cm^{-1}) 2944, 2866, 1712, 1586, 1442, 1315, 1194, 1114.

Rf 0.4 (Cyclohexane:AcOEt = 96:4)

Ethyl (2-bromophenyl)(1-phenyl-2-((triisopropylsilyl)oxy)ethyl)carbamate (10e):

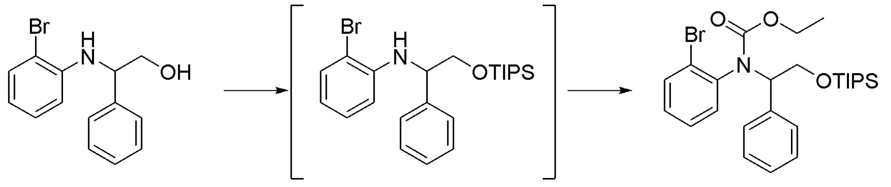

10e
Chemical Formula: $C_{26}H_{38}BrNO_3Si$
Molecular Weight: 520.58

To a solution of 2-((2-bromophenyl)amino)-2-phenylethan-1-ol (934 mg, 3.2 mmol, 1 equiv.) in DMF (11 mL, 0.3 M) was added imidazole (327 mg, 4.8 mmol, 1.5 equiv.), DMAP (39 mg, 0.32 mmol, 10 mol%) and TIPSCl (324 µL, 4.8 mmol, 1.3 equiv.) at 0 ºC and stirred at room temperature overnight. H_2O was added and extracted with Et_2O, washed with brine, dried over Na_2SO_4 anhydrous, filtered and concentrated *in vacuo*. The residue was purified by silica gel column chromatography using cyclohexane/ CH_2Cl_2 (94:6, Rf= 0.35) as a solvent to afford the pure compound 2-bromo-*N*-(1-phenyl-2-((triisopropylsilyl)oxy)ethyl) aniline as a yellow oil (1.03 g, 2.3 mmol, 72%).

Following general procedure B, a mixture of 2-bromo-*N*-(1-phenyl-2-((triisopropylsilyl)oxy)ethyl)aniline (686 mg, 1.53 mmol, 1 equiv.) in ethyl chloroformate (6 mL) was heated under reflux overnight. The mixture was concentrated *in vacuo*. The crude material was purified by flash column chromatography using cyclohexane/AcOEt as a solvent to afford the pure compound Ethyl (2-bromophenyl)(1-phenyl-2-((triisopropylsilyl)oxy)ethyl)carbamate **10e** as a pale yellow oil (760 mg, 1.46 mmol, 95%).

^{1}H NMR (400 MHz, Chloroform-*d*) δ 7.61 – 7.54 (m, 0.4H), 7.51 (dd, *J* = 7.7, 1.6 Hz, 0.6H), 7.46 (dd, *J* = 8.0, 1.4 Hz, 0.6H), 7.40 – 6.99 (m, 7H), 6.92 (d, *J* = 7.0 Hz, 0.4H), 5.55 (br, 0.6H), 5.29 (“t”, *J* = 7.4 Hz, 0.4H), 4.49 (br, 0.4H), 4.28 – 3.97 (m, 3.2H), 3.90 (dd, *J* = 10.1, 7.7 Hz, 0.4H), 1.21 – 1.02 (m, 17H), 0.97 (dd, *J* = 6.4, 3.3 Hz, 7H).

^{13}C{^{1}H} NMR (101 MHz, Chloroform-*d*) δ 155.5, 154.96, 140.2, 138.5, 137.97, 136.6, 133.1, 133.0, 131.3, 129.1, 128.9, 128.8, 128.6, 128.0, 127.99, 127.9, 127.7, 127.6, 127.5, 126.7, 125.4, 65.4, 63.4, 62.7, 61.8, 18.0, 18.0, 17.9, 17.9, 14.6, 14.6, 12.0, 11.9.

HRMS (ESI): Calcd for $C_{26}H_{39}BrNO_3Si$ $[M+H]^+$: 520.1877, found: 520.1877.

IR (neat): ν (cm^{-1}) 2943, 2866, 1708, 1856, 1484, 1404, 1295, 1113.

Rf 0.24 (Cyclohexane:AcOEt = 98:2)

2-((2-bromophenyl)(methoxycarbonyl)amino)-2-phenylethyl pivalate (10f):

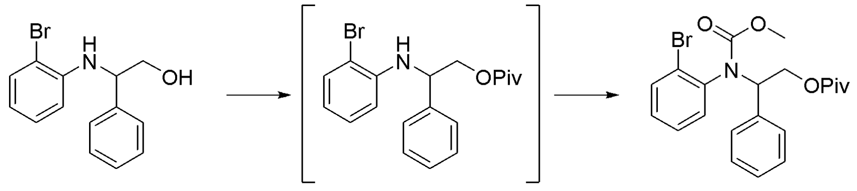

10f
Chemical Formula: $C_{21}H_{24}BrNO_4$
Molecular Weight: 434.33

To a solution of 2-((2-bromophenyl)amino)-2-phenylethan-1-ol (250 mg, 0.856 mmol, 1 equiv.) in THF (3 mL, 0.3 M) was added DIPEA (598 µL, 3.42 mmol, 4 equiv.)and PivCl (211 µL, 1.71 mmol, 2 equiv.) at 0 ºC. After 30 min at that temperature, DMAP (10.5 mg, 0.0856 mmol, 10 mol%) was added and stirred at room temperature overnight. NH_4Cl aq was added and extracted with AcOEt, washed with brine, dried over Na_2SO_4 anhydrous, filtered and concentrated *in vacuo*. The residue was purified by silica gel column chromatography using cyclohexane/AcOEt as solvent to afford the pure compound 2-((2-bromophenyl)amino)-2-phenylethyl pivalate (322 mg, 0.856 mmol, 100%).

Following general procedure B, a mixture of 2-((2-bromophenyl)amino)-2-phenylethyl pivalate (322 mg, 0.856 mmol, 1 equiv.) in methyl chloroformate (3 mL) was heated under reflux overnight. The mixture was concentrated *in vacuo*. The crude material was purified by flash column chromatography using cyclohexane/AcOEt as a solvent to afford the pure compound 2-((2-bromophenyl)(methoxycarbonyl)amino)-2-phenylethyl pivalate **10f** as a white solid (331 mg, 0.762 mmol, 89%).

^{1}H NMR (500 MHz, Chloroform-*d*) δ 7.61 (dd, *J* = 7.9, 1.6 Hz, 0.4H), 7.44 (dd, *J* = 8.0, 1.5 Hz, 0.6H), 7.38 – 7.33 (m, 0.6H), 7.31 – 7.21 (m, 3.6H), 7.19 – 7.14 (m, 1.8H), 7.11 ("t"d, *J* = 7.7, 1.8 Hz, 0.4H), 7.06 ("t"d, *J* = 7.6, 1.6 Hz, 0.4H), 7.02 – 6.96 (m, 1.2H), 6.53 (dd, *J* = 7.8, 1.7 Hz, 0.4H), 5.81 (br, 1H), 4.67 – 4.52 (m, 1.6H), 4.53 – 4.39 (m, 0.4H), 3.80 – 3.52 (m, 3H), 1.17 (s, 5.4H), 1.03 (s, 3.6H).

^{13}C{^{1}H} NMR (101 MHz, Chloroform-*d*) δ 178.3, 178.1, 138.1, 137.5, 134.8, 133.6, 133.5, 130.9, 129.4, 129.4, 129.2, 128.9, 128.5, 128.5, 128.3, 128.3, 128.2, 127.7, 126.9, 125.9, 63.5, 62.8, 60.6, 60.2, 53.5, 53.4, 38.9, 38.7, 27.2, 27.1.

HRMS (ESI): Calcd for $C_{21}H_{24}BrNNaO_4$ $[M+Na]^+$: 456.0781, found: 456.0789.

IR (neat): ν (cm^{-1}) 2969, 0703, 1442, 1388, 1282, 1138, 1033.

Rf 0.33 (Cyclohexane:AcOEt = 10:1)

Melting point: 74 ºC

Methyl (2-bromophenyl)(2-(*tert*-butoxy)-1-phenylethyl)carbamate (10g):

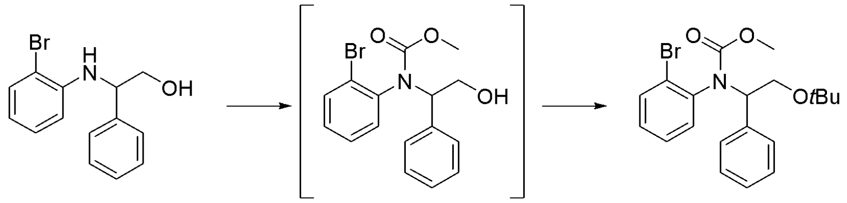

10g
Chemical Formula: $C_{20}H_{24}BrNO_3$
Molecular Weight: 406.32

Following general procedure B, a mixture of 2-((2-bromophenyl)amino)-2-phenylethan-1-ol (1.75 g, 6 mmol, 1 equiv.) in methyl chloroformate (18 mL) was heated under reflux overnight. The mixture was concentrated *in vacuo*. The crude material was purified by flash column chromatography using cyclohexane/AcOEt (2:1, Rf= 0.25) as a solvent to afford the pure compound methyl (2-bromophenyl)(2-hydroxy-1-phenylethyl)carbamate as a white solid (1.94 g, 5.54 mmol, 92%).

In a two-necked flask equipped with a magnetic stirring bar and a condenser coil, $Mg(ClO_4)_2$ (47.5 mg, 0.197 mmol, 10 mol%) and methyl (2-bromophenyl)(2-hydroxy-1-phenylethyl)carbamate (689 mg, 1.97 mmol, 1 equiv.) were dissolved in CH_2Cl_2 (6 mL, 0.3 M). Then Boc_2O (989 mg, 4.53 mmol, 2.3 equiv.) was added, and bubbling was immediately observed. The mixture was stirred at reflux overnight. The crude mixture was diluted with water and extracted with CH_2Cl_2. The organic layer was separated, dried over Na_2SO_4 anhydrous, filtered and concentrated *in vacuo.* The crude material was purified by flash column chromatography using cyclohexane/AcOEt as a solvent to afford the pure compound Methyl (2-bromophenyl)(2-(*tert*-butoxy)-1-phenylethyl)carbamate **10g** as a colorless oil (562 mg, 1.38 mmol, 70%).[3]

^{1}H NMR (400 MHz, Chloroform-*d*) δ 7.58 (dd, *J* = 7.9, 1.6 Hz, 0.5H), 7.50 – 7.37 (m, 2H), 7.30 (m, 2H), 7.25 – 7.03 (m, 4.5H), 5.38 (“t”, *J* = 6.8 Hz, 0.5H), 5.26 (“t”, *J* = 7.1 Hz, 0.5H), 4.11 (br, 0.5H), 3.98 – 3.79 (m, 1H), 3.65 (s, 3H), 3.59 (dd, *J* = 9.4, 6.4 Hz, 0.5H), 1.22 (s, 4.5H), 1.08 (s, 4.5H).

^{13}C{^{1}H} NMR (101 MHz, Chloroform-*d*) δ 155.9, 155.4, 140.1, 139.1, 138.5, 137.3, 133.2, 133.0, 131.2, 130.8, 129.1, 128.8, 128.6, 128.5, 128.1, 127.97, 127.8, 127.6, 127.6, 127.5, 126.2, 125.7, 73.4, 73.2, 64.3, 62.98, 62.3, 61.2, 53.1, 53.0, 27.6, 27.3.

HRMS (ESI): Calcd for $C_{20}H_{25}BrNO_3$ $[M+H]^+$: 406.1012, found: 406.1004.

IR (neat): ν (cm^{-1}) 2973, 1709, 1586, 1441, 1364, 1308, 1192, 1083, 1028.

Rf 0.25 (Cyclohexane:AcOEt = 20:1)

Methyl (2-(benzyloxy)-1-phenylethyl)(2-bromophenyl)carbamate (10h):

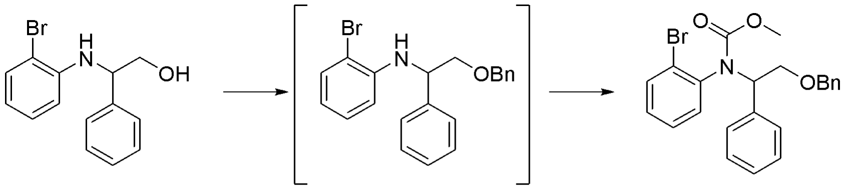

10h
Chemical Formula: $C_{23}H_{22}BrNO_3$
Molecular Weight: 440.34

To a solution of 2-((2-bromophenyl)amino)-2-phenylethan-1-ol (438 mg, 1.5 mmol, 1 equiv.) in THF (5 mL, 0.3 M) was added NaH (120 mg, 3 mmol, 2 equiv.) and BnBr (215 µL, 1.8 mmol, 1.2 equiv.) at 0 ºC and stirred at room temperature for three hours. NH_4Cl aq was added and extracted with AcOEt, washed with brine, dried over Na_2SO_4 anhydrous, filtered and concentrated *in vacuo*. The residue was purified by silica gel column chromatography using cyclohexane/AcOEt (99:1, Rf= 0.25) as solvent to afford the pure compound *N*-(2-(benzyloxy)-1-phenylethyl)-2-bromoaniline as a yellow oil (514 mg, 1.34 mmol, 90%).

Following general procedure B, a mixture of *N*-(2-(benzyloxy)-1-phenylethyl)-2-bromoaniline **2.7** (514 mg, 1.34 mmol, 1 equiv.) in methyl chloroformate (6 mL) was heated under reflux overnight. The mixture was concentrated *in vacuo*. The crude material was purified by flash column chromatography using cyclohexane/AcOEt as a solvent to afford the pure compound methyl (2-(benzyloxy)-1-phenylethyl)(2-bromophenyl)carbamate **10h** as a colorless oil (570 mg, 1.29 mmol, 97%).

^{1}H NMR (400 MHz, Chloroform-*d*) δ 7.58 (dd, *J* = 6.0, 3.5 Hz, 0.4H), 7.45 (dd, *J* = 8.0, 1.5 Hz, 0.6H), 7.40 – 7.00 (m, 12.6H**),** 6.81 – 6.72 (m, 0.4H), 5.74 (br, 0.6H), 5.59 (“t”, *J* = 7.1 Hz, 0.4H), 4.72 (d, *J* = 12.0 Hz, 0.6H), 4.66 (d, *J* =12.0Hz, 0.6H), 4.50 (d, *J* = 12.1 Hz, 0.4H), 4.45 (d, J = 12.1 Hz, 0.4H), 4.22 – 4.11 (br, 0.4H), 4.07 (dd, *J* = 10.6, 8.8 Hz, 0.6H), 3.85 (dd, *J* = 10.6, 5.8 Hz, 0.6H), 3.79 (dd, *J* = 9.9, 7.1 Hz, 0.4H), 3.75 – 3.53 (brs, 3H).

^{13}C{^{1}H} NMR (101 MHz, Chloroform-*d*) δ 156.1, 155.8, 139.3, 138.6, 138.1, 137.8, 136.3, 133.3, 133.3, 131.3, 129.1, 128.8, 128.5, 128.4, 128.4, 128.3, 128.2, 128.0, 127.96, 127.9, 127.8, 127.7, 127.7, 127.6, 126.6, 125.8, 73.2, 72.96, 69.9, 69.3, 62.4, 60.9, 53.3, 53.3.

HRMS (ESI): Calcd for $C_{23}H_{23}BrNO_3$ $[M+H]^+$: 440.0856, found: 440.0850.

IR (neat): ν (cm^{-1}) 2941, 2861, 1706, 1585, 1441, 1331, 1091, 1029.

Rf 0.3 (Cyclohexane:AcOEt = 10:1)

Methyl (2-bromophenyl)(2-((4-methoxybenzyl)oxy)-1-phenylethyl)carbamate (10i):

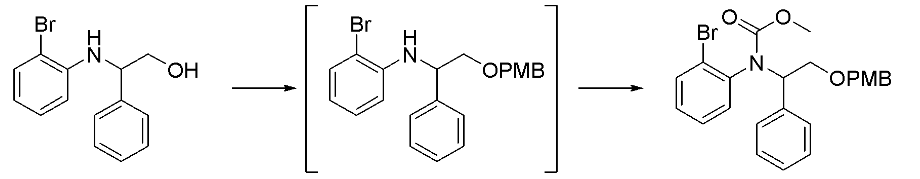

10i
Chemical Formula: $C_{24}H_{24}BrNO_4$
Molecular Weight: 470.36

To a solution of 2-((2-bromophenyl)amino)-2-phenylethan-1-ol (438 mg, 1.5 mmol, 1 equiv.) in THF (5 mL, 0.3 M) was added NaH (120 mg, 3 mmol, 2 equiv.) and PMBBr (245 µL, 1.8 mmol, 1.2 equiv.) at 0 ºC and stirred at room temperature for three hours. NH_4Cl aq was added and extracted with AcOEt, washed with brine, dried over Na_2SO_4 anhydrous, filtered and concentrated *in vacuo*. The residue was purified by silica gel column chromatography using cyclohexane/AcOEt (94:6, Rf= 0.43) as a solvent to afford the pure compound 2-bromo-*N*-(2-((4-methoxybenzyl)oxy)-1-phenylethyl)aniline as a yellow oil (445 mg, 1.08mmol, 72%).

Following general procedure B, a mixture of 2-bromo-*N*-(2-((4-methoxybenzyl)oxy)-1-phenylethyl)aniline (445 mg, 1.08 mmol, 1 equiv.) in methyl chloroformate (3 mL) was heated under reflux overnight. The mixture was concentrated *in vacuo*. The crude material was purified by flash column chromatography using cyclohexane/AcOEt as a solvent to afford the pure compound methyl (2-bromophenyl)(2-((4-methoxybenzyl)oxy)-1-phenylethyl)carbamate **10i** as a colorless oil (468 mg, 0.995 mmol, 92%).

^{1}H NMR (400 MHz, Chloroform-*d*) δ 7.56 (d”t”, *J* = 7.5, 3.7 Hz, 0.4H), 7.42 (dd, *J* = 8.0, 1.5 Hz, 0.6H), 7.37 – 6.97 (m, 9.8H), 6.91 – 6.84 (m, 1.2H), 6.84 – 6.78 (m, 0.6H), 6.78 – 6.69 (m, 0.4H), 5.72 (br, *J* = 9.7 Hz, 0.6H), 5.56 (t, *J* = 7.1 Hz, 0.4H), 4.64 (d, *J* = 11.7 Hz, 0.6H), 4.52 (d, *J* = 11.7 Hz, 0.6H), 4.41 (d, *J* = 11.7 Hz, 0.4H), 4.33 (d, *J* = 11.6 Hz, 0.4H), 4.09 (br, 0.4H), 4.01 (dd, *J* = 10.6, 8.9 Hz, 0.6H), 3.81-3.75 (br, 0.6H), 3.79 (s, 1.8H), 3.77 (s, 1.2H), 3.73 (dd, *J* = 9.9, 7.2 Hz, 0.4H), 3.70 – 3.56 (s, 3H).

^{13}C{^{1}H} NMR (101 MHz, Chloroform-*d*) δ 159.4, 159.2, 156.0, 155.7, 139.1, 138.6, 137.7, 136.3, 133.3, 133.3, 131.3, 130.2, 129.7, 129.4, 129.1, 128.8, 128.4, 128.2, 128.2, 127.9, 127.9, 127.7, 126.7, 125.8, 113.9, 113.8, 72.8, 72.6, 69.5, 69.2, 62.3, 60.9, 55.4, 55.4, 53.3, 53.3.

HRMS (ESI): Calcd for $C_{24}H_{25}BrNO_4$ $[M+H]^+$: 470.0961, found: 470.0953.

IR (neat): ν (cm-1) 2952, 2857, 1706, 1612, 1513, 1441, 1389, 1312, 1246, 1175, 1087, 1030.

Rf 0.13 (Cyclohexane:AcOEt = 91:9)

Methyl (2-bromophenyl)(2-(methoxymethoxy)-1-phenylethyl)carbamate (10j):

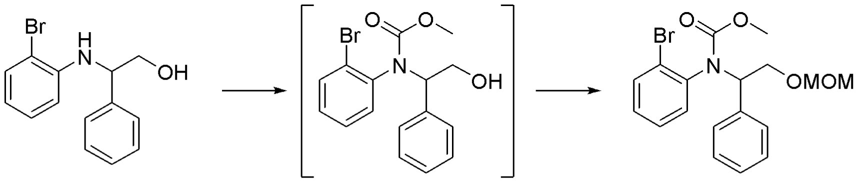

10j
Chemical Formula: $C_{18}H_{20}BrNO_4$
Molecular Weight: 394.27

Following general procedure B, a mixture of 2-((2-bromophenyl)amino)-2-phenylethan-1-ol (1.75 g, 6 mmol, 1 equiv.) in methyl chloroformate (18 mL) was heated under reflux overnight. The mixture was concentrated *in vacuo*. The crude material was purified by flash column chromatography using cyclohexane/AcOEt (2:1, R= 0.25) as a solvent to afford the pure compound methyl (2-bromophenyl)(2-hydroxy-1-phenylethyl)carbamate as a white solid (1.94 g, 5.54 mmol, 92%).

To a solution of methyl (2-bromophenyl)(2-hydroxy-1-phenylethyl)carbamate (700 mg, 2 mmol, 1 equiv.) was added DIPEA (2.1 mL, 12 mmol, 6 equiv.) and MOMBr (653 µL, 8 mmol, 4 equiv.) at 0 ºC and stirred at room temperature for 4 h. After reaction completion, NH_4Cl aq was added and extracted with CH_2Cl_2. The organic layer was separated, dried over Na_2SO_4 anhydrous, filtered and concentrated *in vacuo.* The crude material was purified by flash column chromatography using cyclohexane/AcOEt as a solvent to afford the pure compound methyl (2-bromophenyl)(2-(methoxymethoxy)-1-phenylethyl)carbamate **10j** as a colorless oil (618 mg, 1.57 mmol, 78%).

^{1}H NMR (400 MHz, Chloroform-*d*) δ 7.66 – 7.57 (m, 0.4H), 7.53 – 7.42 (m, 0.6H), 7.38 – 7.27 (m, 3H), 7.25 – 7.01 (m, 4.6H), 6.74 – 6.61 (m, 0.4H), 5.63 (“t”, *J* = 7.2 Hz, 1H), 4.76 – 4.67 (m, 1.2H), 4.55 (m, 0.8H), 4.14 (dd, *J* = 10.5, 8.2 Hz, 0.8H), 4.03 (dd, *J* = 10.5, 6.7 Hz, 0.6H), 3.88 (dd, *J* = 10.1, 8.0 Hz, 0.6H), 3.66 (s, 3H), 3.35 (s, 1.8H), 3.23 (s, 1.2H).

^{13}C{^{1}H} NMR (101 MHz, Chloroform-*d*) δ 155.9, 155.6, 138.7, 138.3, 137.8, 135.98, 133.3, 133.2, 131.1, 131.0, 129.1, 128.9, 128.8, 128.4, 128.2, 128.1, 127.99, 127.8, 127.6, 126.5, 125.7, 96.6, 96.4, 67.6, 66.6, 61.8, 61.2, 55.6, 55.5, 53.2, 53.2.

HRMS (ESI): Calcd for $C_{18}H_{20}BrNNaO_4$ $[M+Na]^+$: 416.0468, found: 416.0476.

IR (neat): ν (cm^{-1}) 2951, 2886, 1706, 1585, 1441, 1308, 1193, 1149, 1110, 1030.

Rf 0.5 (Cyclohexane:AcOEt = 3:1)

Methyl (2-(benzyloxy)-1-phenylethyl)(2-iodophenyl)carbamate (10k):

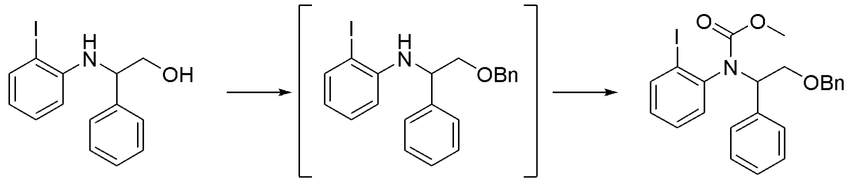

10k
Chemical Formula: $C_{23}H_{22}INO_3$
Molecular Weight: 487.34

To a solution of 2-((2-iodophenyl)amino)-2-phenylethan-1-ol (2.78 g, 8.2 mmol, 1 equiv.) in THF (27 mL, 0.3 M) was added NaH (394 mg, 16.4 mmol, 2 equiv.) and BnBr (1.3 mL, 10.7 mmol, 1.3 equiv.) at 0 ºC and stirred at room temperature for three hours. NH_4Cl aq was added and extracted with AcOEt, washed with brine, dried over Na_2SO_4 anhydrous, filtered and concentrated *in vacuo*. The residue was purified by silica gel column chromatography using cyclohexane/AcOEt as solvent to afford the pure compound *N*-(2-(benzyloxy)-1-phenylethyl)-2-iodoaniline as a yellow oil (2.99 g, 6.96 mmol, 85%).

Following general procedure B, a mixture of *N*-(2-(benzyloxy)-1-phenylethyl)-2-iodoaniline (1.50 g, 3.48 mmol, 1 equiv.) in methyl chloroformate (12 mL) was heated under reflux overnight. The mixture was concentrated *in vacuo*. The crude material was purified by flash column chromatography using cyclohexane/AcOEt as a solvent to afford the pure compound methyl (2-(benzyloxy)-1-phenylethyl)(2-iodophenyl)carbamate **10k** as a colorless oil (1.1 g, 2.28 mmol, 65%).

^{1}H NMR (500 MHz, Chloroform-*d*) δ 7.85 (dd, *J* = 7.9, 1.5 Hz, 0.4H), 7.74 (dd, *J* = 7.9, 1.5 Hz, 0.6H), 7.43 – 7.23 (m, 8H), 7.22 – 7.15 (m, 2.4H), 7.06 – 7.01 (m, 1.2H), 6.99 – 6.93 (m, 1H), 6.85 (dd, *J* = 7.9, 1.6 Hz, 0.4H), 5.71 (br, 0.6H), 5.53 ("t", *J* = 7.1 Hz, 0.4H), 4.73 (d, *J* = 12.1 Hz, 0.6H), 4.61 (d, *J* = 12.0 Hz, 0.6H), 4.49 (d, *J* = 12.1 Hz, 0.4H), 4.41 (d, *J* = 12.1 Hz, 0.4H), 4.19 (br, 0.4H), 4.09 (dd, *J* = 10.6, 8.8 Hz, 0.6H), 3.84 (dd, *J* = 10.6, 5.8 Hz, 0.6H), 3.76 (dd, *J* = 9.9, 7.0 Hz, 0.4H), 3.68 (s, 3H).

^{13}C{^{1}H} NMR (101 MHz, Chloroform-*d*) δ 155.9, 155.5, 143.0, 141.1, 139.8, 139.7, 138.7, 138.1, 138.1, 136.2, 130.6, 130.4, 129.9, 129.9, 129.7, 129.5, 129.1, 128.8, 128.7, 128.5, 128.4, 128.4, 128.3, 128.3, 128.0, 127.98, 127.8, 127.7, 127.6, 103.95, 102.8, 73.2, 72.95, 70.2, 69.6, 62.8, 61.2, 53.3, 53.3.

HRMS (ESI): Calcd for $C_{23}H_{23}INO_3$ $[M+H]^+$: 488.0717, found: 488.0709.

IR (neat): ν (cm^{-1}) 3031, 2951, 2862, 1704, 1441, 1313, 1194, 1093, 1020.

Rf 0.28 (Cyclohexane:AcOEt = 10:1)

_N_-(2-(benzyloxy)-1-phenylethyl)-_N_-(2-bromophenyl)-2,2,2-trifluoroacetamide (10l):

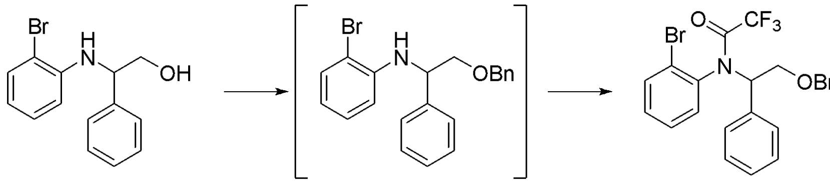

10l
Chemical Formula: $C_{23}H_{19}BrF_3NO_2$
Molecular Weight: 478.31

To a solution of 2-((2-bromophenyl)amino)-2-phenylethan-1-ol (438 mg, 1.5 mmol, 1 equiv.) in THF (5 mL, 0.3 M) was added NaH (120 mg, 3 mmol, 2 equiv.) and BnBr (215 µL, 1.8 mmol, 1.2 equiv.) at 0 ºC and stirred at room temperature for three hours. NH_4Cl aq was added and extracted with AcOEt, washed with brine, dried over Na_2SO_4 anhydrous, filtered and concentrated *in vacuo*. The residue was purified by silica gel column chromatography using cyclohexane/AcOEt (99:1, Rf= 0.25) as a solvent to afford the pure compound *N*-(2-(benzyloxy)-1-phenylethyl)-2-bromoaniline as a yellow oil (514 mg, 1.34 mmol, 90%).

Following general procedure B, a mixture of *N*-(2-(benzyloxy)-1-phenylethyl)-2-bromoaniline (3.08 g, 8.05 mmol, 1 equiv.) in methyl chloroformate (26 mL) was heated under reflux overnight. The mixture was concentrated *in vacuo*. The crude material was purified by flash column chromatography using cyclohexane/AcOEt as a solvent to afford the pure compound *N*-(2-(benzyloxy)-1-phenylethyl)-*N*-(2-bromophenyl)-2,2,2-trifluoroacetamide **10l** as a yellow oil (3.49 g, 7.3 mmol, 91%).

^{1}H NMR (400 MHz, Chloroform-*d*) δ 7.61 (dd, *J* = 7.9, 1.6 Hz, 0.3H), 7.52 – 7.45 (m, 0.7H), 7.41 – 7.08 (m, 11.3H), 7.05 – 6.98 (m, 1.4H), 6.85 (d, *J* = 8.1 Hz, 0.3H), 5.94 (dd, *J* = 8.7, 5.6 Hz, 0.7H), 5.71 ("t", *J* = 7.0 Hz, 0.3H), 4.71 (d, *J* = 12.0 Hz, 0.7H), 4.57 (d, *J* = 11.9 Hz, 0.7H), 4.51 (d, *J* = 12.0 Hz, 0.3H), 4.42 (d, *J* = 12.0 Hz, 0.3H), 4.25 (dd, *J* = 10.0, 6.8 Hz, 0.3H), 4.09 (dd, *J* = 10.7, 8.7 Hz, 0.7H), 3.92 (dd, *J* = 10.7, 5.6 Hz, 0.7H), 3.85 (dd, *J* = 10.0, 7.0 Hz, 0.3H).

^{13}C{^{1}H} NMR (126 MHz, Chloroform-*d*) δ 157.7 (q, *J* = 35.9 Hz), 137.8, 136.7, 136.4, 134.98, 134.4, 133.6, 133.4, 133.0, 132.5, 130.9, 130.6, 129.8, 129.3, 128.99, 128.6, 128.6, 128.5, 128.5, 127.98, 127.95, 127.8, 127.7, 127.6, 126.96, 125.8, 116.2 (q, *J* = 289.2 Hz), 73.3, 73.1, 69.2, 68.8, 64.8, 62.1.

^{19}F{^{1}H} NMR (376 MHz, Chloroform-d) δ -68.71, -68.75.

HRMS (ESI): Calcd for $C_{23}H_{20}BrF_3NO_2$ $[M+H]^+$: 478.0624, found: 478.0619.

IR (neat): ν (cm^{-1}) 3034, 2866, 1696, 1585, 1475, 1410, 1365, 1181, 1121, 699, 632.

Rf 0.18 (Cyclohexane:AcOEt = 60:1)

C–H Activation Products of Model Substrates via Parallel Kinetic Resolution

Methyl 6-(((*tert*-butyldimethylsilyl)oxy)methyl)phenanthridine-5(6H)-carboxylate (12a):

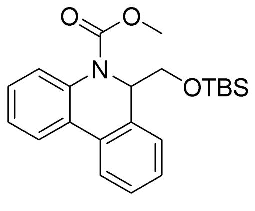

Chemical Formula: $C_{22}H_{29}NO_3Si$
Molecular Weight: 383.56

Following general procedure C (with IBioxtBu as chiral ligand), methyl (2-bromophenyl)(2-((*tert*-butyldimethylsilyl)oxy)-1-phenylethyl)carbamate **10a** (0.2 mmol) was engaged. The residue was purified by preparative thin-layer chromatography (17 mg, 0.0443 mmol, 22%) as a colorless oil.

^{1}H NMR (400 MHz, Chloroform-*d*) δ 8.01 – 7.46 (m, 3H), 7.38 (ddd, *J* = 7.8, 5.9, 2.9 Hz, 1H), 7.36 – 7.25 (m, 3H), 7.22 ("t"d, *J* = 7.6, 1.3 Hz, 1H), 5.62 (br, 1H), 3.80 (s, 3H), 3.61 – 3.42 (m, 2H), 0.81 (s, 9H), -0.16 (s, 3H), -0.19 (s, 3H).

^{13}C{^{1}H} NMR (126 MHz, Chloroform-*d*) δ 154.9, 134.9, 131.3, 128.5, 128.1, 127.6, 125.7, 124.9, 123.7, 123.4, 63.5, 58.1, 53.1, 25.9, 18.2, -5.5, -5.7.

HRMS (ESI): Calcd for $C_{22}H_{30}NO_3Si$ $[M+H]^+$: 384.1989, found: 384.1985.

IR (neat): ν (cm^{-1}) 2954, 2857, 17120, 1605, 1440, 1392, 1330, 1255, 115, 1116, 1072.

Rf 0.28 (Cyclohexane:CH_2Cl_2 = 3:2)

$[\alpha]_D^{20}$: +17.6 ° (c = 0.199, $CHCl_3$)

HPLC separation Chiralcel ® IC; 99:1 (*n*-heptane/*i*-PrOH), 1 ml.min^{-1}, 227 nm, t_R (major) = 21.7 min, t_R (minor) = 30.4 min, 78:22 e.r.

Methyl 6-(((triethylsilyl)oxy)methyl)phenanthridine-5(6H)-carboxylate (12b):

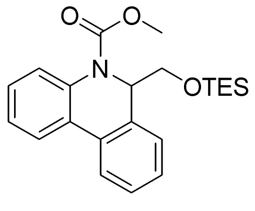

Chemical Formula: $C_{22}H_{29}NO_3Si$
Molecular Weight: 383.56

Following general procedure C (with IBioxtBu as chiral ligand), methyl (2-bromophenyl)(2-((triethylsilyl)oxy)-1-phenylethyl)carbamate **10b** was engaged. The crude was purified by preparative thin-layer chromatography (5.2 mg, 0.0136 mmol, 14%) as a colorless oil.

^{1}H NMR (400 MHz, Chloroform-*d*) δ 7.95 – 7.50 (m, 3H), 7.38 (ddd, *J* = 7.8, 5.9, 2.9 Hz, 1H), 7.35 – 7.27 (m, 3jH), 7.22 ("t"d, *J* = 7.6, 1.3 Hz, 1H), 5.61 (br, 1H), 3.79 (s, 3H), 3.57 – 3.46 (m, 2H), 0.82 (t, *J* = 8.0 Hz, 9H), 0.43 (q, *J* = 8.0 Hz, 6H).

$^{13}C\{^{1}H\}$ NMR (126 MHz, Chloroform-*d*) δ 154.9, 134.9, 131.2, 128.5, 128.1, 127.6, 125.8, 124.98, 123.7, 123.4, 63.3, 58.2, 53.2, 6.7, 4.4.

HRMS (ESI): Calcd for $C_{22}H_{30}NO_3Si$ $[M+H]^+$: 384.1989, found: 384.1988.

IR (neat): ν (cm^{-1}) 2955, 2910, 1711, 1605, 1441, 1391, 1330, 1258, 1195, 1156, 1072, 1013.

Rf 0.4 (Cyclohexane : CH_2Cl_2 = 3 : 2)

$[\alpha]_D^{20}$: -54.7 º (c = 0.167, $CHCl_3$)

HPLC separation Chiralcel® IC; 99:1 (*n*-heptane/*i*-PrOH), 1 ml.min^{-1}, 240 nm, t_R (major) = 14.6 min, t_R (minor) = 16.9 min, 67:33 e.r.

Methyl 6-(((triisopropylsilyl)oxy)methyl)phenanthridine-5(6H)-carboxylate (12d):

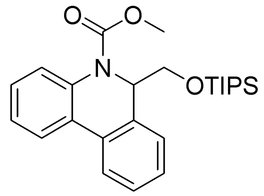

Chemical Formula: $C_{25}H_{35}NO_3Si$
Molecular Weight: 425.64

Following general procedure C (with IBioxtBu as chiral ligand), methyl (2-bromophenyl)(1-phenyl-2-((triisopropylsilyl)oxy)ethyl)carbamate **10d** was engaged. The crude was purified by preparative thin-layer chromatography (18.3 mg, 0.043 mmol, 43%) as a white solid.

^{1}H NMR (400 MHz, Chloroform-*d*) δ 7.96 – 7.51 (m, 3H), 7.38 (ddd, *J* = 7.9, 5.2, 3.7 Hz, 1H), 7.35 – 7.27 (m, 3H), 7.21 ("t"d, *J* = 7.5, 1.3 Hz, 1H), 5.65 (br, 1H), 3.80 (s, 3H), 3.65 – 3.54 (m, 2H), 1.02 – 0.85 (m, 21H).

$^{13}C\{^{1}H\}$ NMR (101 MHz, Chloroform-*d*) δ 154.9, 134.9, 131.3, 128.4, 128.0, 127.6, 125.7, 124.9, 123.6, 123.4, 63.9, 58.3, 53.1, 17.95, 12.0.

HRMS (ESI): Calcd for $C_{25}H_{36}NO_3Si$ $[M+H]^+$: 426.2459, found: 426.2454.

IR (neat): ν (cm^{-1}) 2944, 2866, 1712, 1440, 1391, 1330, 1257, 1195, 1121, 1071.

Rf 0.13 (Cyclohexane:CH_2Cl_2 = 85:15)

$[\alpha]_D^{20}$: -10.8 º (c = 0.176, $CHCl_3$)

HPLC separation Chiralcel® IC; 99:1 (*n*-heptane/*i*-PrOH), 1 ml.min^{-1}, 270 nm, t_R (major) = 13.1 min, t_R (minor) = 15.7 min, 84:16 e.r.

Melting point: 61.5 ºC

Ethyl 6-(((triisopropylsilyl)oxy)methyl)phenanthridine-5(6H)-carboxylate (12e):

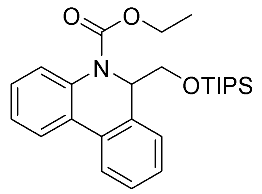

Chemical Formula: $C_{26}H_{37}NO_3Si$
Molecular Weight: 439.67

Following general procedure C (with IBioxtBu as chiral ligand), ethyl (2-bromophenyl)(1-phenyl-2-((triisopropylsilyl)oxy)ethyl)carbamate **10e** was engaged. The crude was purified by preparative thin-layer chromatography (14 mg, 0.0318 mmol, 32 %) as a white solid.

^{1}H NMR (400 MHz, Chloroform-*d*) δ 7.95 – 7.49 (m, 3H), 7.38 (ddd, *J* = 7.9, 5.8, 3.0 Hz, 1H), 7.34 – 7.26 (m, 3H), 7.25 – 7.17 (m, 1H), 5.65 (br, 1H), 4.45 – 4.13 (m, 2H), 3.67 – 3.45 (m, 2H), 1.32 (t, *J* = 7.1 Hz, 3H), 0.93 (d, *J* = 3.9 Hz, 21H).

^{13}C{^{1}H} NMR (101 MHz, Chloroform-*d*) δ 154.4, 135.1, 131.4, 130.1, 128.4, 128.3, 127.95, 127.6, 127.5, 125.8, 124.8, 123.6, 123.4, 63.9, 62.2, 58.0, 17.98, 14.7, 12.0.

HRMS (ESI): Calcd for $C_{26}H_{38}NO_3Si$ $[M+H]^+$: 440.2615, found: 440.2610.

IR (neat): ν (cm^{-1}) 2944, 2865, 1707, 1604, 1401, 1322, 1254, 1120, 1070.

Rf 0.15 (Cyclohexane : CH_2Cl_2 = 85 : 15)

$[\alpha]_D^{20}$: -145.8 º (c = 0.166, $CHCl_3$)

HPLC separation Chiralcel® IC; 99:1 (*n*-heptane/*i*-PrOH), 1 ml.min^{-1}, 270 nm, t_R (major) = 13.9 min, t_R (minor) = 19.5 min, 72:28 e.r.

Melting point: 59.9 ºC

Methyl 6-((pivaloyloxy)methyl)phenanthridine-5(6H)-carboxylate (12f):

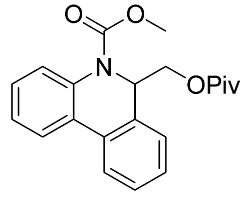

Chemical Formula: $C_{21}H_{23}NO_4$
Molecular Weight: 353.42

Following general procedure C (with IBioxtBu as chiral ligand), 2-((2-bromophenyl)(methoxycarbonyl)amino)-2-phenylethyl pivalate **10f** (0.2 mmol) was engaged. The crude was purified by preparative thin-layer chromatography (42.5 mg, 0.12 mmol, 60%) as a pale yellow oil.

^{1}H NMR (400 MHz, Chloroform-*d*) δ 7.96 – 7.48 (m, 3H), 7.41 (d"t", *J* = 7.8, 4.4 Hz, 1H), 7.38 – 7.28 (m, 3H), 7.28 – 7.20 (m, 1H), 5.98 – 5.74 (br, 1H), 4.11 (dd, *J* = 11.3, 5.0 Hz, 1H), 3.86 ("t", *J* = 10.4 Hz, 1H), 3.79 (s, 3H), 1.14 (s, 9H).

^{13}C{^{1}H} NMR (101 MHz, Chloroform-*d*) δ 178.1, 154.7, 134.3, 133.3, 131.3, 128.9, 128.3, 127.97, 127.5, 127.1, 125.8, 125.3, 123.7, 123.7, 63.7, 55.0, 53.3, 38.9, 27.2.

HRMS (ESI): Calcd for $C_{21}H_{24}NO_4$ $[M+H]^+$: 354.1700, found: 354.1695.

IR (neat): ν (cm^{-1}) 2782, 1708, 1605, 1440, 1394, 1328, 1257, 1194, 1144, 1060.

Rf 0.19 (Cyclohexane:AcOEt = 40:1)

$[\alpha]_D^{20}$: +129.7 ° (c = 0.246, $CHCl_3$)

HPLC separation Chiralcel® AD-H; 99.5:0.5 (*n*-heptane/*i*-PrOH), 1 ml.min^{-1}, 270 nm, t_R (major) = 56.3 min, t_R (minor) = 62.4 min, 77:23 e.r.

Methyl 6-(*tert*-butoxymethyl)phenanthridine-5(6H)-carboxylate (12g):

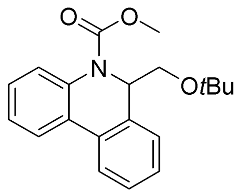

Chemical Formula: $C_{20}H_{23}NO_3$
Molecular Weight: 325.41

Following general procedure C (with IBioxtBu as chiral ligand), methyl (2-bromophenyl)(2-(*tert*-butoxy)-1-phenylethyl)carbamate **10g** (0.2 mmol) was engaged. The crude was purified by preparative thin-layer chromatography (45 mg, 0.138 mmol, 69%) as a white solid.

^{1}H NMR (400 MHz, Chloroform-*d*) δ 8.02 – 7.47 (m, 3H), 7.38 (d”t”, J = 7.8, 4.4 Hz, 1H), 7.35 – 7.27 (m, 3H), 7.22 (“t”d, J = 7.6, 1.3 Hz, 1H), 5.65 (“t”, J = 7.4 Hz, 1H), 3.80 (s, 3H), 3.31 – 3.24 (m, 2H), 0.98 (s, 9H).

^{13}C{^{1}H} NMR (101 MHz, Chloroform-*d*) δ 154.9, 134.9, 131.2, 128.8, 128.4, 127.96, 127.7, 127.6, 127.4, 125.9, 124.9, 123.6, 123.4, 73.3, 62.5, 56.8, 53.1, 27.4.

HRMS (ESI): Calcd for $C_{20}H_{23}NNaO_3$ $[M+Na]^+$: 348.1570, found: 348.1574.

IR (neat): ν (cm^{-1}) 3019, 1704, 1440, 1392, 1331, 1257, 1191, 1061.

Rf 0.28 (Cyclohexane:AcOEt = 20:1)

$[\alpha]_D^{20}$: +15.3 ° (c = 0.347, $CHCl_3$)

HPLC separation Chiralcel® IA; 99.5:0.5 (*n*-heptane/*i*-PrOH), 1 ml.min^{-1}, 270 nm, t_R (minor) = 19.4 min, t_R (major) = 20.6 min, 41:59 e.r.

Melting point: 99.6 ºC

Methyl 6-((benzyloxy)methyl)phenanthridine-5(6H)-carboxylate (12h) / Methyl 3-(benzyloxy)-2-phenylindoline-1-carboxylate (11h)

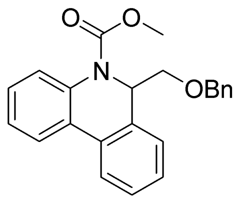

Chemical Formula: $C_{23}H_{21}NO_3$
Molecular Weight: 359.43

Following general procedure C ((with 1,3-bis((*R*)-2,2-dimethyl-1-(*o*-tolyl)propyl)-1H-imidazol-3-ium iodide as chiral ligand), **L²**), methyl (2-(benzyloxy)-1-phenylethyl)(2-bromophenyl)carbamate **10h** was engaged. The crude was purified by preparative thin-layer chromatography.

Methyl 6-((benzyloxy)methyl)phenanthridine-5(6H)-carboxylate (12h) (18.7 mg, 0.0520 mmol, 52%) as a pale yellow oil.

^{1}H NMR (400 MHz, Chloroform-*d*) δ 7.77 (d, *J* = 7.8 Hz, 1H), 7.75 (dd, *J* = 7.8, 1.6 Hz, 1H), 7.70-7.47 (m, 1H), 7.42 – 7.13 (m, 10H), 5.83 (br, 1H), 4.57 – 4.38 (m, 2H), 3.81 (s, 3H), 3.45 – 3.31 (m, 2H).

^{13}C{^{1}H} NMR (126 MHz, Chloroform-*d*) δ 154.8, 138.1, 134.6, 131.2, 128.5, 128.3, 128.1, 127.7, 127.6, 127.5, 127.4, 127.1, 125.1, 123.6, 123.5, 72.8, 70.0, 53.2, 29.7.

HRMS (ESI): Calcd for $C_{23}H_{22}NO_3$ $[M+H]^+$: 360.1594, found: 360.1590.

IR (neat): ν (cm^{-1}) 3030, 2952, 2856, 1703, 1604, 1439, 1388, 1328, 1253, 1193, 1106, 1075.

Rf 0.18 (Cyclohexane : AcOEt = 20 : 1)

$[\alpha]_D^{20}$: +154.5 ° (c = 0.935, $CHCl_3$)

HPLC separation Chiralcel ® IC; 95:5 (*n*-heptane/*i*-PrOH), 1 ml.min^{-1}, 302 nm, t_R (major) = 14.2 min, t_R (minor) = 17.1 min, 8:92 e.r.

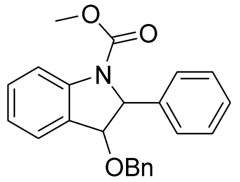

Chemical Formula: $C_{23}H_{21}NO_3$
Molecular Weight: 359.43

Methyl 3-(benzyloxy)-2-phenylindoline-1-carboxylate (11h) (16.8 mg, 0.0467 mmol, 47%) as a pale yellow oil.

^{1}H NMR (400 MHz, Chloroform-*d*) δ 8.20 – 7.52 (br, 1H), 7.42 ("t", *J* = 7.8 Hz, 1H), 7.35 (m, 5H), 7.32 – 7.20 (m, 4H), 7.16 – 7.04 (m, 3H), 5.41 (br, 1H), 4.76 – 4.71 (m, 1H), 4.69 (s, 2H), 3.75 (br, 3H).

^{13}C{^{1}H} NMR (101 MHz, Chloroform-*d*) δ 153.4, 140.1, 137.7, 130.6, 128.8, 128.6, 127.9, 127.9, 127.7, 126.7, 125.5, 123.1, 115.4, 84.6, 70.1, 69.1, 52.7.

HRMS (ESI): Calcd for $C_{23}H_{21}NNaO_3$ $[M+Na]^+$: 382.1414, found: 382.1412.

IR (neat): ν (cm^{-1}) 3031, 1708, 1604, 1441, 1383, 1342, 1263, 1140, 1053.

Rf 0.29 (Cyclohexane : AcOEt = 20 : 1)

[α]D^20: -91.4 ° (c = 0.210, $CHCl_3$)

HPLC separation Chiralcel® IA; 97:3 (*n*-heptane/*i*-PrOH), 1 ml.min^{-1}, 255 nm, t_R (minor) = 11.1 min, t_R (major) = 13.2 min, 99.8:0.2 e.r.

Methyl 6-(((4-methoxybenzyl)oxy)methyl)phenanthridine-5(6H)-carboxylate (12i) / Methyl 3-((4-methoxybenzyl)oxy)-2-phenylindoline-1-carboxylate (11i):

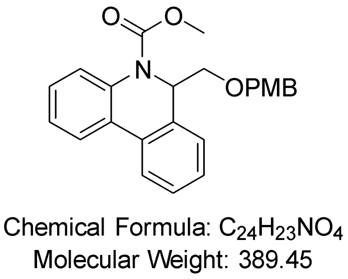

Following general procedure C (with IBioxtBu as chiral ligand), methyl (2-bromophenyl)(2-((4-methoxybenzyl)oxy)-1-phenylethyl)carbamate **10i** was engaged. The crude was purified by preparative thin-layer chromatography.

Methyl 6-(((4-methoxybenzyl)oxy)methyl)phenanthridine-5(6H)-carboxylate (12i) (23.1 mg, 0.0593 mmol, 59%) as pale yellow oil.

^{1}H NMR (400 MHz, Chloroform-*d*) δ 7.88 – 7.44 (m, 3H), 7.39 ("t"d, J = 7.5, 1.9 Hz, 1H), 7.35 – 7.26 (m, 3H), 7.23 ("t"d, J = 7.6, 1.3 Hz, 1H), 7.18 – 7.07 (m, 2H), 6.86 – 6.76 (m, 2H), 5.81 (br, 1H), 4.43 (d, J = 11.8 Hz, 1H), 4.37 (d, J = 11.7 Hz, 1H), 3.81 (s, 3H), 3.79 (s, 3H), 3.43 – 3.28 (m, 2H).

^{13}C{^{1}H} NMR (101 MHz, Chloroform-*d*) δ 159.2, 154.9, 134.6, 131.3, 130.3, 129.1, 128.5, 128.1, 127.8, 127.7, 127.2, 126.1, 125.2, 123.7, 123.6, 113.8, 72.6, 69.7, 56.0, 55.4, 53.3.

HRMS (ESI): Calcd for $C_{24}H_{24}NO_4$ $[M+H]^+$: 390.1700, found: 390.1694.

IR (neat): ν (cm^{-1}) 2954, 2862, 1705, 1611, 1513, 1441, 1331, 1251, 1104, 1037.

Rf 0.15 (Cyclohexane:AcOEt = 8:1)

[α]D^20: -16.6 ° (c = 0.169, $CHCl_3$)

HPLC separation Chiralcel ® IC; 98:2 (*n*-heptane/*i*-PrOH), 1 ml.min^{-1}, 230 nm, t_R (major) = 51.3 min, t_R (minor) = 58.2 min, 64:36 e.r.

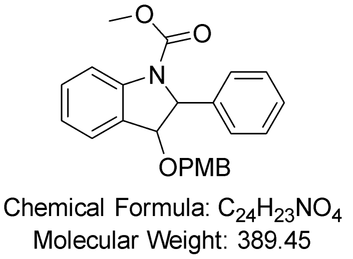

Methyl 3-((4-methoxybenzyl)oxy)-2-phenylindoline-1-carboxylate (11i) (5.6 mg, 0.0144 mmol, 14%) as a pale yellow oil

^{1}H NMR (400 MHz, Chloroform-*d*) δ 7.85 (br, 1H), 7.41 ("t", J = 7.9 Hz, 1H), 7.33 (dd, J = 7.5, 1.3 Hz, 1H), 7.30 – 7.22 (m, 5H), 7.15 – 7.04 (m, 3H), 6.91 – 6.84 (m, 2H), 5.39 (br, 1H), 4.71 (d, J = 1.0 Hz, 1H), 4.67 – 4.57 (m, 2H), 3.80 (s, 3H), 3.66 (s, 3H).

$^{13}C\{^1H\}$ **NMR** (126 MHz, Chloroform-*d*) δ 159.5, 140.3, 130.7, 129.9, 129.7, 128.9, 127.7, 126.8, 125.6, 123.2, 115.5, 114.1, 84.5, 69.9, 69.3, 55.5, 52.8.

HRMS (ESI): Calcd for $C_{24}H_{23}NNaO_4$ $[M+Na]^+$: 412.1519, found: 412.1519.

IR (neat): ν (cm^{-1}) 3030, 2950, 1708, 1609, 1442, 1386, 1247, 1177, 1031.

Rf 0.26 (Cyclohexane:AcOEt = 8:1)

$[\alpha]_D^{20}$: -89.6 ° (c = 0.280, $CHCl_3$)

HPLC separation Chiralcel ® IA; 95:5 (*n*-heptane/*i*-PrOH), 1 $ml.min^{-1}$, 250 nm, t_R (minor) = 20.0 min, *t*R (major) = 22.0 min, 1:99 e.r.

Methyl 6-((methoxymethoxy)methyl)phenanthridine-5(6H)-carboxylate (12j) / Methyl 3-(methoxymethoxy)-2-phenylindoline-1-carboxylate (11j)

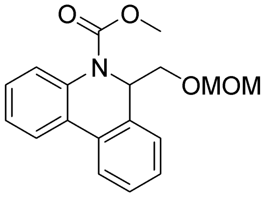

Chemical Formula: $C_{18}H_{19}NO_4$
Molecular Weight: 313.35

Following general procedure C (with IBioxtBu as chiral ligand), methyl (2-bromophenyl)(2-((4-methoxybenzyl)oxy)-1-phenylethyl)carbamate **10j** was engaged. The crude was purified by preparative thin-layer chromatography.

Methyl 6-((methoxymethoxy)methyl)phenanthridine-5(6H)-carboxylate (12j) (11.9 mg, 0.038 mmol, 38%) as white solid. Recrystallization from EtOAc/pentane afforded crystals for X-ray crystallography (98.5:1.5 e.r.).

^1H **NMR** (400 MHz, Chloroform-*d*) δ 7.87 – 7.48 (m, 3H), 7.40 (ddd, *J* = 7.8, 5.5, 3.3 Hz, 1H), 7.36 – 7.28 (m, 3H), 7.23 (dd, *J* = 7.4, 1.3 Hz, 1H), 5.78 (br, 1H), 4.57 (d, *J* = 6.6 Hz, 1H), 4.49 (d, *J* = 6.6 Hz, 1H), 3.80 (s, 3H), 3.51 – 3.36 (m, 2H), 3.23 (s, 3H).

$^{13}C\{^1H\}$ **NMR** (101 MHz, Chloroform-*d*) δ 154.8, 134.4, 131.1, 128.5, 128.1, 127.8, 127.7, 127.1, 125.9, 125.2, 123.7, 123.5, 96.1, 67.2, 55.9, 55.2, 53.2.

HRMS (ESI): Calcd for $C_{18}H_{19}NNaO_4$ $[M+Na]^+$: 336.1206, found: 336.1212.

IR (neat): ν (cm^{-1}) 2955, 1701, 1442, 1328, 1265, 1112, 1028.

Rf 0.3 (Cyclohexane:AcOEt = 6:1)

$[\alpha]_D^{20}$: -109.8 ° (c = 0.112, $CHCl_3$)

HPLC separation Chiralcel ® IC; 95:5 (*n*-heptane/*i*-PrOH), 1 $ml.min^{-1}$, 270 nm, t_R (major) = 21.5 min, t_R (minor) = 24.6 min, 69:31 e.r.

Melting point: 116.7 °C

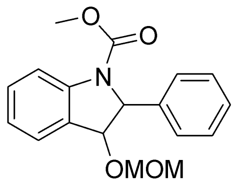

Methyl 3-(methoxymethoxy)-2-phenylindoline-1-carboxylate (11j) (8.5 mg, 0.0271 mmol, 27%) as pale yellow oil.

^{1}H NMR (400 MHz, Chloroform-*d*) δ 8.07 (br, 1H), 7.42 ("t", *J* = 7.8 Hz, 1H), 7.34 (d, *J* = 7.4 Hz, 1H), 7.32 – 7.19 (m, 3H), 7.13 (d, *J* = 7.2 Hz, 2H), 7.09 ("t"d, *J* = 7.4, 1.0 Hz, 1H), 5.40 (br, 1H), 4.85 (s, 3H), 3.68 (br, 3H), 3.47 (s, 3H).

^{13}C{^{1}H} NMR (126 MHz, Chloroform-*d*) δ 153.6, 140.0, 130.8, 128.97, 127.8, 126.9, 125.6, 123.4, 115.6, 95.2, 82.8, 69.7, 55.9, 52.9.

HRMS (ESI): Calcd for $C_{18}H_{19}NNaO_4$ $[M+Na]^+$: 336.1206, found: 336.1209.

IR (neat): ν (cm^{-1}) 2954, 1413, 1605, 1484, 1443, 1387, 1148, 1026.

Rf 0.52 (Cyclohexane:AcOEt = 6:1)

$[\alpha]_D^{20}$: +3.67 ° (c = 0.49, $CHCl_3$)

HPLC separation Chiralcel ® IA; 99:1 (*n*-heptane/*i*-PrOH), 0.5 ml.min^{-1}, 245 nm, t_R(major) = 43.9 min, t_R(minor) = 47.7 min 99.6:0.4 e.r.

Synthetic Procedures and Characterization Data

Methyl 2-amino-2-(3-(benzyloxy)-4-methoxyphenyl)acetate (6):

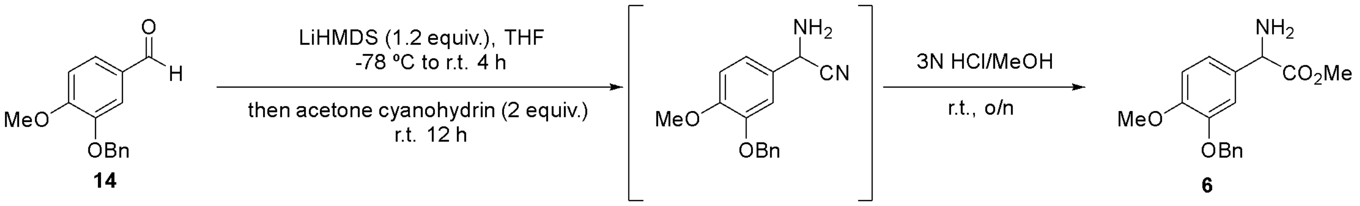

Chemical Formula: $C_{17}H_{19}NO_4$
Molecular Weight: 301.3420

3-(benzyloxy)-4-methoxybenzaldehyde **14** (4 g, 16.5 mmol, 1 equiv.) was dissolved in anhydrous THF (48 mL) and cooled to -78 ºC under argon atmosphere. To this solution was added lithium bis(trimethylsilyl)amide (LiHMDS) (1.0 M solution in THF, 19.8 mL, 19.8 mmol, 1.2 equiv.) dropwise. The reaction mixture was warmed to r.t. and stirred for 4 h. Acetone cyanohydrin (3.02 mL, 33 mmol, 2 equiv.) was then added. The reaction mixture was stirred at r.t. for 12 h and then quenched with $NaHCO_3$ aq. and extracted with AcOEt, washed with brine, dried over Na_2SO_4, filtered and concentrated *in vacuo* to obtain the crude of aminonitrile as a yellow oil (4.58 g, 17.1 mmol, quant).[4]

This product is easily decomposed, thus was engaged in next reaction without storing.

^{1}H NMR (400 MHz, Chloroform-*d*) δ 7.50 – 7.43 (m, 2H), 7.41 – 7.35 (m, 2H), 7.34 – 7.27 (m, 1H), 7.13 – 7.03 (m, 2H), 6.90 (d, *J* = 8.0 Hz, 1H), 5.17 (s, 2H), 4.81 (s, 1H), 3.89 (s, 3H).

^{13}C{^{1}H} NMR (126 MHz, Chloroform-*d*) δ 150.4, 148.7, 136.8, 128.7, 128.2, 127.6, 121.1, 119.7, 112.6, 111.9, 71.3, 56.2, 47.1.

The crude of aminonitrile (924 mg, 3.44 mmol, 1 equiv.) was dissolved in methanolic 3N HCl (23 mL, 68.8 mmol, 20 equiv.) and stirred at r.t. overnight. The solvent was evaporated *in vacuo*, and the residue was treated with $NaHCO_3$ aq., extracted with AcOEt, washed with brine, dried over Na_2SO_4, filtered and concentrated *in vacuo*. The residue was purified by silica gel column chromatography using CH_2Cl_2/AcOEt/Et_3N to provide the title compound **6** (446 mg, 1.48 mmol, 43% over 2 steps) as a pale yellow oil.

^{1}H NMR (500 MHz, Chloroform-*d*) δ 7.47 – 7.41 (m, 2H), 7.39 – 7.34 (m, 2H), 7.32 – 7.27 (m, 1H), 6.93 (dd, *J* = 6.2, 2.2 Hz, 2H), 6.86 (d, *J* = 8.8 Hz, 1H), 5.15 (s, 2H), 4.51 (s, 1H), 3.87 (s, 3H), 3.64 (s, 3H).

^{13}C {^{1}H} NMR (126 MHz, Chloroform-*d*) δ 174.7, 149.7, 148.5, 137.1, 132.9, 128.7, 128.0, 127.6, 119.8, 112.8, 111.99, 71.2, 58.5, 56.2, 52.5.

HRMS (ESI) m/z: calculated for $C_{17}H_{19}NO_4Na$ $[M+Na]^+$: 324.1206; found: 324.1203.

IR (neat): ν (cm^{-1}) 3316, 3198, 2911, 2362, 1735, 1591, 1514, 1442, 1383, 1336, 1256, 1139, 1082, 1015, 847, 803, 736, 696.

Rf 0.18 (CH_2Cl_2:AcOEt = 1:5 with 1% v/v Et_3N)

1-(3-(Benzyloxy)-4-methoxyphenyl)-2-((*tert*-butyldimethylsilyl)oxy)ethan-1-amine (15):

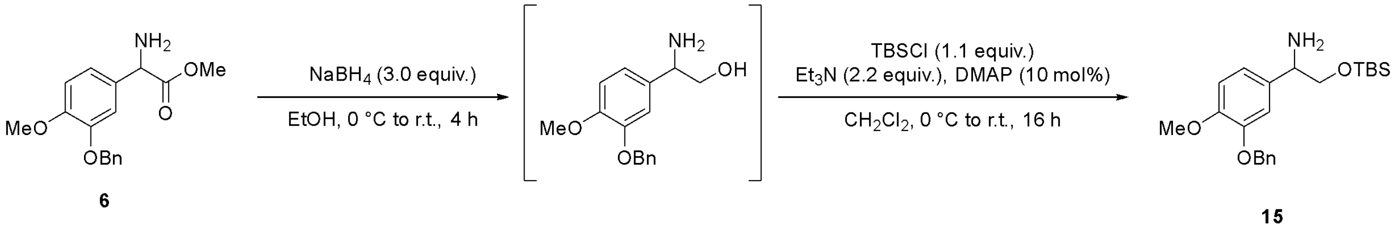

2-Amino ester **6** (600 mg, 1.99 mmol, 1.0 equiv.) was dissolved in EtOH (7 mL) in a round bottom flask and cooled to 0 °C using an ice bath. $NaBH_4$ (226 mg, 5.97 mmol, 3.0 equiv.) was added in small portions. The ice bath was removed after 15 min and the reaction was stirred at room temperature upon full conversion indicated by TLC (approximately 4 h). The solvent was then removed under reduced pressure followed by addition of water and AcOEt. The phases were separated and the aqueous phase was extracted with AcOEt. The combined organic phases were washed with brine and dried over Na_2SO_4, filtered and concentrated *in vacuo* to give the amino alcohol (536 mg, 1.96 mmol, 99%) as a pale brown oil.

^{1}H NMR (400 MHz, Chloroform-*d*) δ 7.46 – 7.41 (m, 2H), 7.40 – 7.33 (m, 2H), 7.33 – 7.27 (m, 1H), 6.90 – 6.85 (m, 3H), 5.16 (s, 2H), 3.94 (dd, *J* = 8.0, 4.5 Hz, 1H), 3.88 (s, 3H), 3.64 (dd, *J* = 10.7, 4.6 Hz, 1H), 3.46 (dd, *J* = 10.7, 8.0 Hz, 1H).

^{13}C{^{1}H} NMR (101 MHz, Chloroform-*d*) δ 149.3, 148.3, 137.2, 128.7, 128.0, 127.6, 119.3, 112.9, 112.0, 77.4, 71.3, 68.2, 57.0, 56.2.

HRMS (ESI) m/z: calculated for $C_{16}H_{19}NO_3Na$ $[M+Na]^+$: 296.1257; found: 296.1256.

IR (neat): ν (cm^{-1}) 3316, 3197, 2886, 2362, 1591, 1516, 1443, 1384, 1336, 1244, 1140, 1081, 1013, 875, 803, 737, 695.

Rf 0.25 (CH_2Cl_2/MeOH = 80:20)

The obtained amino alcohol (586 mg, 2.14 mmol, 1.0 equiv.) was dissolved in dry CH_2Cl_2 (6 mL) in a round bottom flask and cooled to 0 °C using an ice bath. Subsequently Et_3N (654 µL, 4.71 mmol, 2.2 equiv.), TBSCl (355 mg, 2.35 mmol, 1.1 equiv.) and DMAP (26 mg, 0.214 mmol, 10 mol%) were added. The ice bath was removed after 15 min and the reaction was stirred at room temperature for 16 h. The reaction was then quenched by addition of water. The phases were separated and the aqueous phase was extracted with CH_2Cl_2 The combined organic phases were washed with brine and dried over Na_2SO_4, filtered and concentrated *in vacuo.* The crude was purified using flash column chromatography on silica gel (cyclohexane/AcOEt 90:10 to 70:30 with 1% v/v Et_3N) to yield 1-(3-(Benzyloxy)-4-methoxyphenyl)-2-((*tert*-butyldimethylsilyl)oxy)ethan-1-amine (**15**) (764 mg, 2.14 mmol, 92%) as a light-yellow oil.

^{1}**H NMR** (400 MHz, Chloroform-*d*) δ 7.48 – 7.41 (m, 2H), 7.40 – 7.31 (m, 2H), 7.32 – 7.27 (m, 1H), 6.98 (d, *J* = 2.0 Hz, 1H), 6.91 (dd, *J* = 8.5, 2.2 Hz, 1H), 6.85 (d, *J* = 8.3 Hz, 1H), 5.15 (s, 2H), 3.97 (dd, *J* = 8.7, 3.9 Hz, 1H), 3.87 (s, 3H), 3.61 (dd, *J* = 9.8, 3.9 Hz, 1H), 3.41 (dd, *J* = 9.8, 8.7 Hz, 1H), 0.89 (s, 9H), 0.02 (s, 6H).

^{13}**C{^{1}H} NMR** (101 MHz, Chloroform-*d*) δ 149.1, 148.2, 137.3, 135.4, 128.6, 127.9, 127.6, 119.7, 113.2, 111.8, 71.2, 69.8, 57.3, 56.2, 26.1, 18.4, -5.2, -5.3.

HRMS (ESI) m/z: calculated for $C_{22}H_{33}NO_3SiH$ $[M+H]^+$: 388.2302; found: 388.2300.

IR (neat): ν/cm^{-1} 2929, 2856, 2362, 1691, 1590, 1513, 1462, 1382, 1256, 1083, 1026, 837, 776, 666, 640.

Rf 0.25 (Cyclohexane/AcOEt = 70:30 with 1% v/v Et_3N)

5,6-Dibromo-1,3-benzodioxole (5):

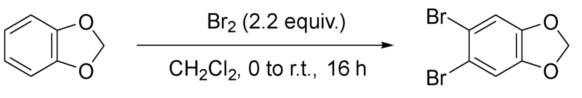

5

The title compound was prepared according to a procedure reported in the literature.[5] To a solution of 1,2-methylenedioxybenzene (1.55 mL, 15.0 mmol, 1.0 eq.) in CH_2Cl_2 (30 mL) was added Br_2 (1.69 mL, 33.0 mmol, 2.2 eq.) in a round bottom flask at 0 °C using an ice bath. The ice bath was removed after 15 min and the mixture was stirred for 16 h. The reaction was quenched with $Na_2S_2O_3$ aq. and neutralized with 2M NaOH. The phases were separated and aqueous phase was extracted with CH_2Cl_2. The combined organic phases were washed with brine and dried over Na_2SO_4, filtered and concentrated *in vacuo*. The resulting crude was purified using flash column chromatography on silica gel (cyclohexane/AcOEt 100:0 to 90:10) to give 5,6-dibromo-1,3-benzodioxole (**5**) (3.55 g, 12.8 mmol, 85%) as white crystals. The analytical data was in full agreement with the literature data.[5]

^{1}H NMR (400 MHz**,** Chloroform-*d*) δ 7.07 (s, 2H), 6.00 (s, 2H).

^{13}C{^{1}H} NMR (101 MHz, Chloroform-*d*) δ 148.1, 115.5, 113.4, 102.5.

Rf 0.32 (Cyclohexane)

***N*-(1-(3-(Benzyloxy)-4-methoxyphenyl)-2-((*tert*-butyldimethylsilyl)oxy)ethyl)-6-bromobenzo[*d*][1,3]dioxol-5-amine (16):**

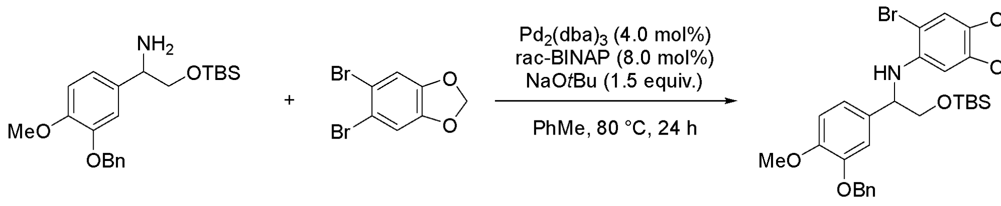

15 **5** **16**

A round bottom flask was charged with amine **15** (617 mg, 1.59 mmol, 1.0 equiv.) and aryl dibromide **5** (468 mg, 1.67 mmol, 1.05 equiv.). The flask was then transferred to an Ar-filled glovebox and $Pd_2(dba)_3$ (58 mg, 0.0636 mmol, 4.0 mol%), *rac*-BINAP (79 mg, 0.127 mmol, 8.0 mol%) and NaO*t*Bu (229 mg, 2.39 mmol, 1.5 equiv.) were added. The flask was sealed with a rubber septum and outside the glovebox, dry and degassed toluene (16 mL) was added. This suspension was stirred for 5 min at room temperature and then directly immersed in an 80 °C pre-heated oil bath. The reaction was cooled to room temperature after 16 h and subsequently filtered through a short plug of *Celite* and washed with copious amounts of AcOEt. The solvent was evaporated under reduced pressure and the resulting crude was purified using flash column chromatography on silica gel (cyclohexane/AcOEt 98:2 to 84:16) to give *N*-(1-(3-(Benzyloxy)-4-methoxyphenyl)-2-((*tert*-butyldimethylsilyl)oxy)ethyl)-6-bromobenzo[*d*][1,3]dioxol-5-amine (**16**) (820 mg, 1.40 mmol, 88%) as an orange oil.

^{1}H NMR (400 MHz, Chloroform-*d*) δ 7.43 – 7.36 (m, 2H), 7.33 – 7.27 (m, 2H), 7.25 – 7.20 (m, 1H), 6.91 (s, 1H), 6.90 – 6.87 (m, 1H), 6.86 – 6.82 (m, 1H), 5.78 (d, *J* = 6.4 Hz, 1H), 5.78 (d, *J* = 6.4 Hz, 1H), 5.18 – 5.05 (m, 3H), 4.18 (dt, *J* = 7.7, 3.8 Hz, 1H), 3.81 (dd, *J* = 10.2, 4.0 Hz, 1H), 3.57 (dd, *J* = 10.1, 7.7 Hz, 1H), 0.90 (s, 9H), 0.03 (s, 3H), 0.00 (s, 3H).

^{13}C{^{1}H} NMR (101 MHz, Chloroform-*d*) δ 149.2, 148.3, 147.7, 140.4, 139.5, 137.1, 132.9, 128.6, 127.9, 127.6, 119.7, 113.0, 112.3, 112.0, 101.1, 99.7, 96.2, 71.1, 67.9, 60.6, 56.2, 26.0, 18.3, -5.3, -5.4.

HRMS (ESI) m/z: calculated for $C_{29}H_{36}BrNO_5SiNa$ $[M+Na]^+$: 608.1438; found: 608.1428.

IR (neat): ν/cm^{-1} 3375, 2929, 2857, 2361, 1737, 1631, 1606, 1503, 1468, 1425, 1378, 1257, 1230, 1193, 1096, 1037, 935, 838, 777, 735, 697, 634.

Rf 0.25 (Cyclohexane/AcOEt = 95:5)

N-(2-(Benzyloxy)-1-(3-(benzyloxy)-4-methoxyphenyl)ethyl)-6-bromobenzo[*d*][1,3]dioxol-5-amine:

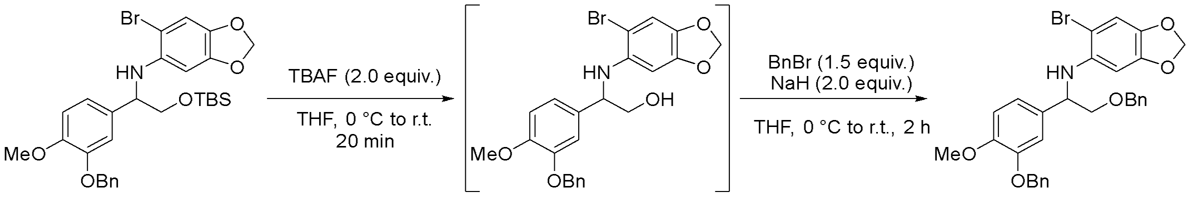

16

TBS-protected amino alcohol **16** (744 mg, 1.27 mmol, 1.0 equiv.) was dissolved in dry THF (4 mL) under an inert atmosphere in a round bottom flask and subsequently cooled to 0 °C using an ice bath. 1M TBAF in THF (2.5 mL, 2.54 mmol, 2.0 equiv.) was then added dropwise. After complete conversion indicated by TLC (approximately after 20 min), $NaHCO_3$ aq. and AcOEt were added. The phases were separated and the aqueous phase was extracted with AcOEt. The combined organic phases were washed with brine and dried over Na_2SO_4, filtered and concentrated *in vacuo*. The crude alcohol was directly transferred to a two-necked flask. Dry THF (6 mL) was added under an inert atmosphere and the resulting solution was cooled to 0 °C using an ice bath. Then a 60% w/w NaH suspension in mineral oil (102 mg, 2.54 mmol, 2.0 equiv.) was added in small portions under an Ar counterflow. After 30 min, BnBr (228 μL, 1.91 mmol, 1.5 equiv.) was added and the reaction mixture was warmed up to room temperature. After complete conversion indicated by TLC (approximately after 2 h), water was added at 0 °C. AcOEt was then added and the phases were separated and the aqueous phase was extracted with AcOEt. The combined organic phases were washed with brine, dried over Na_2SO_4, filtered and concentrated *in vacuo*. The crude was purified by flash column chromatography on silica gel (cyclohexane/AcOEt 98:2 to 82:18) to yield N-(2-(Benzyloxy)-1-(3-(benzyloxy)-4-methoxyphenyl)ethyl)-6-bromobenzo[*d*][1,3]dioxol-5-amine (698 mg, 1.27 mmol, 98% over two steps) as a yellow oil.

^{1}H NMR (400 MHz Chloroform-*d*) δ 7.35 – 7.14 (m, 10H), 6.85 (s, 1H), 6.84 – 6.80 (m, 2H), 6.77 (d, J = 8.7 Hz, 1H), 5.88 (s, 1H), 5.72 (d, J = 7.0 Hz, 1H), 5.72 (d, J = 7.0 Hz, 1H), 5.08 – 4.97 (m, 2H), 4.92 (d, J = 3.6 Hz, 1H), 4.53 – 4.41 (m, 2H), 4.27 (dt, J = 7.8, 3.8 Hz, 1H), 3.79 (s, 3H), 3.59 (dd, J = 9.8, 4.0 Hz, 1H), 3.45 (dd, J = 9.9, 8.4 Hz, 1H).

^{13}C{^{1}H} NMR (126 MHz, Chloroform-*d*) δ 149.3, 148.4, 147.8, 140.2, 139.6, 137.9, 137.1, 132.6, 128.6, 128.6, 127.9, 127.9, 127.9, 127.6, 119.6, 112.8, 112.3, 112.1, 101.1, 99.7, 96.1, 74.5, 73.1, 71.2, 58.7, 56.2.

HRMS (ESI) m/z: calculated for $C_{30}H_{28}BrNO_5H$ [M+H]$^+$: 562.1224; found: 562.1215.

IR (ν/cm^{-1}): 3377, 2897, 2861, 2362, 1735, 1631, 1606, 1503, 1425, 1354, 1322, 1258, 1229, 1192, 1135, 1100, 1034, 933, 841, 736, 698, 635.

Rf 0.5 (Cyclohexane/AcOEt= 83:17)

Methyl (2-(benzyloxy)-1-(3-(benzyloxy)-4-methoxyphenyl)ethyl)(6-bromobenzo[*d*][1,3]dioxol-5-yl)carbamate (4):

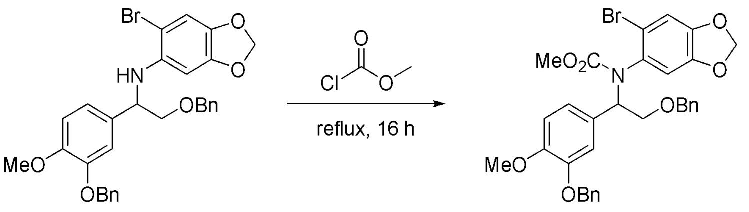

4

A two-neck flask equipped with a reflux condenser was charged with benzyl-protected alcohol **I** (1.82 g, 3.23 mmol, 1.0 equiv.) under an inert atmosphere. Methyl chloroformate (11.7 mL, 152 mmol, 47 equiv.) was added and the reaction mixture was heated up to reflux for 16 h. The reaction was then allowed to cool to room temperature and the volatiles were evaporated under reduced pressure. The crude oil was then purified by column chromatography on silica gel (cyclohexane/AcOEt 90:10 to 80:20) to yield the methyl carbamate **J** (1.35 g, 2.18 mmol, 67%) as a pale brown solid.

***Note**: This compound was obtained as a mixture of rotamers. These data represent empirically observed chemical shifts.*

^{1}H NMR (400 MHz, Chloroform-*d*) δ 7.44 – 7.27 (m, 9H), 7.25 – 7.19 (m, 1H), 7.02 – 6.91 (m, 1H), 6.89 – 6.77 (m, 2H), 6.75 – 6.56 (m, 2H), 6.04 – 5.89 (m, 2H), 5.58 (brs, 0.6H, *major*), 5.40 (t, *J* = 7.0 Hz, 0.4H, *minor*), 5.11 – 4.86 (m, 2H), 4.70 – 4.33 (m, 2H), 3.96 (d, *J* = 9.7 Hz, 1H), 3.88 (s, 1H), 3.84 (s, 2H), 3.77 – 3.56 (m, 4H).

^{13}C{^{1}H} NMR (126 MHz, Chloroform-*d*) δ 149.7, 149.5, 147.9, 147.7, 147.6, 147.4, 147.3, 147.0, 138.2, 137.3, 137.3, 130.8, 128.6, 128.6, 128.5, 128.4, 127.9, 127.9, 127.8, 127.8, 127.7, 127.5, 127.4, 122.2, 121.9, 118.0, 116.6, 115.6, 115.3, 112.5, 112.3, 111.4, 111.2, 102.3, 102.3, 73.2, 72.9, 71.2, 56.1, 56.0, 53.3, 53.3.

HRMS (ESI) m/z: calculated for $C_{32}H_{30}BrNO_7Na$ $[M+Na]^+$: 642.1098; found: 642.1095.

IR (ν/cm^{-1}): 2952, 2907, 1693, 1590, 1515, 1478, 1444, 1368, 1341, 1317, 1285, 1251, 1198, 1141, 1079, 1033, 1008, 932, 847, 750, 701, 649, 609.

Rf 0.28 (Cyclohexane/AcOEt = 83:17)

Melting point: 141 – 143 °C.

PKR for Enantioselective Core Synthesis of Cryptowolinol

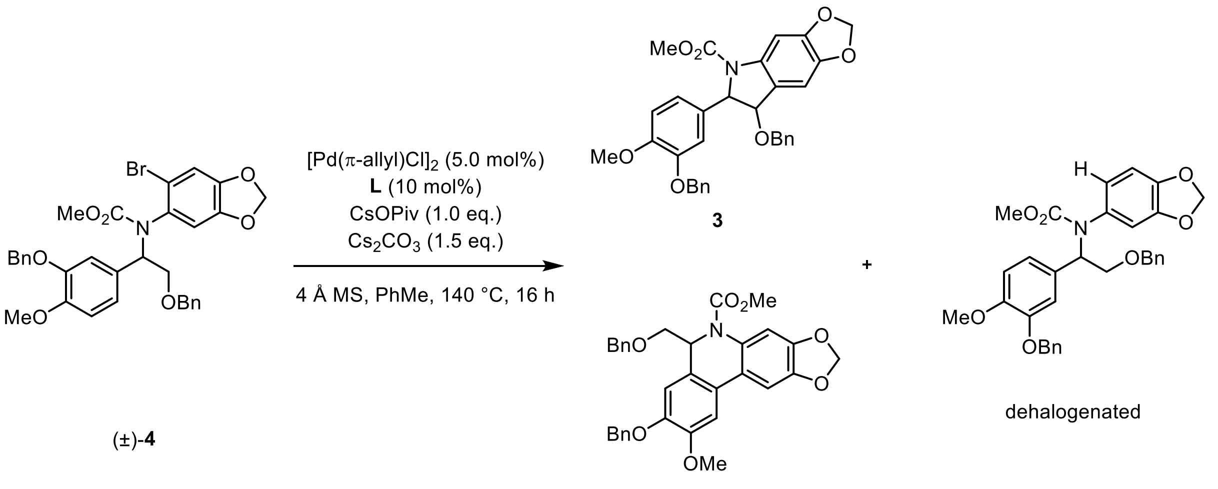

(±)-**4** **3** **17** dehalogenated

A 10 mL screw-cap catalyst tube was charged with methyl carbamate **4** (62.0 mg, 0.100 mmol, 1.0 eq.). The tube was then transferred to an Ar-filled glovebox were [Pd(π-allyl)Cl]$_2$ (1.83 mg, 0.005 mmol, 5.0 mol%), NHC precursor (0.01 mmol, 10 mol%), CsOPiv (23.4 mg, 0.1 mmol, 1.0 equiv.), Cs_2CO_3 (48.9 mg, 0.15 mmol, 1.5 equiv.) and 4Å MS (25.0 mg) were added. The vial was capped with a rubber septum and outside the glovebox, dry and degassed toluene (1.0 mL) was added. The rubber septum was then replaced by a plastic cap under an Ar-counterflow and the reaction was stirred for 5 min at room temperature before the vial was directly put in a 140 °C pre-heated heating block. After 16 h, the reaction was allowed to cool to room temperature, filtered over a short plug of *Celite* and washed with copious AcOEt. The solvent was then evaporated under reduced pressure and the crude was analysed by ^{1}H NMR using trichloroethylene as standard. **3**, **17** and dehalogenated compound were then separated by preparative HPLC (cyclohexane/AcOEt 75:25). The enantiomeric ratio of **3** and **17** was determined by HPLC using a chiral stationary phase.

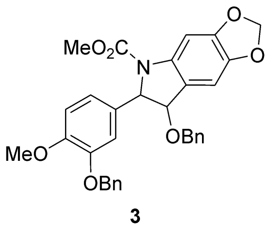

3

^{1}H NMR (400 MHz, Dichloromethane-*d2*) δ 7.70 – 7.50 (brs, 1H), 7.40 – 7.25 (m, 10H), 6.80 (d, *J* = 8.3 Hz, 1H), 6.75 (s, 1H), 6.70 – 6.64 (m, 1H), 6.62 (brs, 1H), 6.00 (d, *J* = 8.3 Hz, 1H), 6.00 (d, *J* = 8.3 Hz, 1H), 5.26 (brs, 1H), 4.97 (s, 2H), 4.64 (d, *J* = 11.7 Hz, 1H), 4.56 (d, *J* = 11.7 Hz, 1H), 4.49 (s, 1H), 3.80 (s, 3H), 3.61 (brs, 2H).

***Note**: The broad downfield signal from 7.70 to 7.50 ppm corresponds to an aryl signal although it only integrates to 0.4. However, the same phenomenon was observed with indoline products in Kündig's PKR[6]*

^{13}C{^{1}H} NMR (126 MHz, Dichloromethane-*d2*) δ 153.4, 149.9, 149.6, 148.7, 144.0, 138.5, 137.4, 133.0, 128.9, 128.8, 128.3, 128.2, 128.1, 128.1, 118.4, 112.4, 111.8, 106.7, 102.9, 102.2, 98.2, 85.2, 73.2, 71.3, 70.3, 69.6, 56.3, 52.9.

HRMS (ESI) m/z: calculated for $C_{32}H_{29}NO_7Na$ $[M+Na]^+$: 562.1836; found: 562.1832.

IR (v/cm^{-1}): 2952, 2894, 2361, 1700, 1608, 1484, 1437, 1395, 1348, 1314, 1248, 1207, 1141, 1101, 1035, 935, 856, 734, 698, 646, 613.

Rf 0.55 (Cyclohexane/AcOEt 60:40)

$[\alpha]_D^{20}$: +4.6 (c = 0.48, $CHCl_3$).

HPLC separation: Chiralcel ®IA, 85:15 (*n*-heptane/*i*PrOH), 1.0 mLmin^{-1}, 216 nm, t_R (major) = 17.7 min, $t_{R,}$(minor) = 20.7 min, 0.5:99.5 e.r. ((*R*,*R*)-**L^2** was employed.)

***Note**: **3** was dissolved only in AcOEt prior to analysis, otherwise no peaks appeared.*

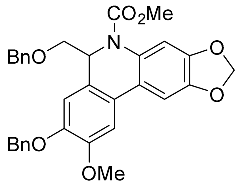

17

^{1}H NMR (400 MHz, Dichloromethane-*d2*) δ 7.48 – 7.18 (m, 11H), 7.12 (s, 2H), 6.84 (s, 1H), 6.00 (d, *J* = 9.3 Hz, 1H), 6.00 (d, *J* = 9.3 Hz, 1H), 5.62 (brs, 1H), 5.18 – 4.95 (m, 2H), 4.47 (d, *J* = 12.0 Hz, 1H), 4.38 (d, *J* = 12.0 Hz, 1H), 3.90 (s, 3H), 3.74 (s, 3H), 3.32 (d, *J* = 7.0 Hz, 2H).

^{13}C{^{1}H} NMR (126 MHz, Dichloromethane-*d2*) δ 155.1, 150.3, 148.2, 147.0, 145.7, 138.7, 137.3, 128.9, 128.6, 128.5, 128.2, 127.9, 125.0, 122.2, 112.6, 107.2, 102.9, 102.0, 73.2, 71.5, 70.1, 56.6, 55.9.

HRMS (ESI) m/z: calculated for $C_{32}H_{29}NO_7Na$ $[M+Na]^+$: 562.1836; found: 562.1832.

IR (v/cm^{-1}): 3031, 2895, 2861, 2361, 1700, 1607, 1485, 1436, 1394, 1349, 1314, 1250, 1206, 1139, 1100, 1034, 934, 853, 734, 697, 615.

Rf: 0.53 (Cyclohexane/AcOEt 60:40)

HPLC separation: Chiralcel ® OD-H, 85:15 (*n*-heptane/*i*PrOH), 0.5 mLmin^{-1}, 300 nm, t_R (major) = 33.6 min, $t_{R,}$(minor) = 37.0 min, 34:66 e.r. ((*R*,*R*)-L^3 was employed.)

***Note**: **17** was dissolved only in AcOEt prior to HPLC analysis, otherwise no peaks appeared.*

Scaled up PKR for Enantioselective Core Synthesis of Cryptowolinol

Methyl (6*R*,7*S*)-7-(benzyloxy)-6-(3-(benzyloxy)-4-methoxyphenyl)-6,7-dihydro-5*H*-[1,3]dioxolo[4,5-*f*]indole-5-carboxylate (3):

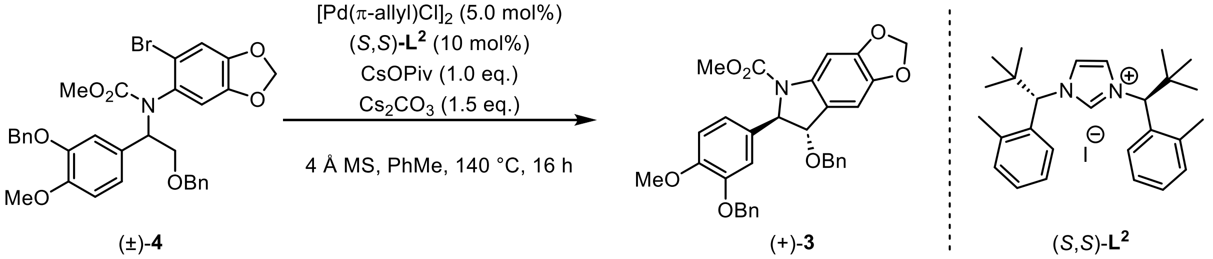

A 25 mL screw-cap glass pressure tube was charged with methyl carbamate **4** (310 mg, 0.500 mmol, 1.0 equiv.). The tube was then transferred to an Ar-filled glovebox where [Pd(π-allyl)Cl]$_2$ (9.15 mg, 25.0 µmol, 5.0 mol%), (*S*,*S*)-**L**^{2} (25.8 mg, 50.0 µmol, 10 mol%), CsOPiv (117 mg, 0.500 mmol, 1.0 equiv.), Cs_2CO_3 (244 mg, 0.750 mmol, 1.5 equiv.) and 4 Å MS (60.0 mg) were added. The vial was capped with a rubber septum and outside the glovebox dry and degassed toluene (5.0 mL) was added. The rubber septum was then replaced by a plastic screw cap under an Ar-counterflow and the reaction was stirred for 5 min at room temperature before the reaction tube was directly put in a 140 °C pre-heated oil bath. After 16 h, the reaction was allowed to cool to room temperature, filtered over a short plug of *Celite* and washed with copious AcOEt. The solvent was removed under reduced pressure. The crude was then divided into two batches and each was purified using preparative HPLC (cyclohexane/AcOEt 75:25). The batches were combined to yield indoline **3** (107 mg, 0.199 mmol, 40%) as a viscous yellow oil. The e.r was determined by HPLC using a chiral stationary phase.

***Note**: The C(sp^2)–H product **17** was not isolated and analysed since a separation of **3** and dehalogenated compound was not possible when the preparative HPLC column was charged with a higher crude loading.*

HPLC separation: Chiralcel ® IA, 85:15 (*n*-heptane/*i*PrOH) 1.0 mLmin^{-1}, 216 nm, t_R (minor) = 19.3 min, t_R (major) = 21.9 min, 98:2 e.r.

Crystallographic data

Experimental: Single colourless plate-shaped crystals of **12j** were obtained by recrystallization from EtOAc/pentane (98.5:1.5 e.r.). A suitable crystal with dimensions 0.20 × 0.16 × 0.07 mm^3 was selected and mounted on a MITIGEN holder in per-fluoro-ether oil on a STOE STADIVARI Cu diffractometer. The crystal was kept at a steady T = 150 K during data collection. The structure was solved with the ShelXT 2018/2 (Sheldrick, 2018) solution program using dual methods and by using Olex2 1.5 (Dolomanov et al., 2009) as the graphical interface. The model was refined with ShelXL 2018/3 (Sheldrick, 2015) using full matrix least squares minimisation on F^2.

Crystal Data. $C_{18}H_{19}NO_4$, M_r = 313.34, monoclinic, $P2_1$ (No. 4), a = 9.3211(3) Å, b = 8.8433(2) Å, c = 9.7618(3) Å, b = 100.965(2)°, a = g = 90°, V = 789.97(4) Å^3, T = 150 K, Z = 2, Z' = 1, m(Cu Ka) = 0.764, 16485 reflections measured, 2897 unique (R_{int} = 0.0141) which were used in all calculations. The final wR_2 was 0.0675 (all data) and R_1 was 0.0255 (I≥2 s(I)).

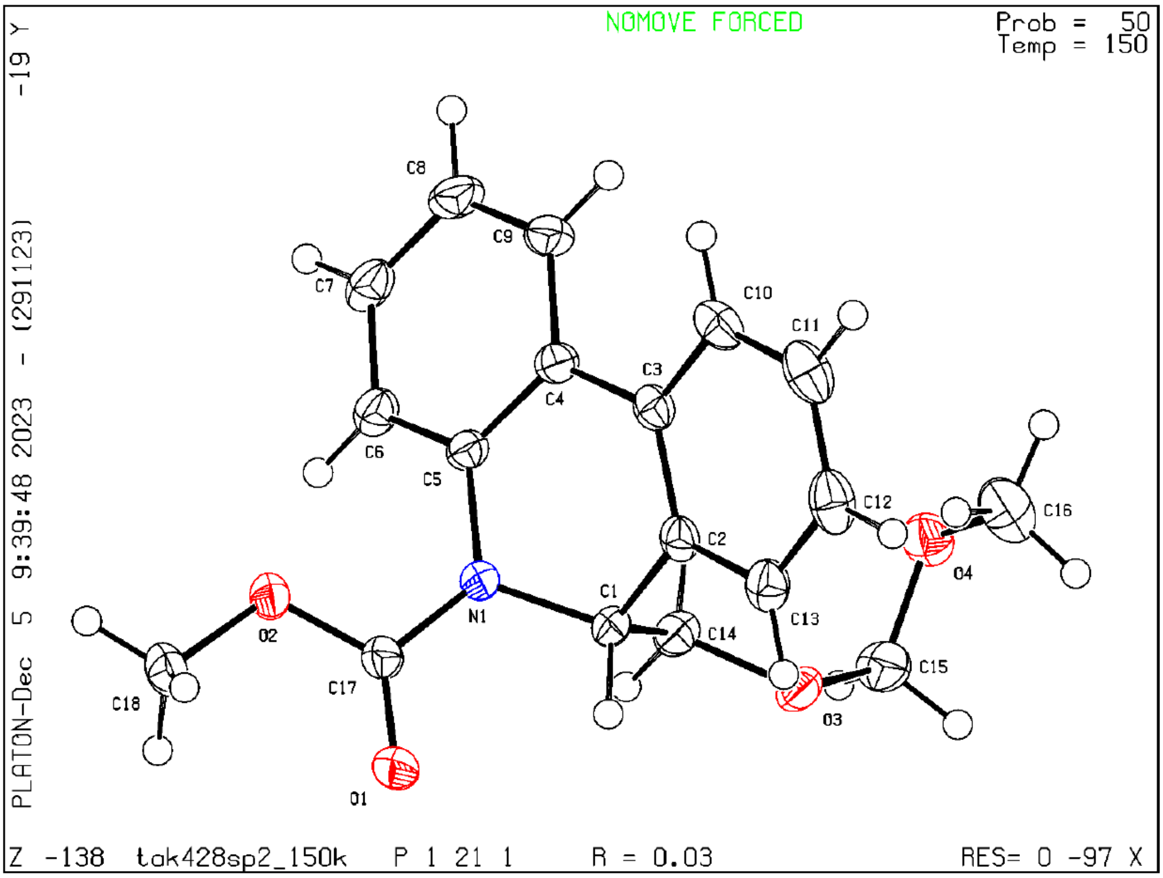

Formula	$C_{18}H_{19}NO_4$
$D_{calc.}$/ g cm^{-3}	1.317
μ/mm^{-1}	0.764
Formula Weight	313.34
Colour	colourless
Shape	plate-shaped
Size/mm^3	0.20×0.16×0.07
T/K	150
Crystal System	monoclinic
Flack Parameter	0.02(7)
Hooft Parameter	0.02(2)
Space Group	$P2_1$
a/Å	9.3211(3)
b/Å	8.8433(2)
c/Å	9.7618(3)
α/°	90
β/°	100.965(2)
γ/°	90
V/Å^3	789.97(4)
Z	2
Z'	1
Wavelength/Å	1.54186
Radiation type	Cu K_α
Θ_{min}/°	4.614
Θ_{max}/°	69.912
Measured Refl's.	16485
Indep't Refl's	2897
Refl's I≥2 σ(I)	2845
R_{int}	0.0141
Parameters	210
Restraints	1
Largest Peak	0.123
Deepest Hole	-0.181
GooF	1.061
wR_2 (all data)	0.0675
wR_2	0.0672
R_1 (all data)	0.0259
R_1	0.0255

```
Bond precision:    C-C = 0.0022 A           Wavelength=1.54186

Cell:              a=9.3211(3)     b=8.8433(2)          c=9.7618(3)
                   alpha=90        beta=100.965(2)      gamma=90
Temperature:       150 K

                  Calculated                    Reported
Volume            789.97(4)                     789.97(4)
Space group       P 21                          P 1 21 1
Hall group        P 2yb                         P 2yb
Moiety formula    C18 H19 N O4                  C18 H19 N O4
Sum formula       C18 H19 N O4                  C18 H19 N O4
Mr                313.34                        313.34
Dx,g cm-3         1.317                         1.317
Z                 2                             2
Mu (mm-1)         0.764                         0.764
F000              332.0                         332.0
F000'             333.06
h,k,lmax                                        11,10,11
Nref                                            2897
Tmin,Tmax         0.866,0.948                   0.400,0.554
Tmin'             0.858

Correction method= # Reported T Limits: Tmin=0.400 Tmax=0.554
AbsCorr = MULTI-SCAN

Data completeness=                  Theta(max)= 69.912

                                                     wR2(reflections)=
R(reflections)= 0.0255( 2845)                        0.0675( 2897)

S = 1.061                   Npar= 210
```

The following ALERTS were generated. Each ALERT has the format
test-name_ALERT_alert-type_alert-level.
Click on the hyperlinks for more details of the test.

Alert level C

```
PLAT911_ALERT_3_C Missing FCF Refl Between Thmin & STh/L=    0.600          3 Report
                   1   0   0,    5   0   1,    5   0   3,
PLAT918_ALERT_3_C Reflection(s) with I(obs) much Smaller I(calc) .          5 Check
PLAT939_ALERT_3_C Large Value of Not (SHELXL) Weight Optimized S .      10.77 Check
```

Alert level G

```
PLAT912_ALERT_4_G Missing # of FCF Reflections Above STh/L=  0.600         7 Note
PLAT933_ALERT_2_G Number of HKL-OMIT Records in Embedded .res File         5 Note
                   0  -1   1,    1   0   0,    5   0   1,    5   0   3,   11   1   0,
PLAT978_ALERT_2_G Number C-C Bonds with Positive Residual Density.         2 Info
```

---

0 **ALERT level A** = Most likely a serious problem - resolve or explain
0 **ALERT level B** = A potentially serious problem, consider carefully
3 **ALERT level C** = Check. Ensure it is not caused by an omission or oversight
3 **ALERT level G** = General information/check it is not something unexpected

0 ALERT type 1 CIF construction/syntax error, inconsistent or missing data
2 ALERT type 2 Indicator that the structure model may be wrong or deficient
3 ALERT type 3 Indicator that the structure quality may be low
1 ALERT type 4 Improvement, methodology, query or suggestion
0 ALERT type 5 Informative message, check

References

[1] Remote Construction of N-Heterocycles via 1,4-Palladium Shift-Mediated Double C−H Activation, T. Miyakoshi, N. E. Niggli, O. Baudoin, *Angew. Chem. Int. Ed.* **2022**, *61*, DOI 10.1002/anie.202116101.

[2] Palladium(0)-Catalyzed Asymmetric C(sp^3)–H Arylation Using a Chiral Binol-Derived Phosphate and an Achiral Ligand, L. Yang, R. Melot, M. Neuburger, O. Baudoin, *Chem. Sci.* **2017**, *8*, 1344–1349.

[3] Unusual and Unexpected Reactivity of t-Butyl Dicarbonate (Boc_2O) with Alcohols in the Presence of Magnesium Perchlorate. A New and General Route to t-Butyl Ethers, G. Bartoli, M. Bosco, M. Locatelli, E. Marcantoni, P. Melchiorre, L. Sambri, *Org. Lett.* **2005**, *7*, 427–430.

[4] Development of a New α-Aminonitrile Synthesis, G. H. Chu, M. Gu, B. Gerard, R. E. Dolle, *Synth. Commun.* **2004**, *34*, 4583–4590.

[5] Enantioselective Total Synthesis of (+)-Homochelidonine by a Pd^{II} - Catalyzed Asymmetric Ring-Opening Reaction of a meso-Azabicyclic Alkene with an Aryl Boronic Acid, H. A. McManus, M. J. Fleming, M. Lautens, *Angew. Chem. Int. Ed.* **2007**, *46*, 433–436.

[6] Asymmetric C(sp^3)-H/C(Ar) coupling reactions. Highly enantio-enriched indolines via regiodivergent reaction of a racemic mixture, D. Katayev, M. Nakanishi, T. Bürgi, E. P. Kündig, *Chem. Sci.* **2012**, *3*, 1422–1425.

NMR Spectrum Data of Model Substrates

^{1}H NMR (400 MHz, Chloroform-*d*)

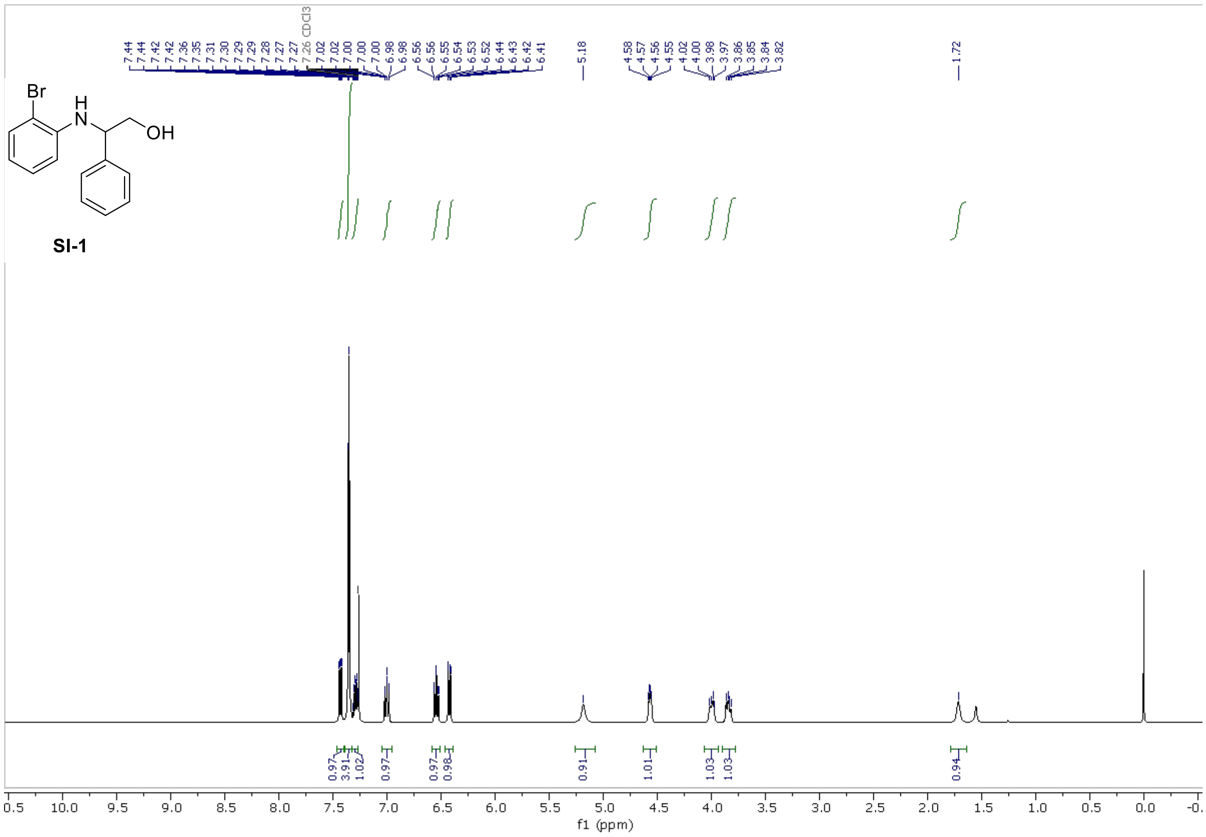

^{13}C{^{1}H} NMR (126 MHz, Chloroform-*d*)

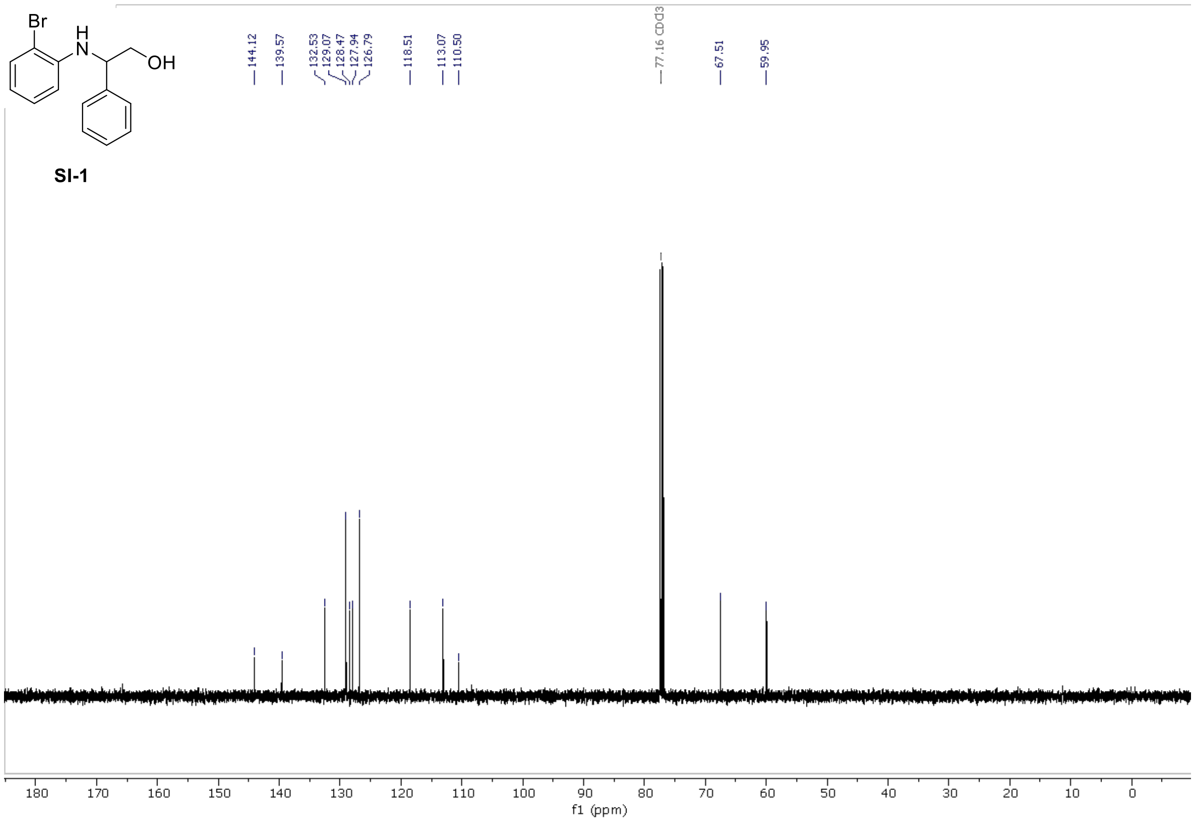

^{1}H NMR (400 MHz, Chloroform-d)

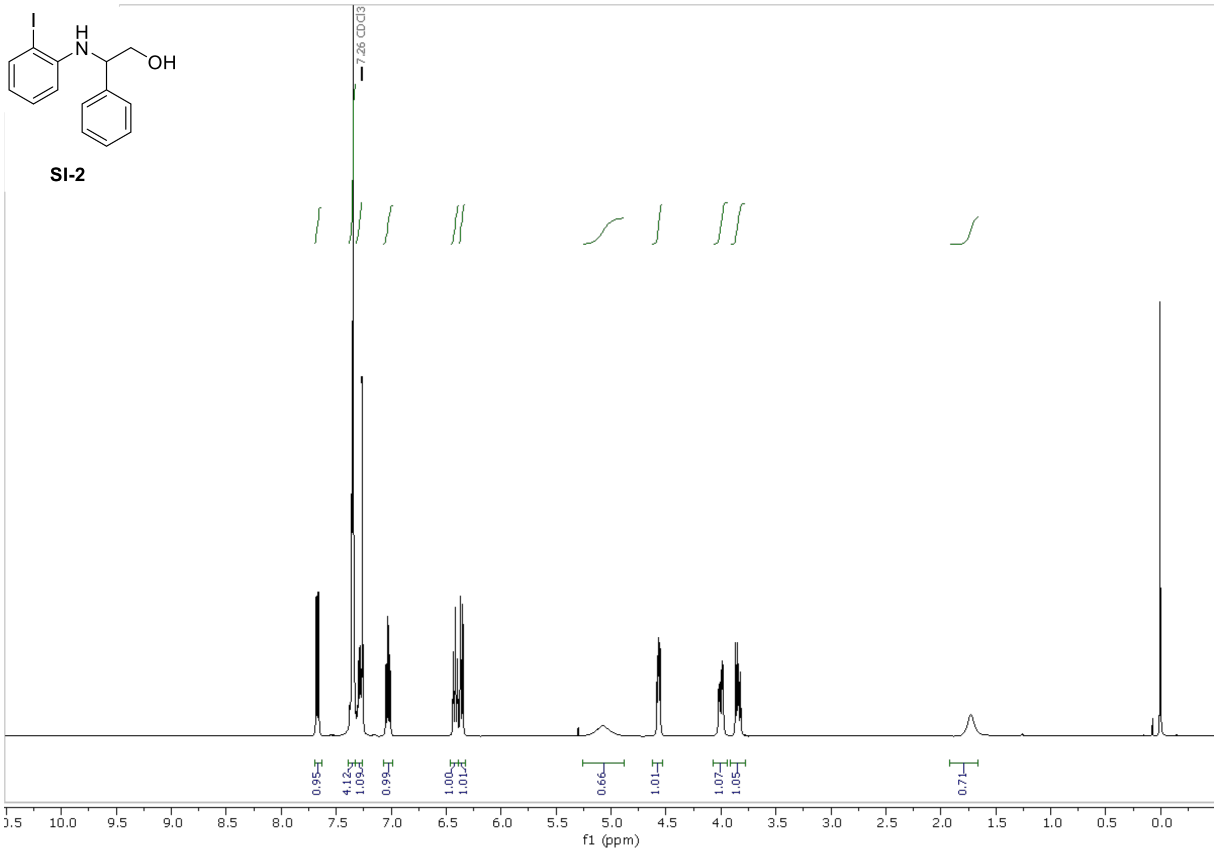

^{13}C{^{1}H} NMR (126 MHz, Chloroform-d)

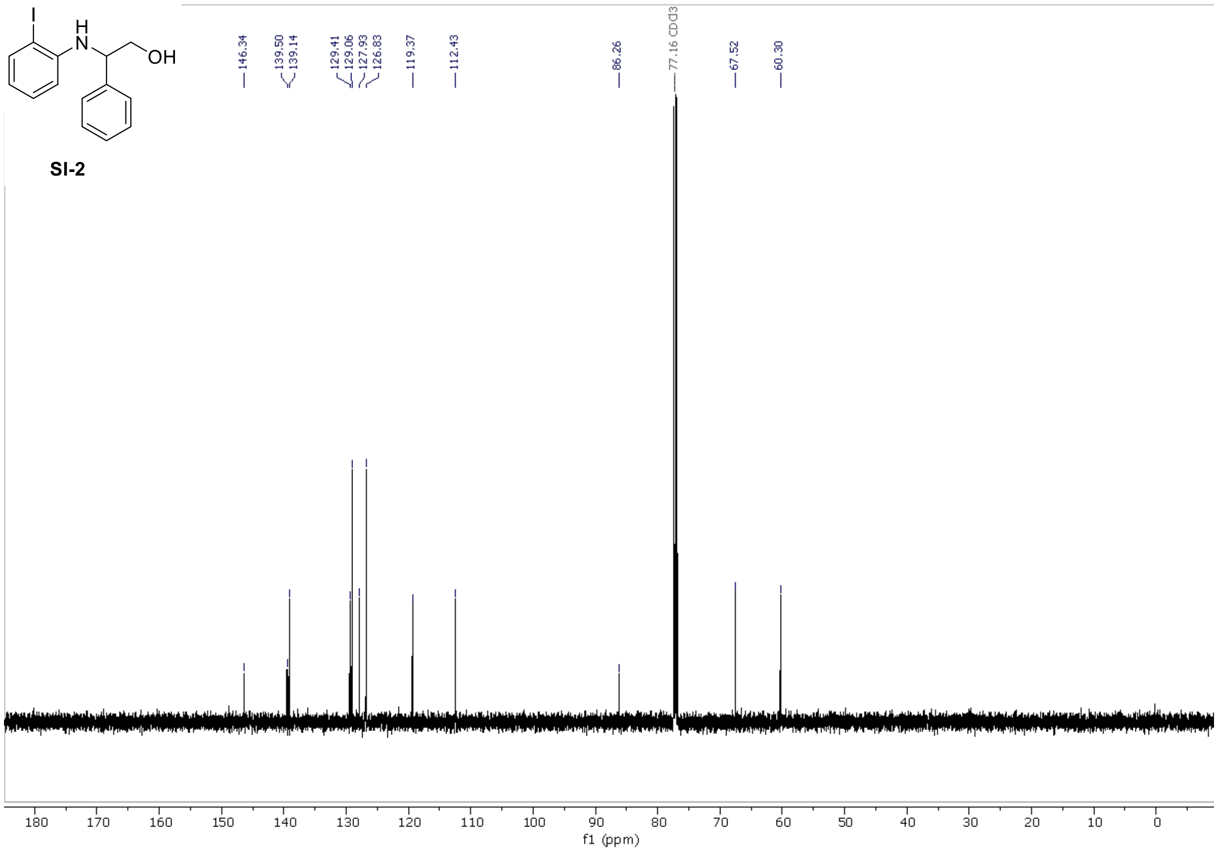

^{1}H NMR (400 MHz, Chloroform-*d*)

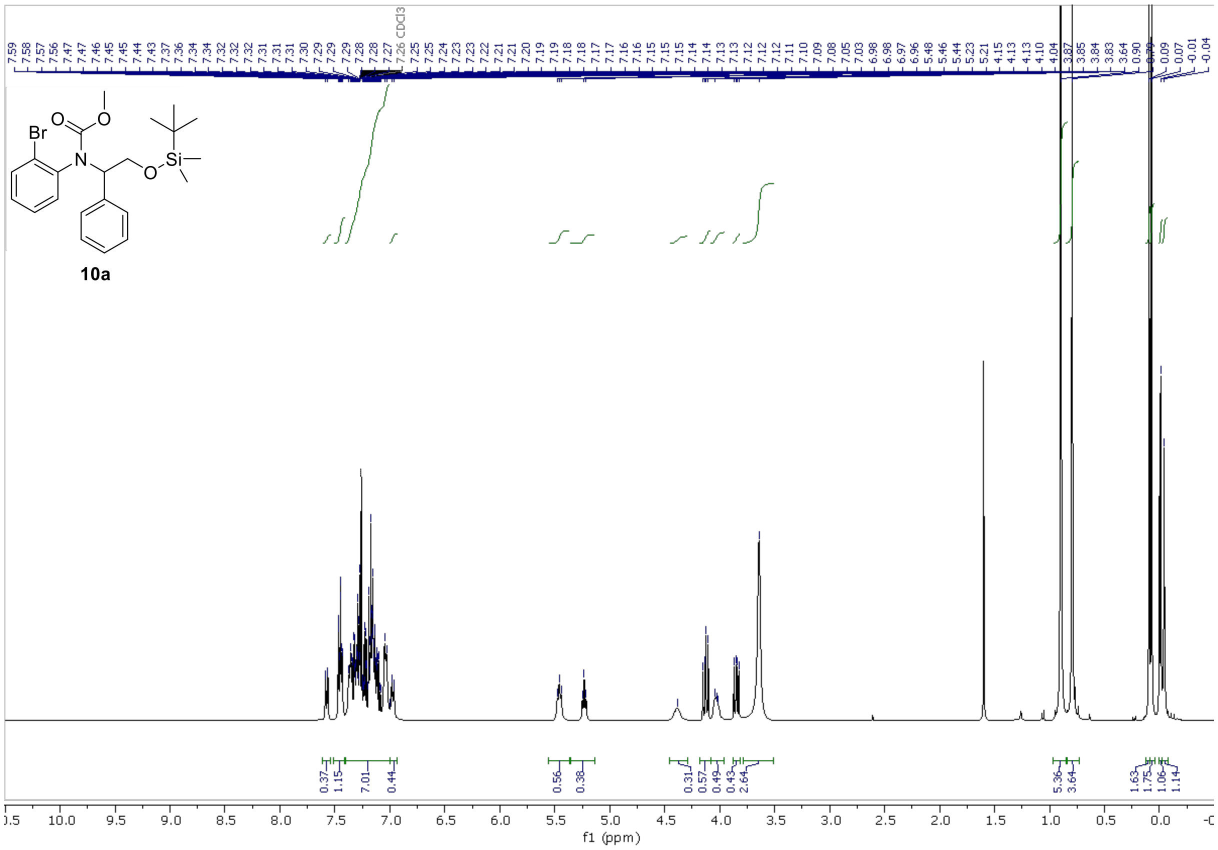

^{13}C{^{1}H} NMR (101 MHz, Chloroform-*d*)

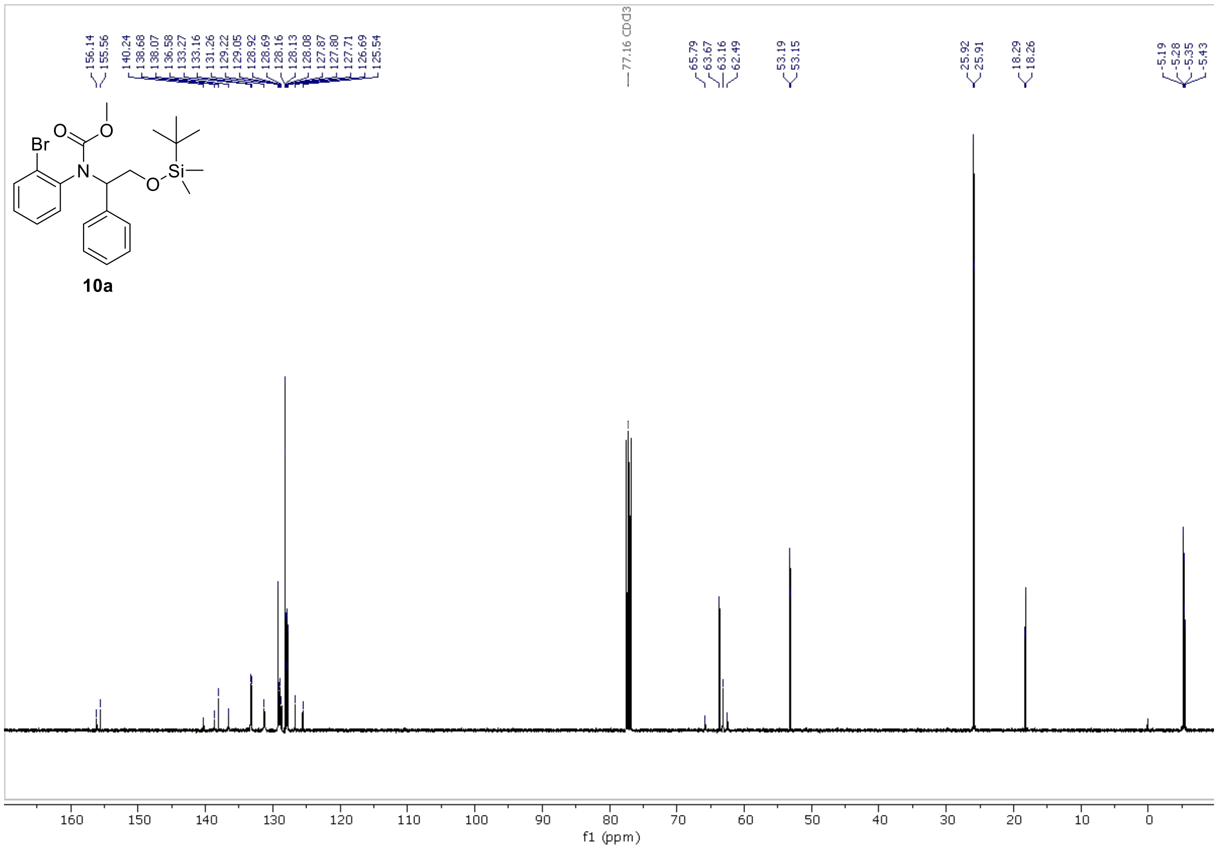

^{1}H NMR (400 MHz, Chloroform-*d*)

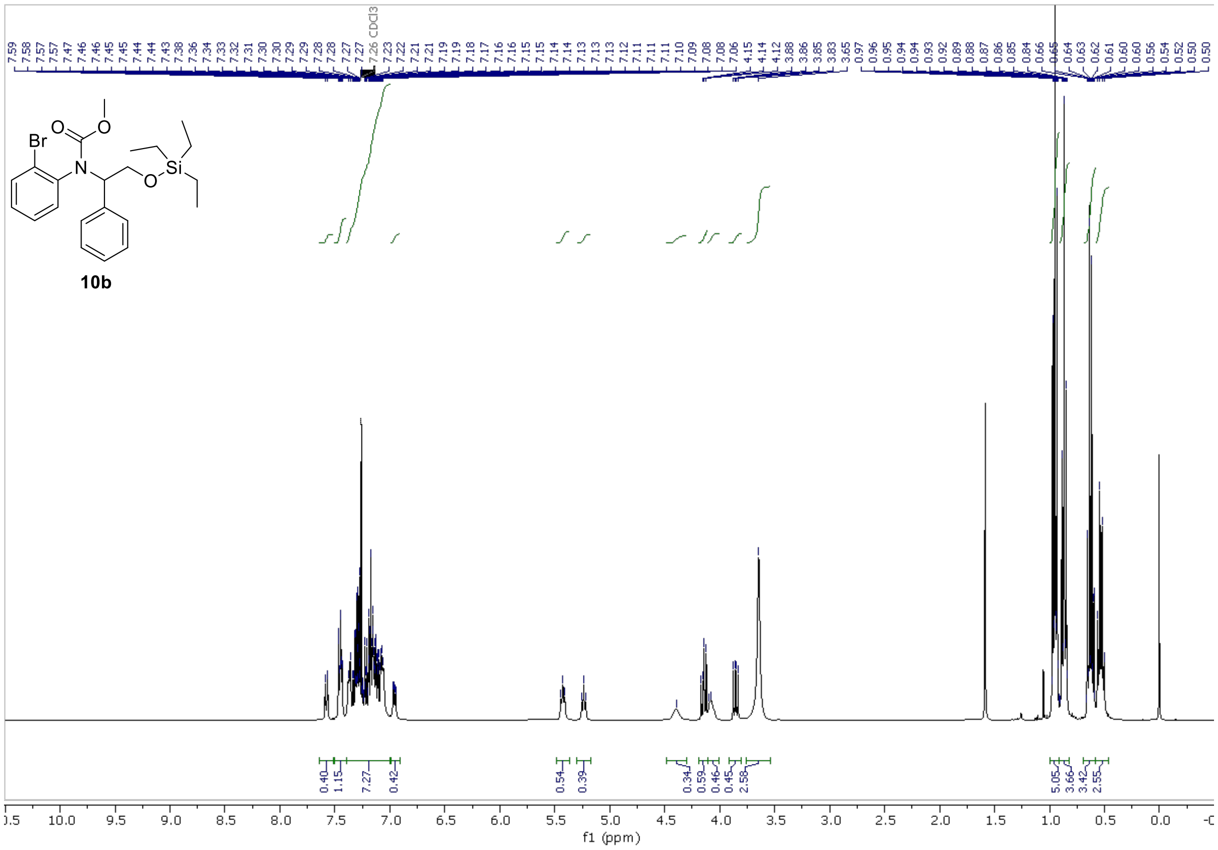

^{13}C{^{1}H} NMR (101 MHz, Chloroform-*d*)

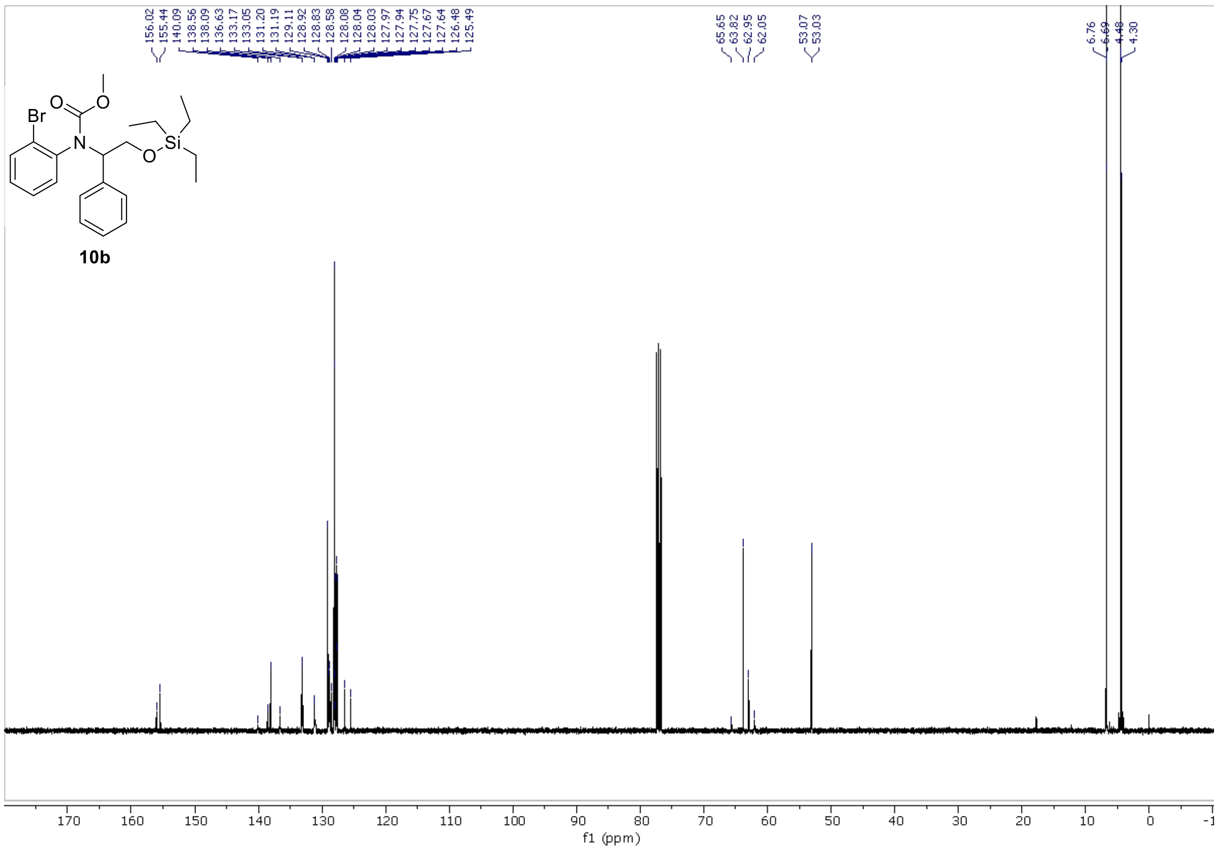

^{1}H NMR (400 MHz, Chloroform-*d*)

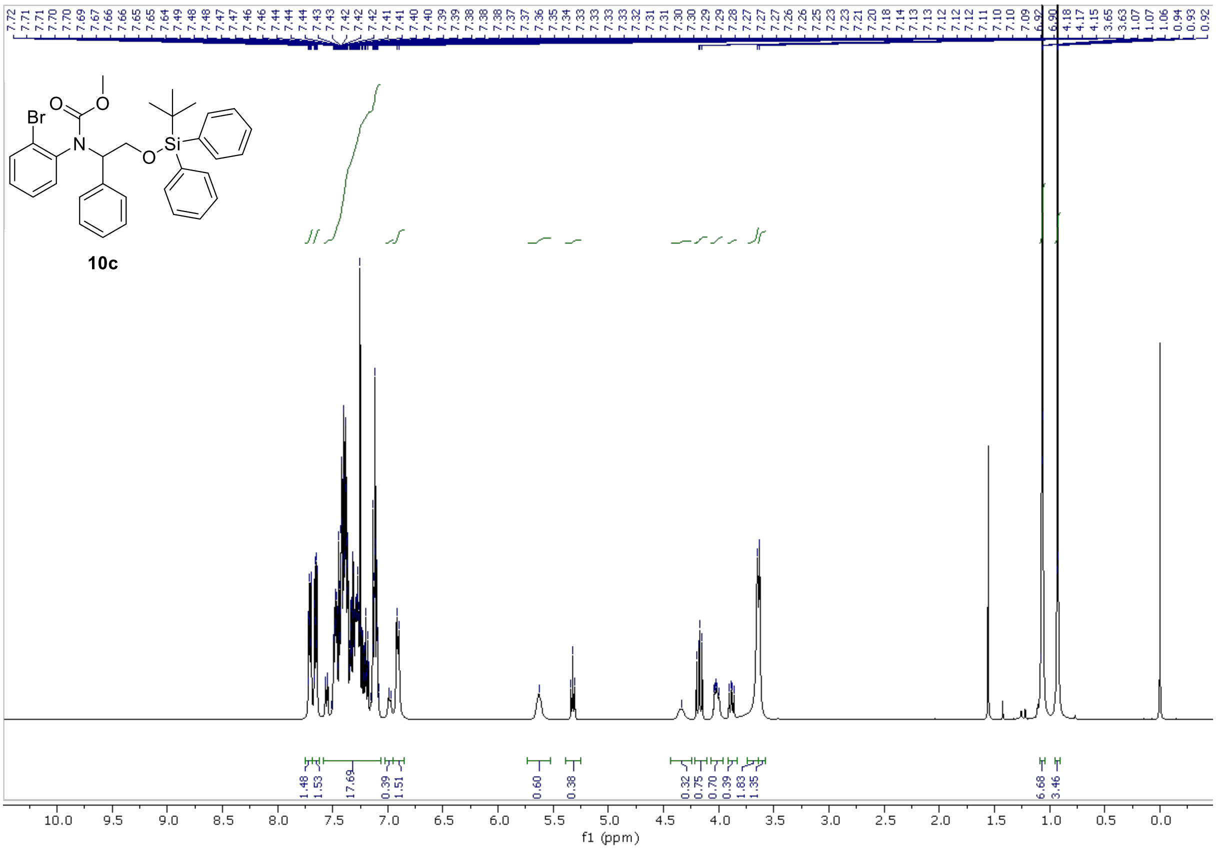

^{13}C{^{1}H} NMR (101 MHz, Chloroform-*d*)

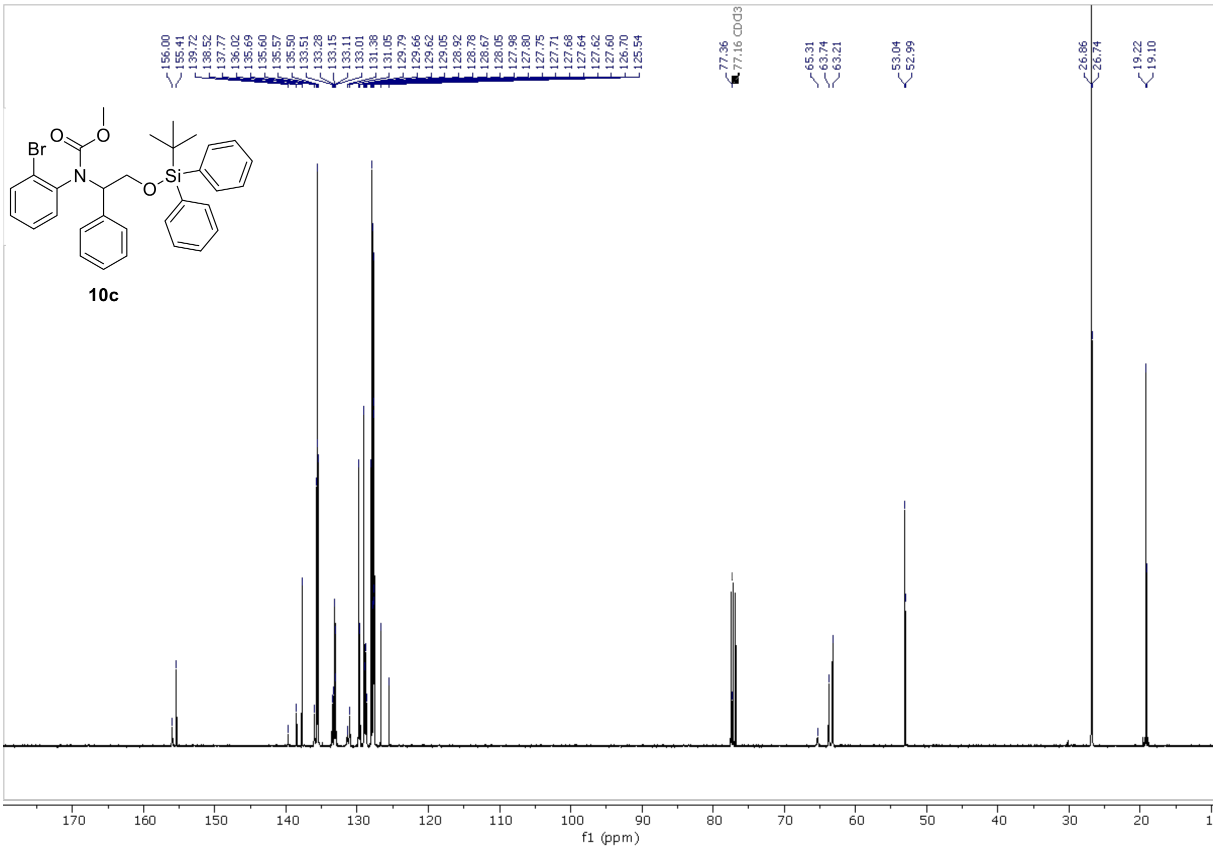

^{1}H NMR (400 MHz, Chloroform-*d*)

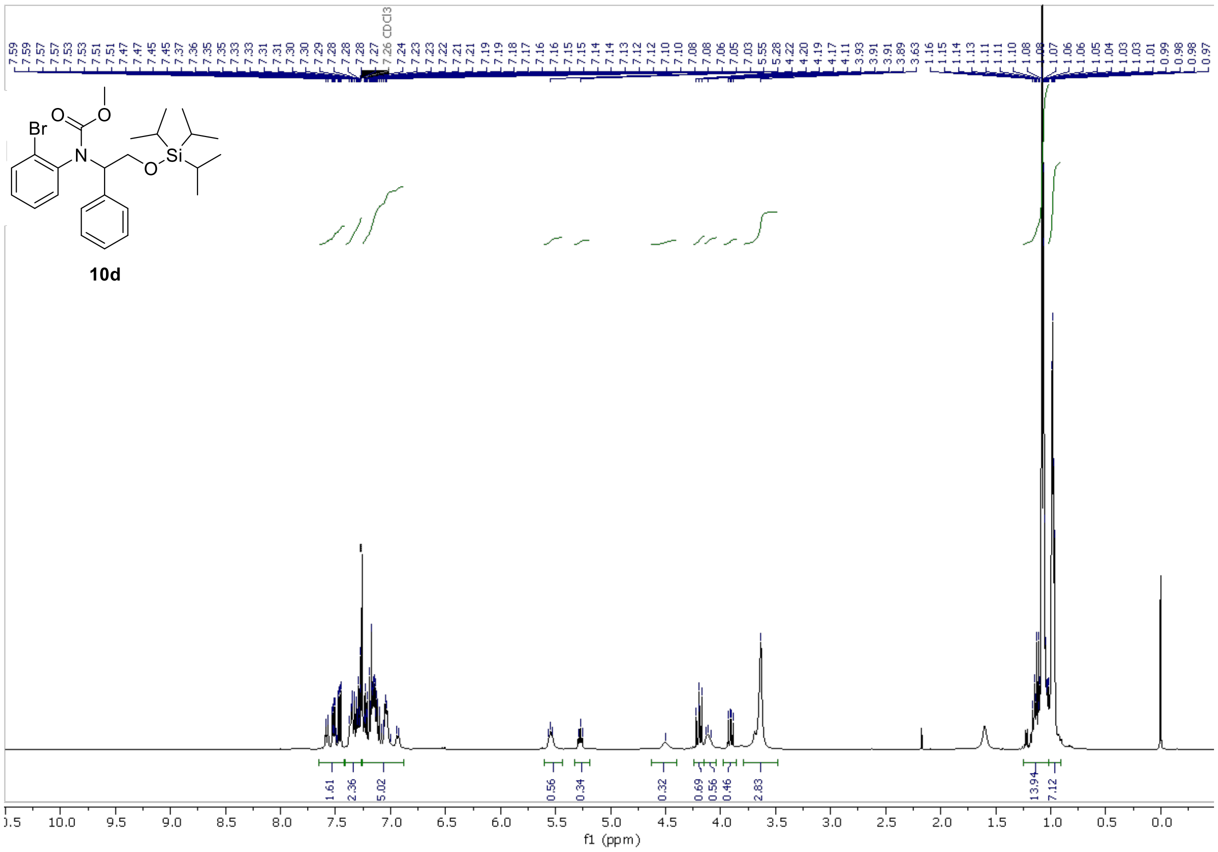

^{13}C{^{1}H} NMR (101 MHz, Chloroform-*d*)

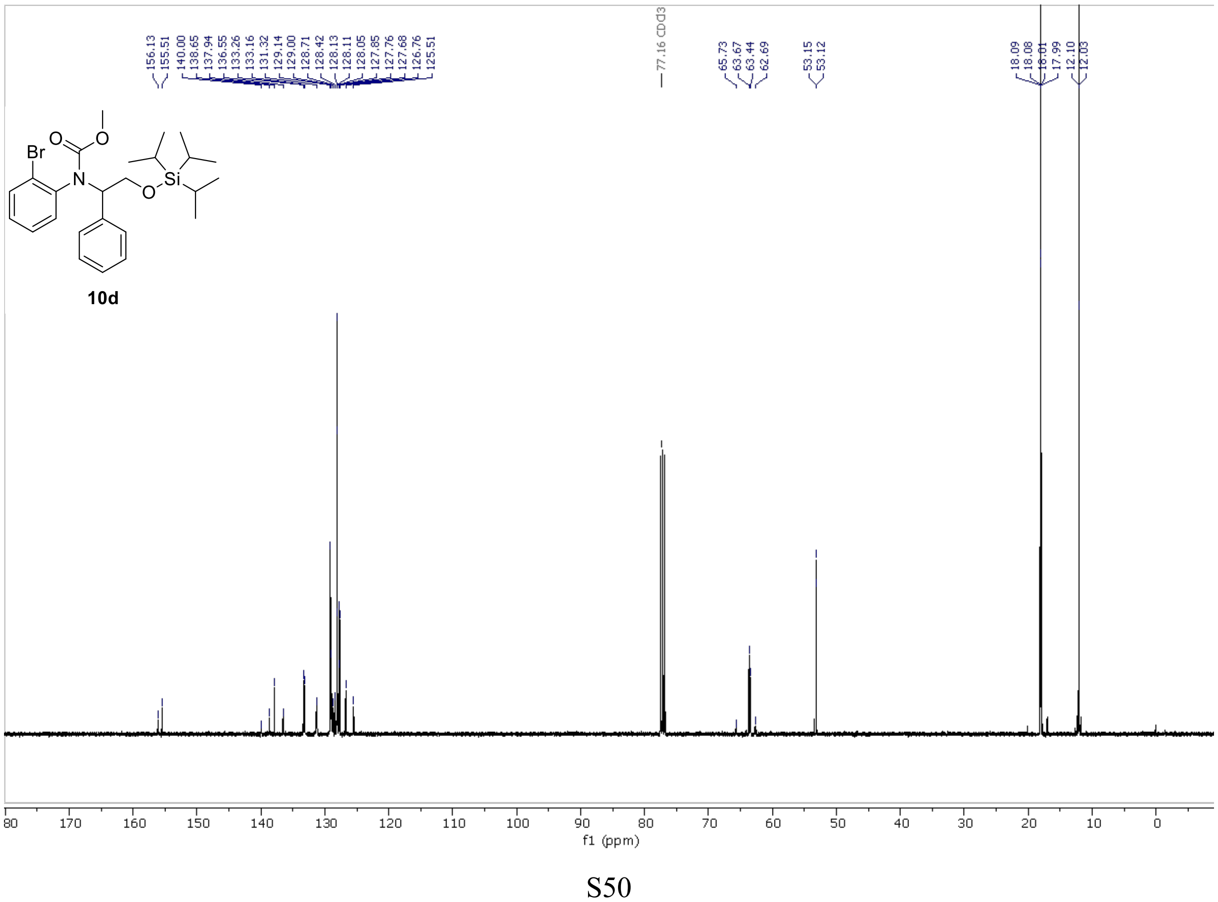

^{1}H NMR (400 MHz, Chloroform-*d*)

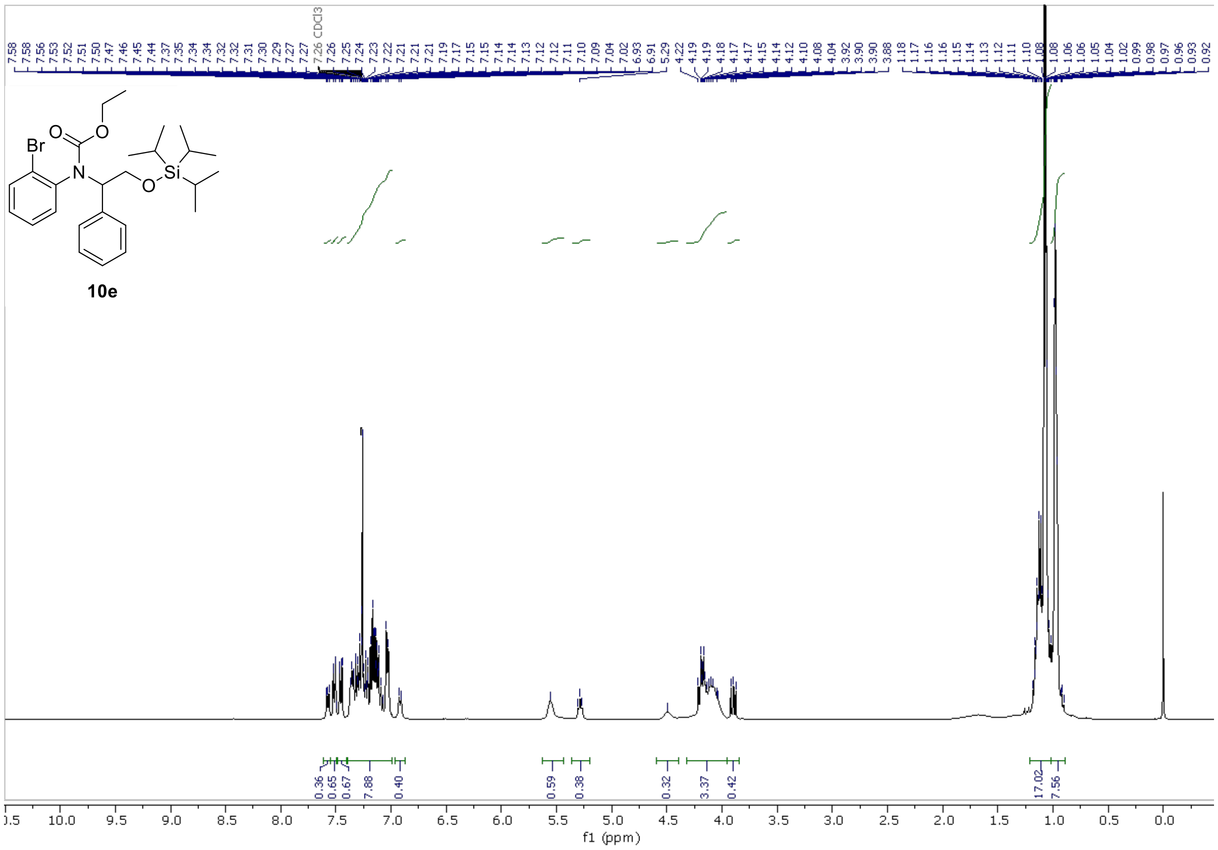

^{13}C{^{1}H} NMR (101 MHz, Chloroform-*d*)

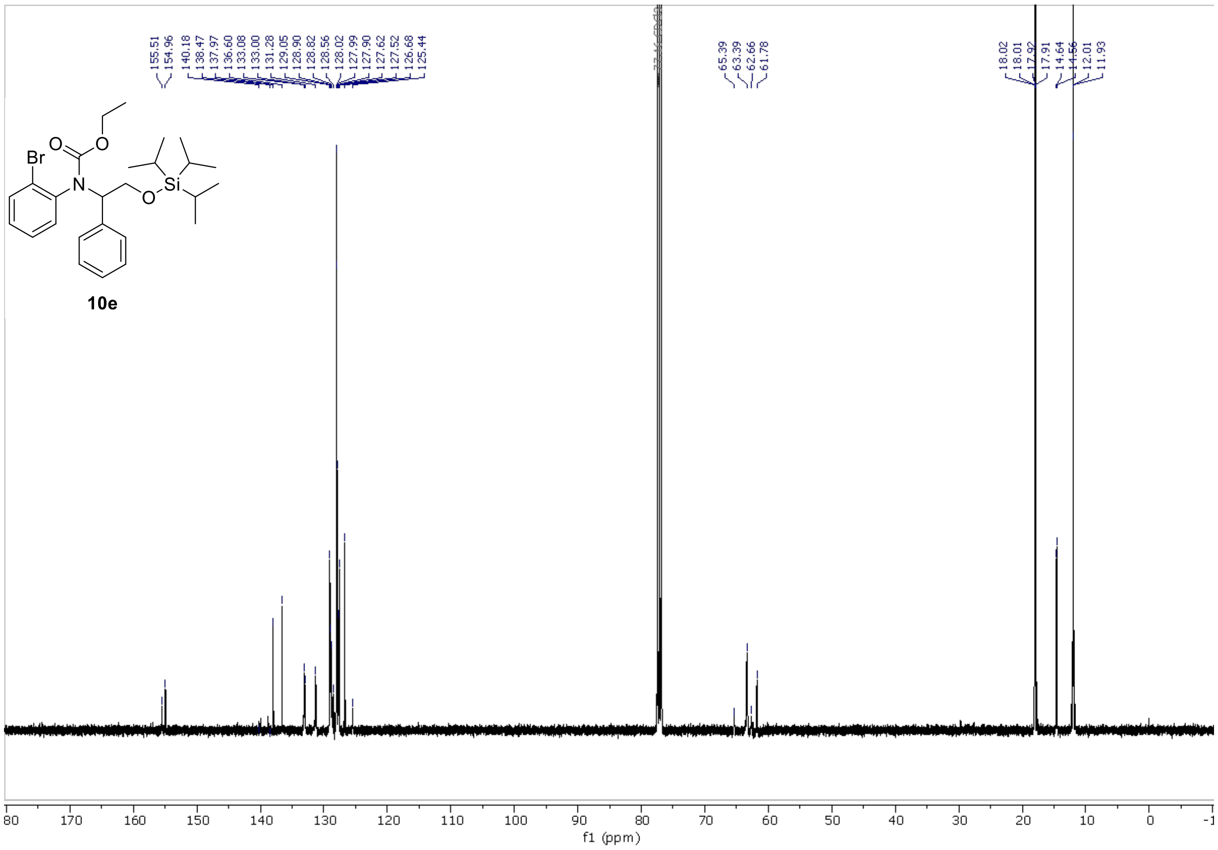

^{1}**H NMR** (500 MHz, Chloroform-*d*)

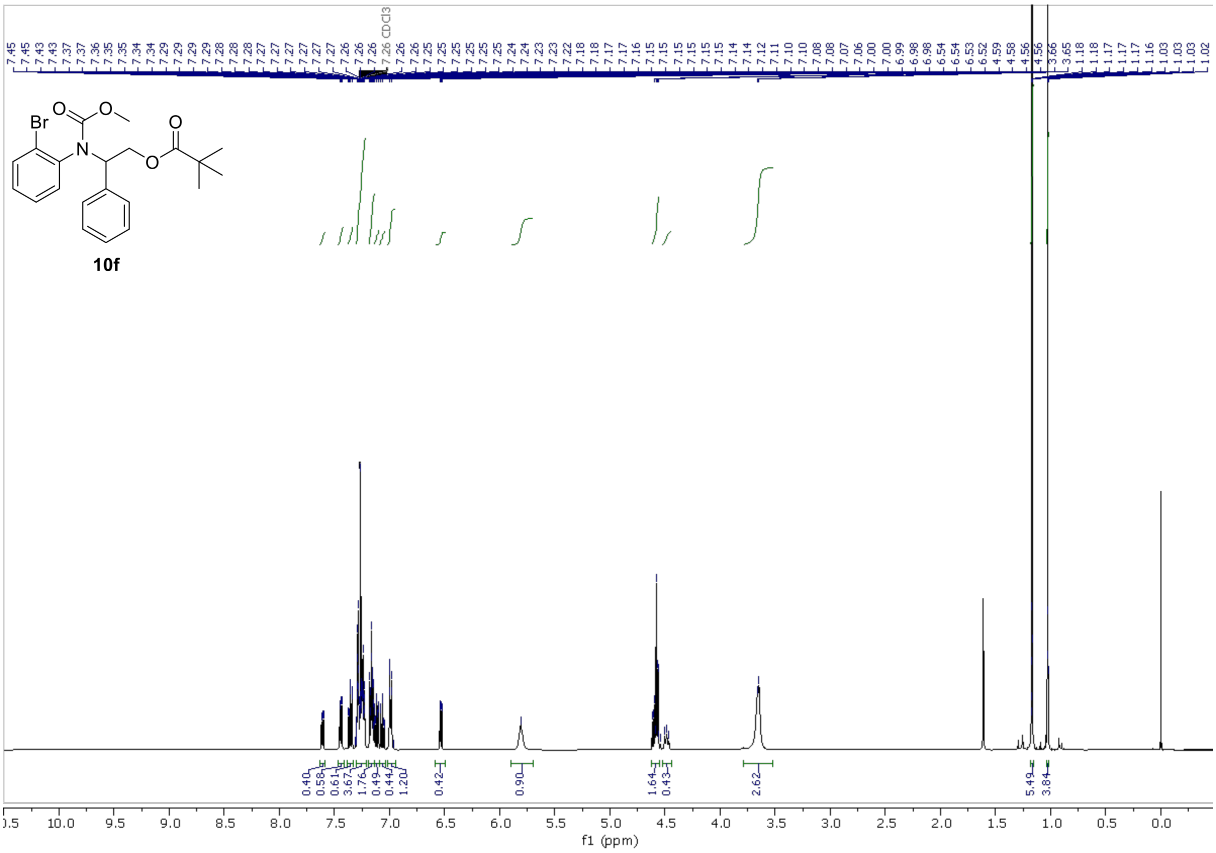

^{13}**C{^{1}H} NMR** (101 MHz, Chloroform-*d*)

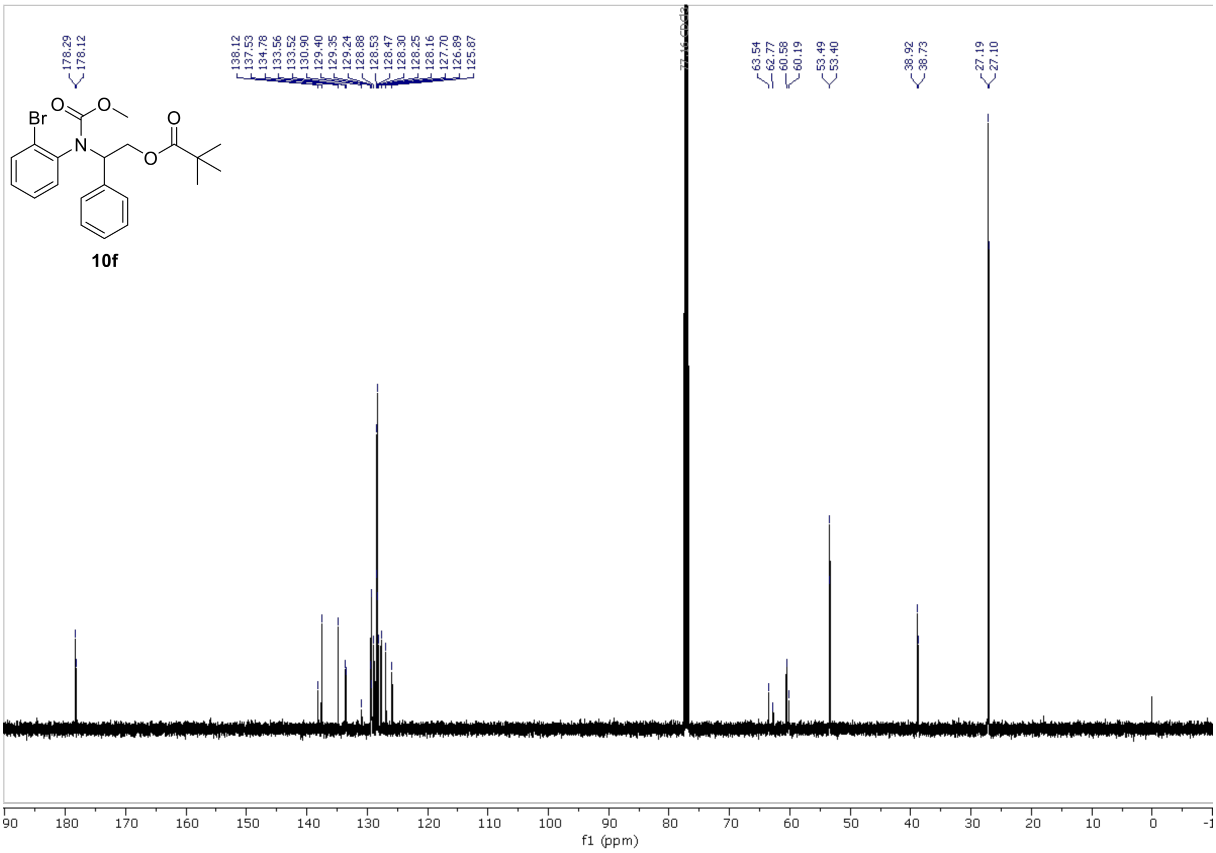

^{1}H NMR (400 MHz, Chloroform-*d*)

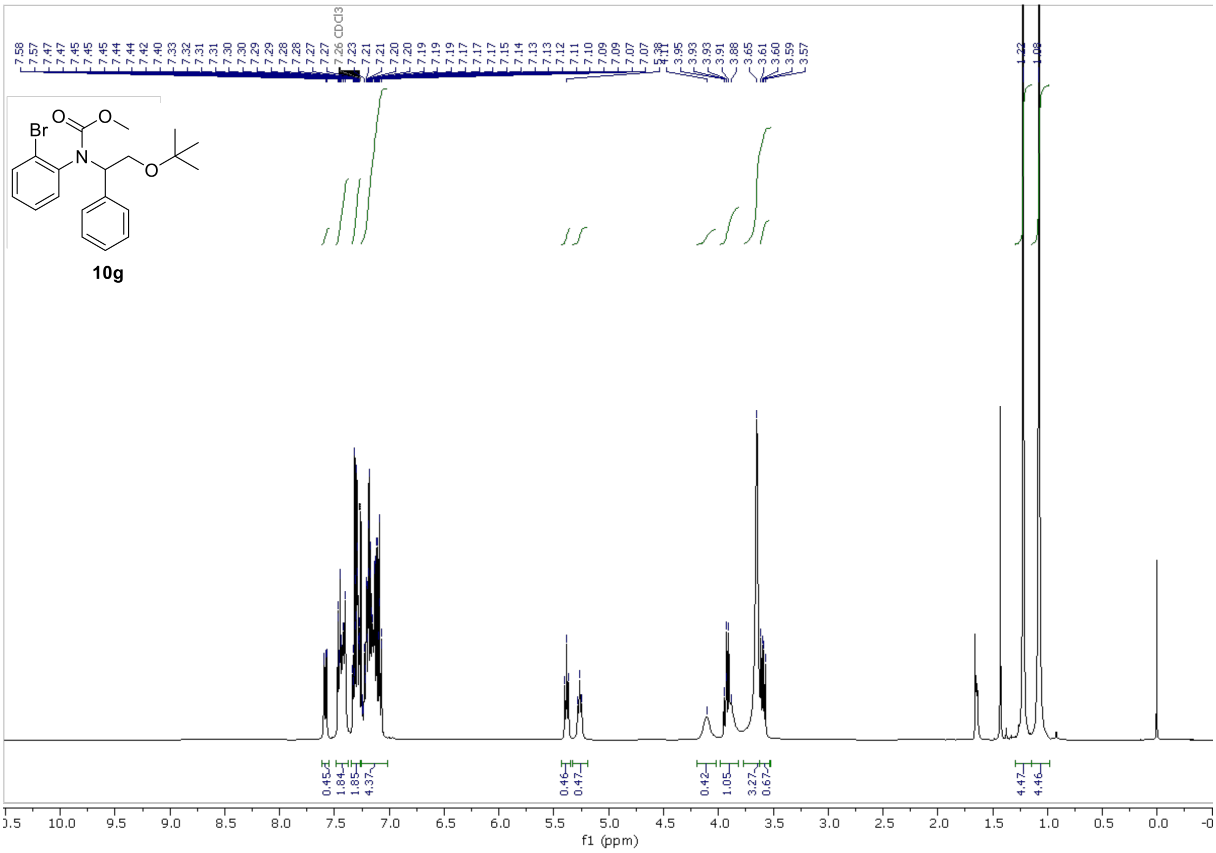

^{13}C{^{1}H} NMR (101 MHz, Chloroform-*d*)

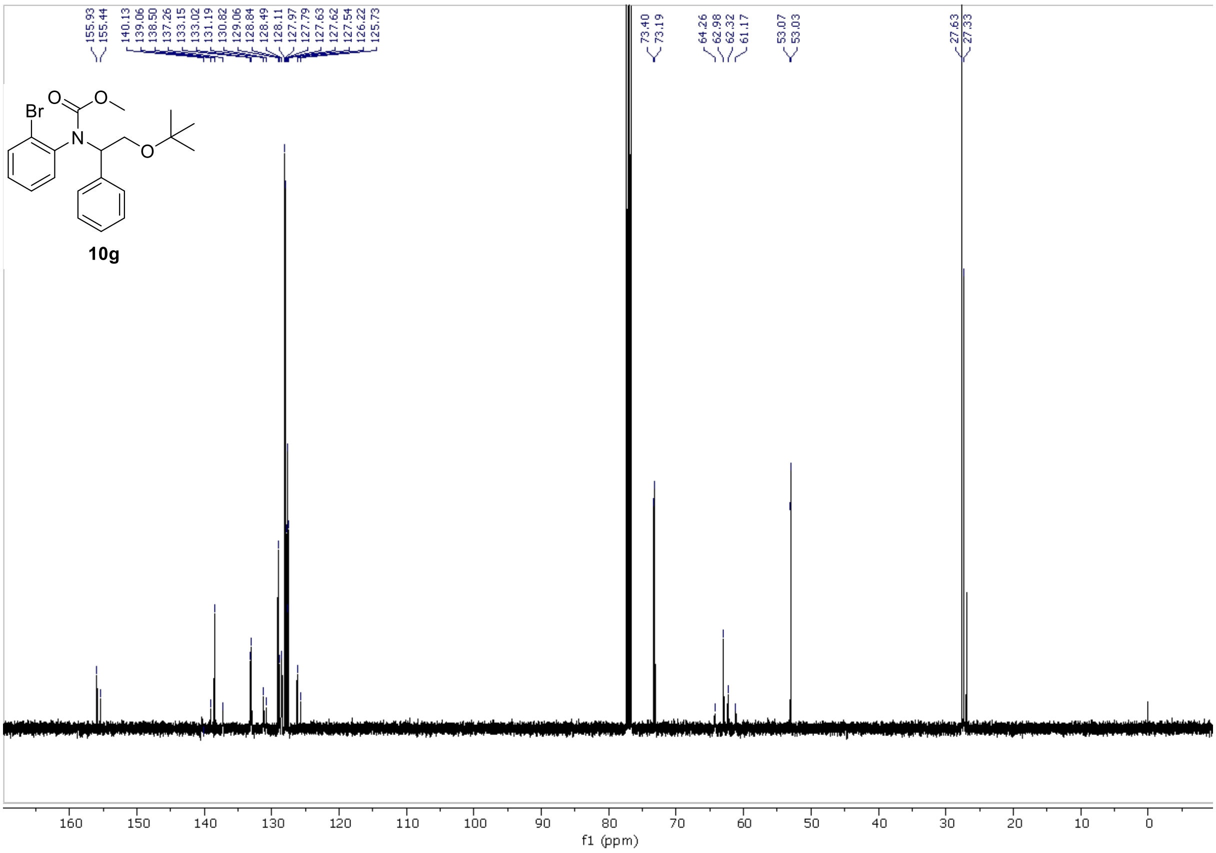

^{1}H NMR (400 MHz, Chloroform-*d*)

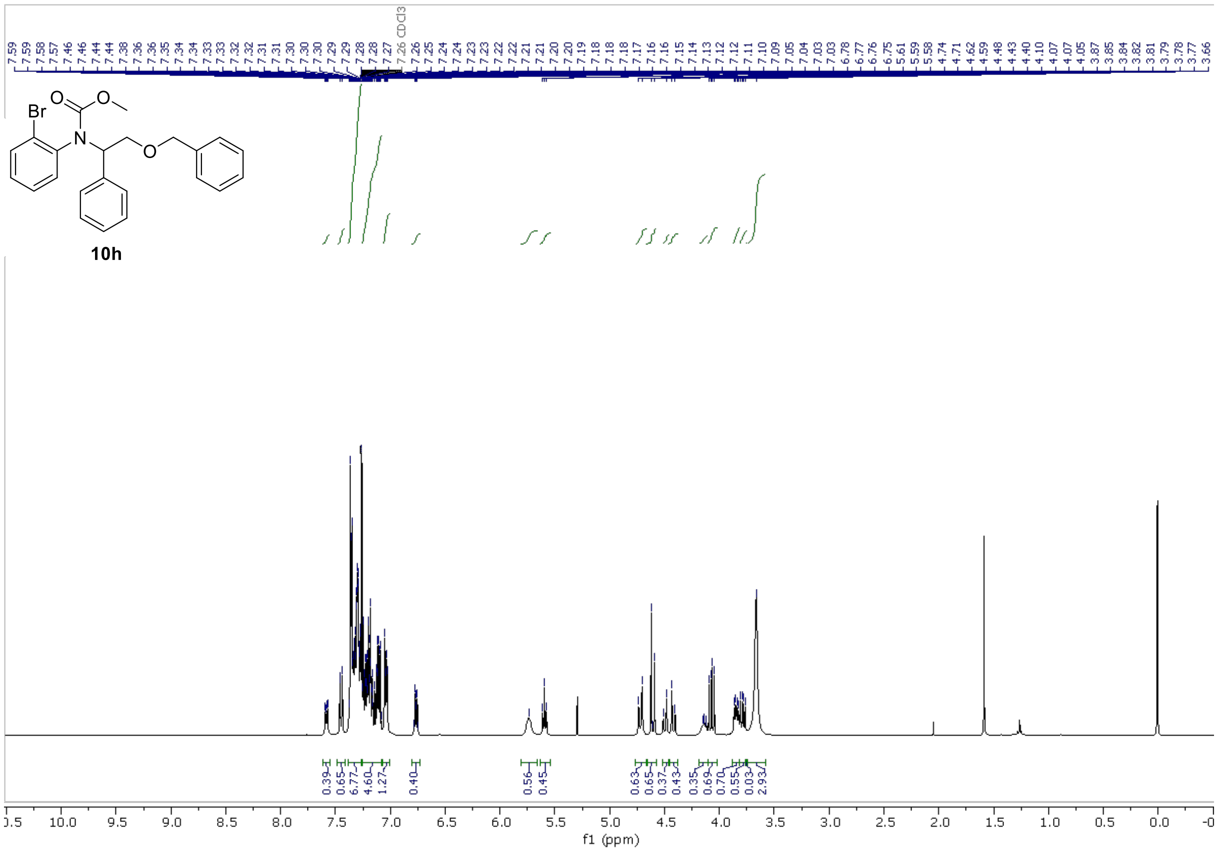

^{13}C{^{1}H} NMR (101 MHz, Chloroform-*d*)

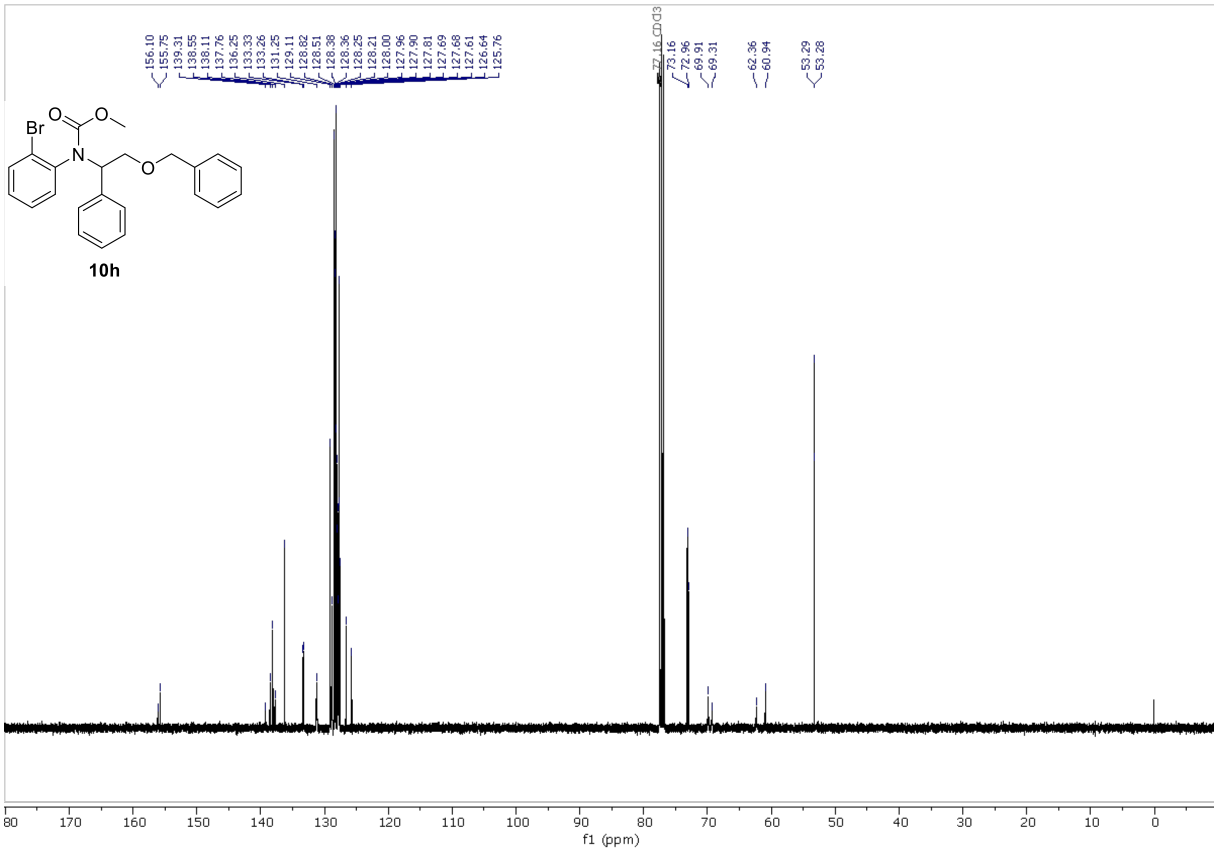

^{1}H NMR (400 MHz, Chloroform-*d*)

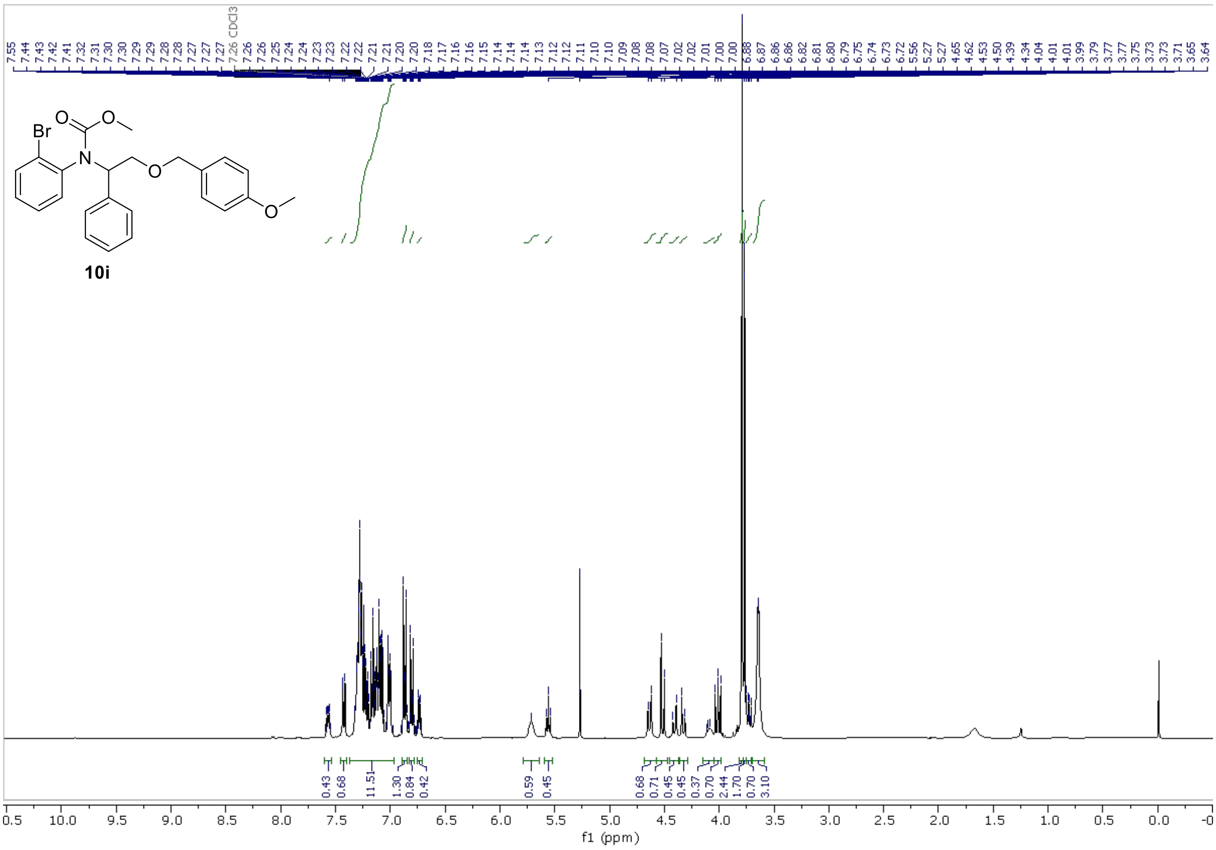

^{13}C{^{1}H} NMR (101 MHz, Chloroform-*d*)

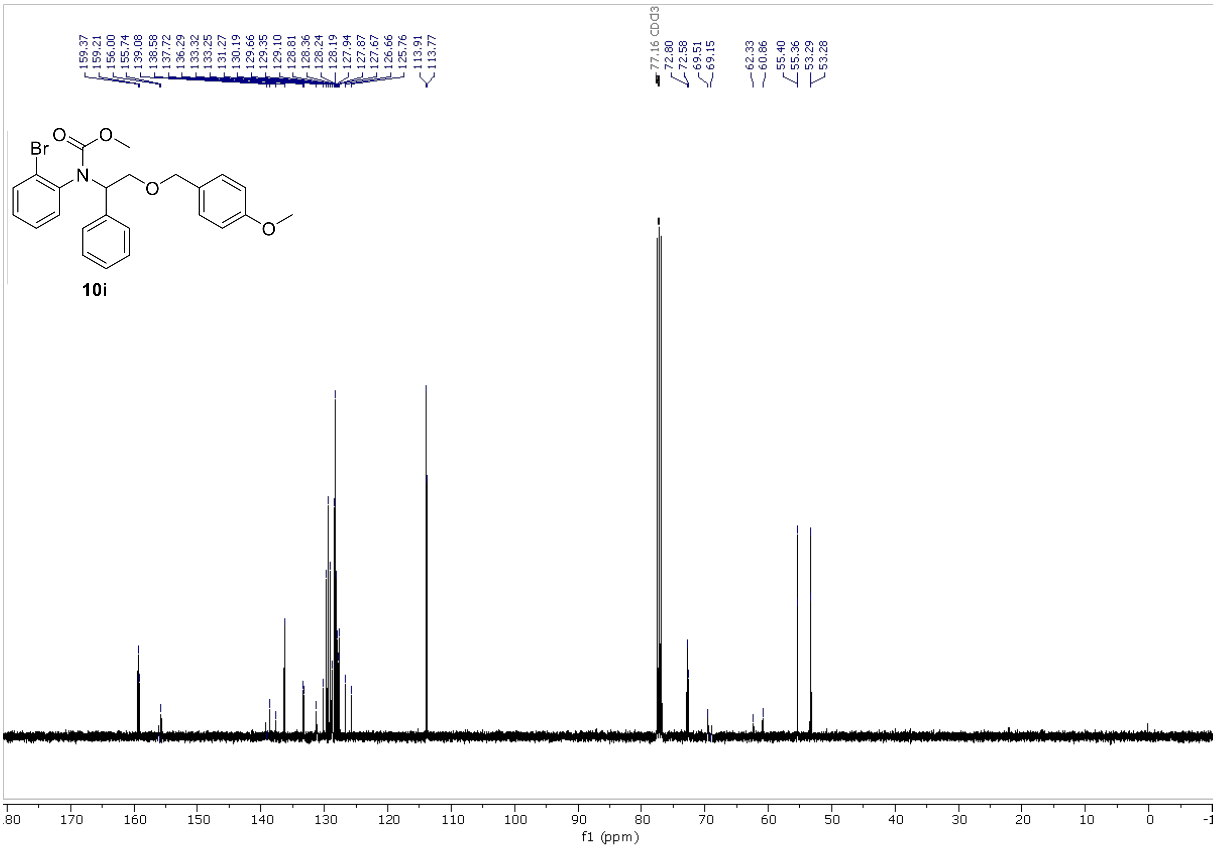

¹H NMR (400 MHz, Chloroform-*d*)

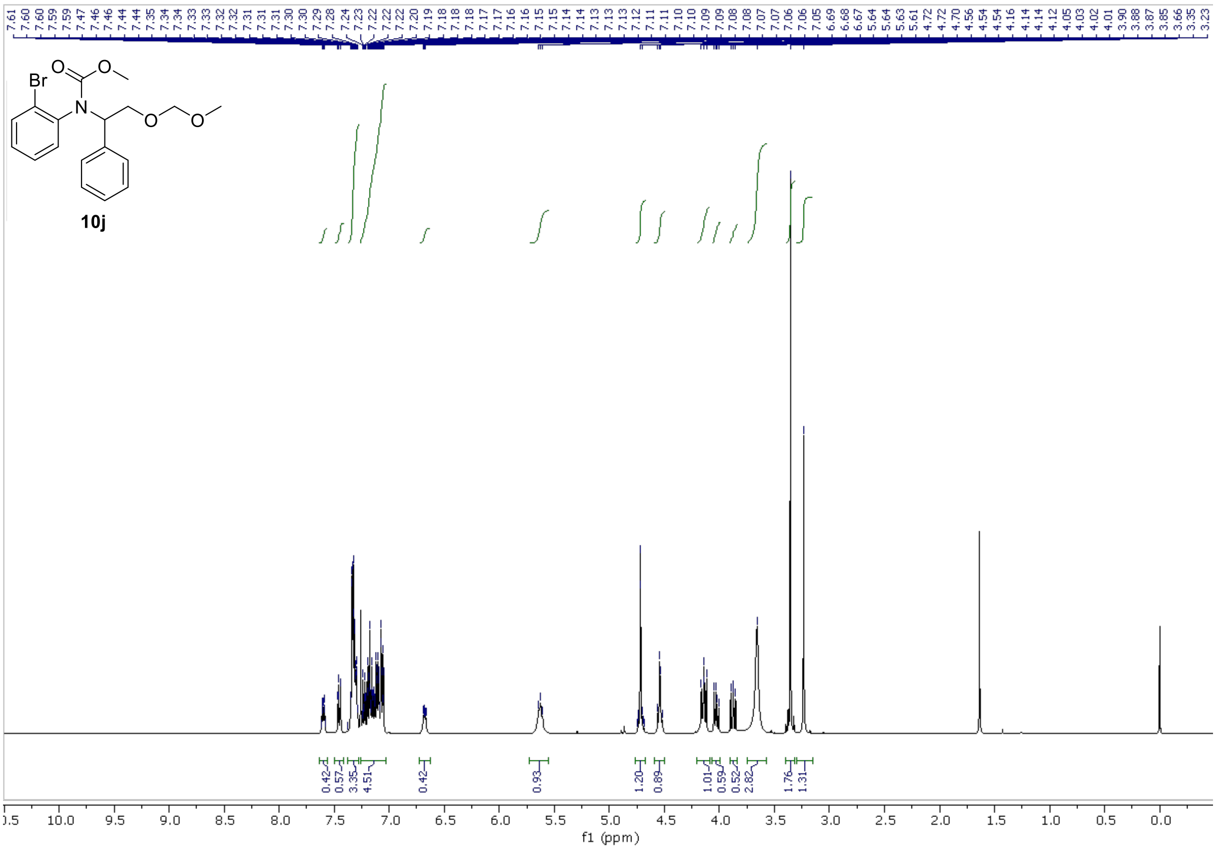

¹³C{¹H} NMR (101 MHz, Chloroform-*d*)

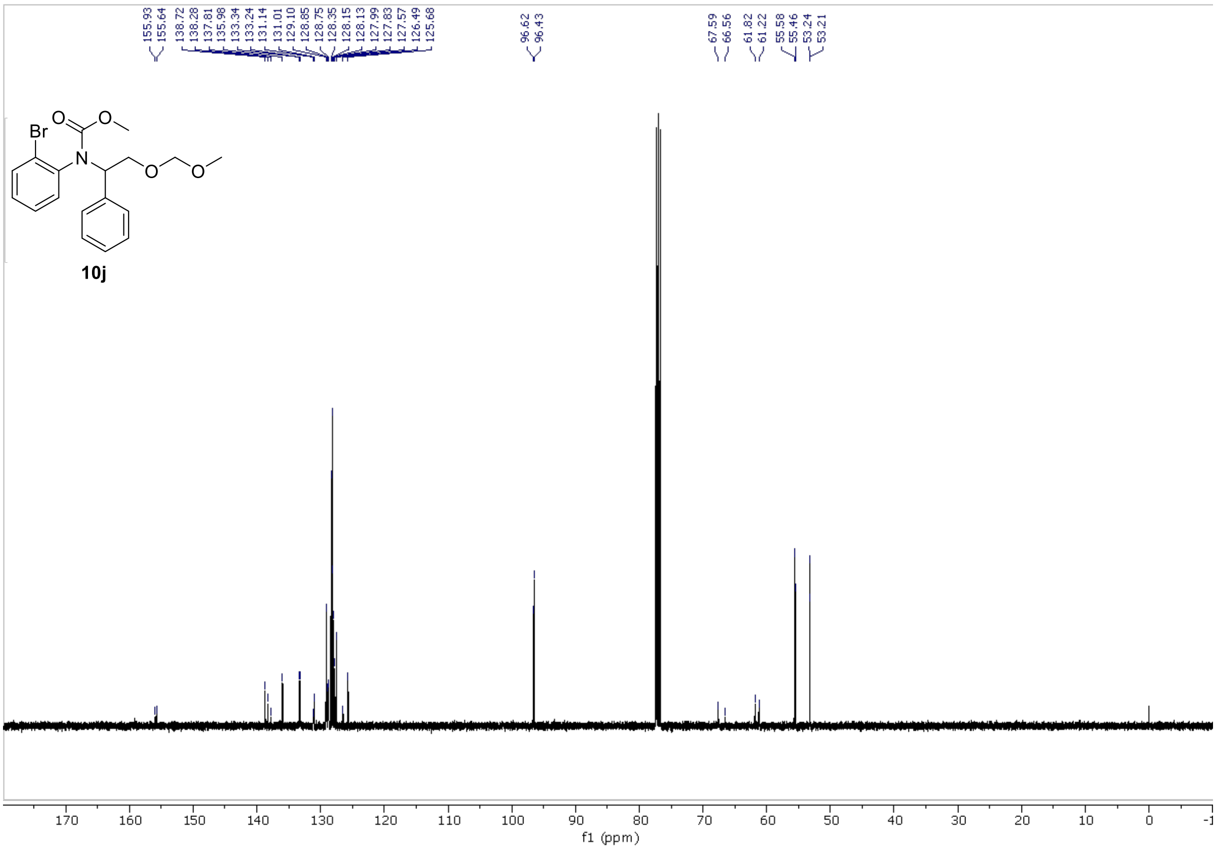

^{1}**H NMR** (500 MHz, Chloroform-*d*)

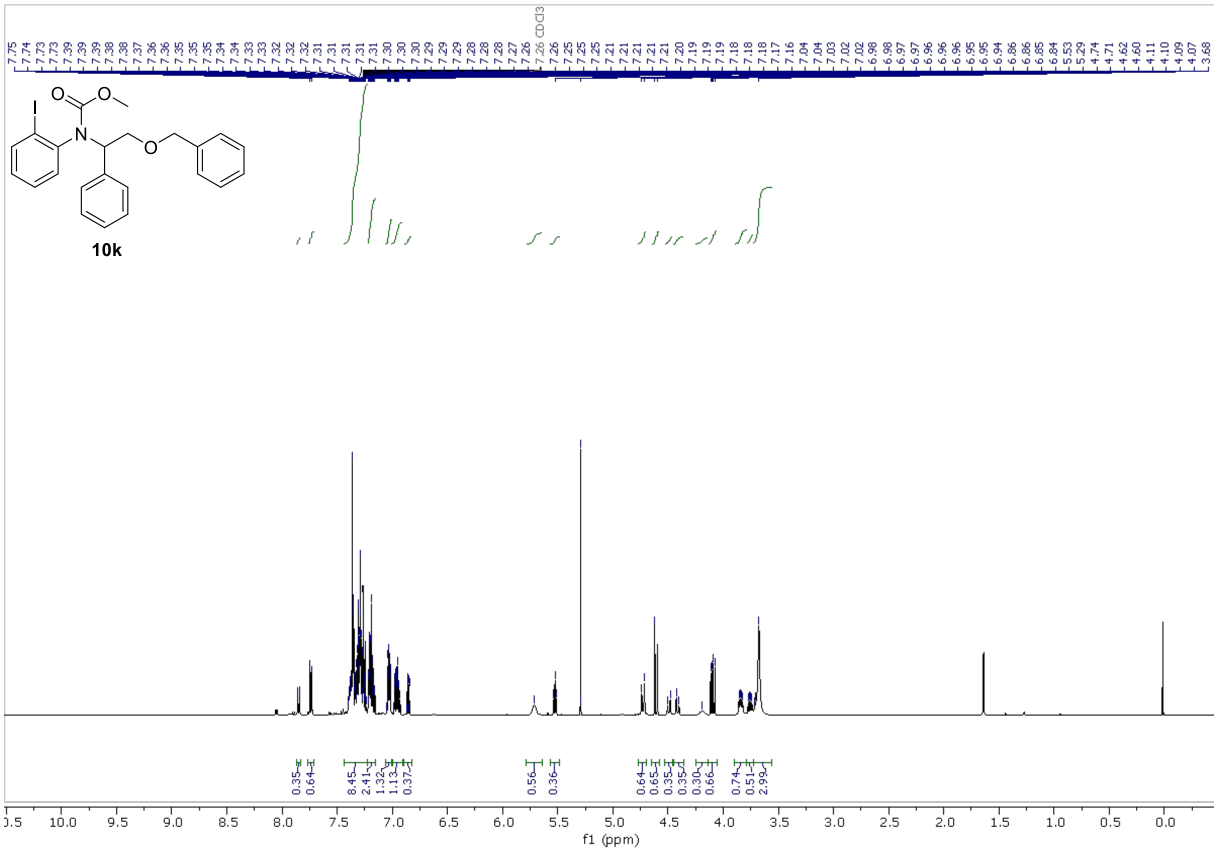

^{13}**C{^{1}H} NMR** (101 MHz, Chloroform-*d*)

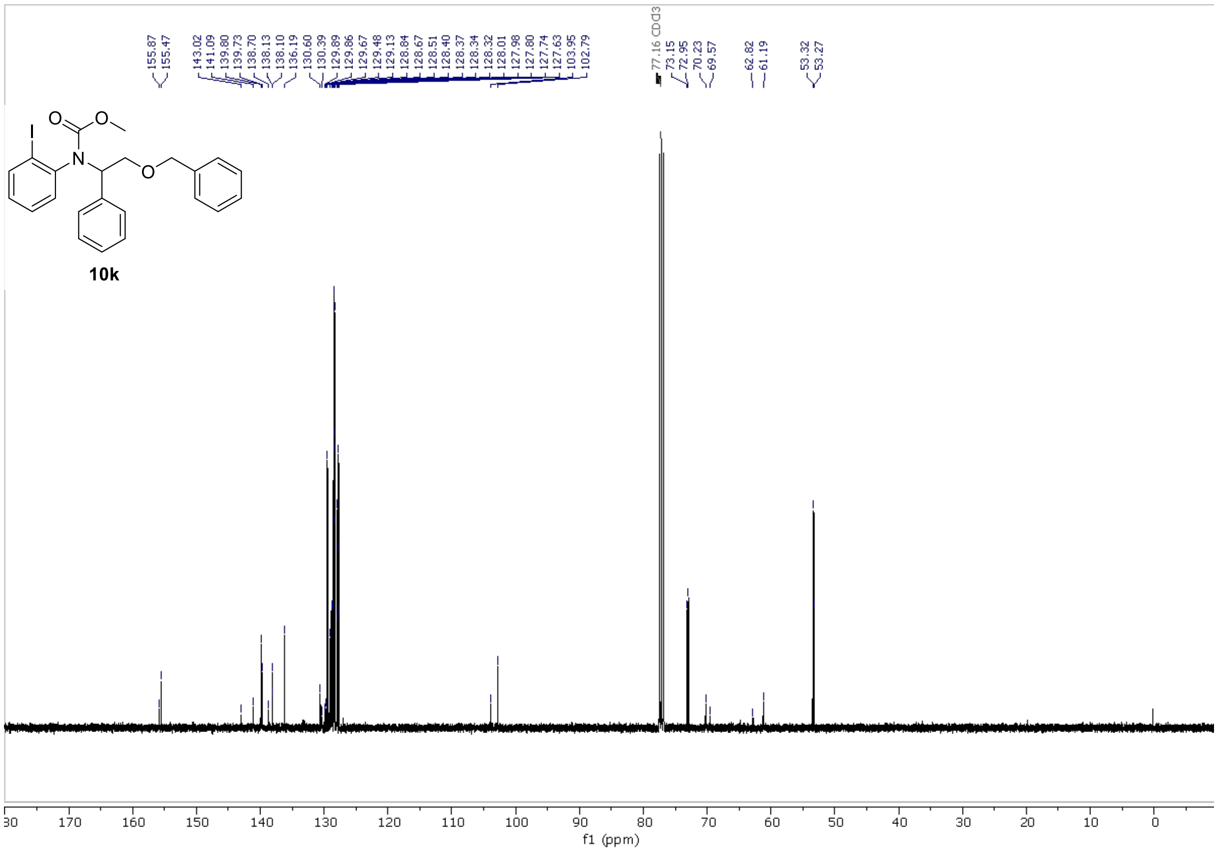

^{1}**H NMR** (400 MHz, Chloroform-*d*)

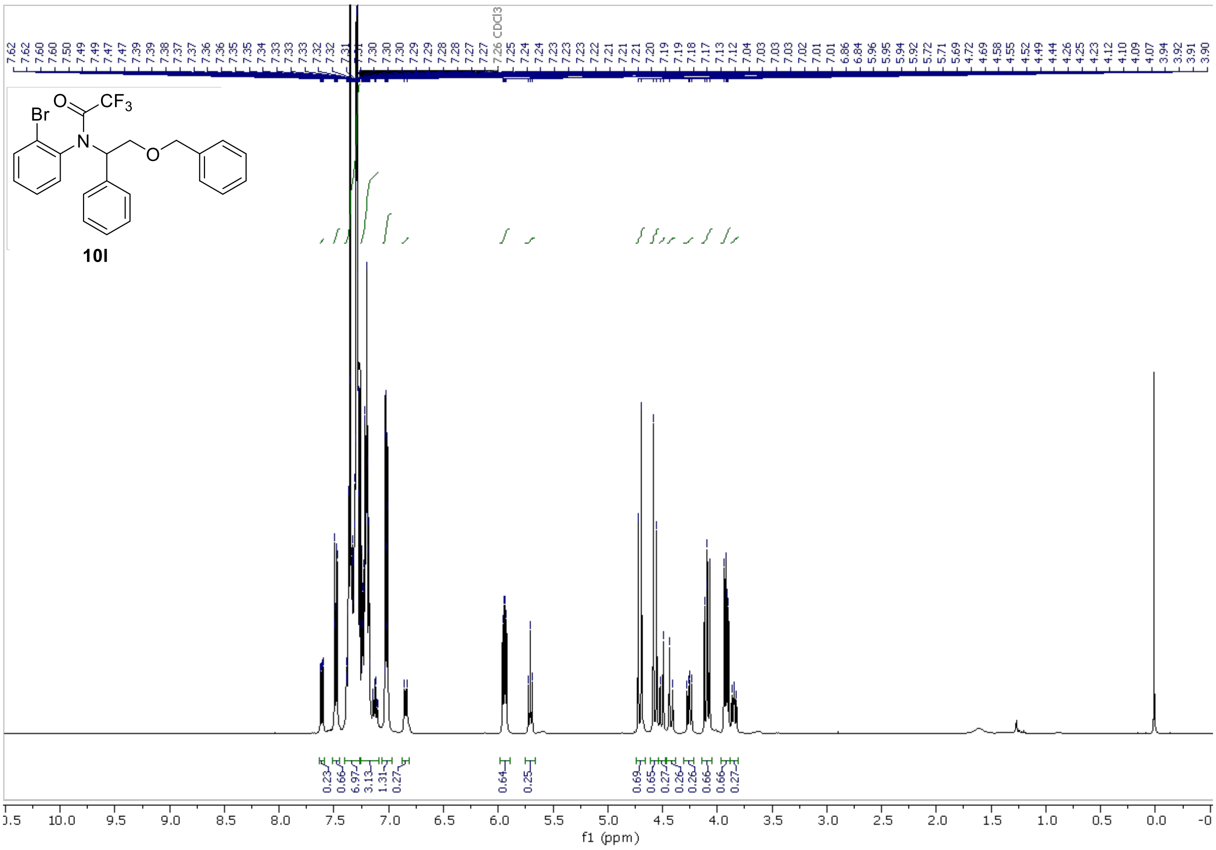

^{13}**C{^{1}H} NMR** (126 MHz, Chloroform-*d*)

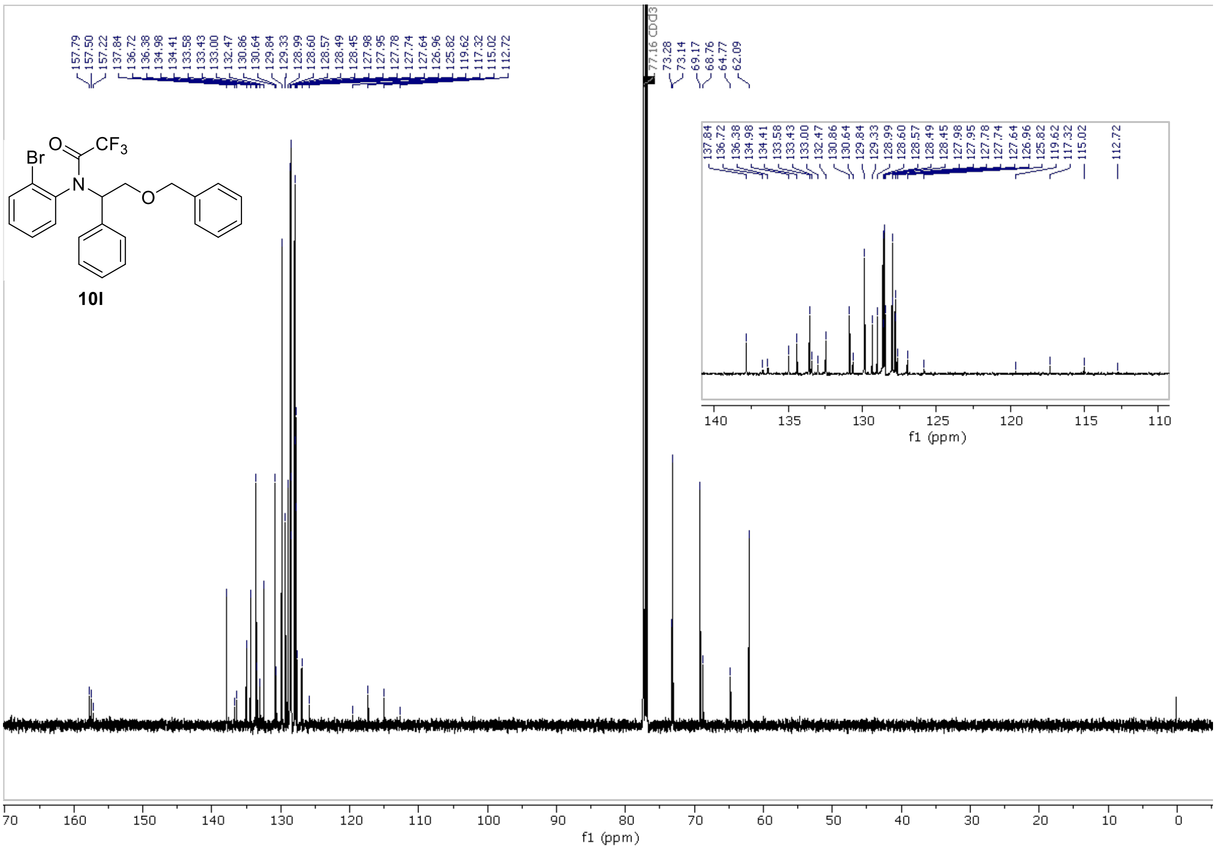

$^{19}F\{^{1}H\}$ NMR (376 MHz, Chloroform-*d*)

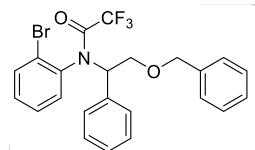

10l

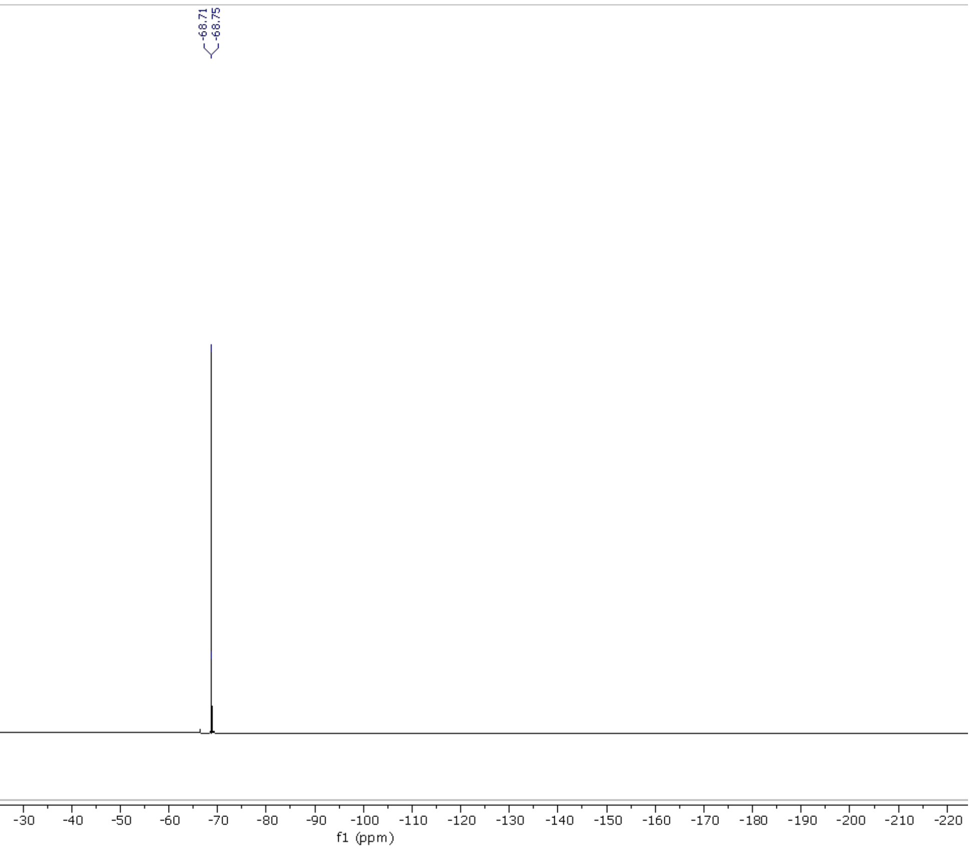

NMR and HPLC Spectrum Data of C–H Products in Kinetic Resolution

^{1}H NMR (400 MHz, Chloroform-*d*)

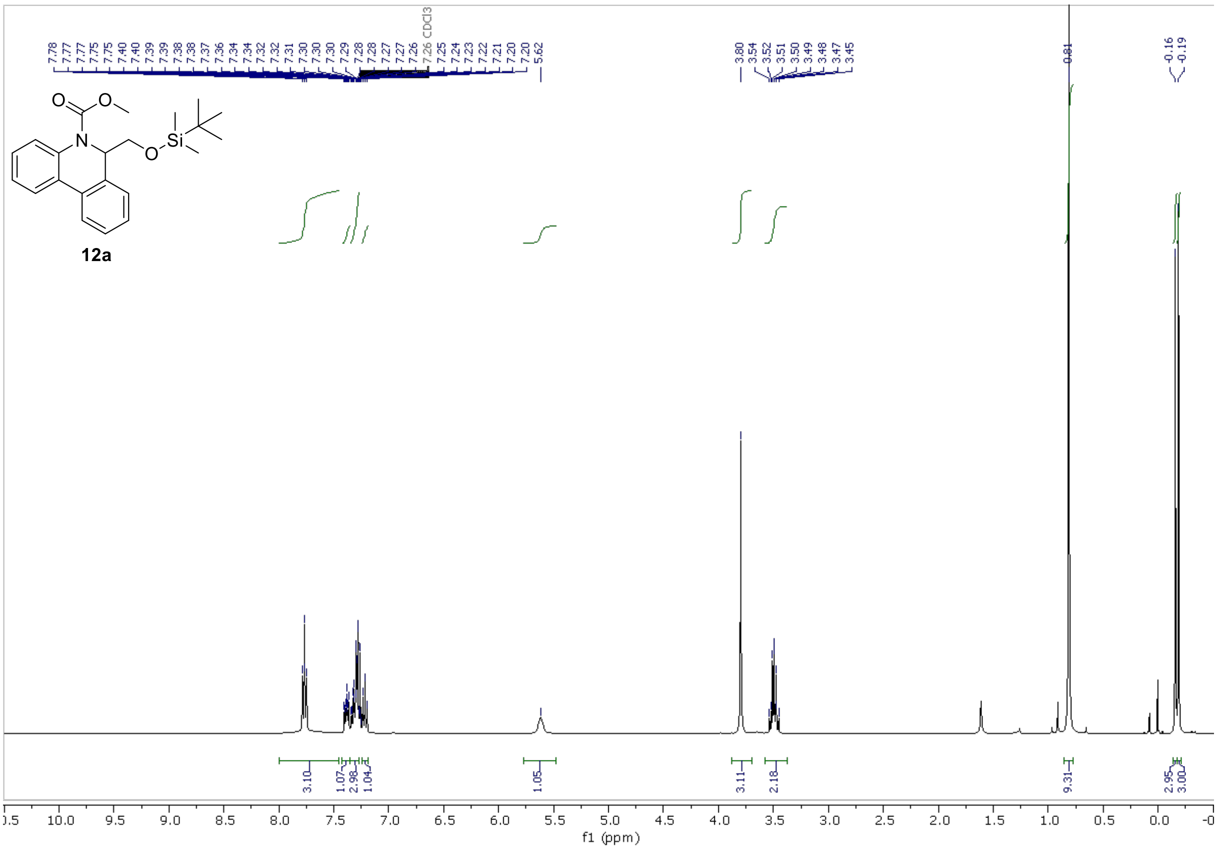

$^{13}C\{^{1}H\}$ **NMR** (126 MHz, Chloroform-*d*)

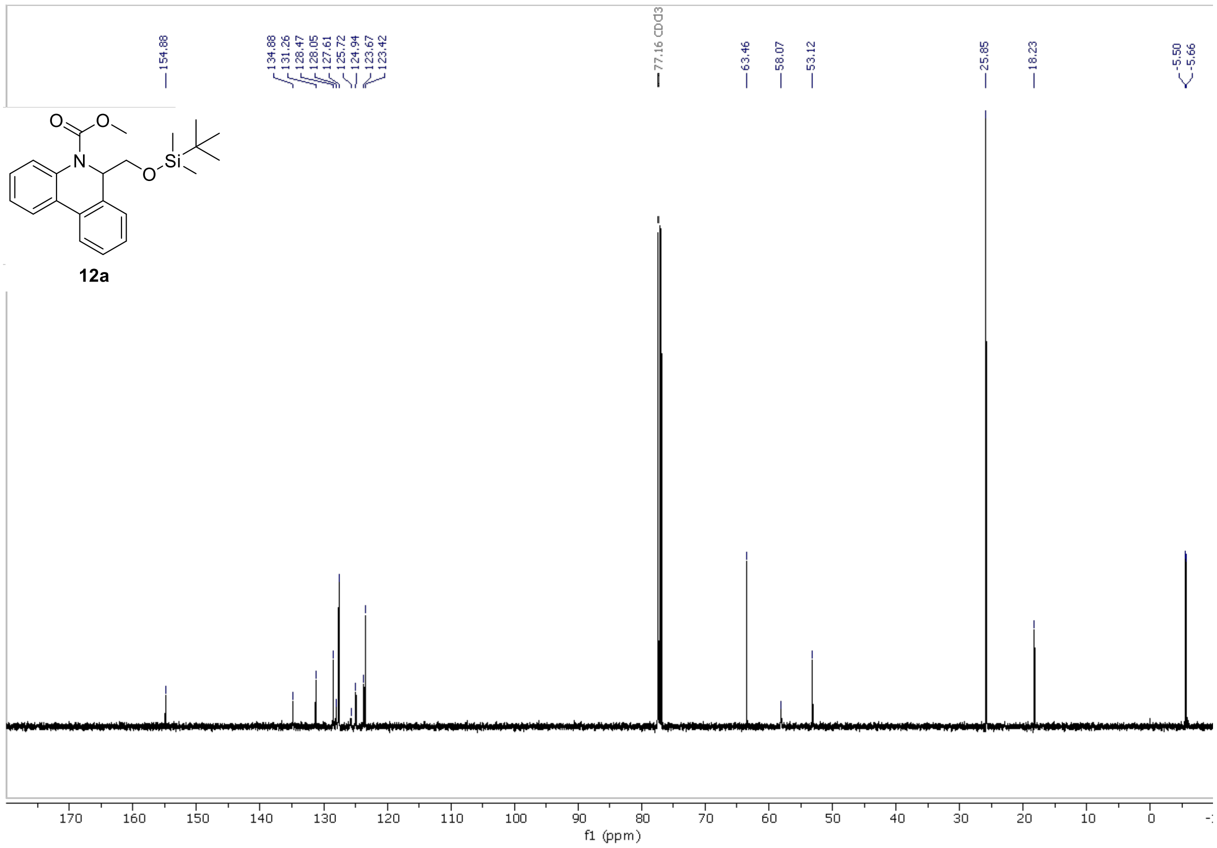

Product obtained using (*S*,*S*)-IBioxtBu:

<Chromatogram>

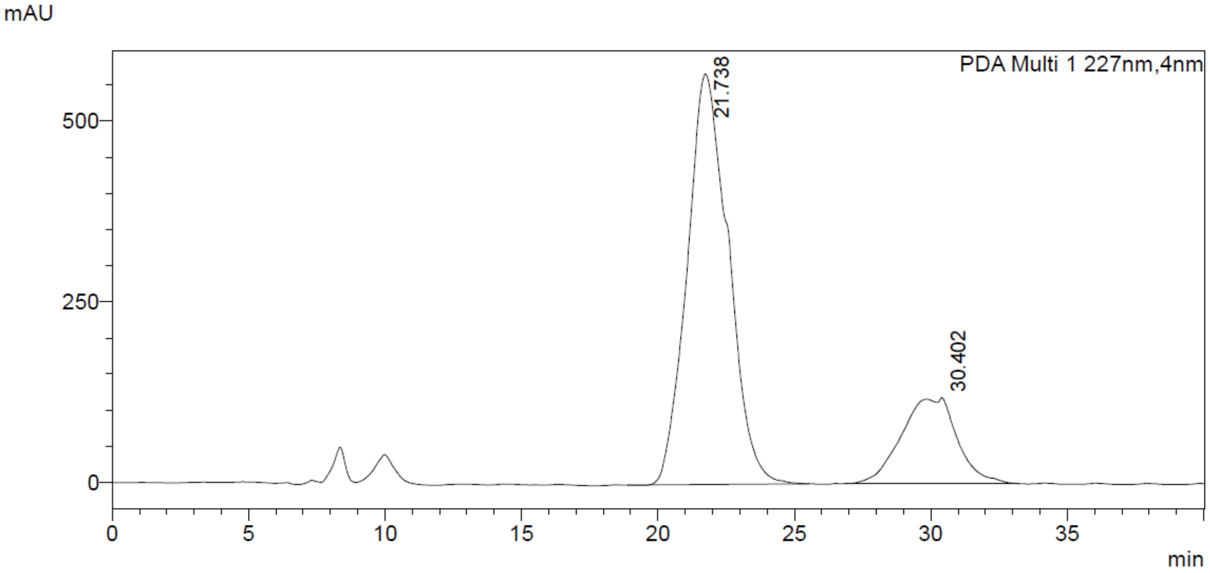

<Peak Table>

PDA Ch1 227nm

Peak#	Ret. Time	Area	Area%
1	21.738	59781836	77.961
2	30.402	16899985	22.039
Total		76681821	100.000

Racemic product obtained using IBioxMe$_4$:

<Chromatogram>

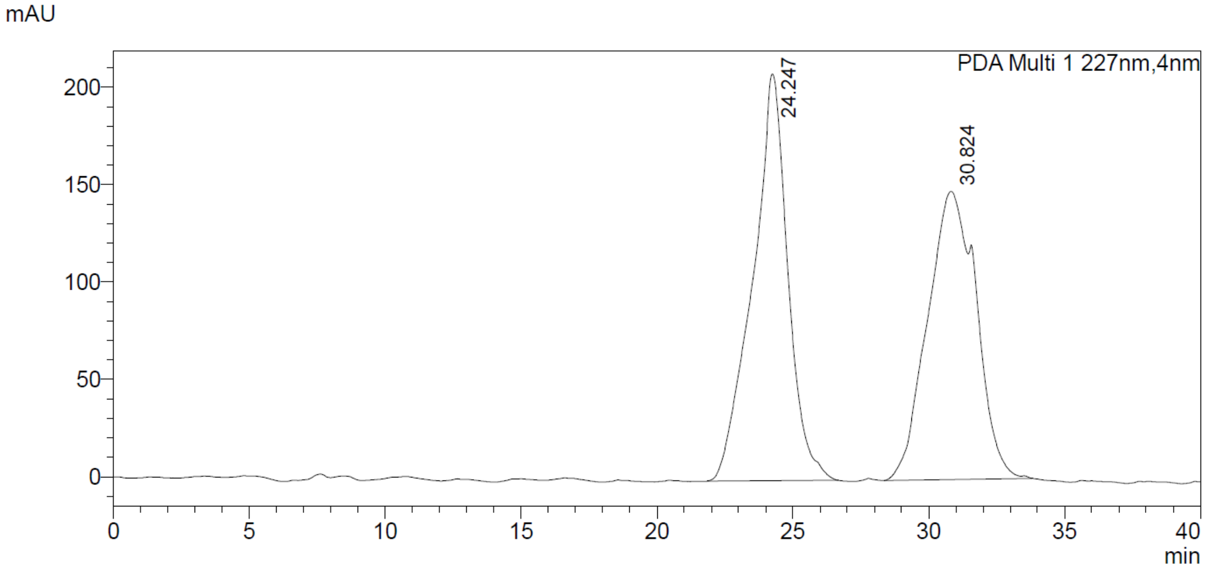

<Peak Table>

PDA Ch1 227nm

Peak#	Ret. Time	Area	Area%
1	24.247	18781771	50.426
2	30.824	18464140	49.574
Total		37245911	100.000

^{1}H NMR (400 MHz, Chloroform-*d*)

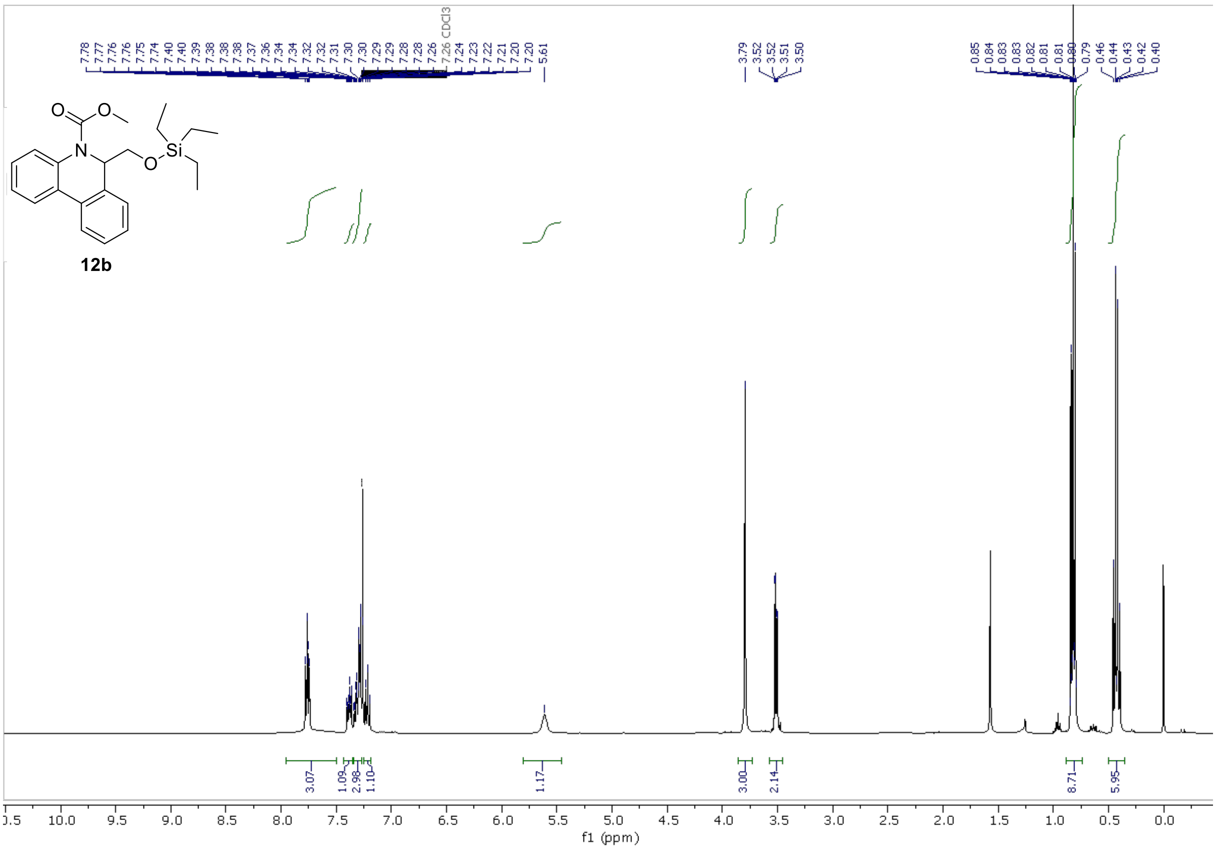

^{13}C{^{1}H} NMR (126 MHz, Chloroform-*d*)

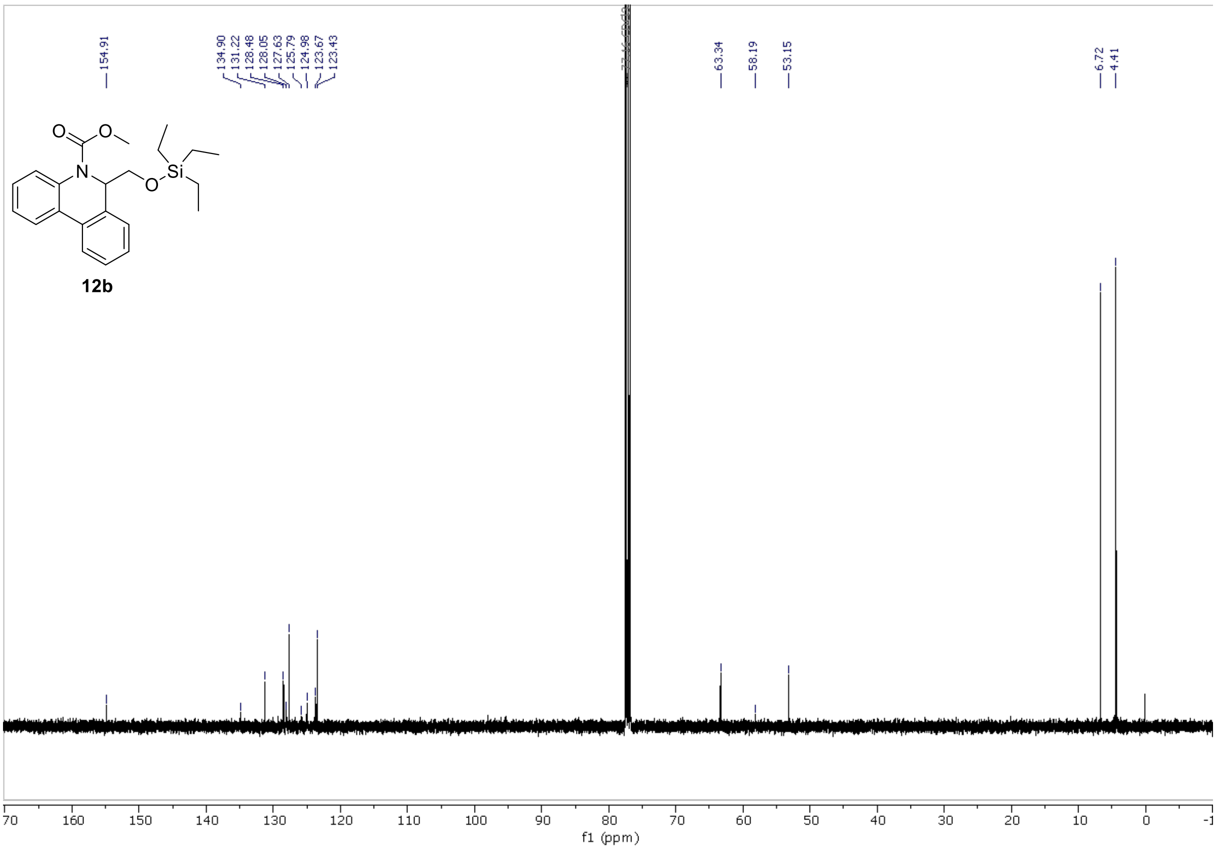

Product obtained using (*S*,*S*)-IBioxtBu:

<Chromatogram>

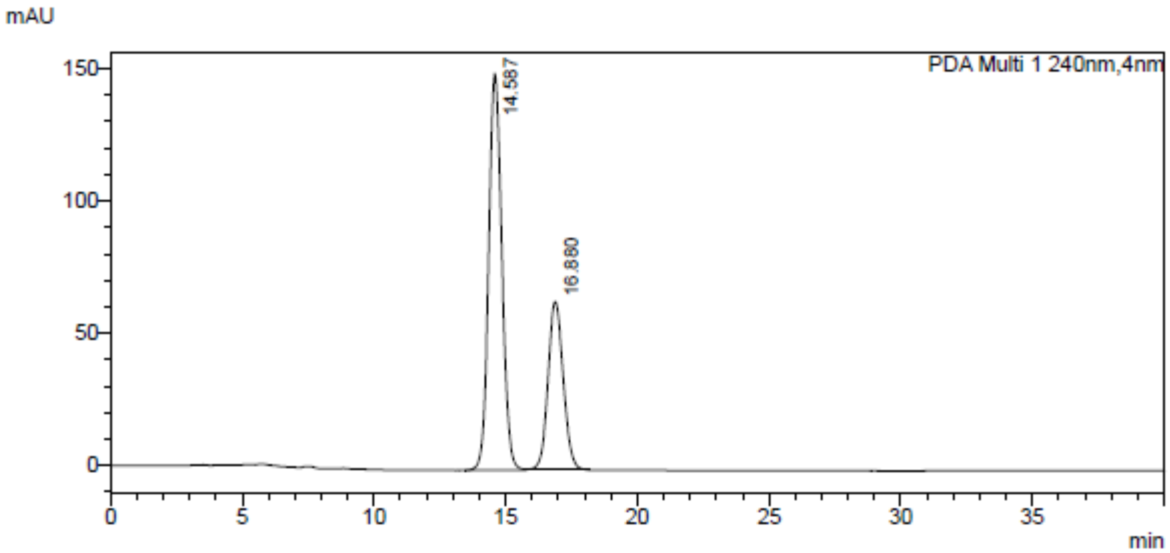

<Peak Table>

PDA Ch1 240nm

Peak#	Ret. Time	Area	Area%
1	14.587	5208190	67.021
2	16.880	2562771	32.979
Total		7770962	100.000

Racemic product obtained using IBioxMe$_4$:

<Chromatogram>

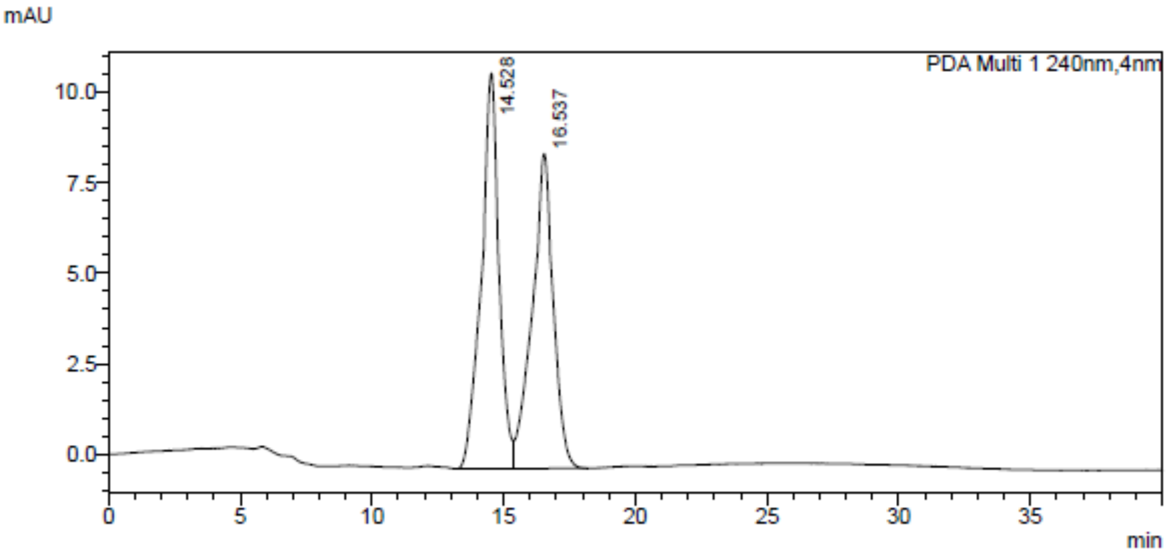

<Peak Table>

PDA Ch1 240nm

Peak#	Ret. Time	Area	Area%
1	14.528	511163	51.368
2	16.537	483930	48.632
Total		995093	100.000

^{1}H NMR (400 MHz, Chloroform-*d*)

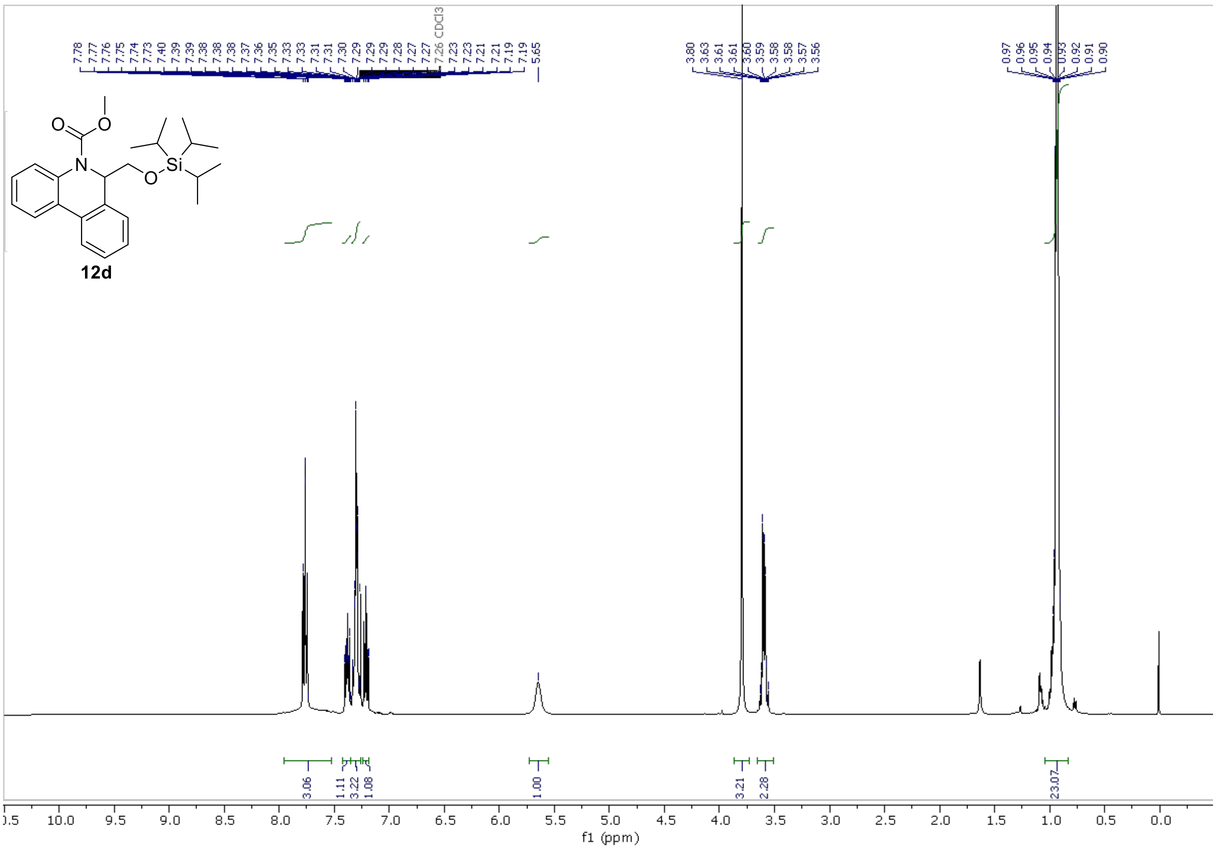

^{13}C{^{1}H} NMR (101 MHz, Chloroform-*d*)

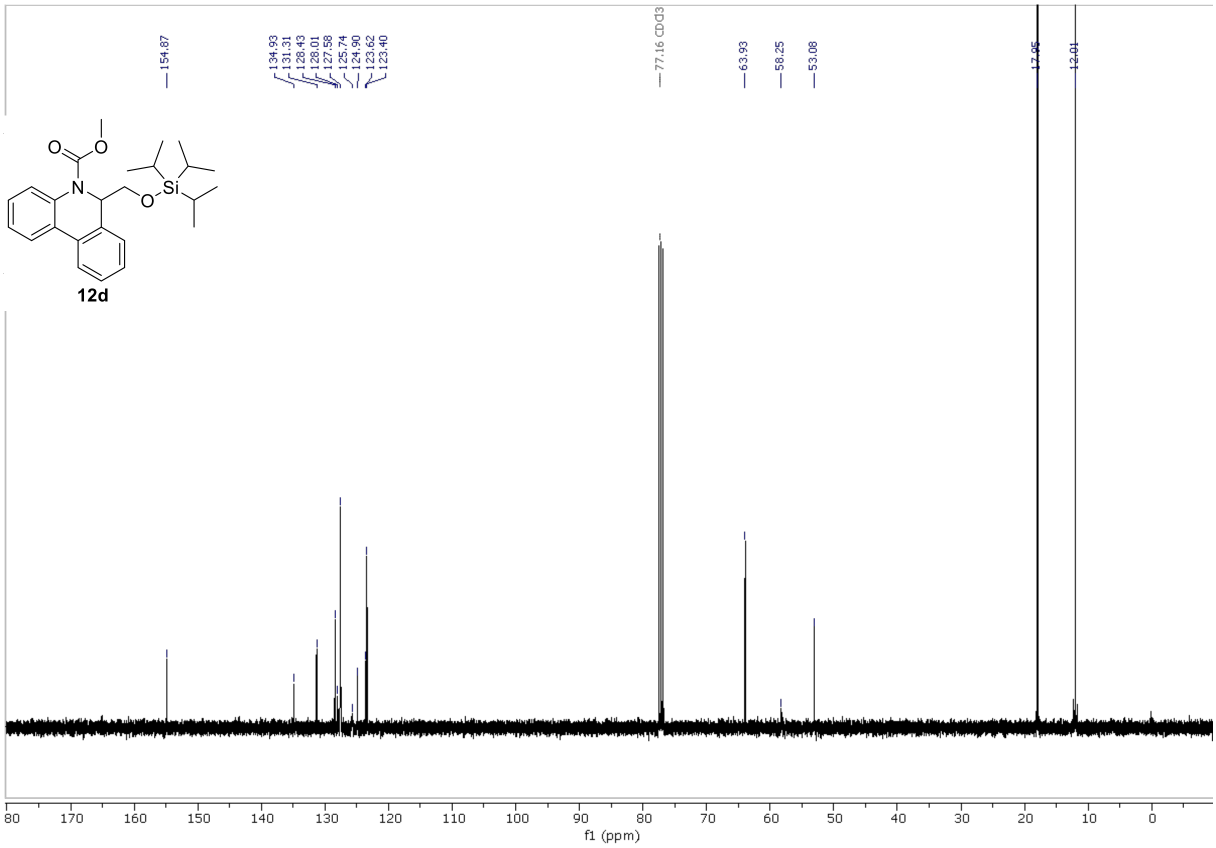

Product obtained using (*S*,*S*)-IBioxtBu:

<Chromatogram>

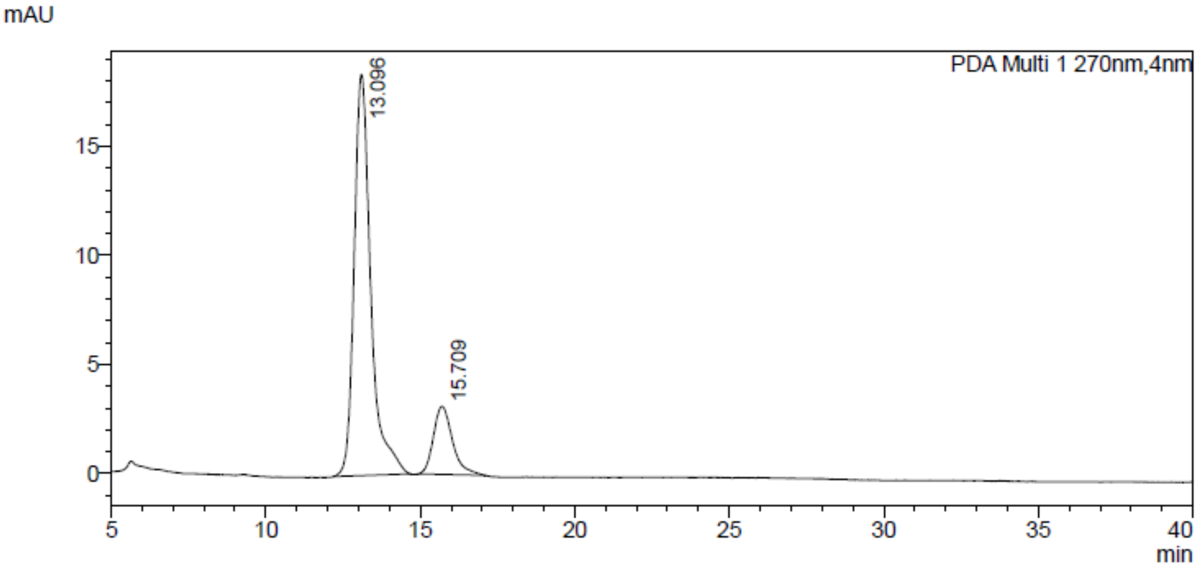

<Peak Table>

PDA Ch1 270nm

Peak#	Ret. Time	Area	Area%
1	13.096	687060	83.641
2	15.709	134379	16.359
Total		821439	100.000

Racemic product obtained using $IBioxMe_4$:

<Chromatogram>

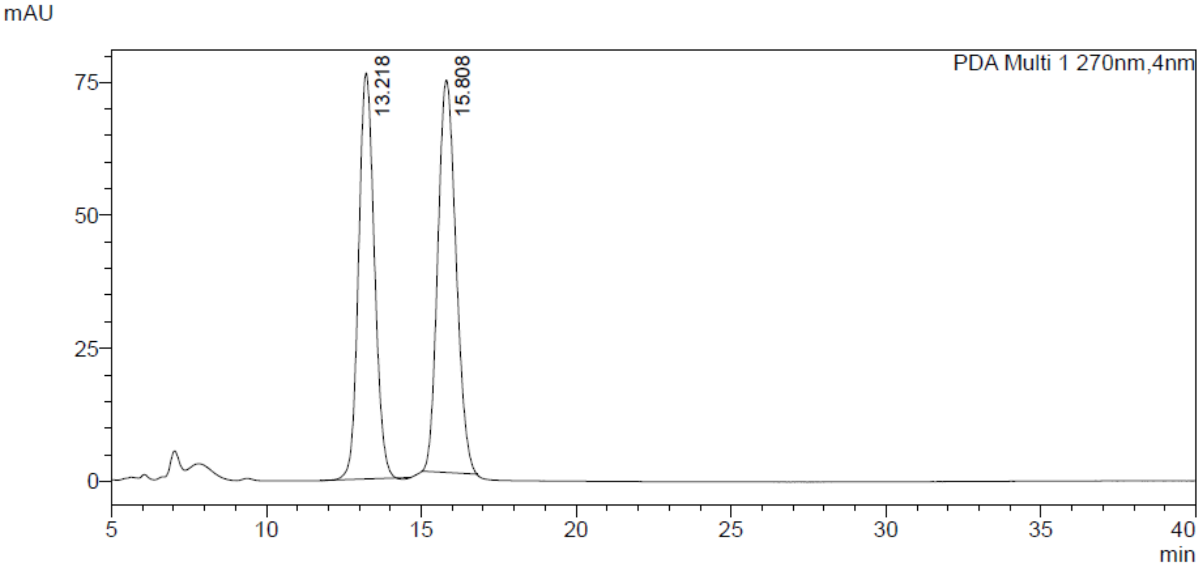

<Peak Table>

PDA Ch1 270nm

Peak#	Ret. Time	Area	Area%
1	13.218	2707157	47.343
2	15.808	3011047	52.657
Total		5718204	100.000

^{1}H NMR (400 MHz, Chloroform-*d*)

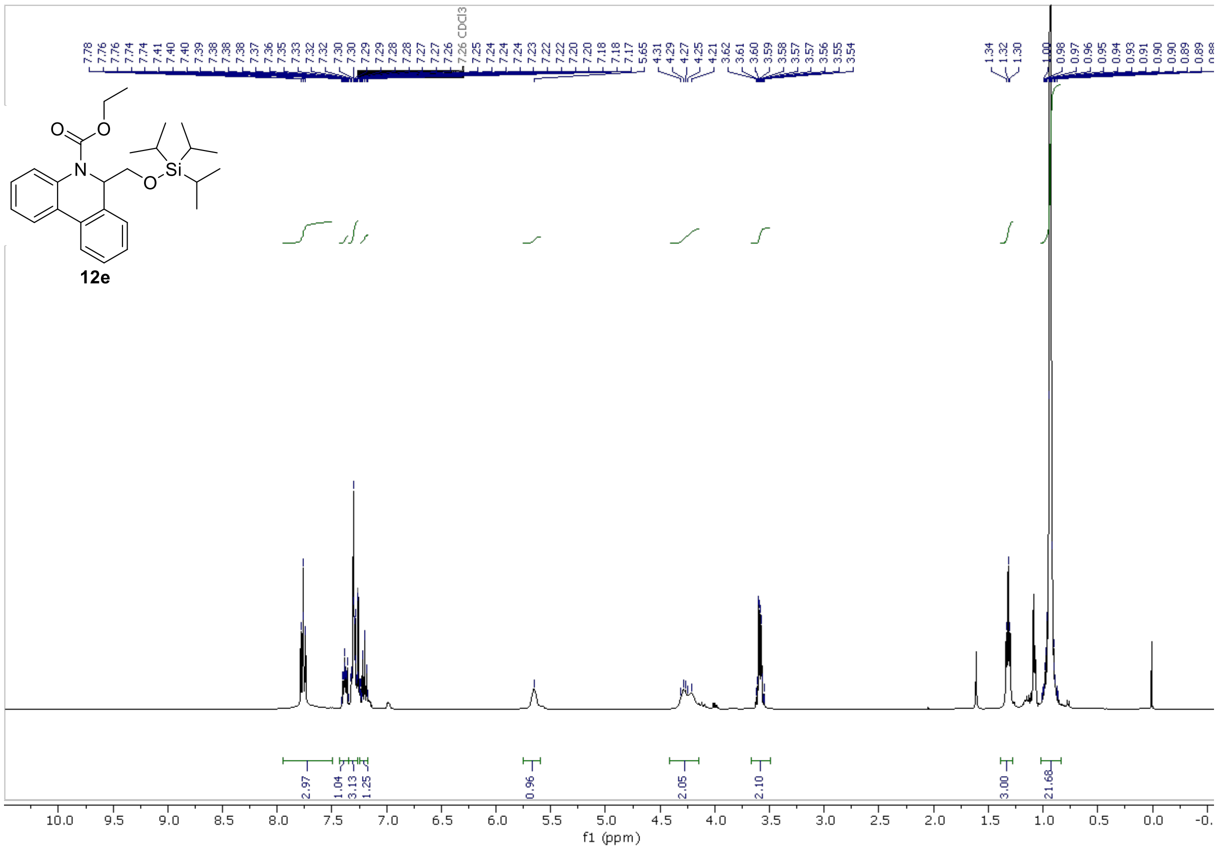

^{13}C{^{1}H} NMR (101 MHz, Chloroform-*d*)

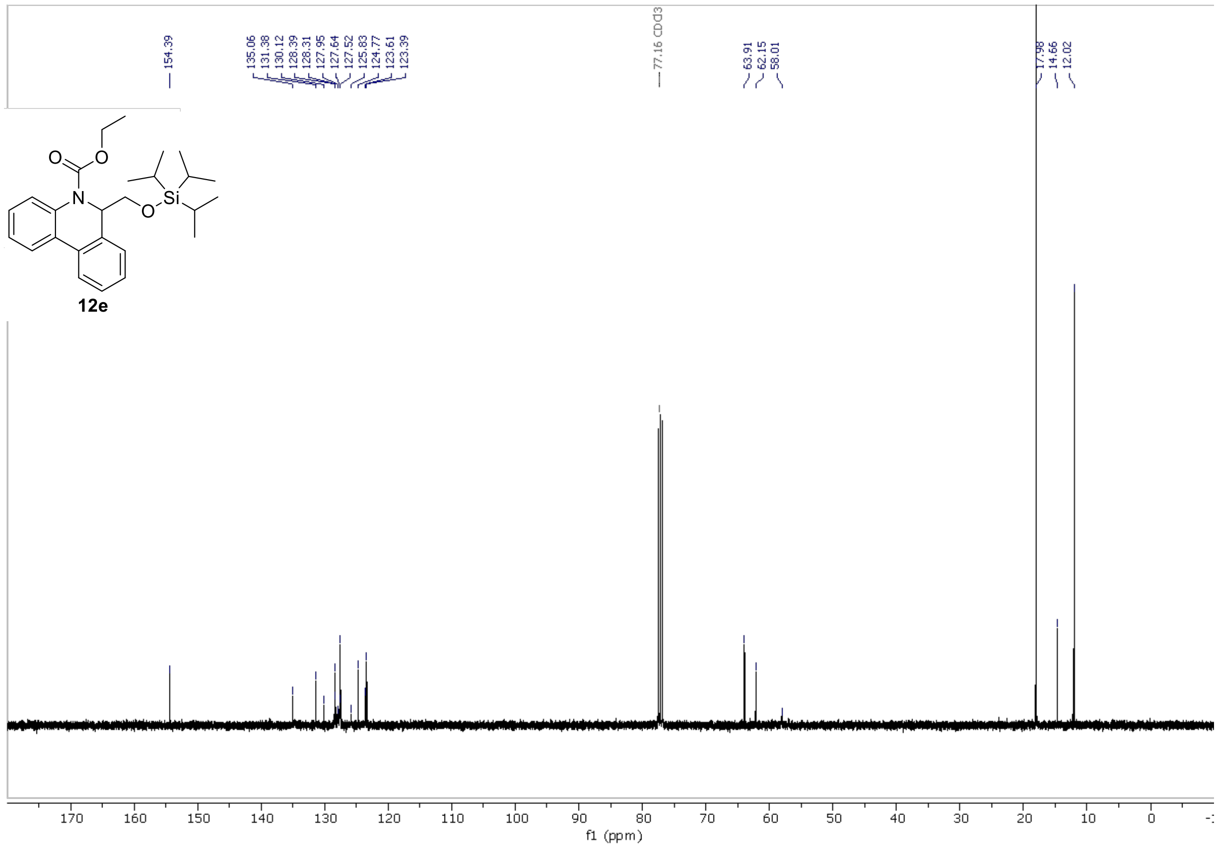

Product obtained using (*S*,*S*)-IBioxtBu:

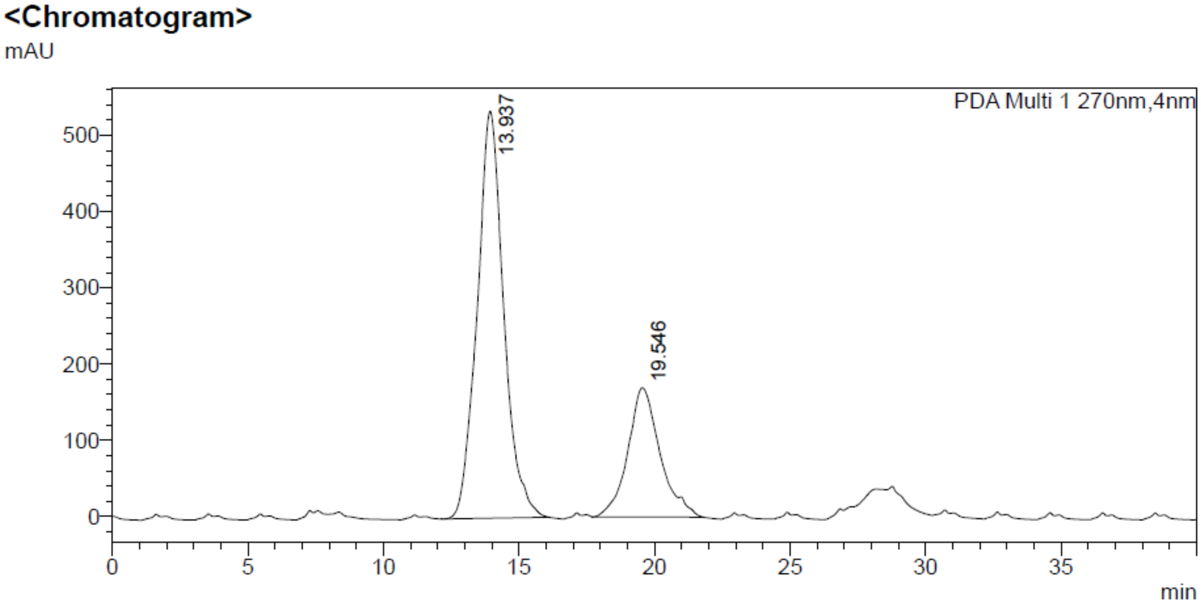

<Peak Table>

PDA Ch1 270nm

Peak#	Ret. Time	Area	Area%
1	13.937	36416725	72.147
2	19.546	14059347	27.853
Total		50476072	100.000

Racemic product obtained using IBioxMe$_4$:

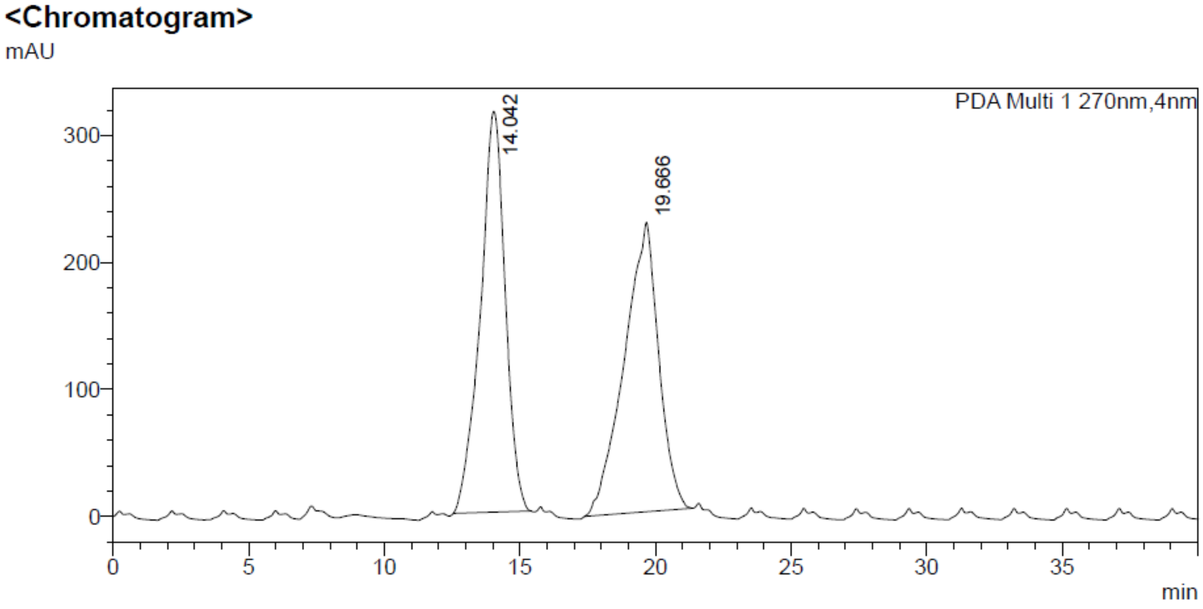

<Peak Table>

PDA Ch1 270nm

Peak#	Ret. Time	Area	Area%
1	14.042	20182017	50.030
2	19.666	20157928	49.970
Total		40339946	100.000

^{1}H NMR (400 MHz, Chloroform-*d*)

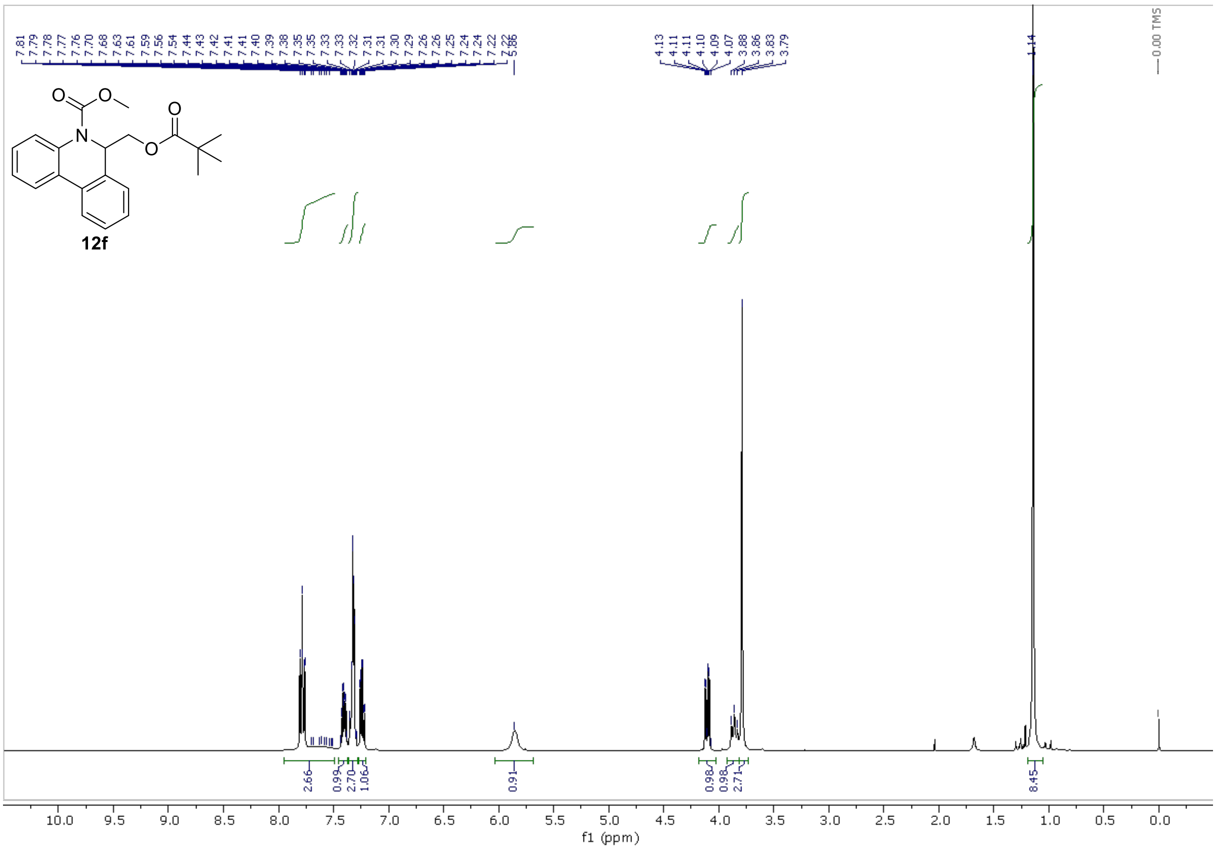

^{13}C{^{1}H} NMR (101 MHz, Chloroform-*d*)

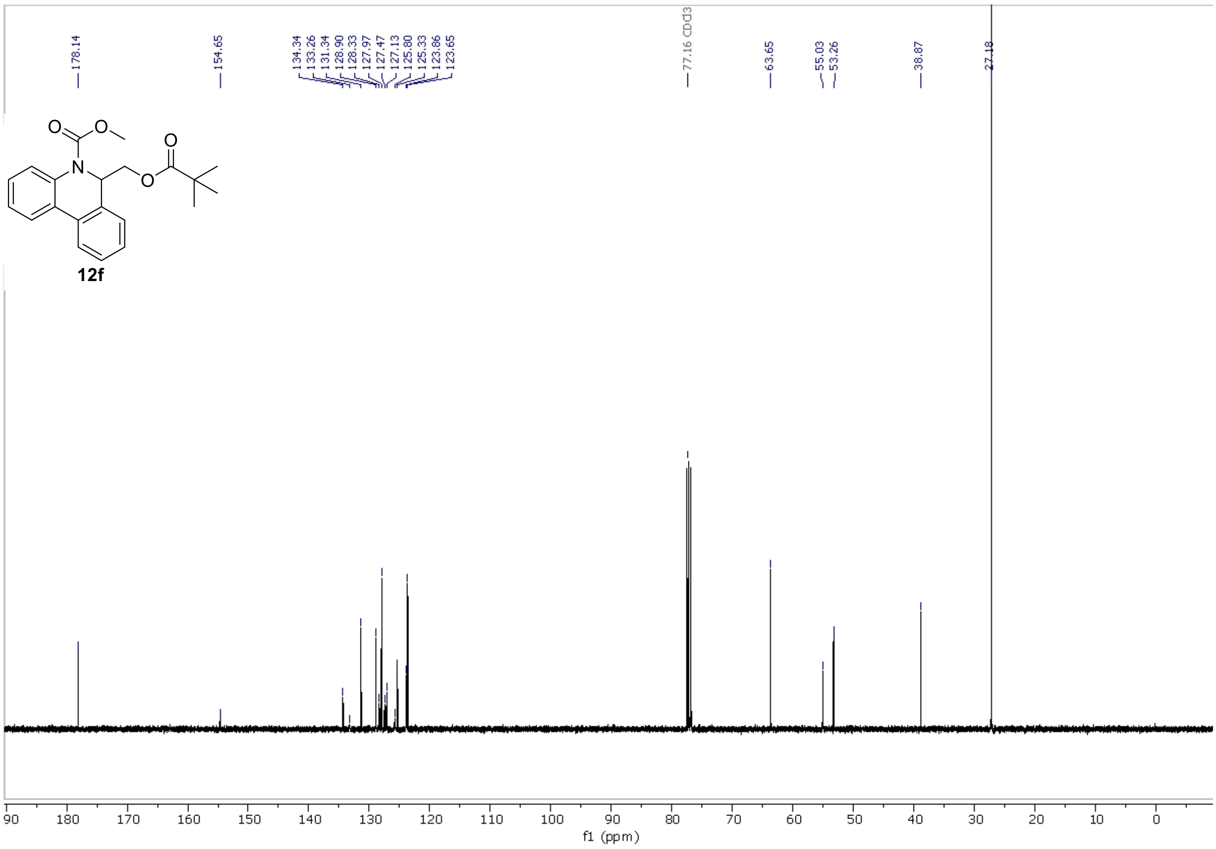

Product obtained using (*S*,*S*)-IBioxtBu:

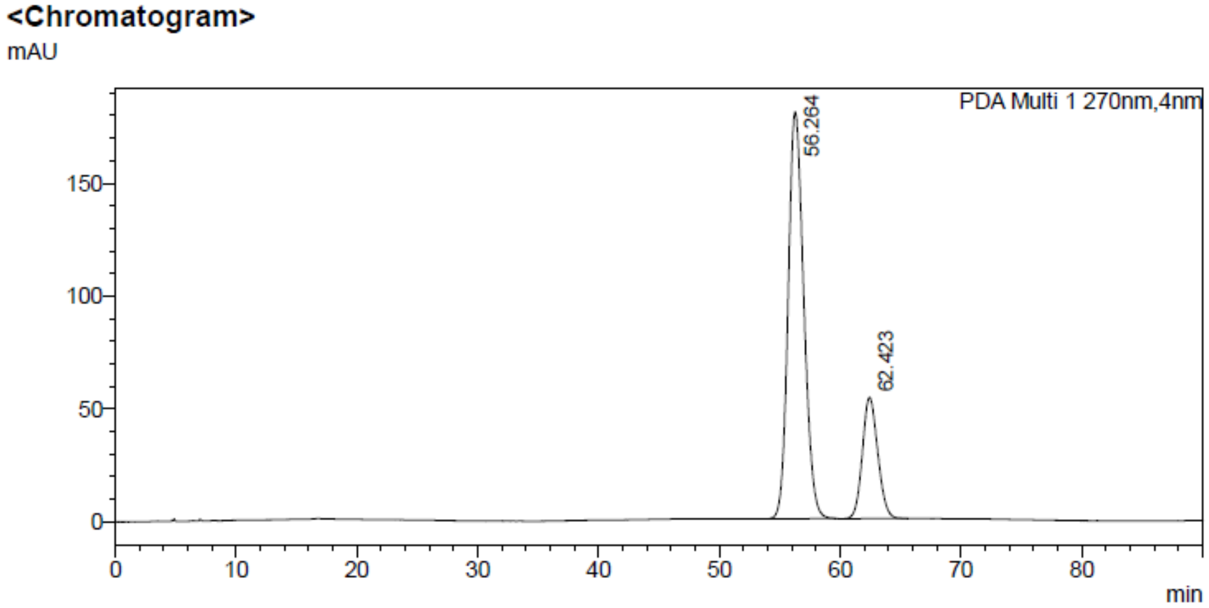

<Peak Table>

PDA Ch1 270nm

Peak#	Ret. Time	Area	Area%
1	56.264	16178273	77.273
2	62.423	4758351	22.727
Total		20936624	100.000

Racemic product obtained using IBioxMe$_4$:

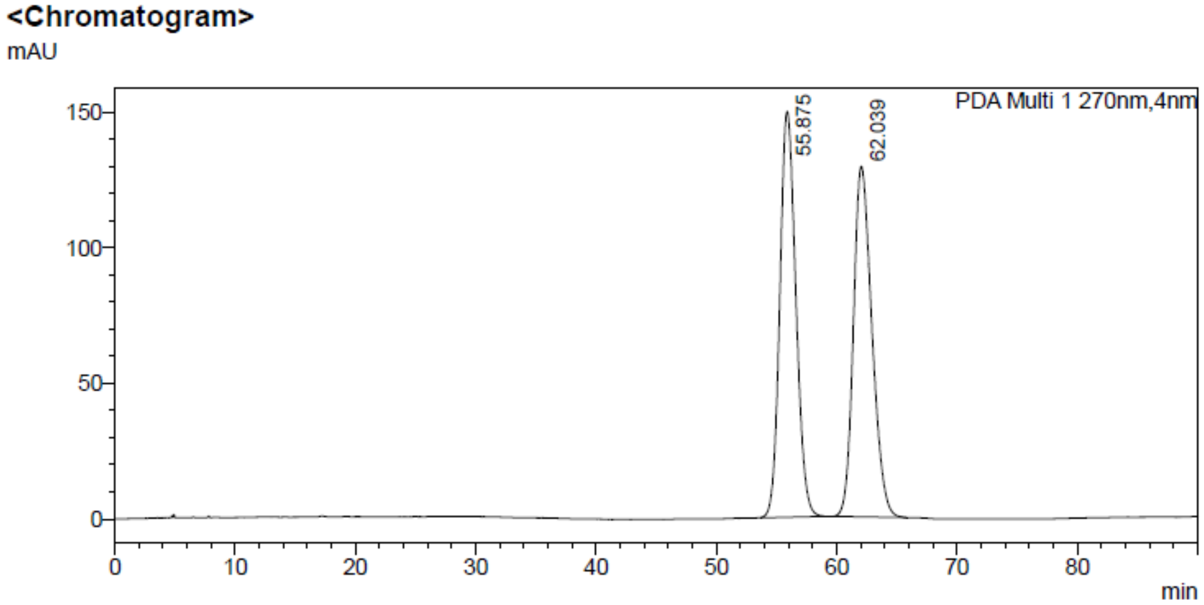

<Peak Table>

PDA Ch1 270nm

Peak#	Ret. Time	Area	Area%
1	55.875	13846718	50.038
2	62.039	13825594	49.962
Total		27672312	100.000

^{1}**H NMR** (400 MHz, Chloroform-*d*)

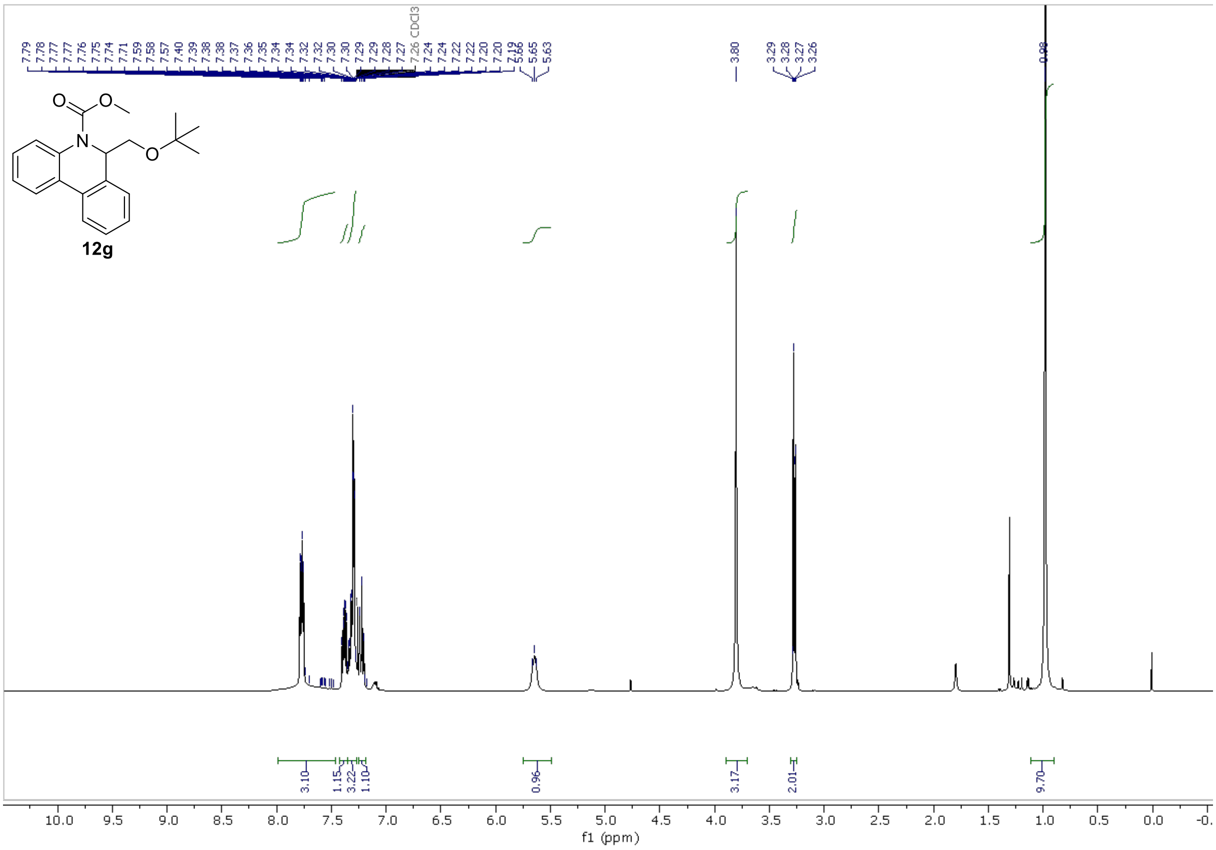

^{13}**C{^{1}H} NMR** (101 MHz, Chloroform-*d*)

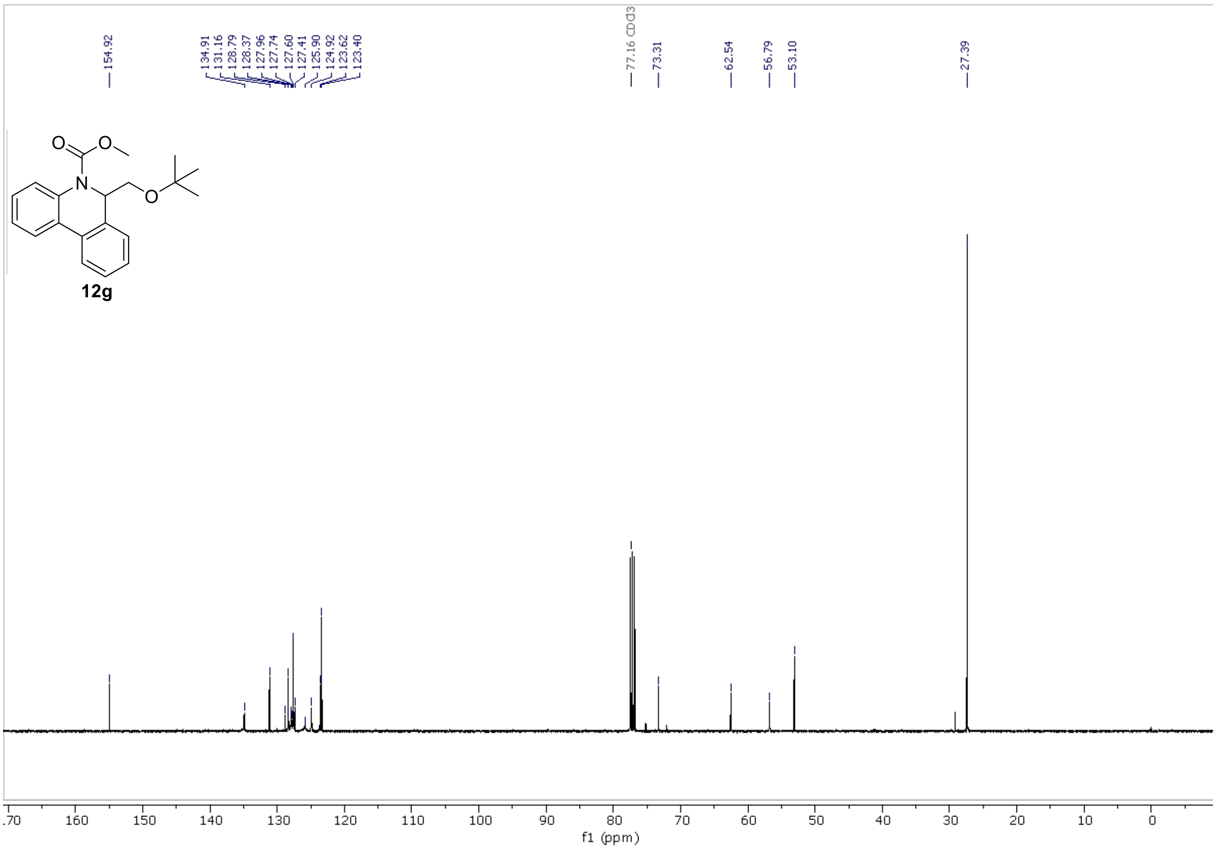

Product obtained using (*S*,*S*)-IBioxtBu:

<Chromatogram>

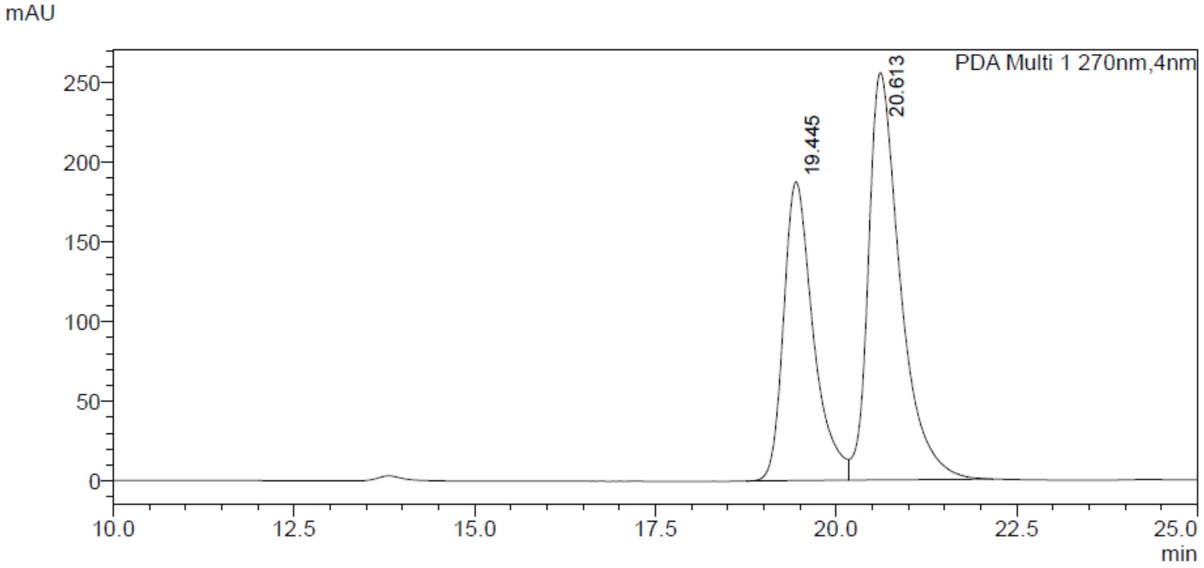

<Peak Table>

PDA Ch1 270nm

Peak#	Ret. Time	Area	Area%
1	19.445	5359271	40.606
2	20.613	7838992	59.394
Total		13198263	100.000

Racemic product obtained using $IBioxMe_4$:

<Chromatogram>

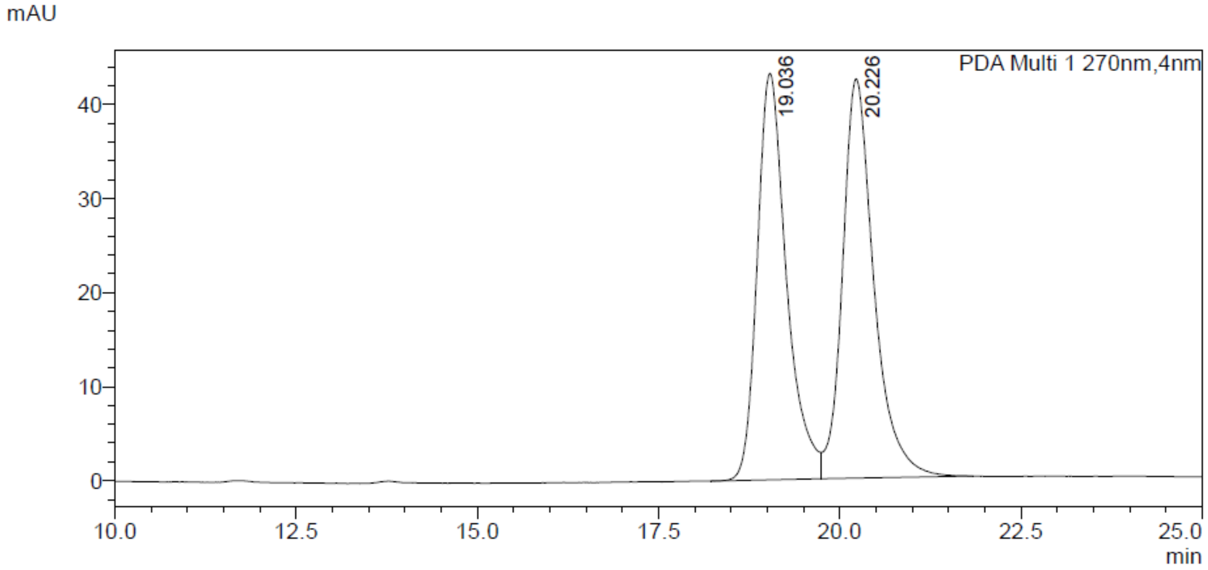

<Peak Table>

PDA Ch1 270nm

Peak#	Ret. Time	Area	Area%
1	19.036	1217764	48.728
2	20.226	1281322	51.272
Total		2499086	100.000

^{1}H NMR (400 MHz, Chloroform-*d*)

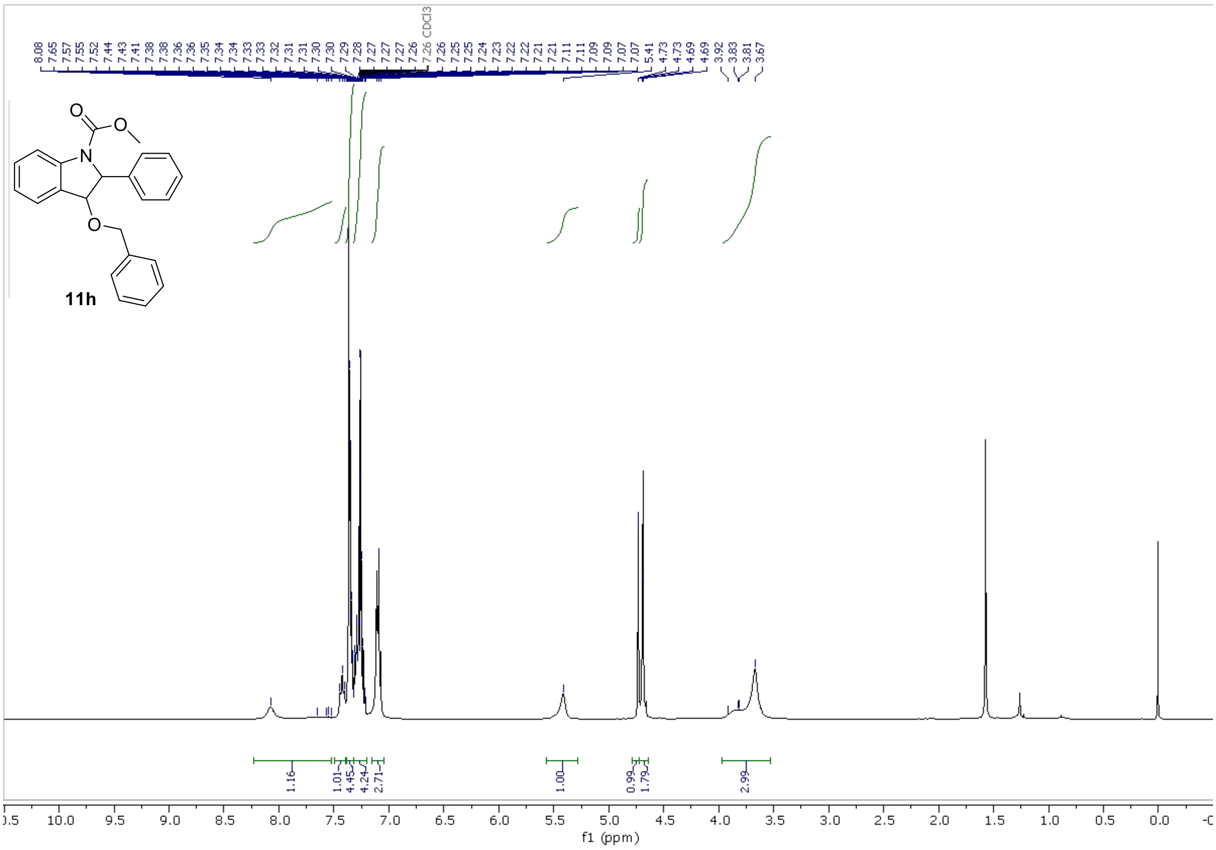

^{13}C{^{1}H} NMR (101 MHz, Chloroform-*d*)

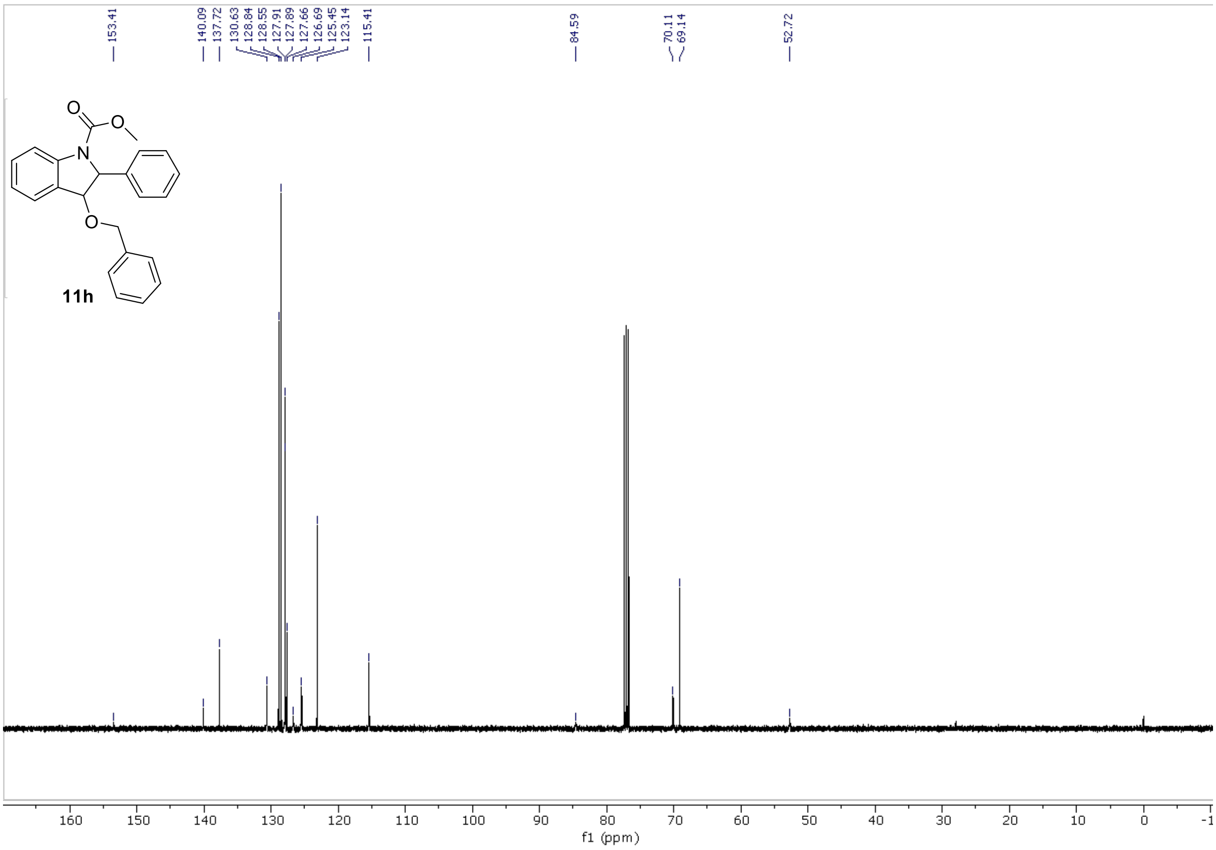

Product obtained using (*S*,*S*)-IBioxAd:

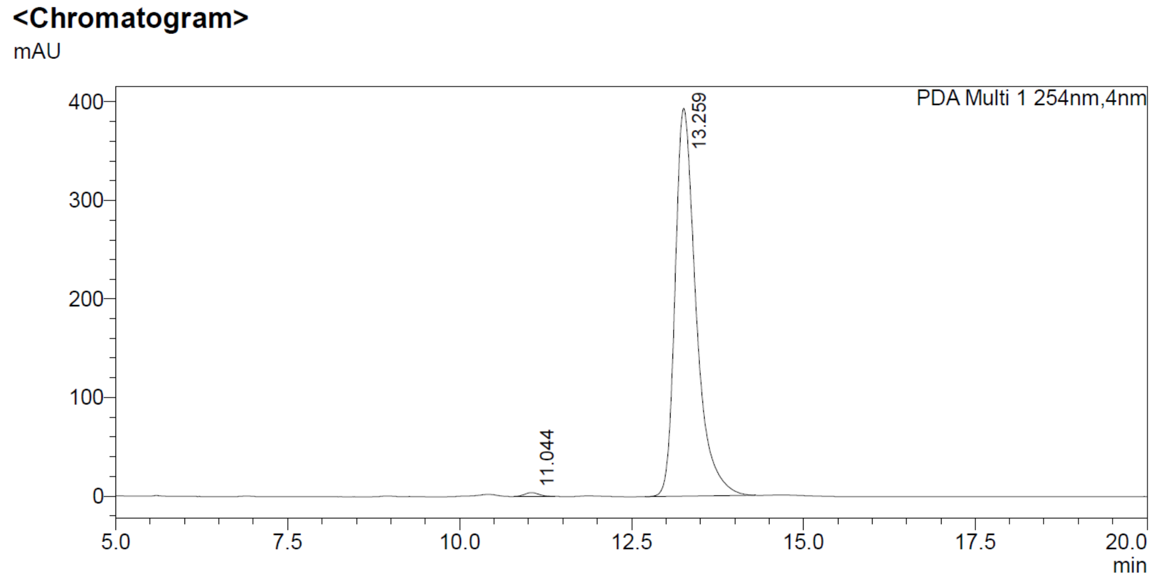

<Peak Table>

PDA Ch1 254nm

Peak#	Ret. Time	Area	Area%
1	11.044	56995	0.678
2	13.259	8354392	99.322
Total		8411386	100.000

Product obtained using (*S*,*S*)-L[1]:

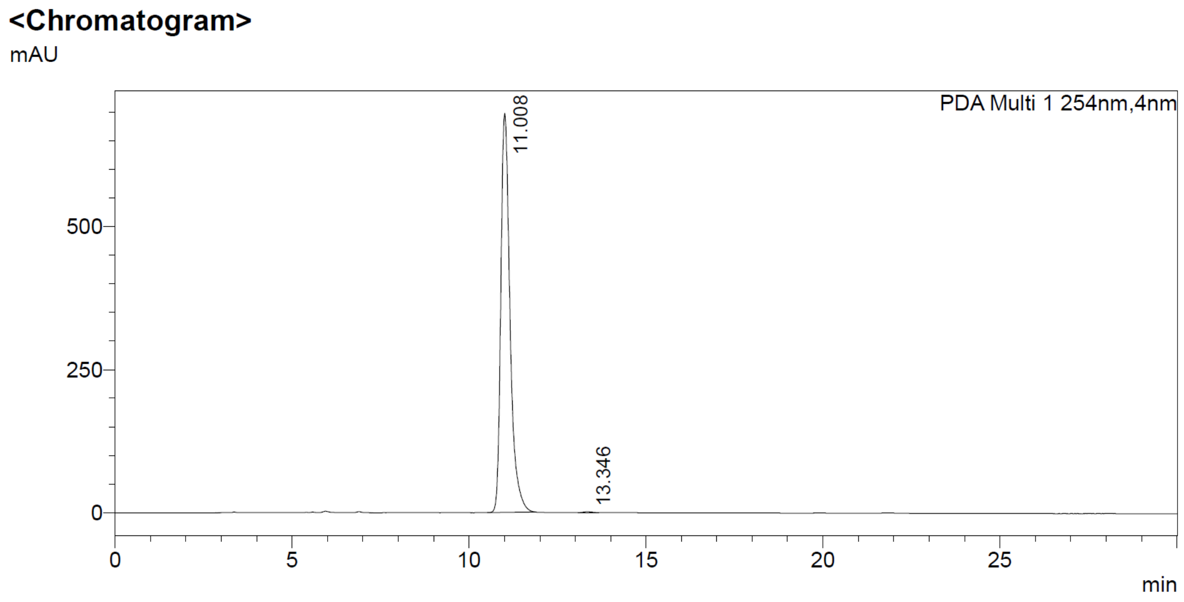

<Peak Table>

PDA Ch1 254nm

Peak#	Ret. Time	Area	Area%
1	11.008	12612330	99.814
2	13.346	23501	0.186
Total		12635831	100.000

Product obtained using (R,R)-**L**[2]:

<Chromatogram>

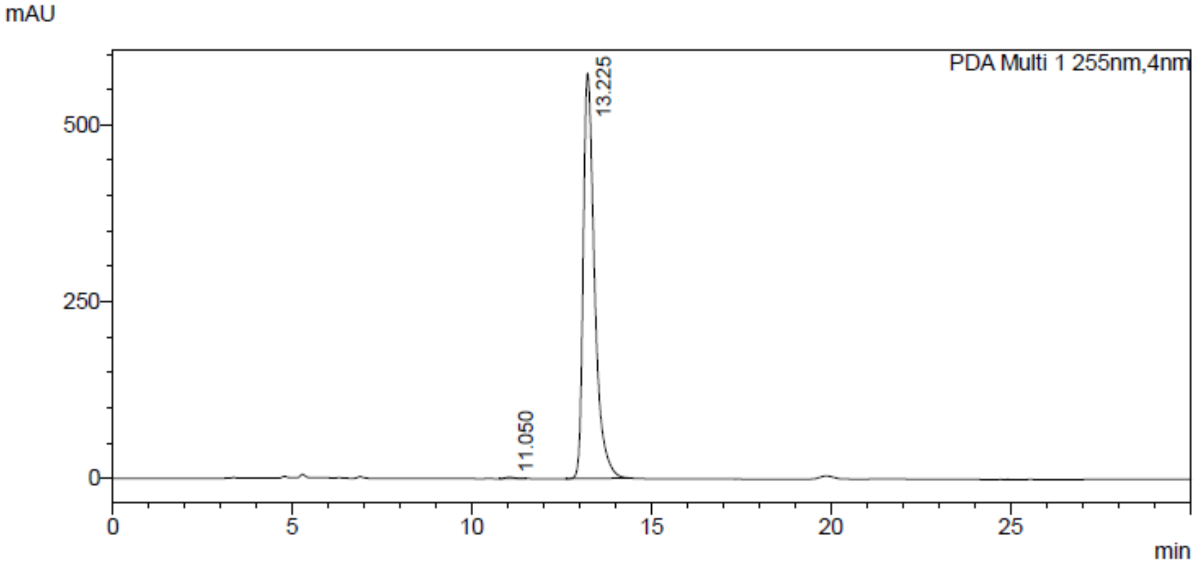

<Peak Table>

PDA Ch1 255nm

Peak#	Ret. Time	Area	Area%
1	11.050	30457	0.240
2	13.225	12635979	99.760
Total		12666436	100.000

Racemic product obtained using $IBioxMe_4$:

<Chromatogram>

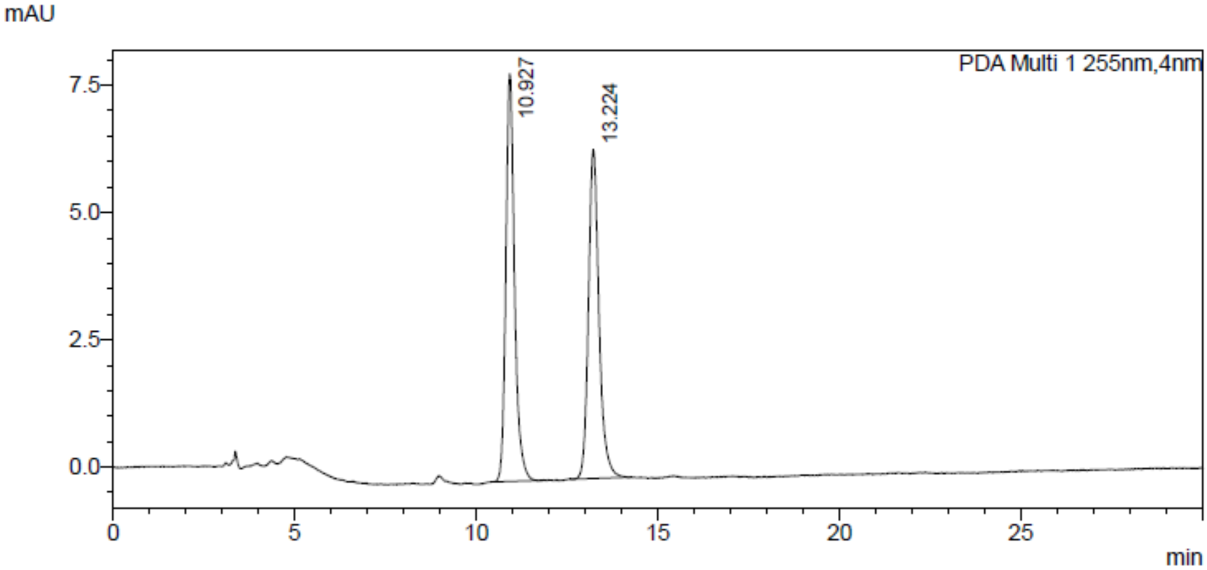

<Peak Table>

PDA Ch1 255nm

Peak#	Ret. Time	Area	Area%
1	10.927	133349	50.531
2	13.224	130544	49.469
Total		263893	100.000

^{1}**H NMR** (400 MHz, Chloroform-*d*)

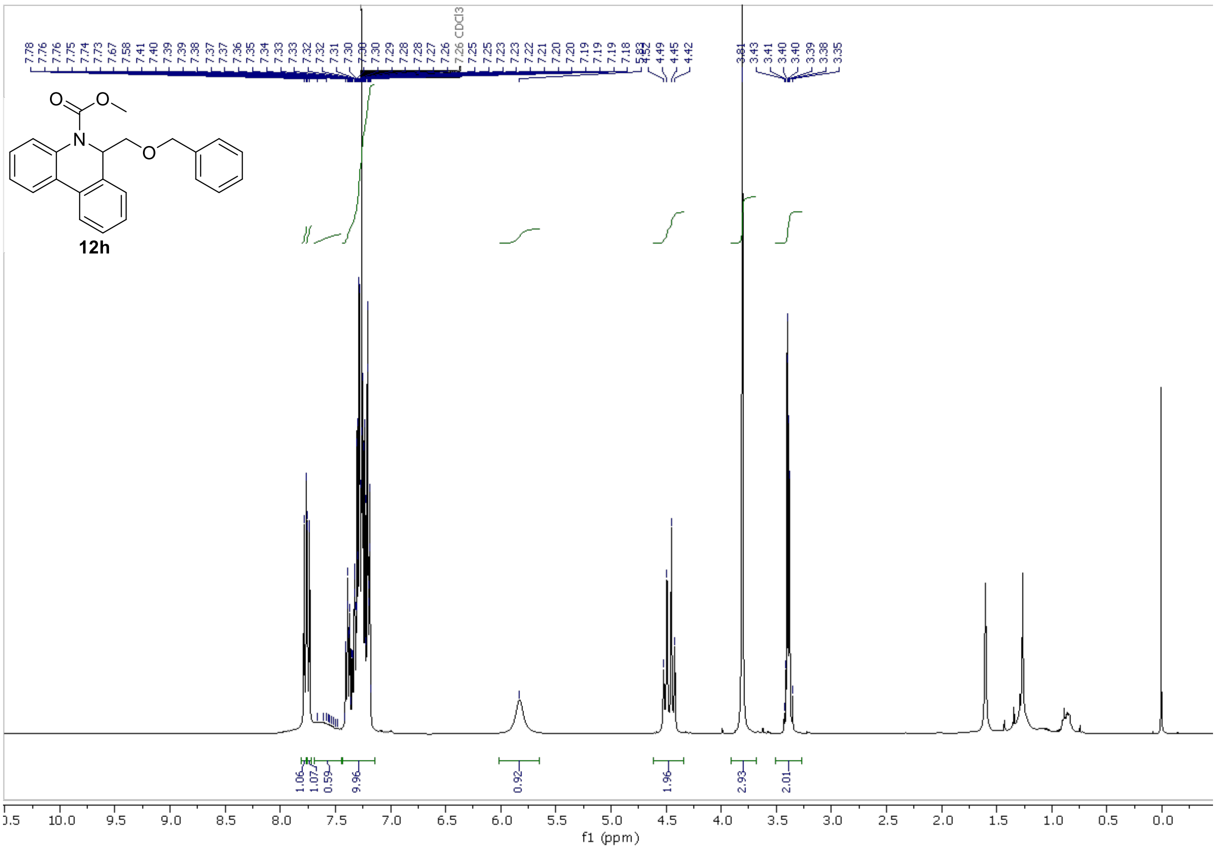

^{13}**C{^{1}H} NMR** (126 MHz, Chloroform-*d*)

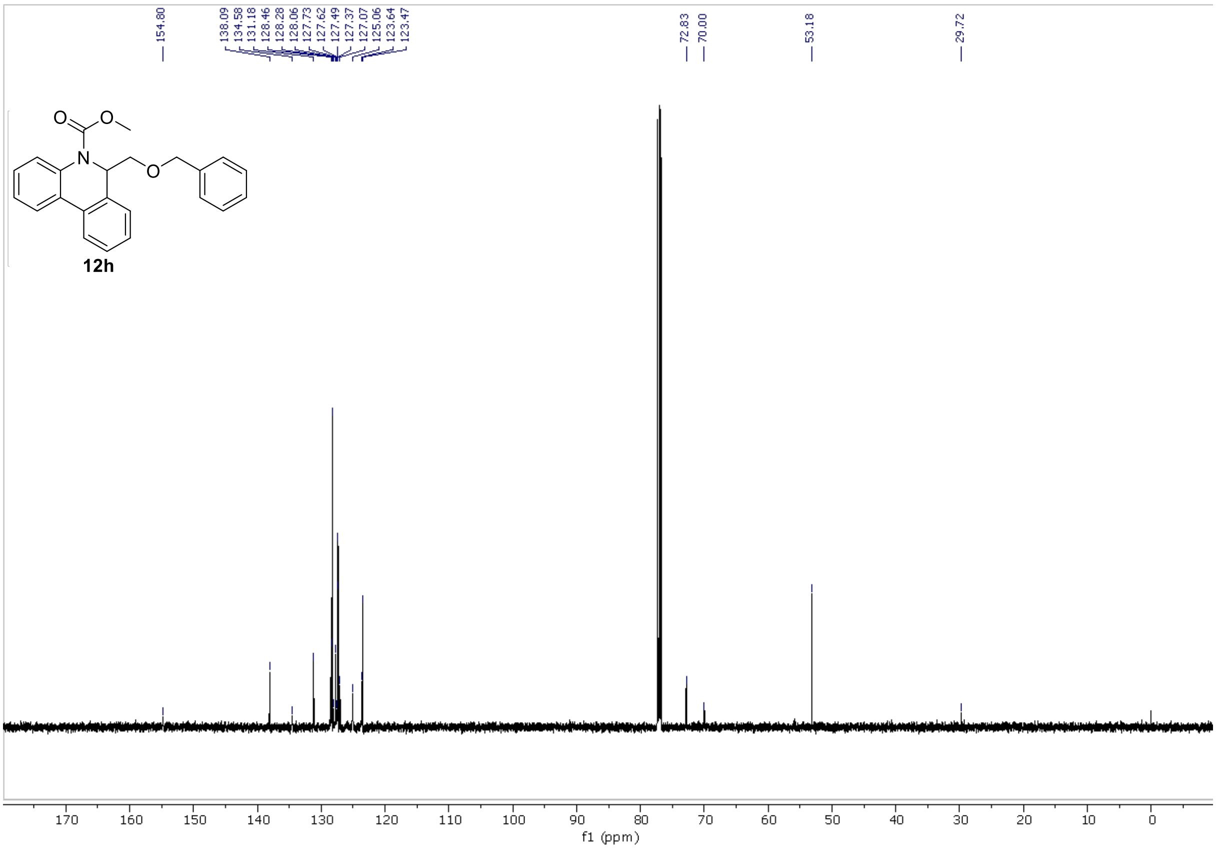

Product obtained using (R,R)-L^2:

<Chromatogram>

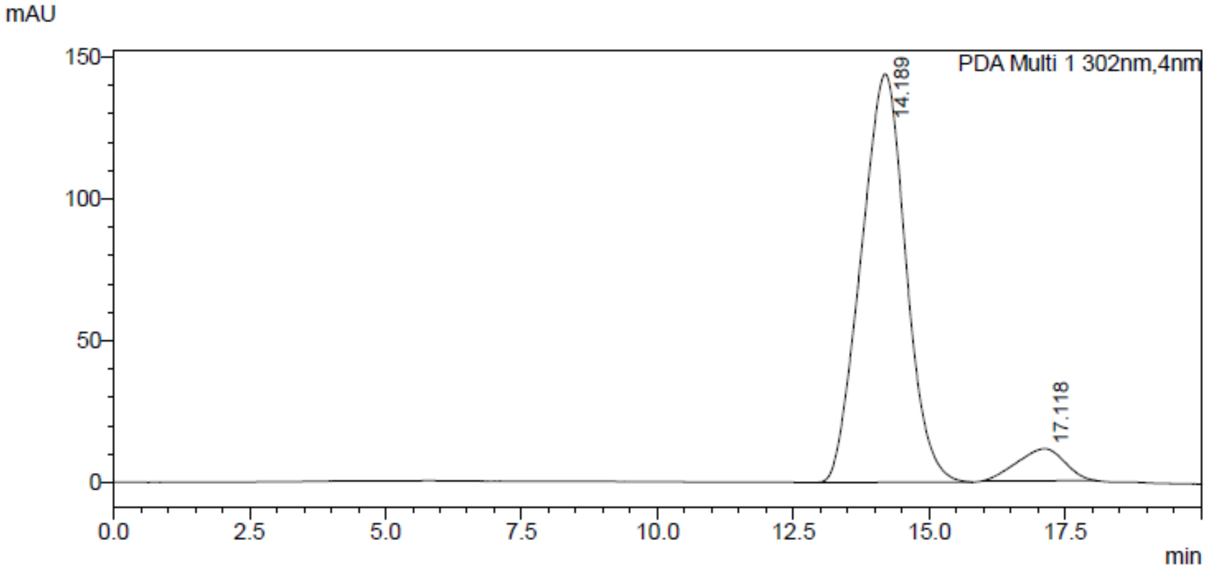

<Peak Table>

PDA Ch1 302nm

Peak#	Ret. Time	Area	Area%
1	14.189	8238696	92.254
2	17.118	691752	7.746
Total		8930448	100.000

Racemic product obtained using $IBioxMe_4$:

<Chromatogram>

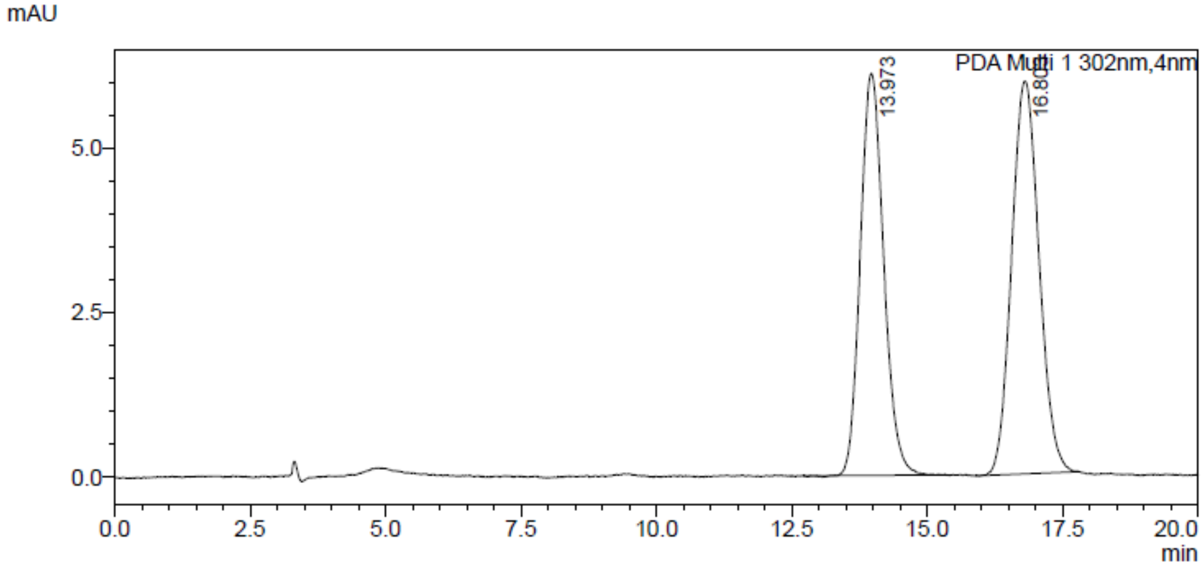

<Peak Table>

PDA Ch1 302nm

Peak#	Ret. Time	Area	Area%
1	13.973	183489	46.586
2	16.803	210384	53.414
Total		393873	100.000

^{1}H NMR (400 MHz, Chloroform-*d*)

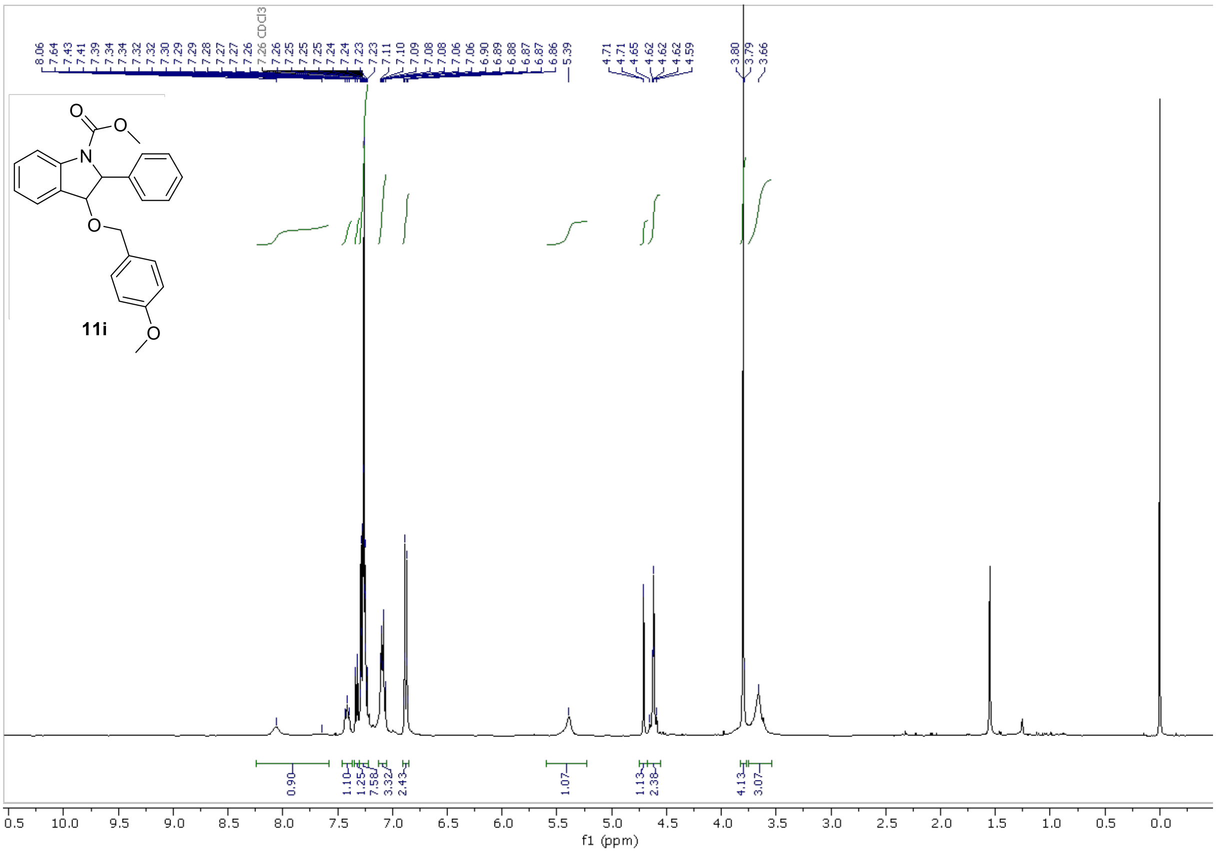

^{13}C{^{1}H} NMR (126 MHz, Chloroform-*d*)

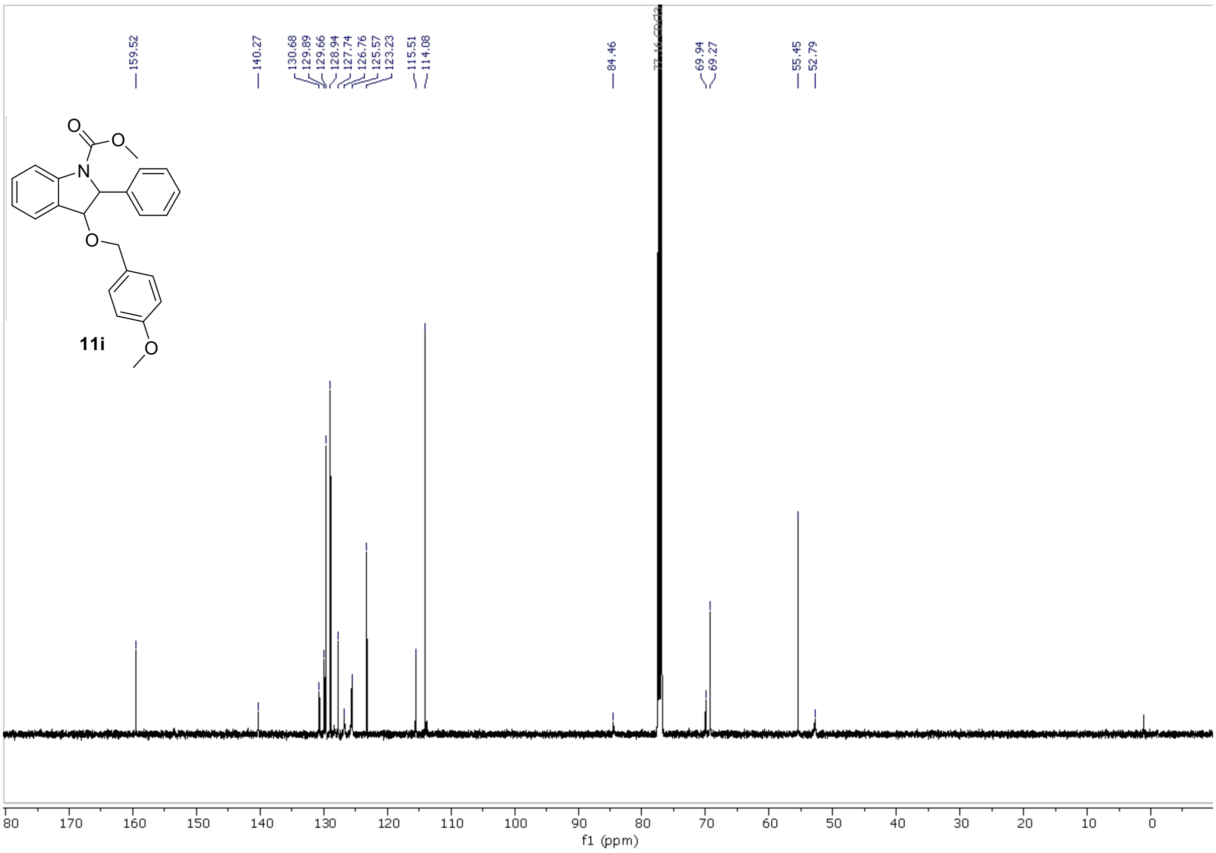

Product obtained using (*S*,*S*)-IBioxtBu:

<Chromatogram>

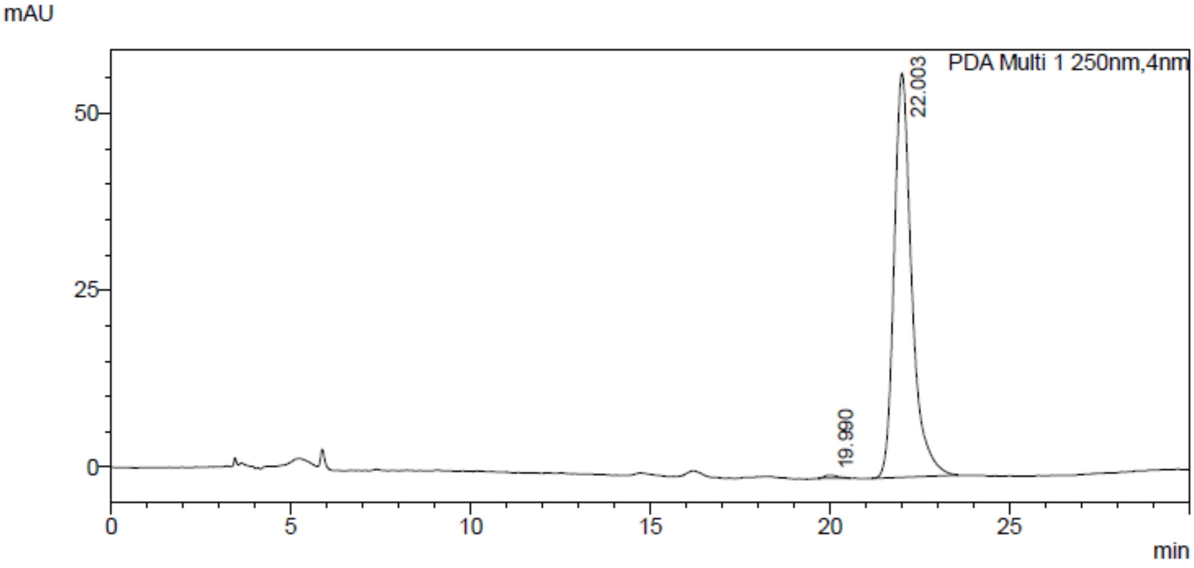

<Peak Table>

PDA Ch1 250nm

Peak#	Ret. Time	Area	Area%
1	19.990	11819	0.604
2	22.003	1944134	99.396
Total		1955953	100.000

Racemic product obtained using $IBioxMe_4$:

<Chromatogram>

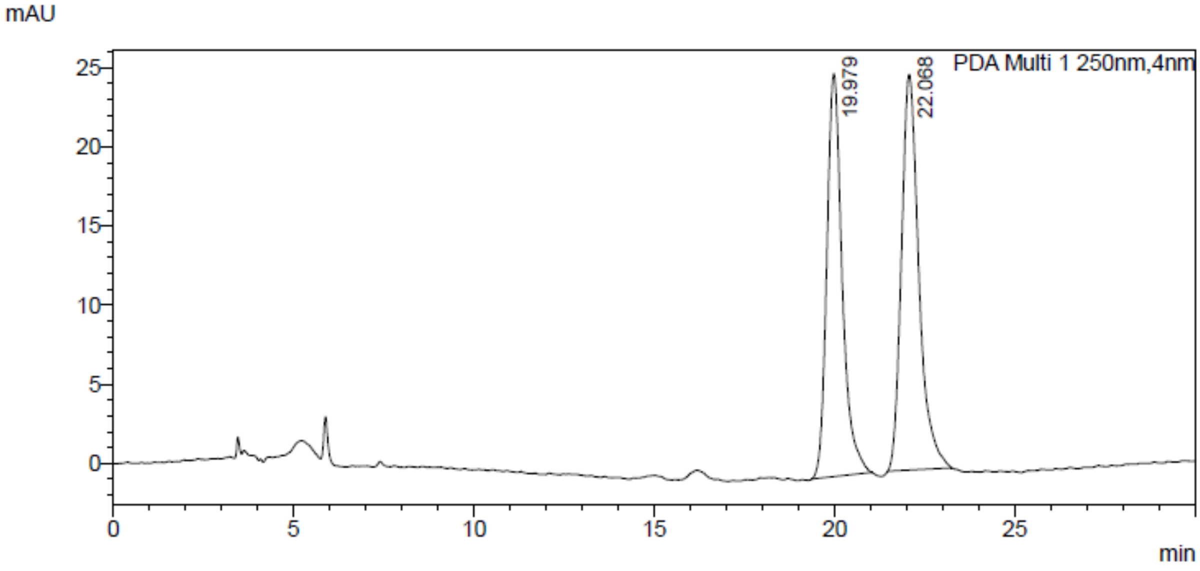

<Peak Table>

PDA Ch1 250nm

Peak#	Ret. Time	Area	Area%
1	19.979	754953	47.351
2	22.068	839425	52.649
Total		1594378	100.000

^{1}H NMR (400 MHz, Chloroform-*d*)

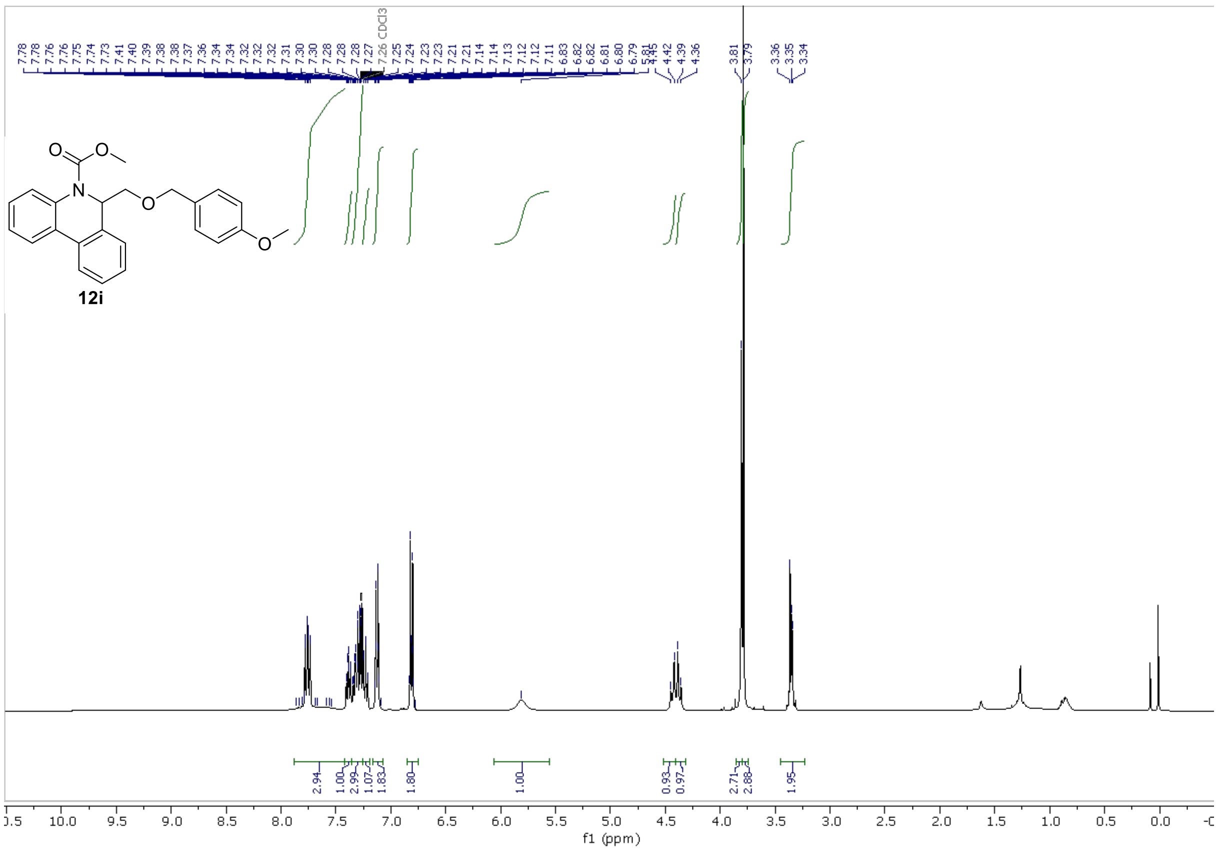

^{13}C{^{1}H} NMR (101 MHz, Chloroform-*d*)

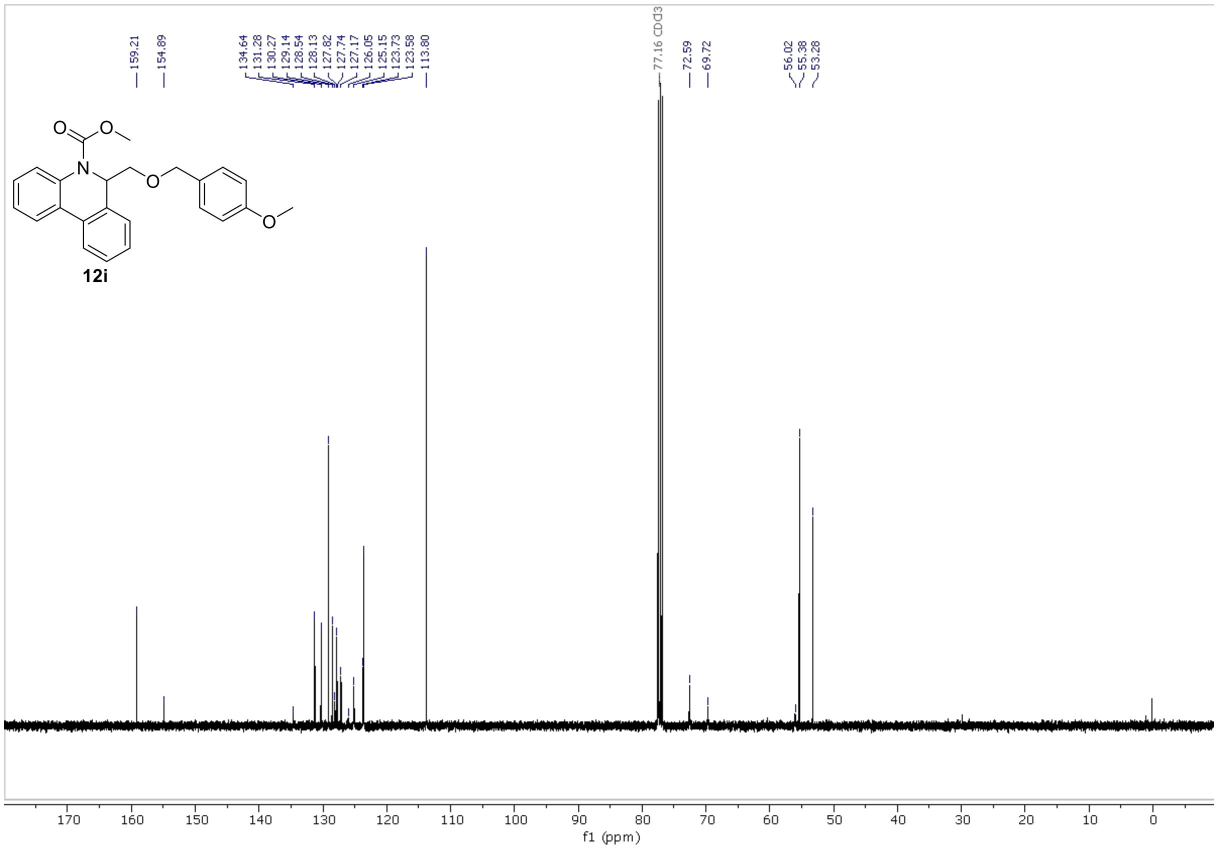

Product obtained using (*S*,*S*)-IBioxtBu:

<Chromatogram>

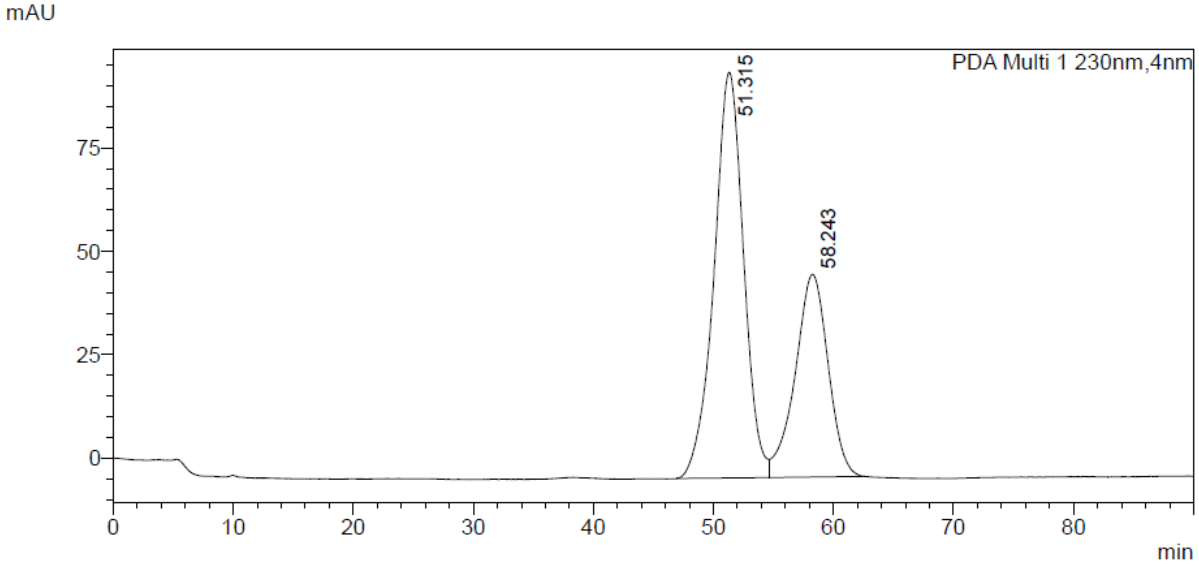

<Peak Table>

PDA Ch1 230nm

Peak#	Ret. Time	Area	Area%
1	51.315	16770515	64.137
2	58.243	9377643	35.863
Total		26148157	100.000

Racemic product obtained using IBioxMe$_4$:

<Chromatogram>

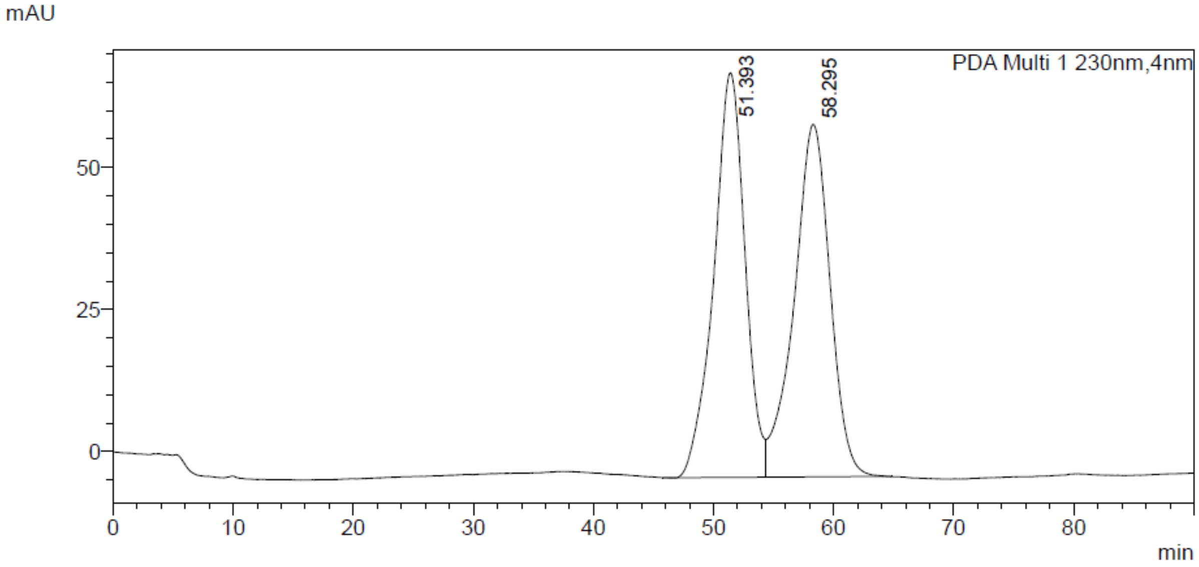

<Peak Table>

PDA Ch1 230nm

Peak#	Ret. Time	Area	Area%
1	51.393	12875934	49.884
2	58.295	12935588	50.116
Total		25811522	100.000

^{1}H NMR (400 MHz, Chloroform-*d*)

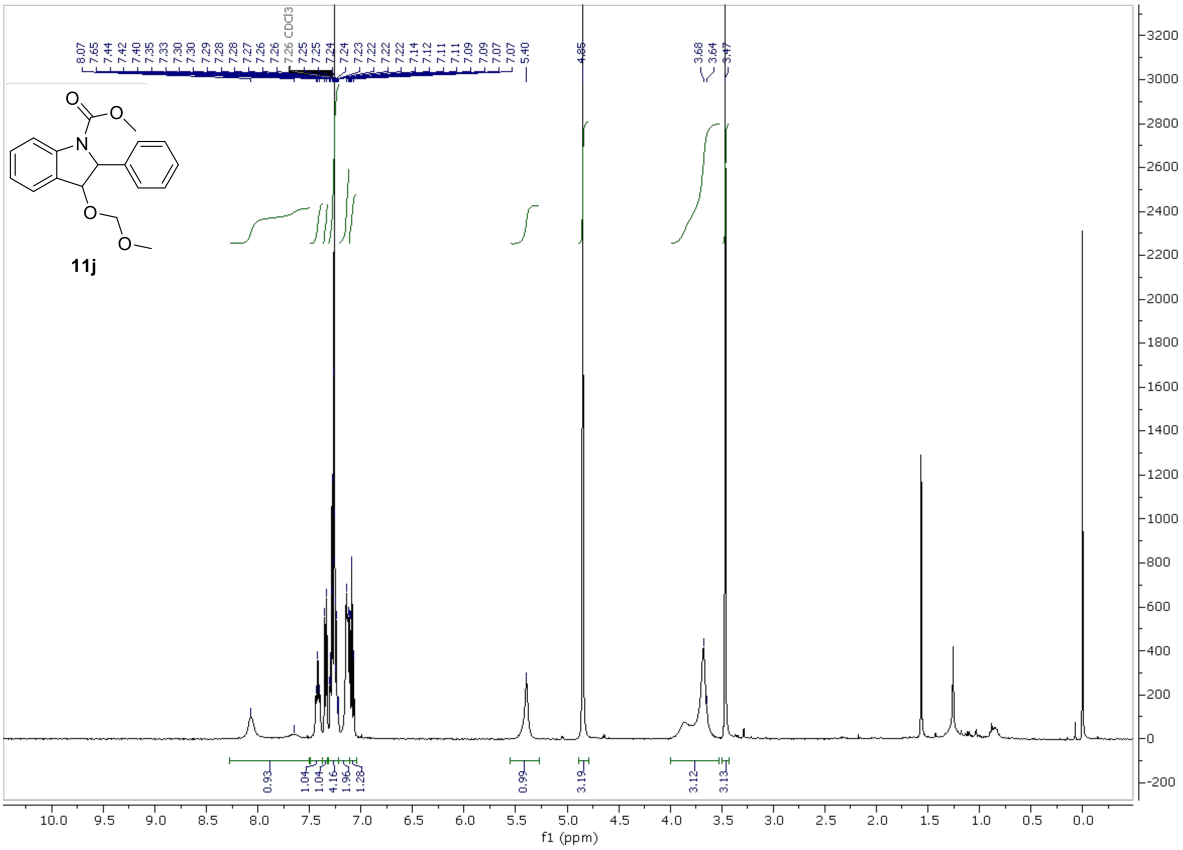

^{13}C{^{1}H} NMR (126 MHz, Chloroform-*d*)

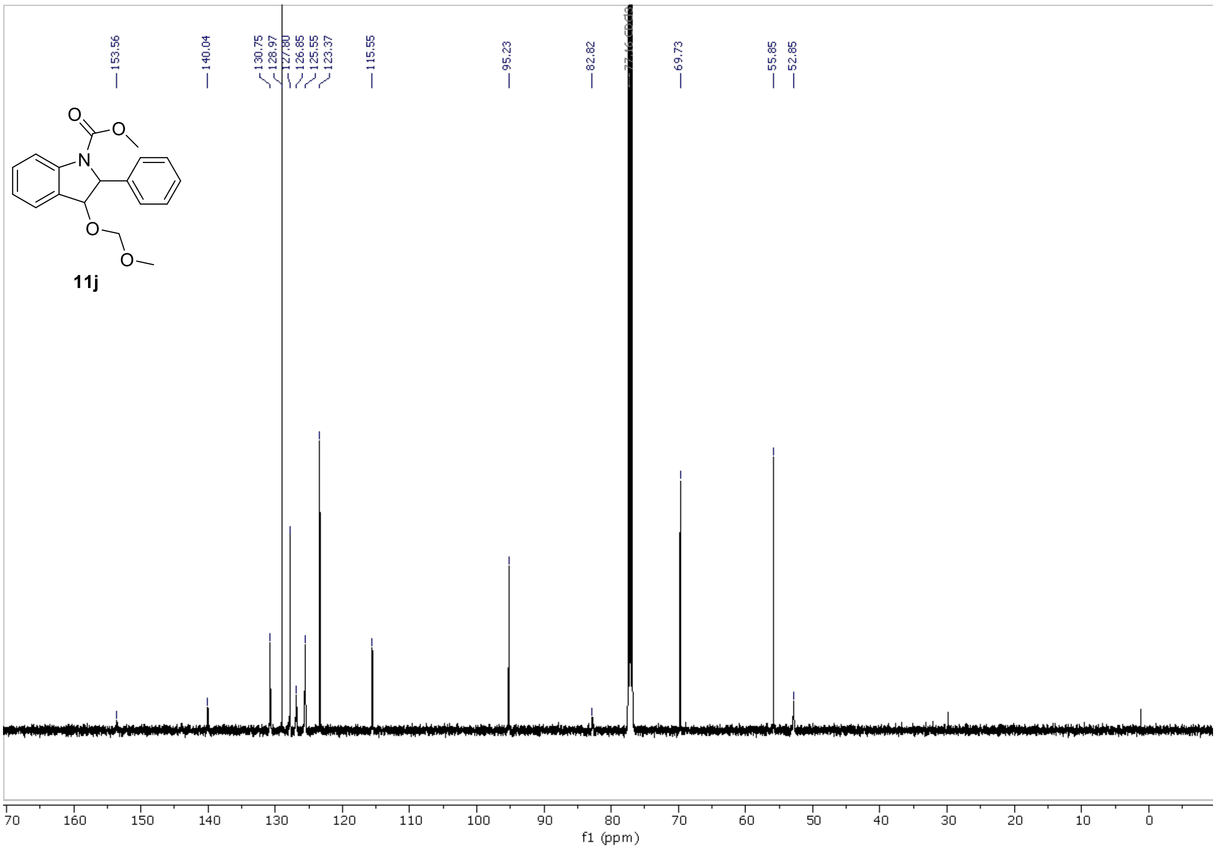

Product obtained using (*S*,*S*)-IBioxtBu:

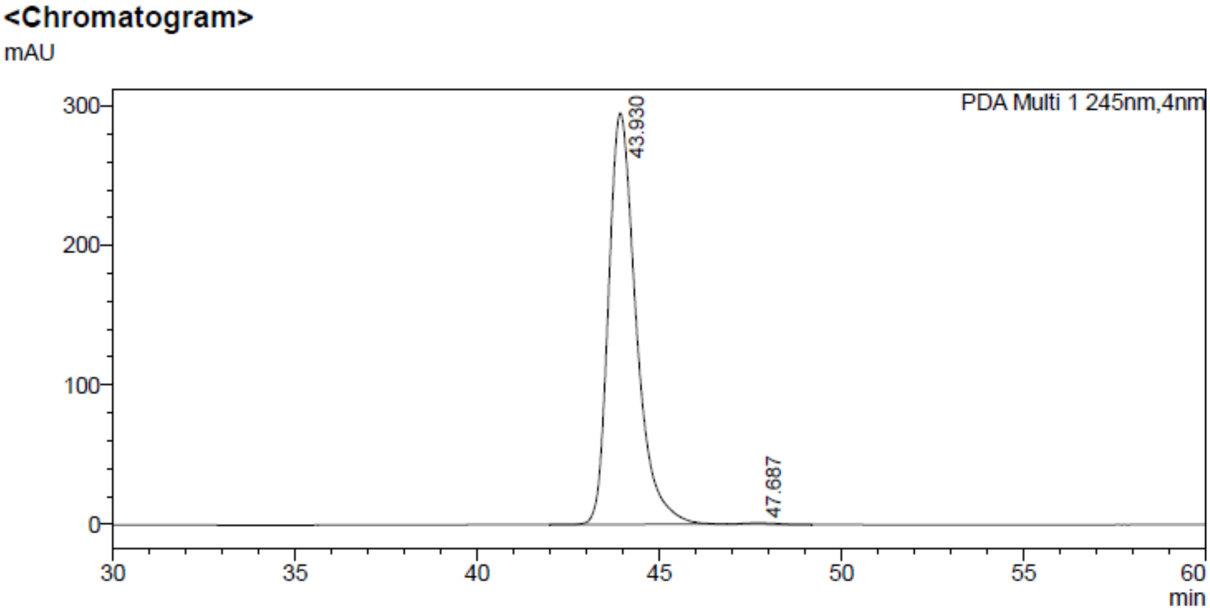

<Peak Table>

PDA Ch1 245nm

Peak#	Ret. Time	Area	Area%
1	43.930	15563110	99.616
2	47.687	60041	0.384
Total		15623151	100.000

Racemic product obtained using IBioxMe$_4$:

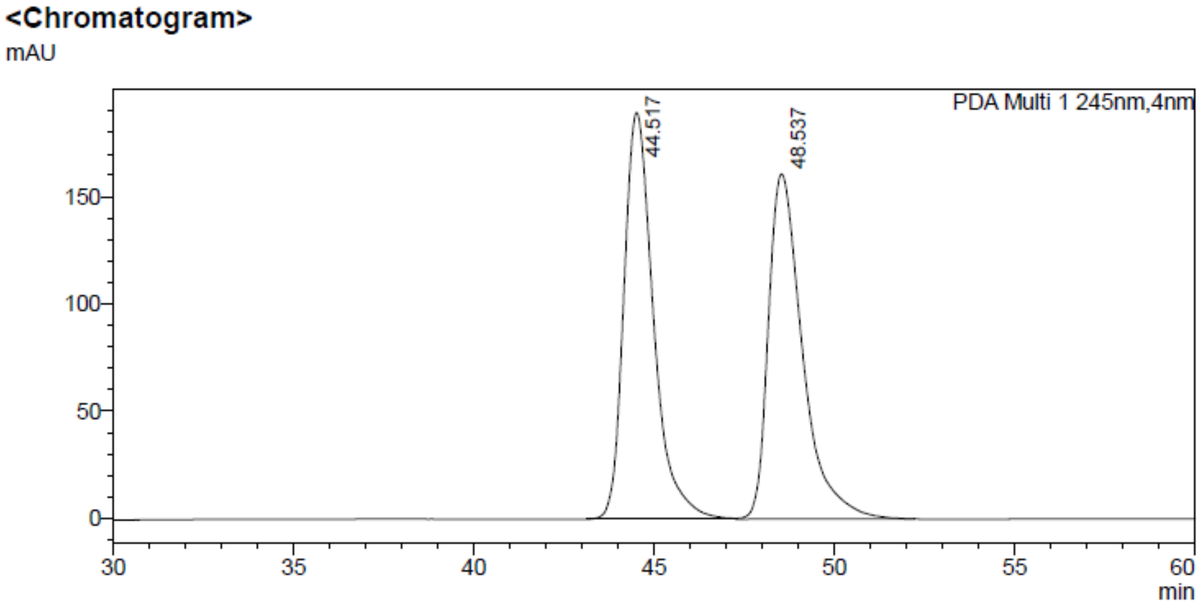

<Peak Table>

PDA Ch1 245nm

Peak#	Ret. Time	Area	Area%
1	44.517	10846819	50.528
2	48.537	10619972	49.472
Total		21466790	100.000

^{1}H NMR (400 MHz, Chloroform-*d*)

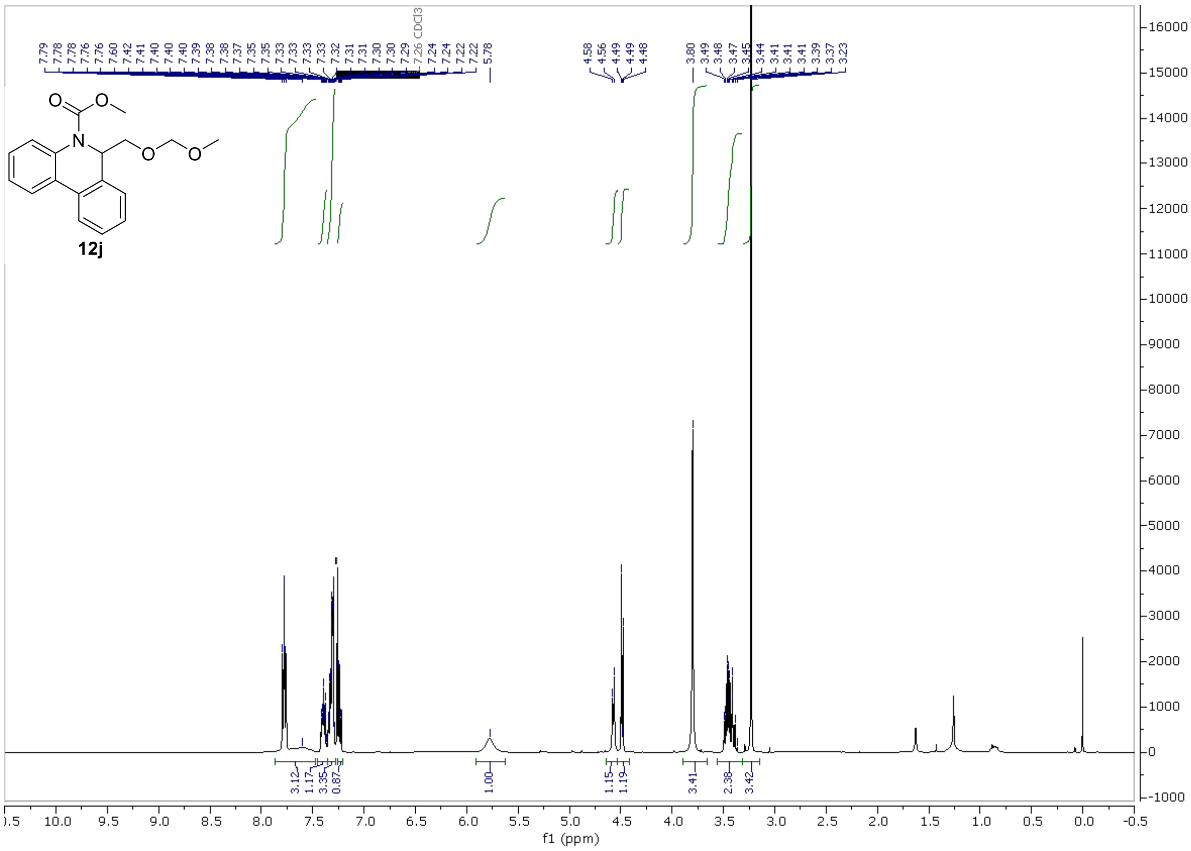

^{13}C{^{1}H} NMR (101 MHz, Chloroform-*d*)

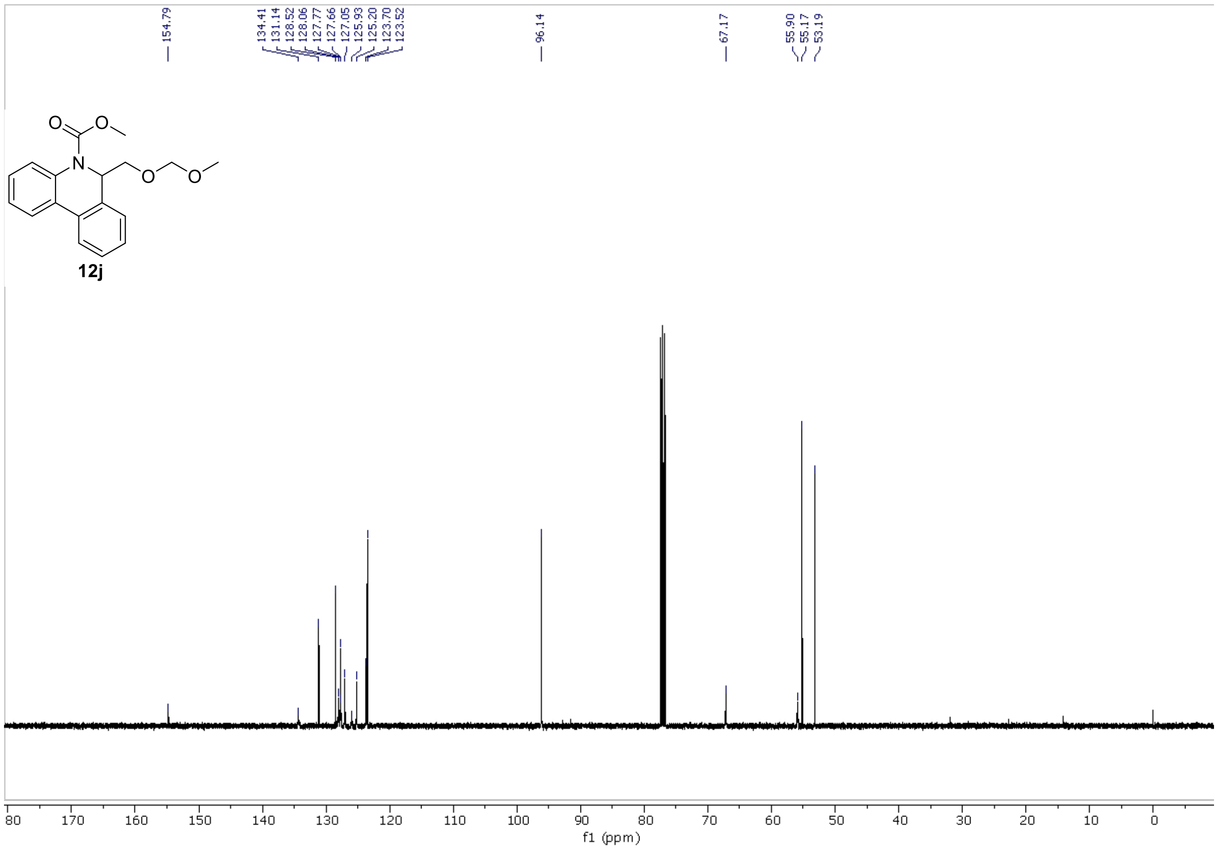

Product obtained using (*S*,*S*)-IBioxtBu:

<Chromatogram>

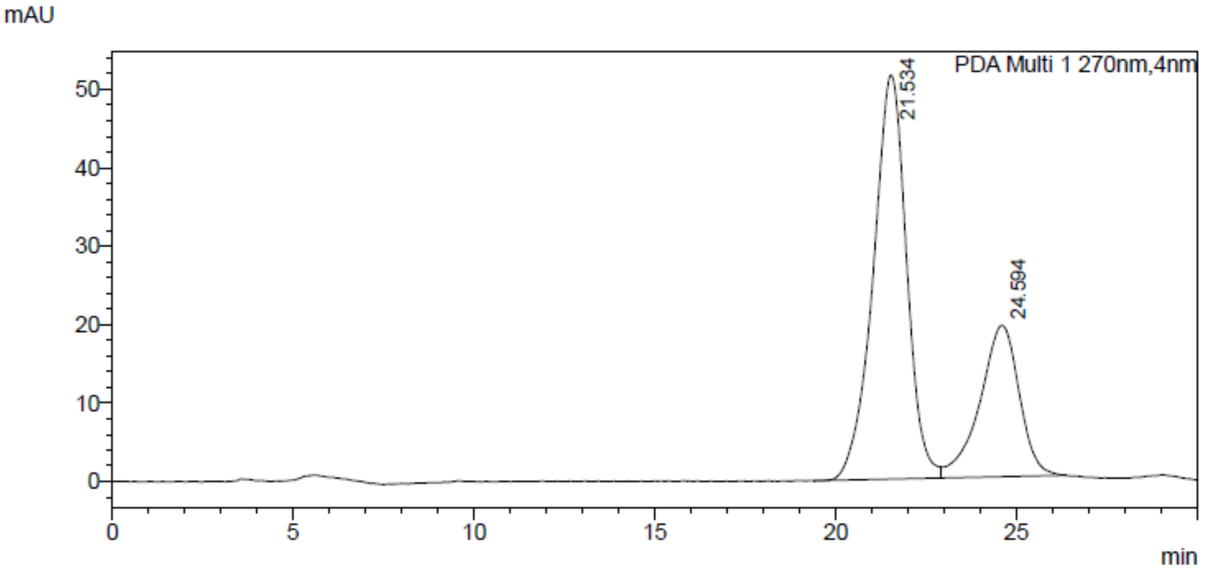

<Peak Table>

PDA Ch1 270nm

Peak#	Ret. Time	Area	Area%
1	21.534	3314975	69.357
2	24.594	1464638	30.643
Total		4779613	100.000

Racemic product obtained using IBioxMe$_4$:

<Chromatogram>

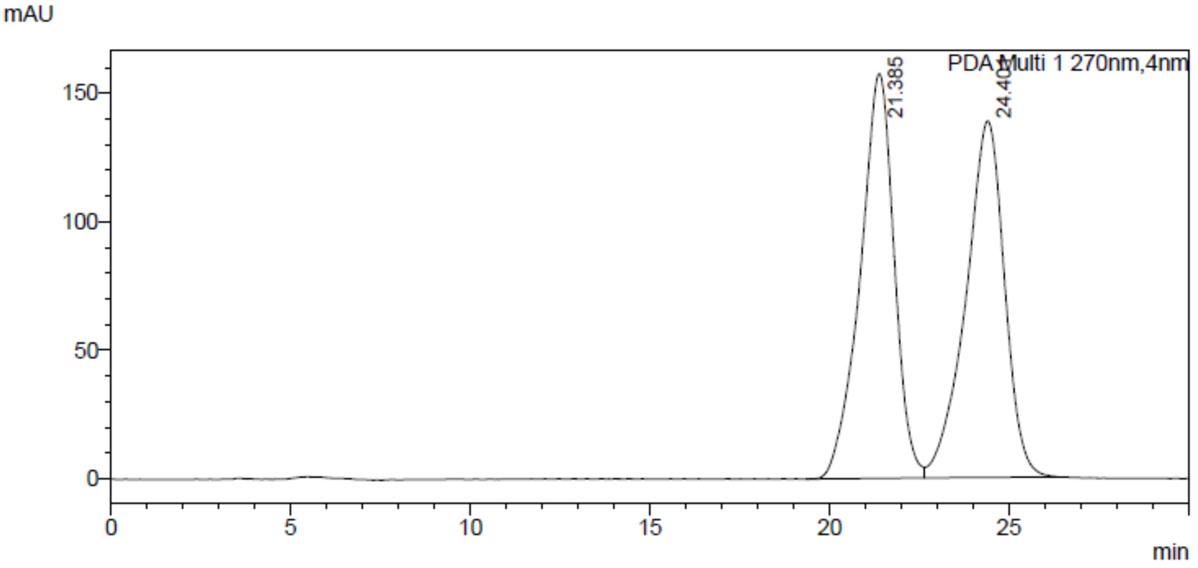

<Peak Table>

PDA Ch1 270nm

Peak#	Ret. Time	Area	Area%
1	21.385	10373093	49.910
2	24.401	10410461	50.090
Total		20783554	100.000

NMR and HPLC Spectrum Data in the Core Construction of Cryptowolinol

^{1}H NMR (400 MHz, Chloroform-*d*)

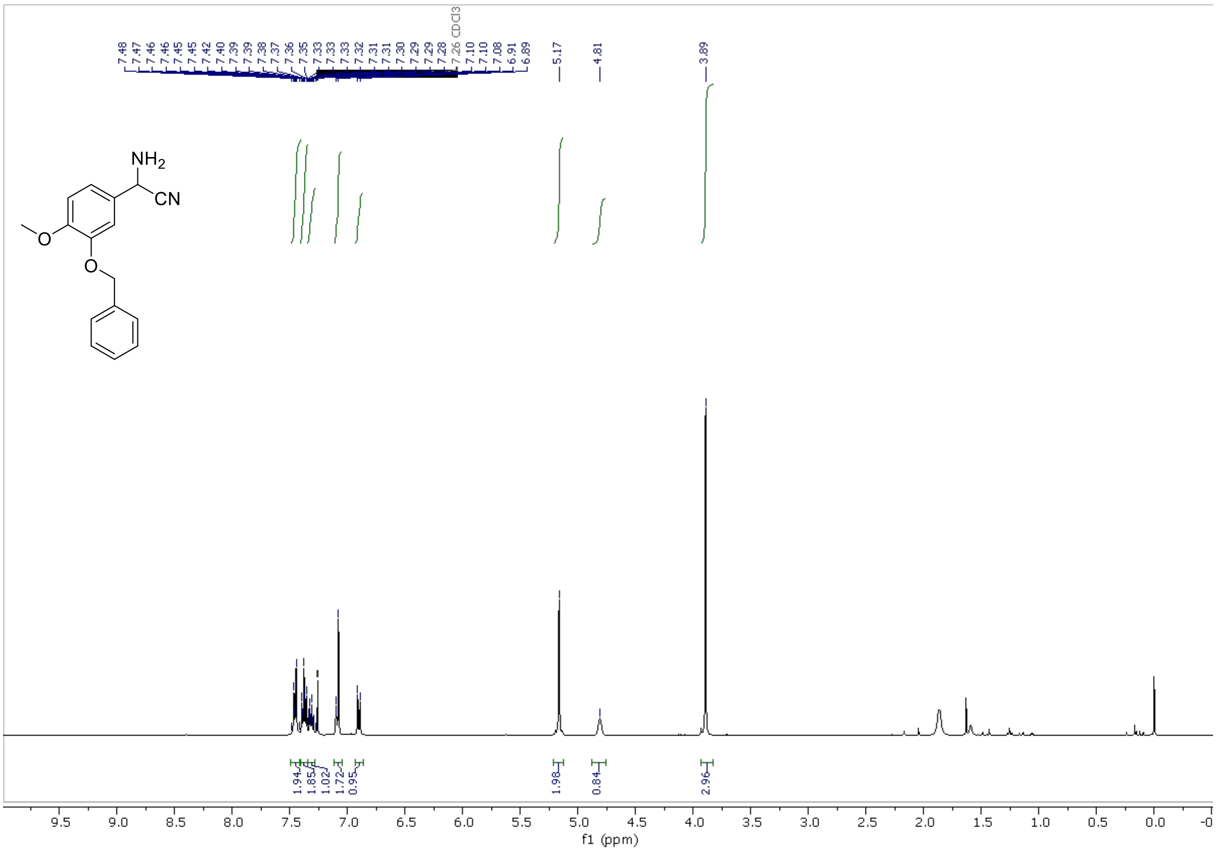

^{13}C{^{1}H} NMR (126 MHz, Chloroform-*d*)

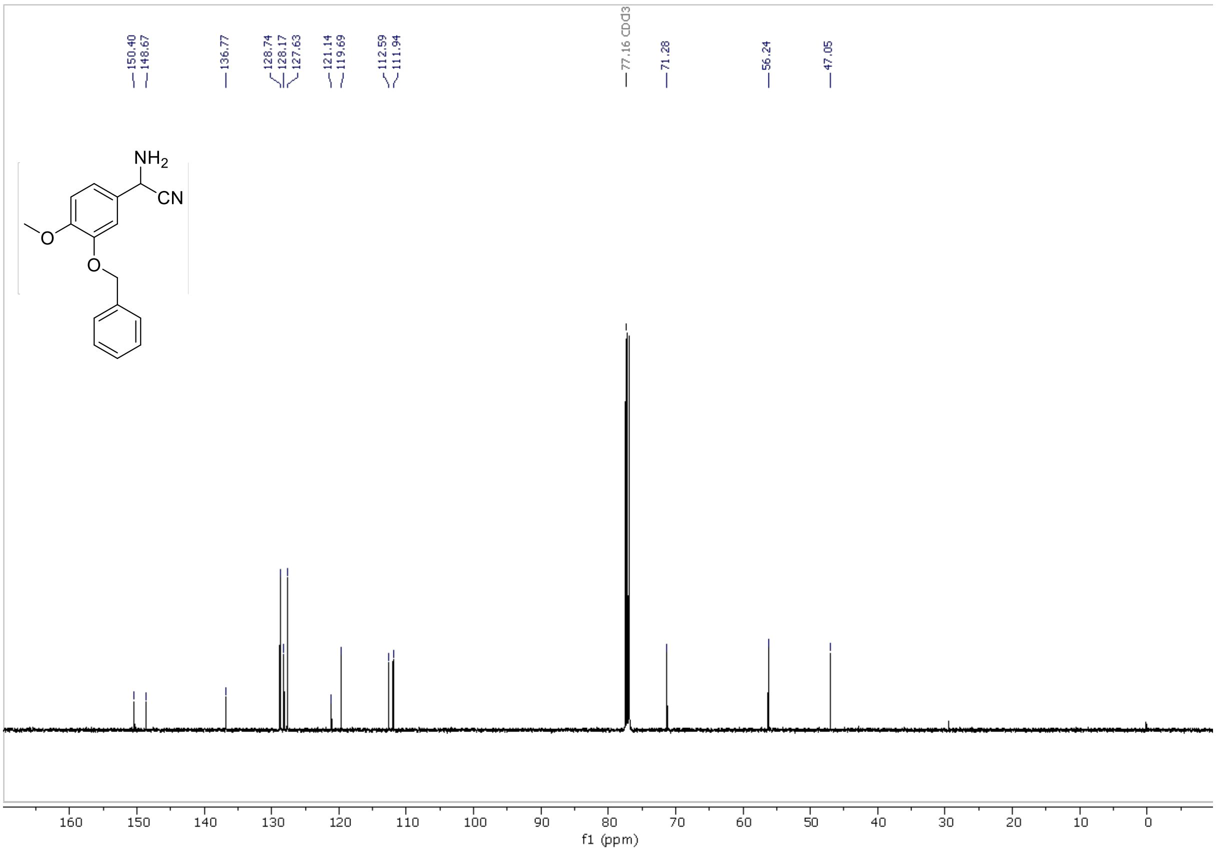

^{1}**H NMR** (500 MHz, Chloroform-*d*)

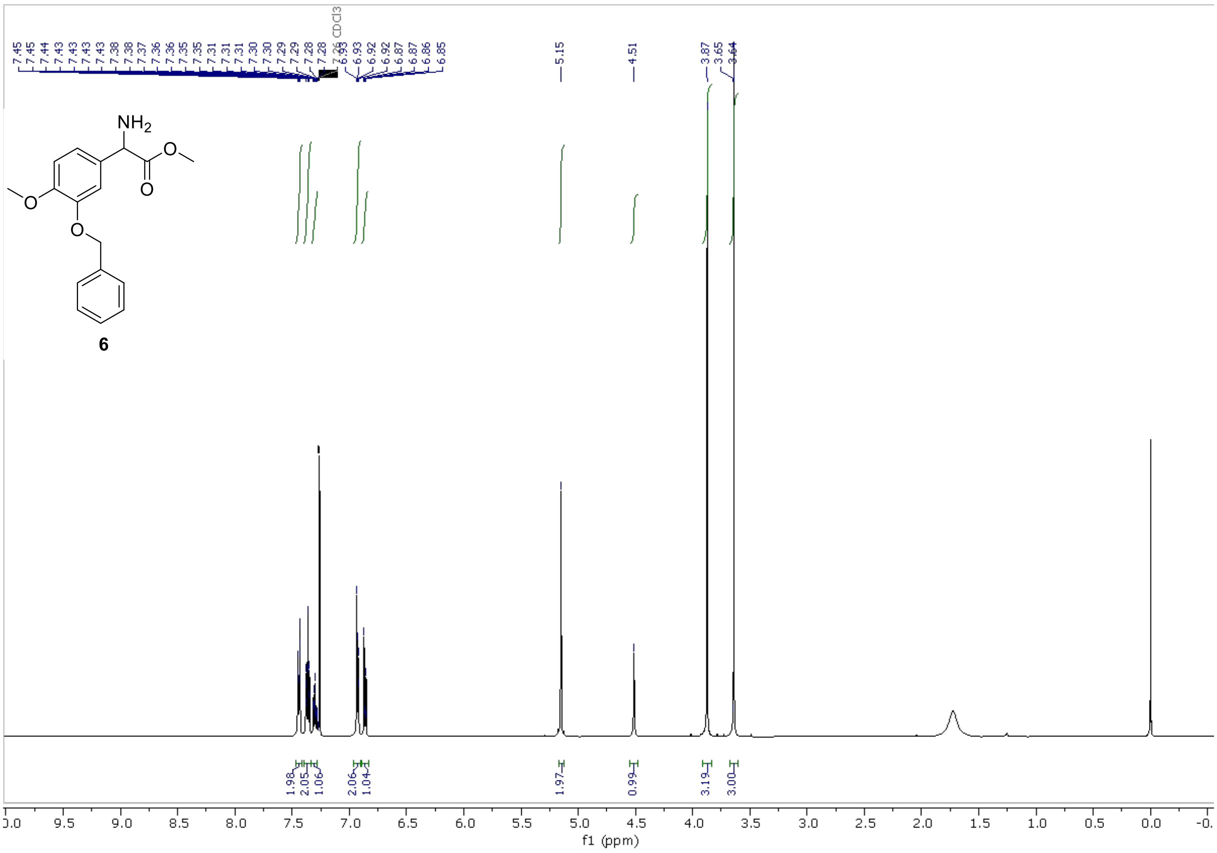

^{13}**C{^{1}H} NMR** (126 MHz, Chloroform-*d*)

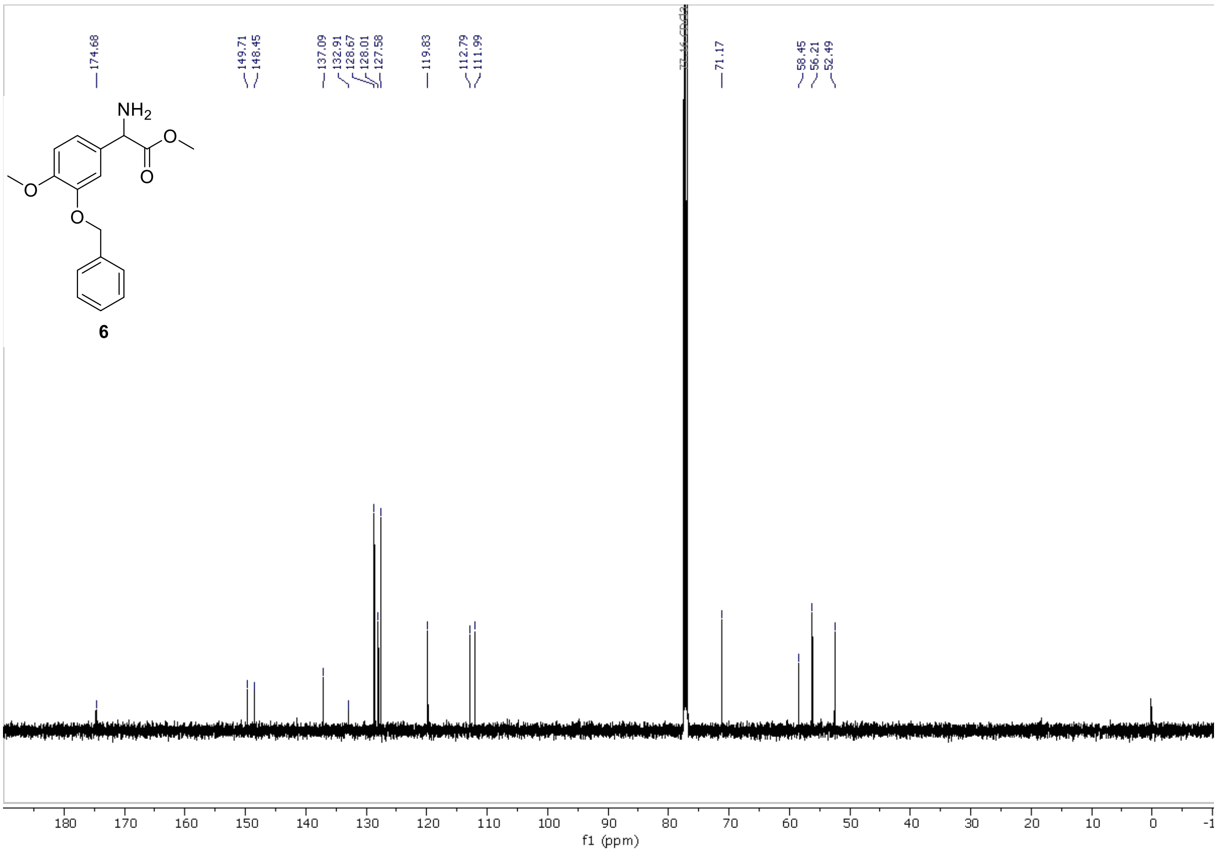

^{1}H NMR (400 MHz, Chloroform-*d*)

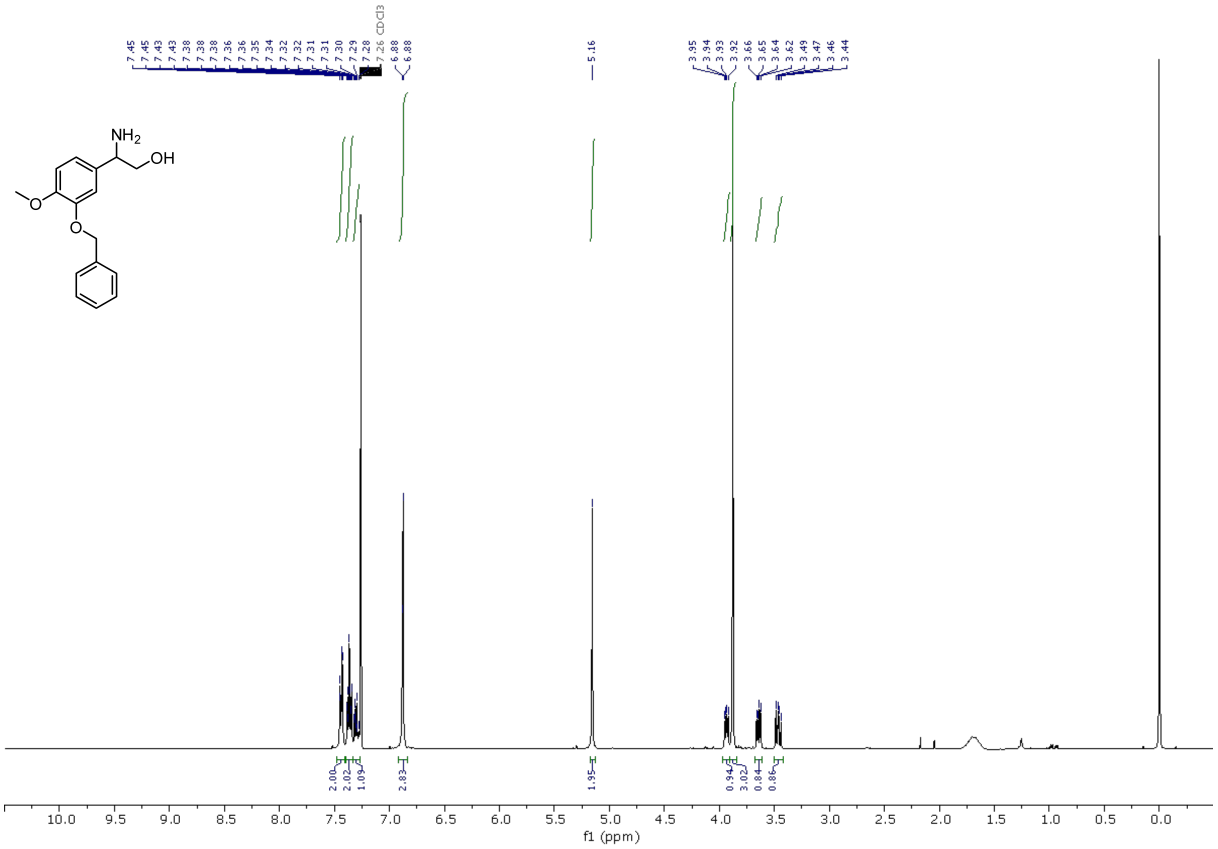

^{13}C{^{1}H} NMR (101 MHz, Chloroform-*d*)

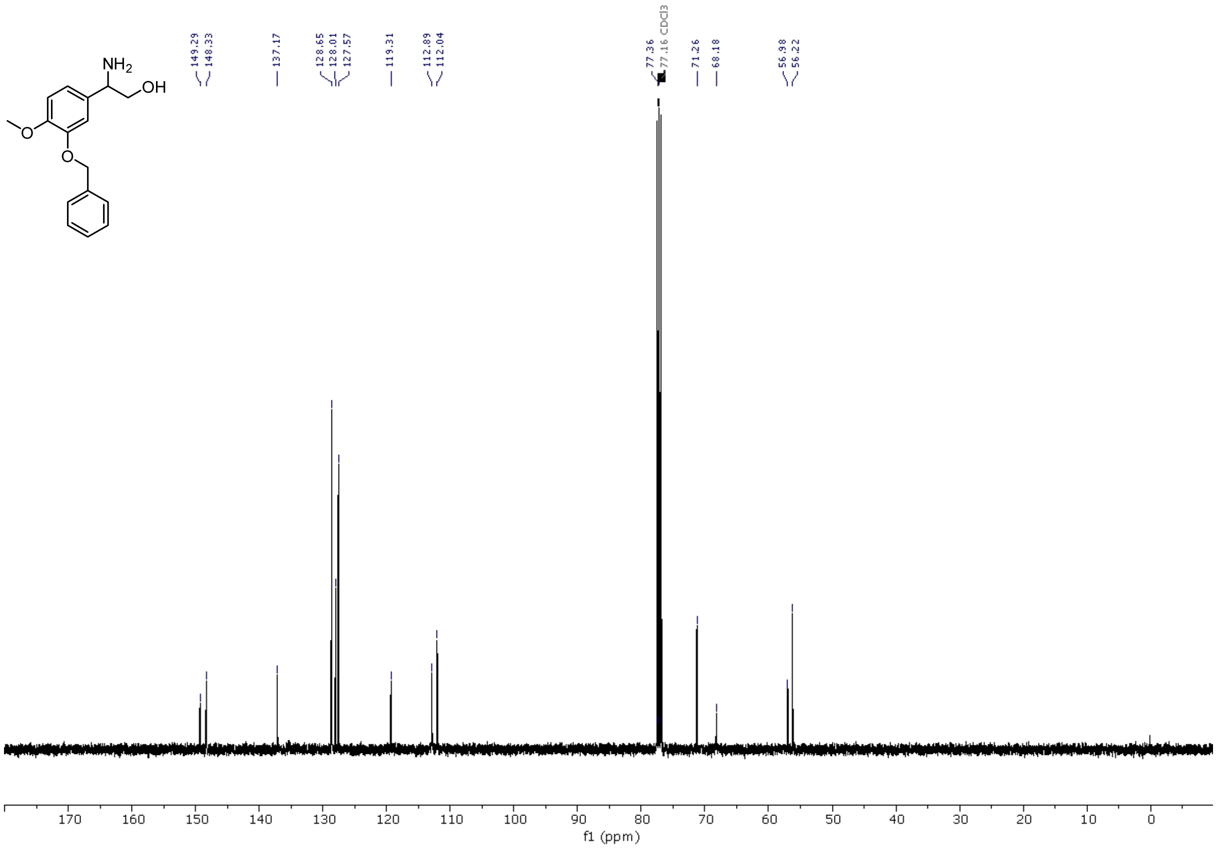

^{1}H NMR (400 MHz, Chloroform-*d*)

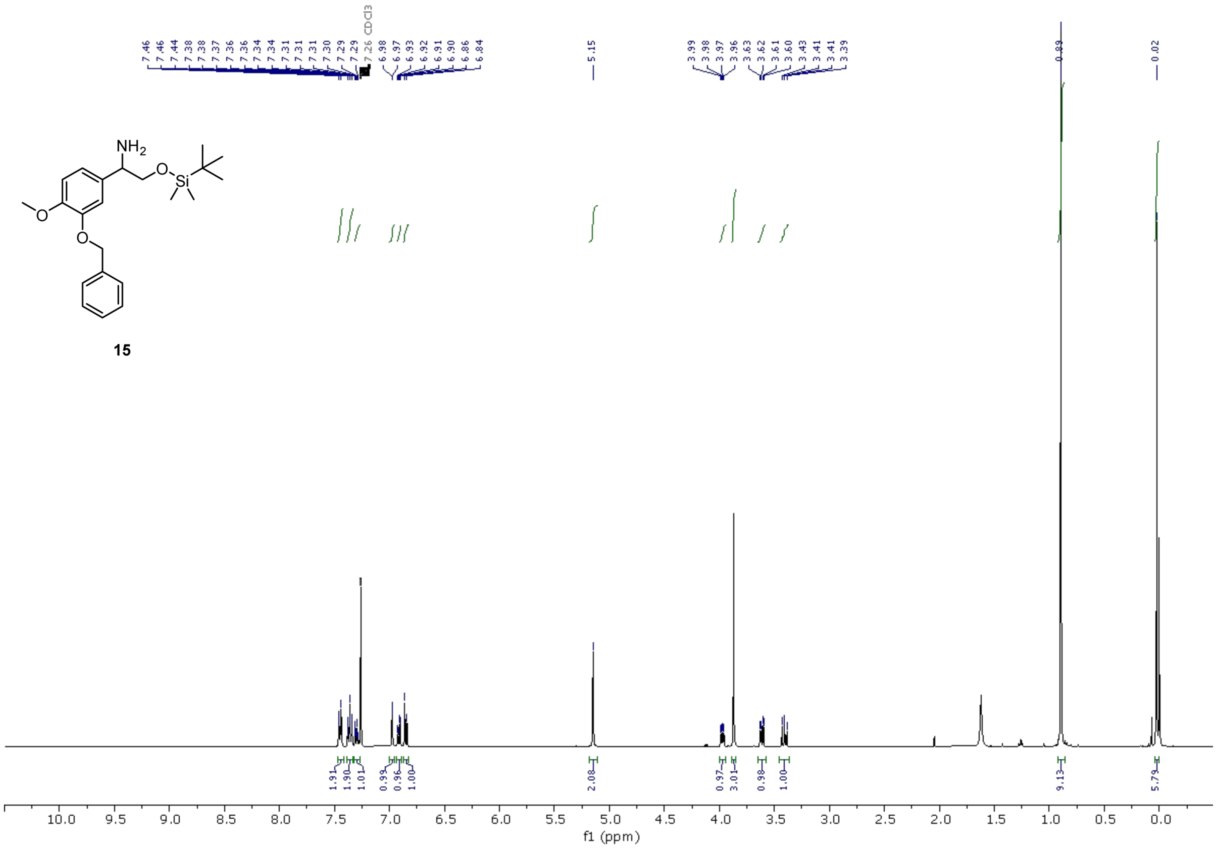

^{13}C{^{1}H} NMR (101 MHz, Chloroform-*d*)

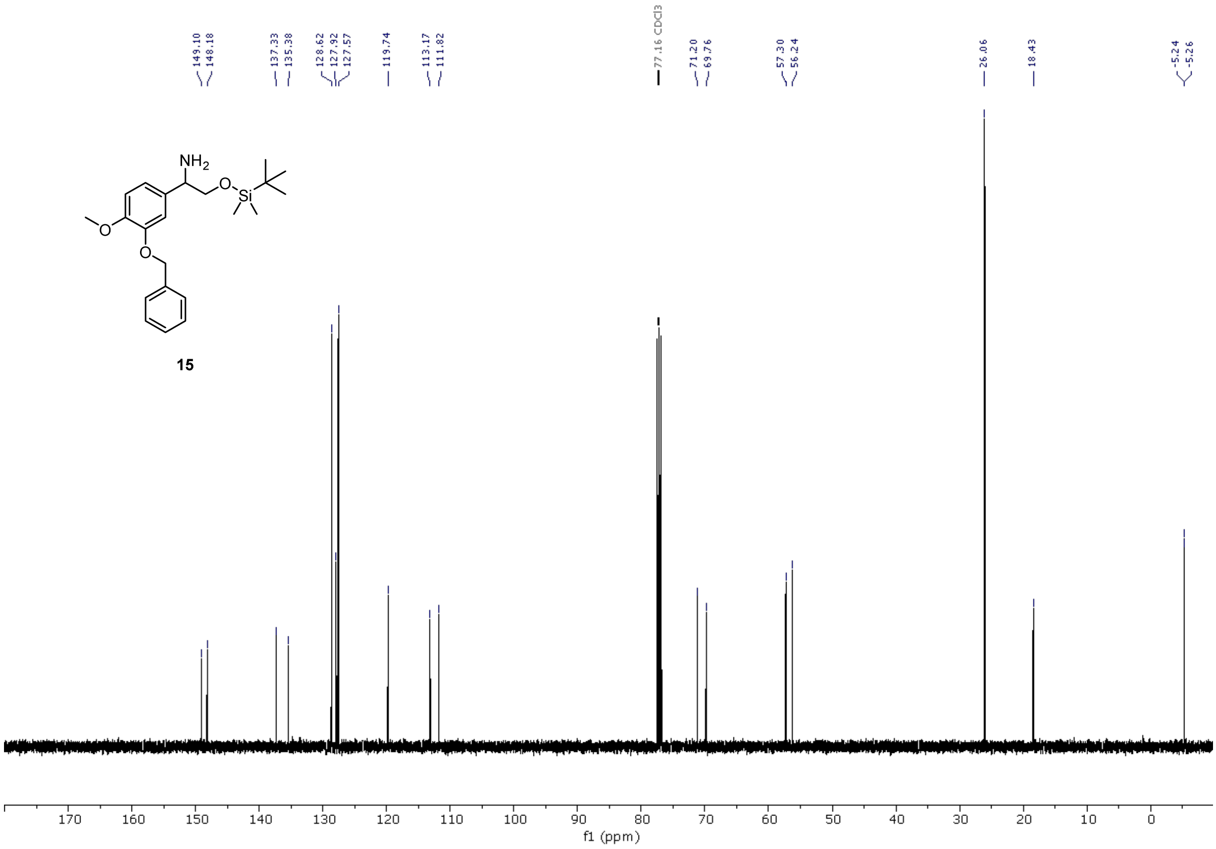

^{1}H NMR (400 MHz, Chloroform-*d*)

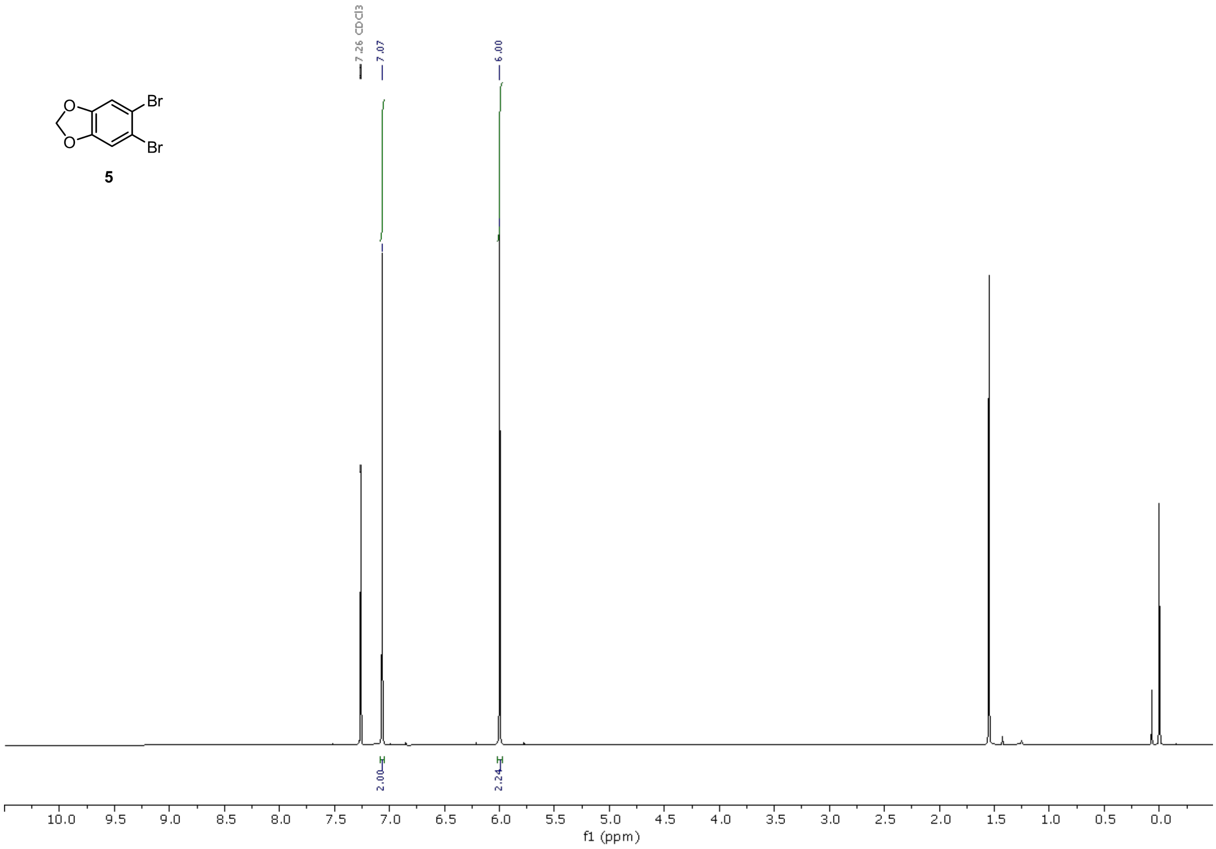

^{13}C{^{1}H} NMR (101 MHz, Chloroform-*d*)

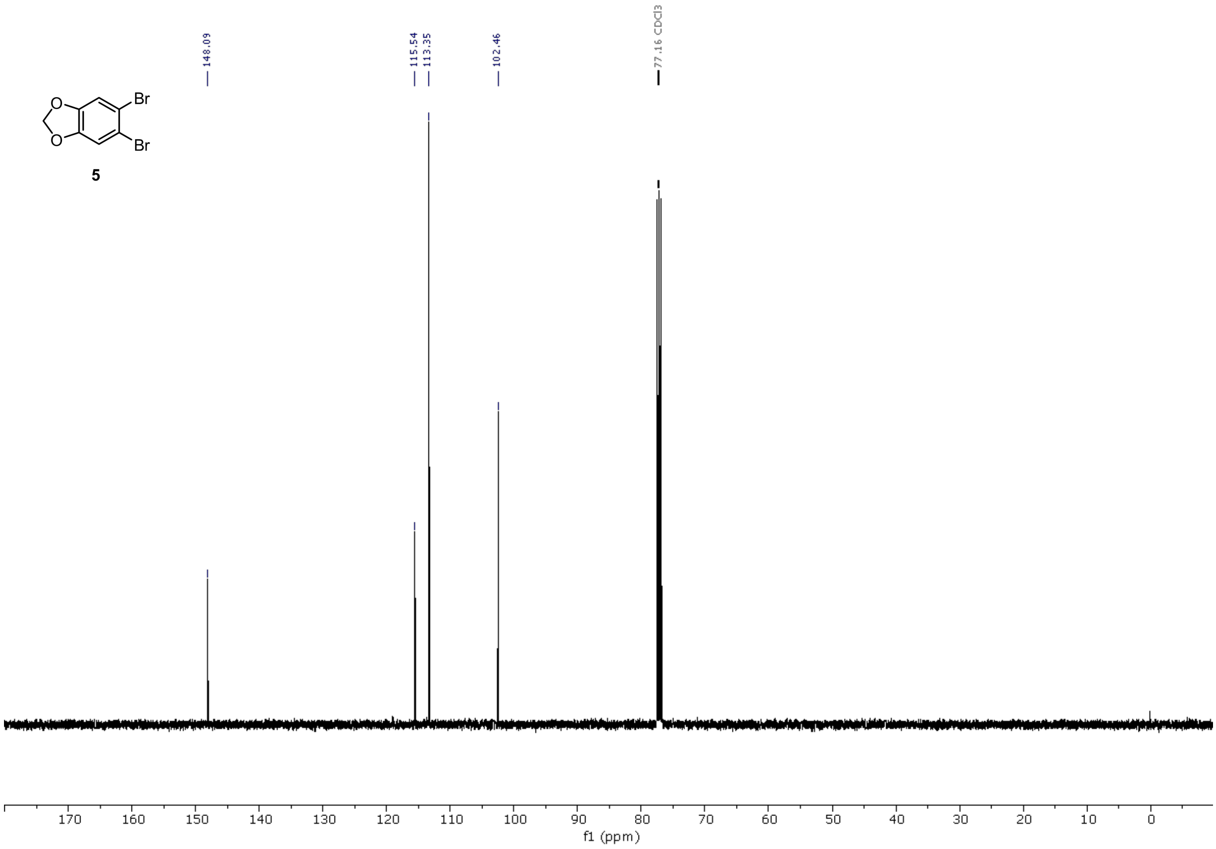

^{1}H NMR (400 MHz, Chloroform-*d*)

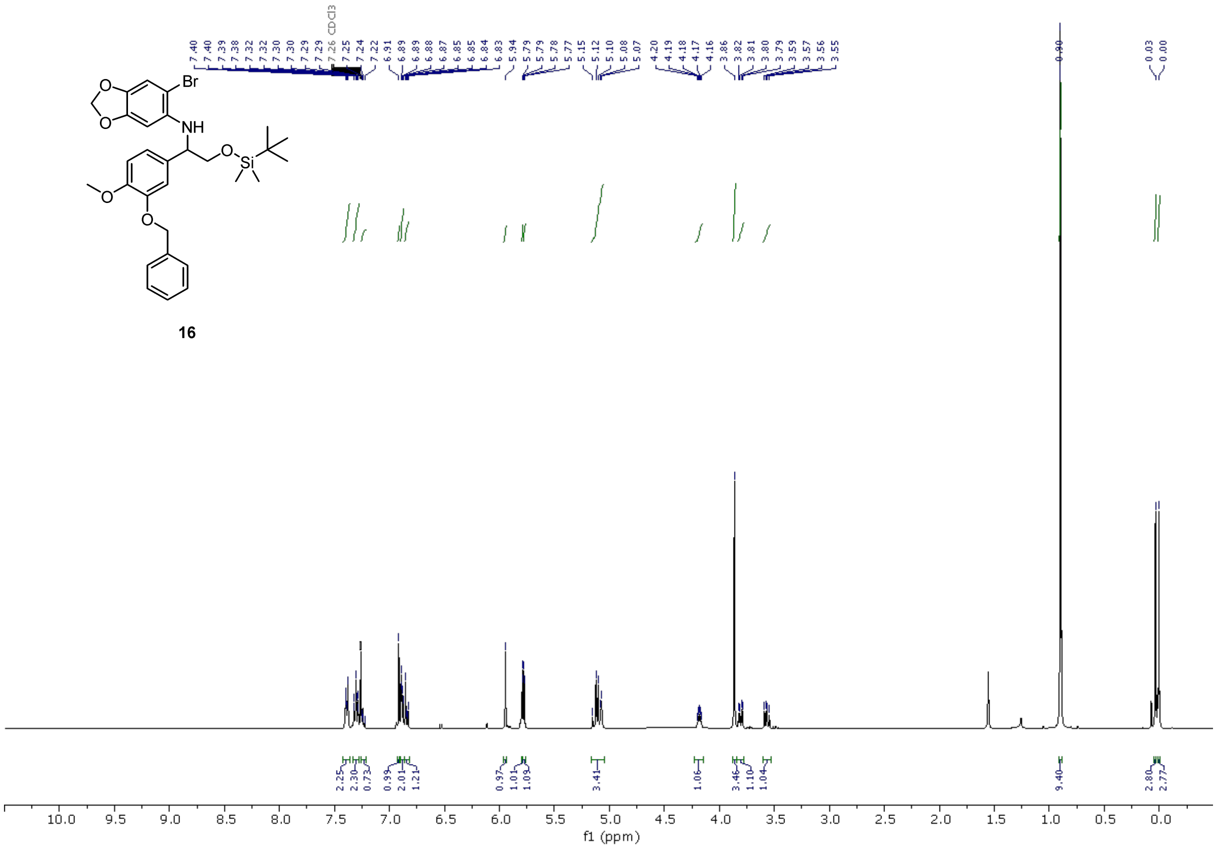

^{13}C{^{1}H} NMR (101 MHz, Chloroform-*d*)

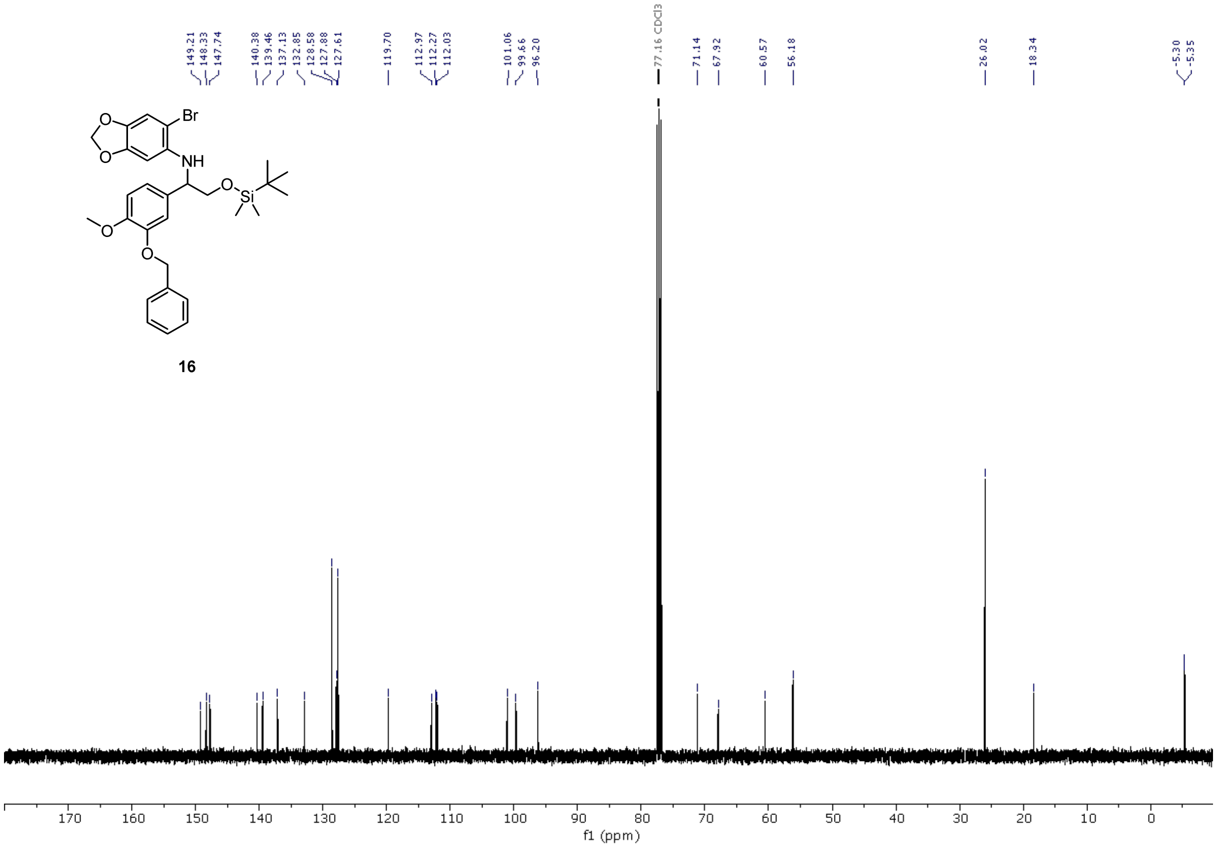

^{1}**H NMR** (400 MHz, Chloroform-*d*)

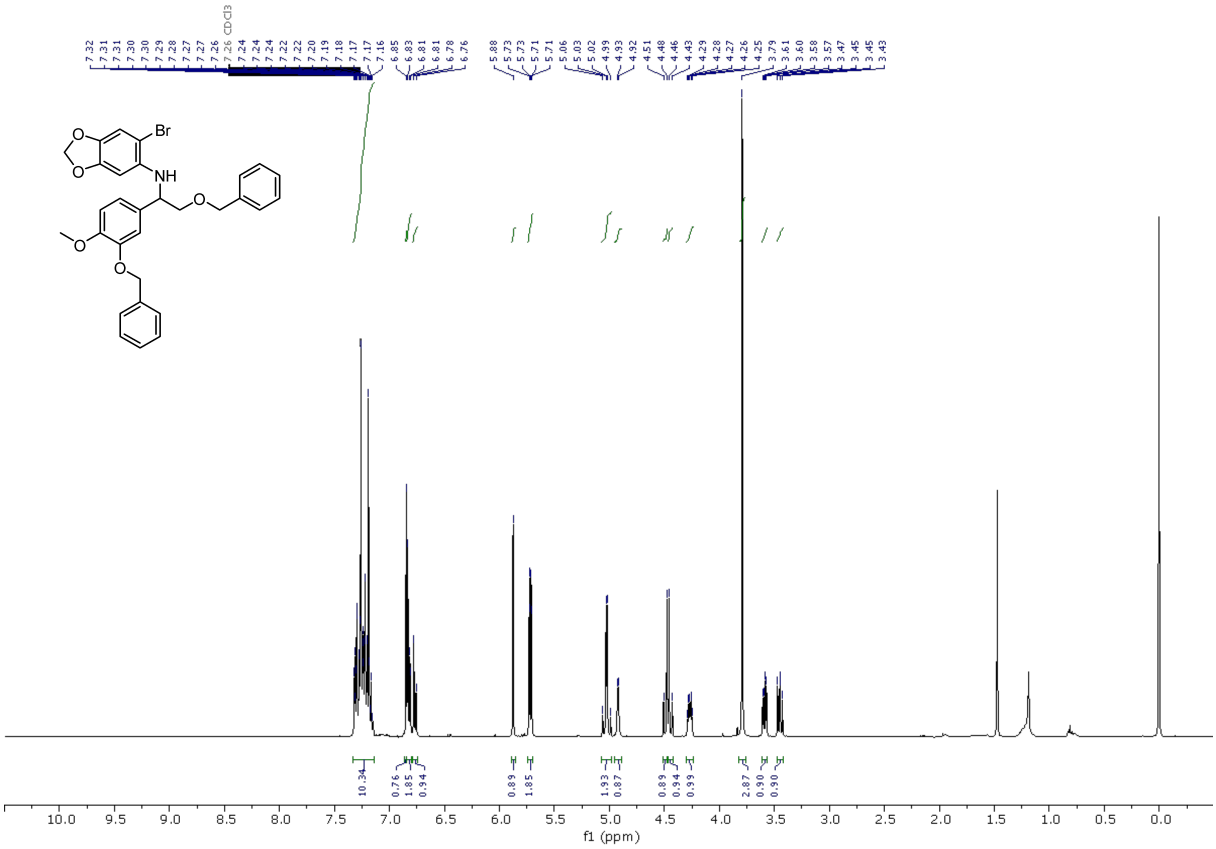

^{13}**C{^{1}H} NMR** (126 MHz, Chloroform-*d*)

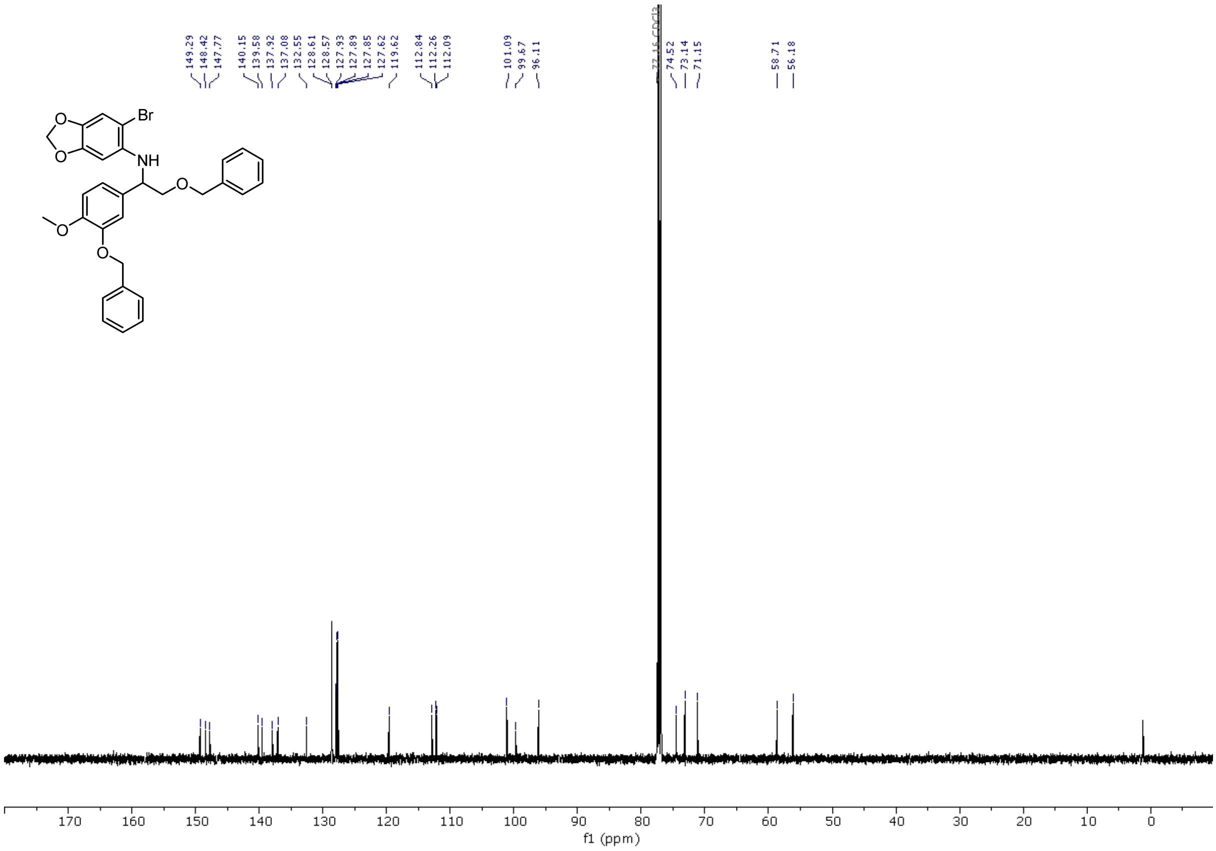

^{1}H NMR (400 MHz, Chloroform-*d*)

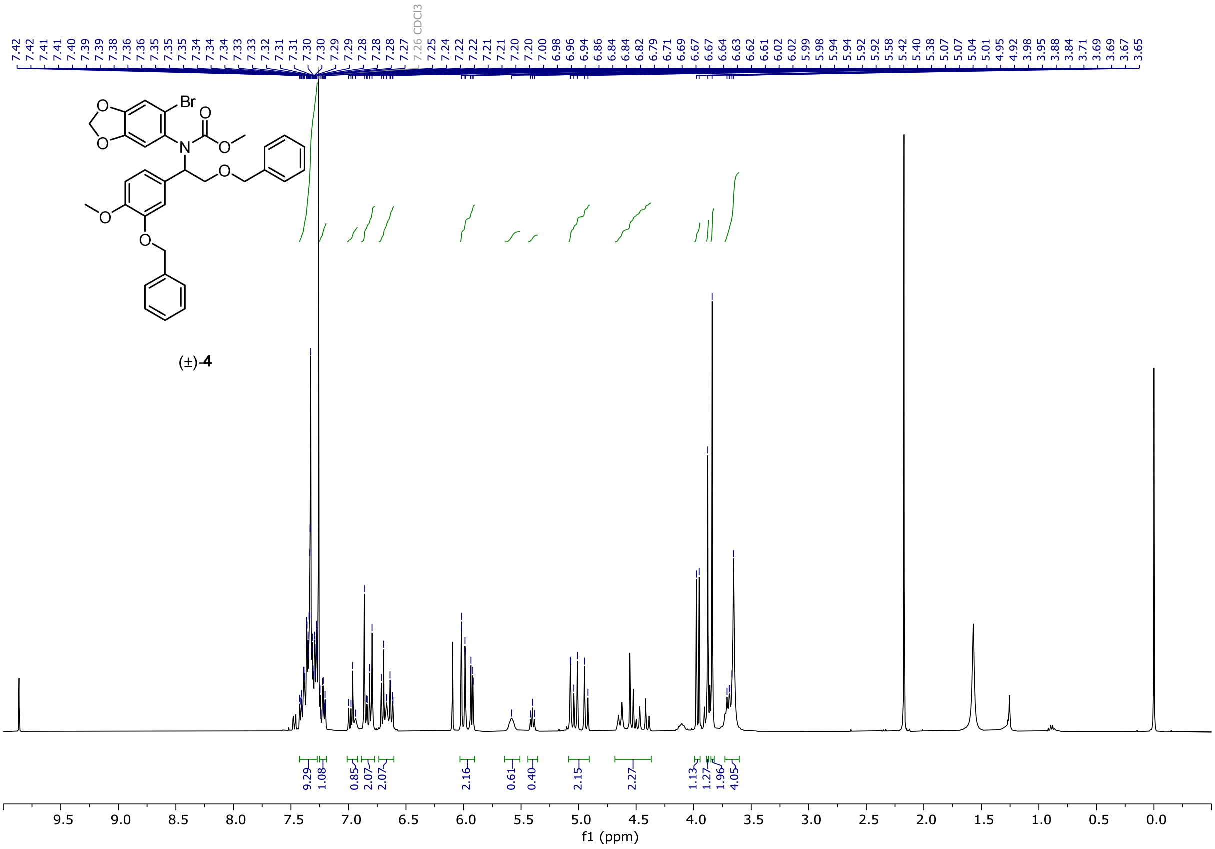

^{13}C{^{1}H} NMR (126 MHz, Chloroform-*d*)

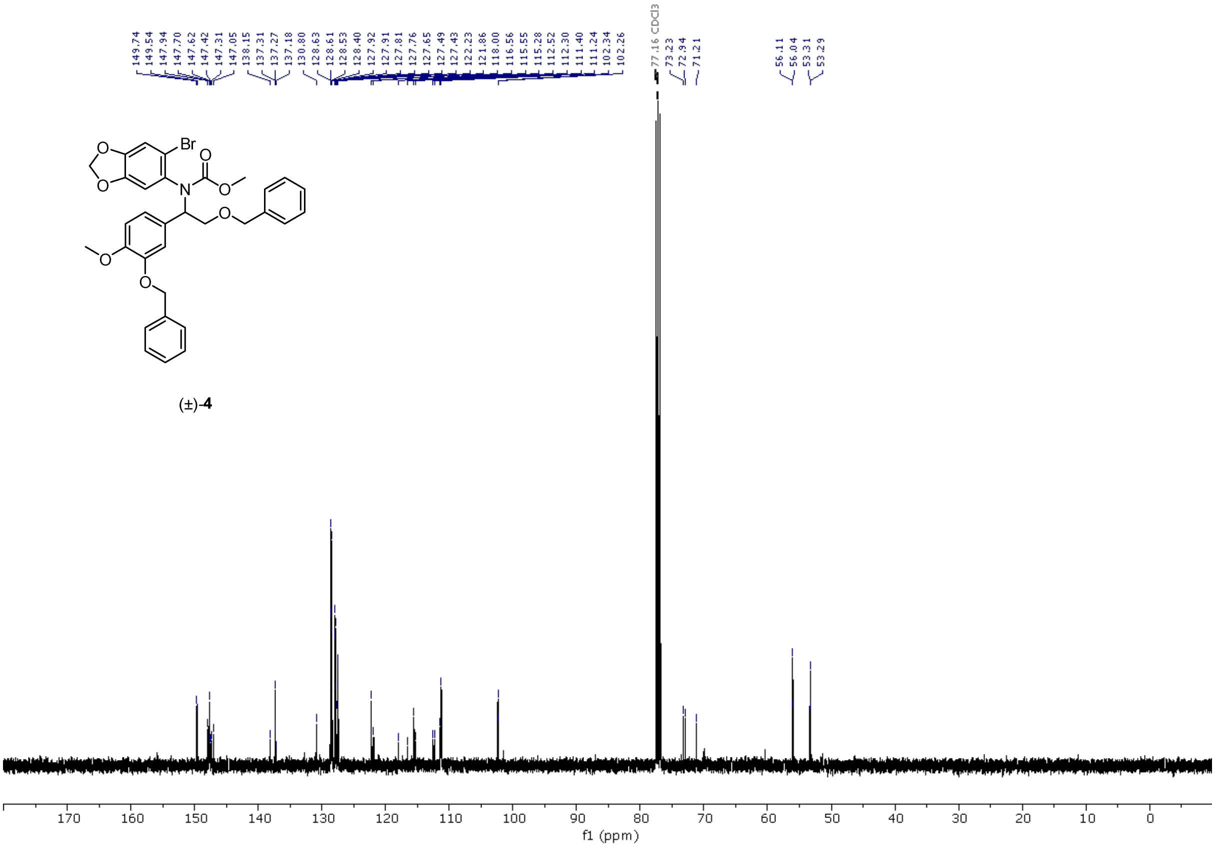

^{1}H NMR (400 MHz, Dichloromethane-*d2*)

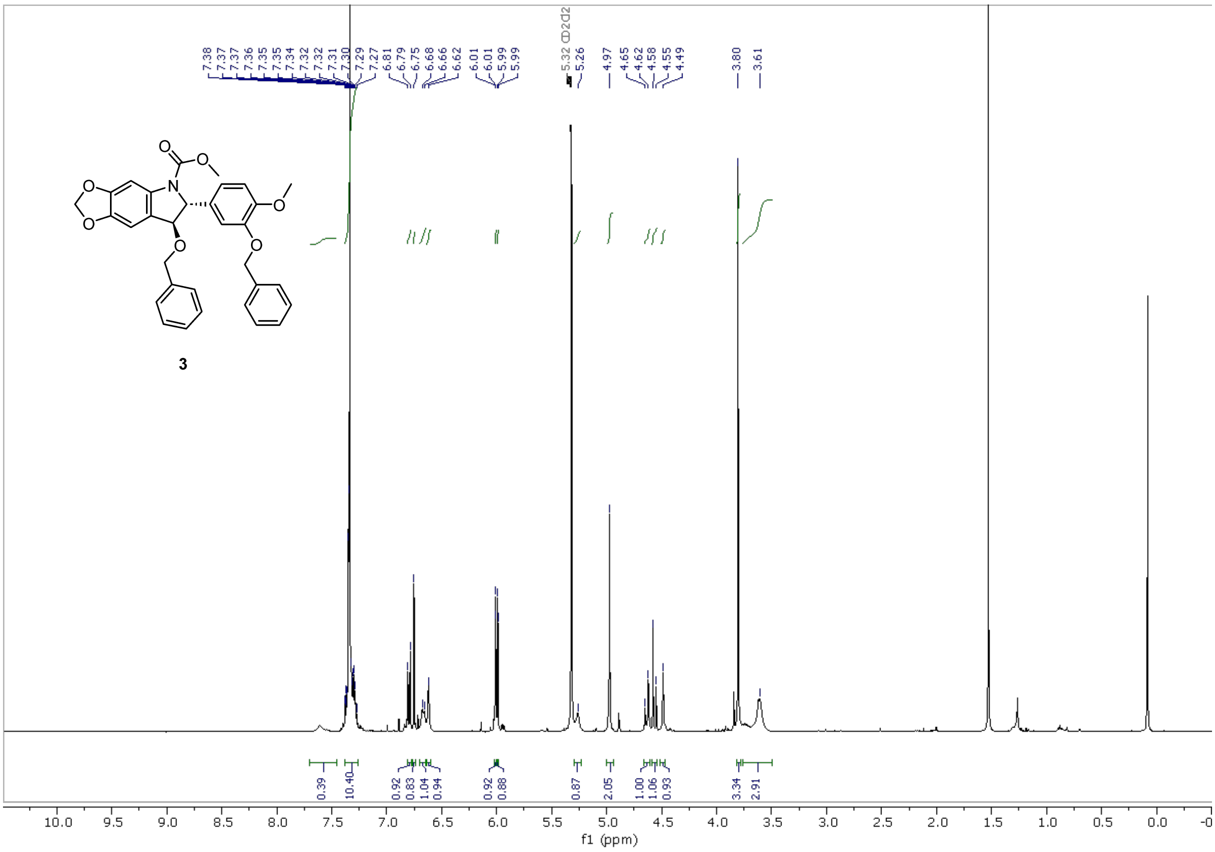

^{13}C{^{1}H} NMR (126 MHz, Dichloromethane-*d2*)

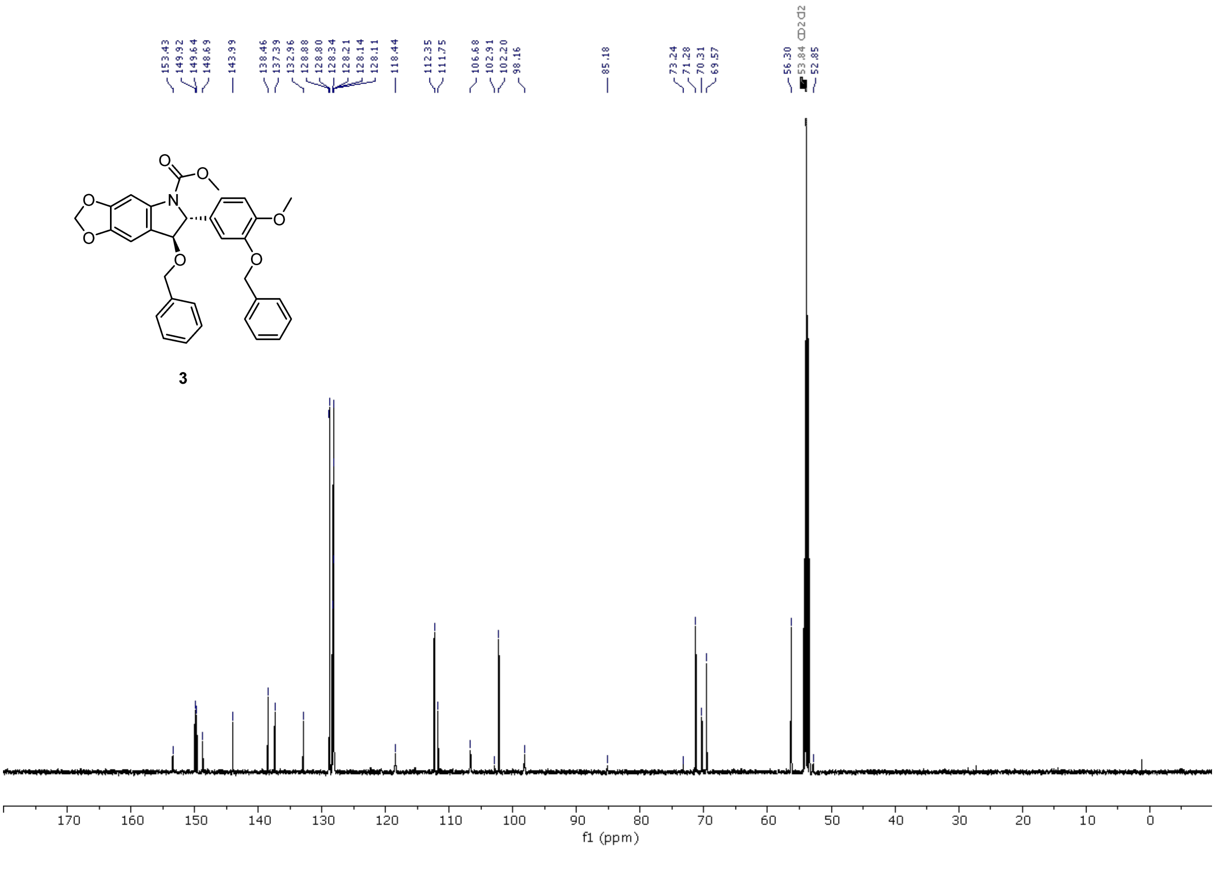

Racemic product obtained using (*rac*)-**L**^{2}:

<Chromatogram>

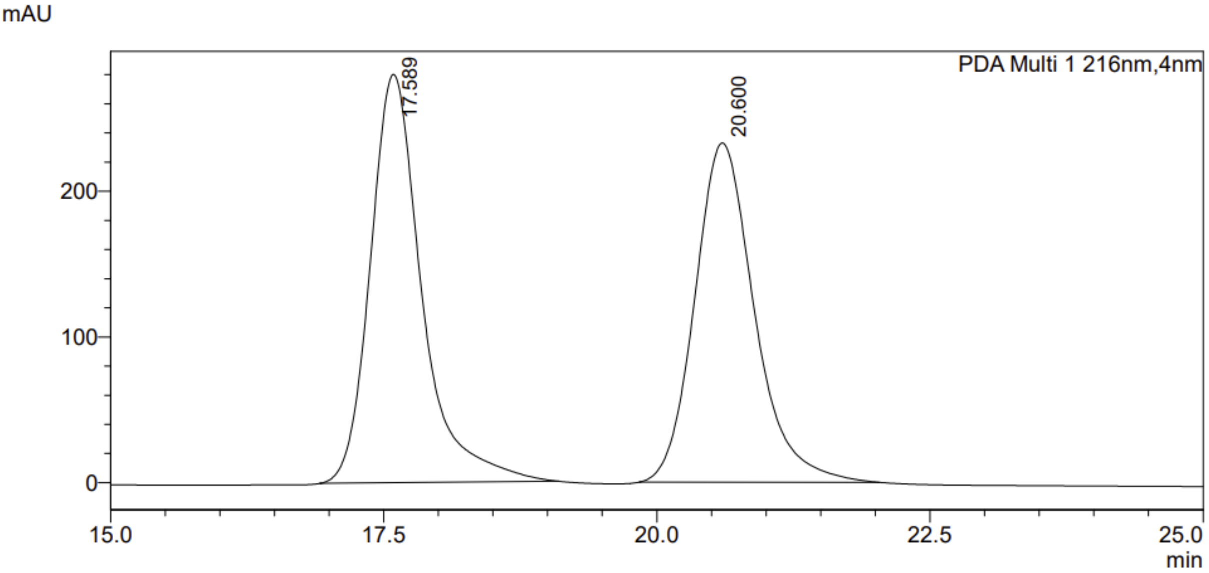

<Peak Table>

PDA Ch1 216nm

Peak#	Ret. Time	Area	Area%
1	17.589	9189424	51.024
2	20.600	8820649	48.976
Total		18010074	100.000

Product obtained using (*R*,*R*)-**L**^{2}:

<Chromatogram>

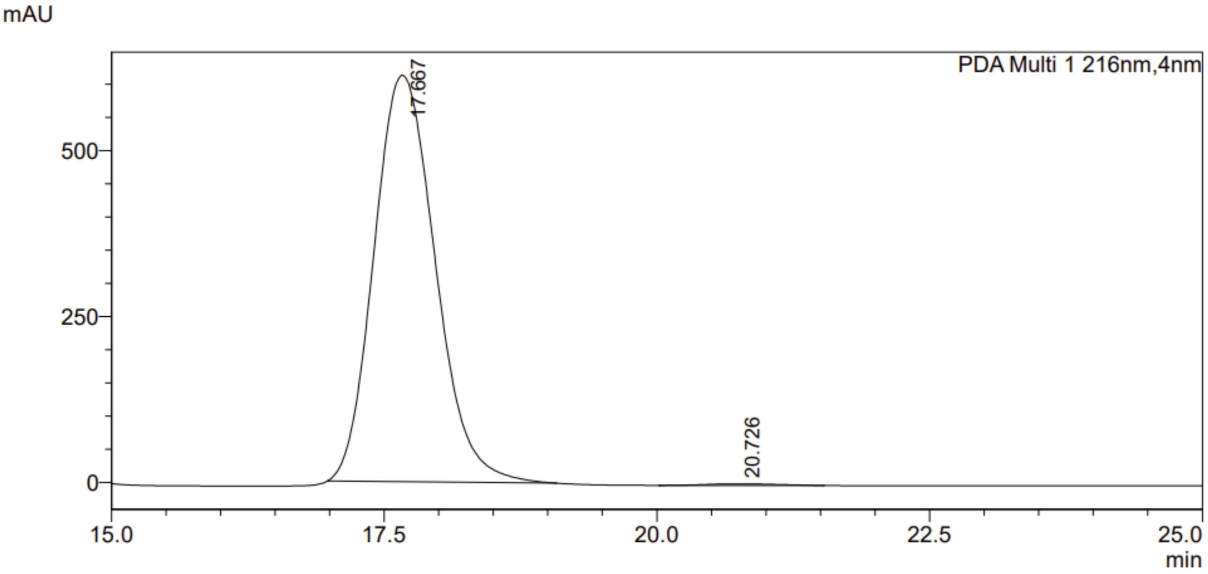

<Peak Table>

PDA Ch1 216nm

Peak#	Ret. Time	Area	Area%
1	17.667	23955135	99.547
2	20.726	108943	0.453
Total		24064078	100.000

Product obtained using (*S*,*S*)-$\mathbf{L^2}$ on 0.5 mmol scale:

<Chromatogram>

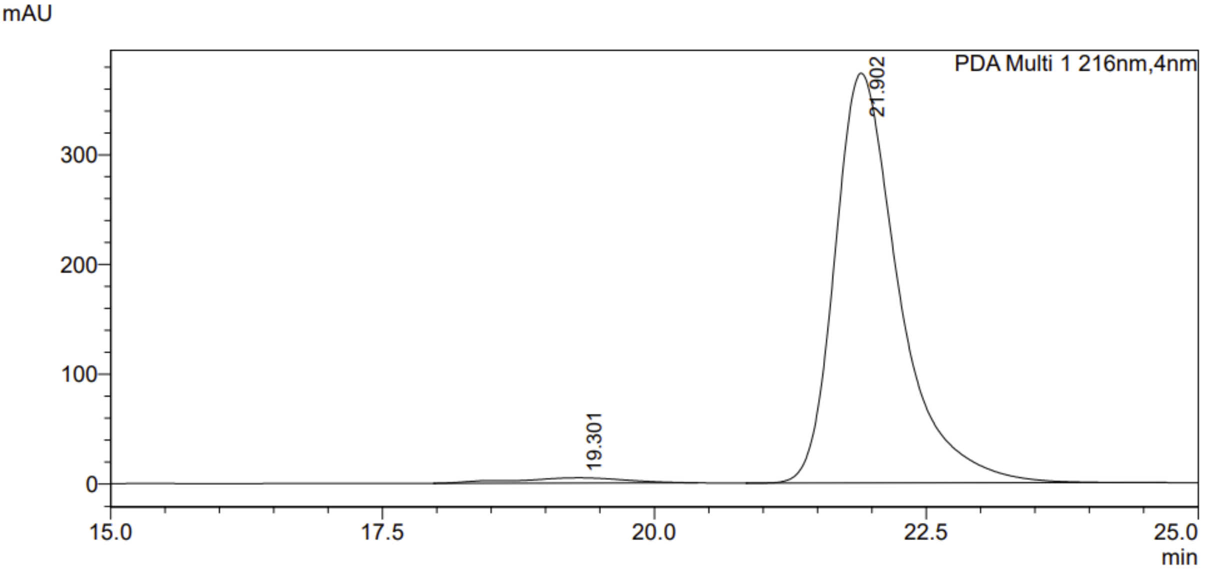

<Peak Table>

PDA Ch1 216nm

Peak#	Ret. Time	Area	Area%
1	19.301	337395	2.160
2	21.902	15284790	97.840
Total		15622184	100.000

^{1}H NMR (400 MHz, Dichloromethane-*d2*)

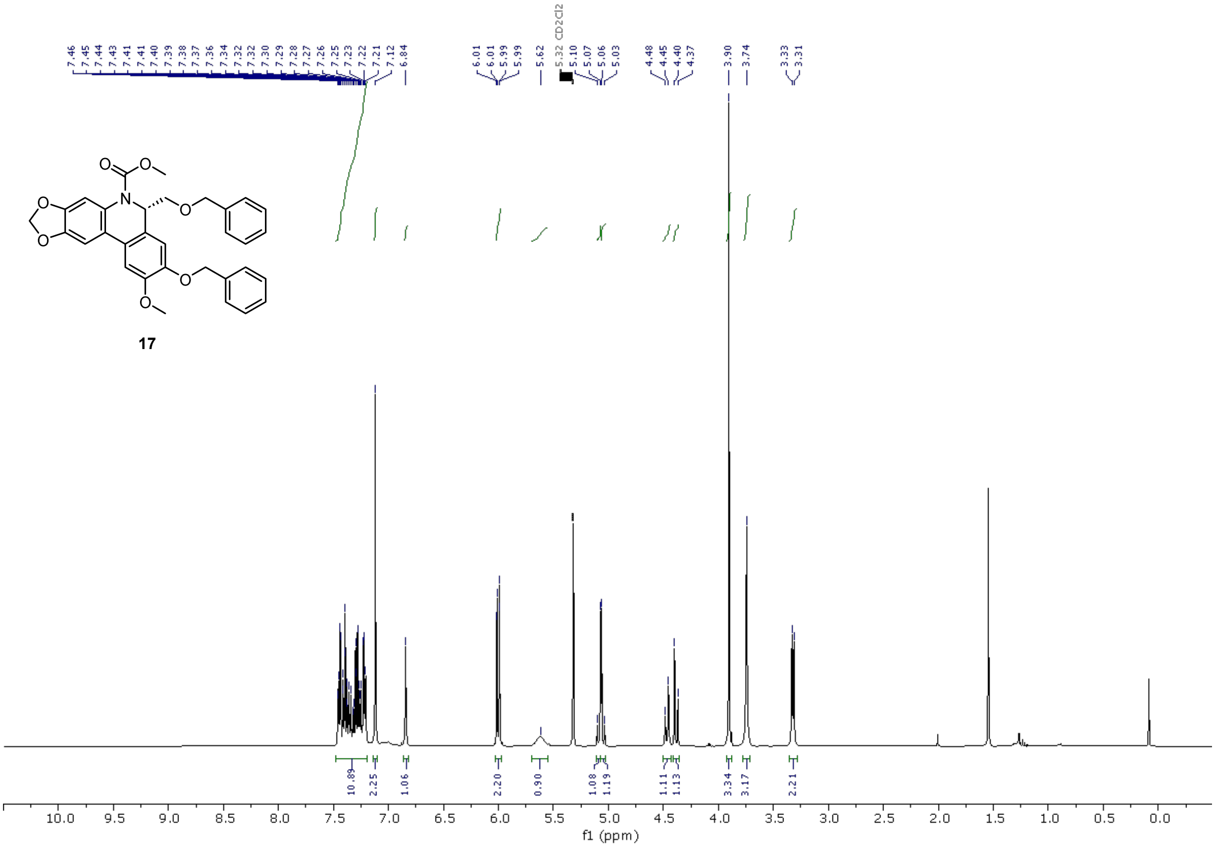

^{13}C{^{1}H} NMR (126 MHz, Dichloromethane-*d2*)

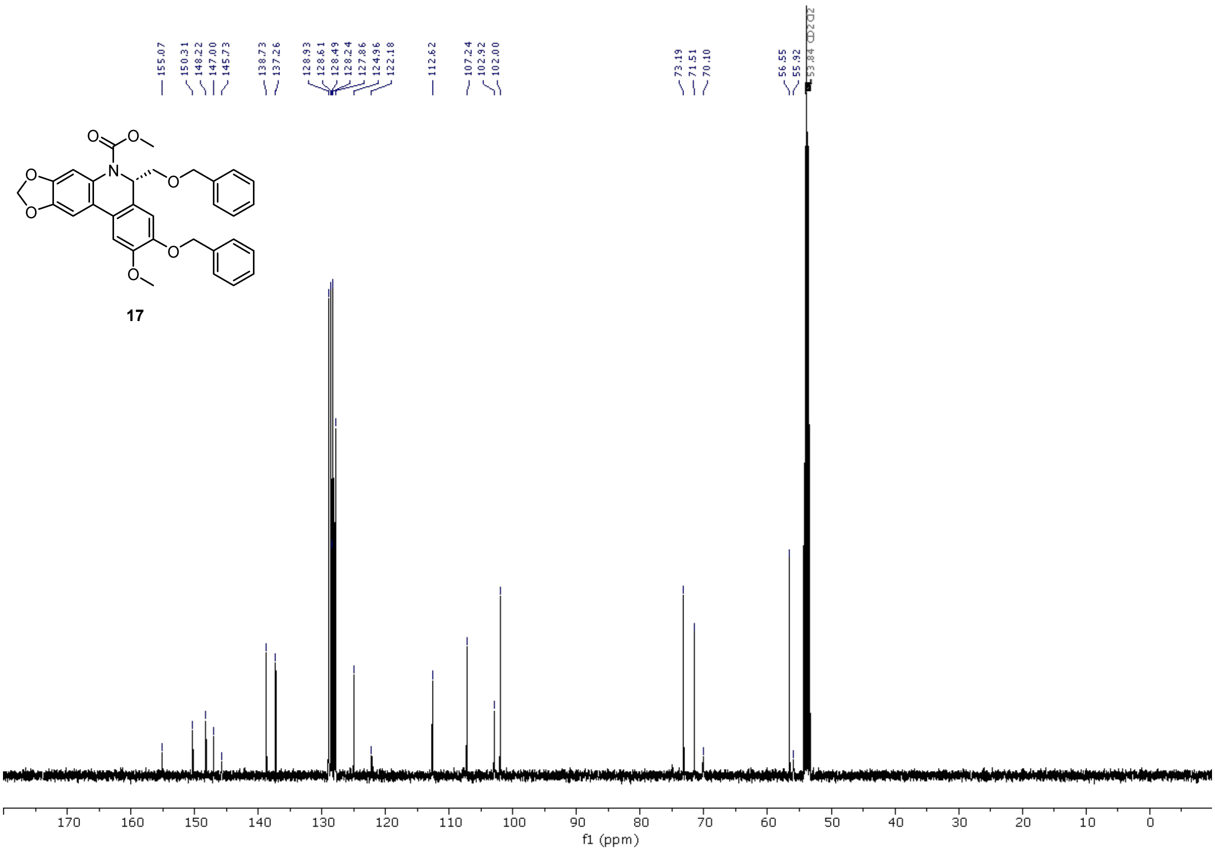

Racemic product obtained using (*rac*)-**L**[2]:

<Chromatogram>

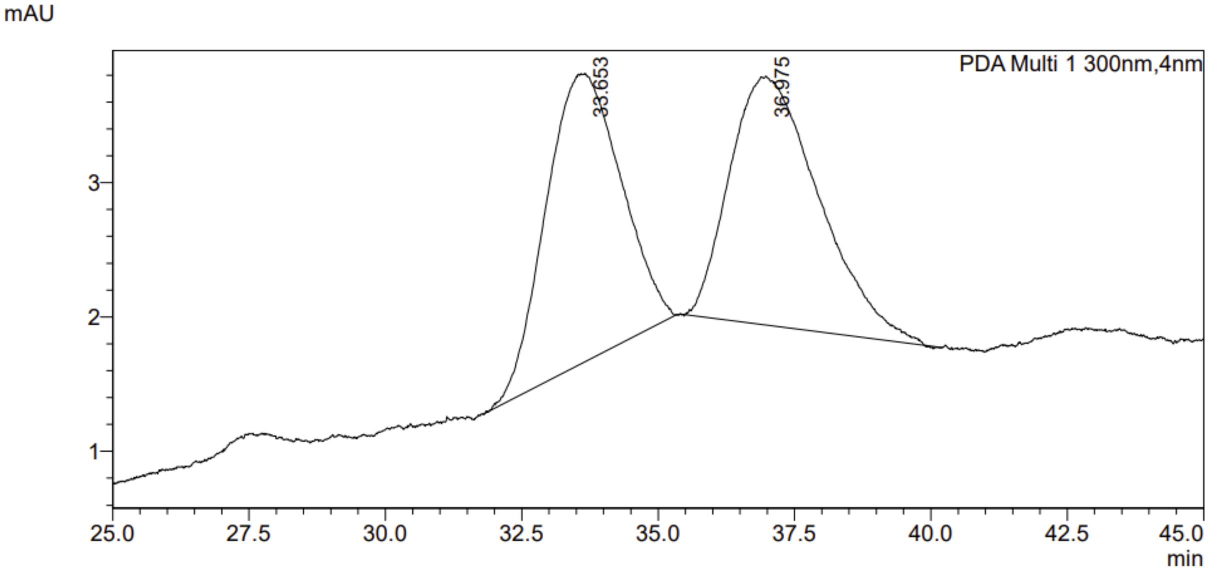

<Peak Table>

PDA Ch1 300nm

Peak#	Ret. Time	Area	Area%
1	33.653	207678	49.605
2	36.975	210983	50.395
Total		418661	100.000

Product obtained using (*R*,*R*)-**L**[3]:

<Chromatogram>

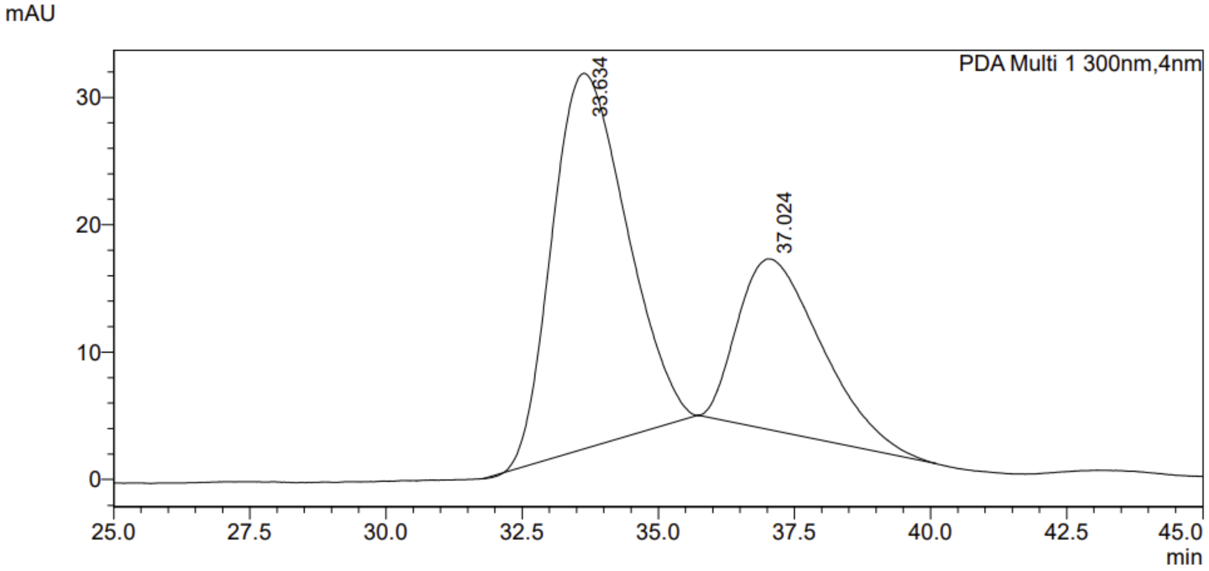

<Peak Table>

PDA Ch1 300nm

Peak#	Ret. Time	Area	Area%
1	33.634	2813622	65.988
2	37.024	1450188	34.012
Total		4263810	100.000

^{1}H NMR (400 MHz, Dichloromethane-*d2*)

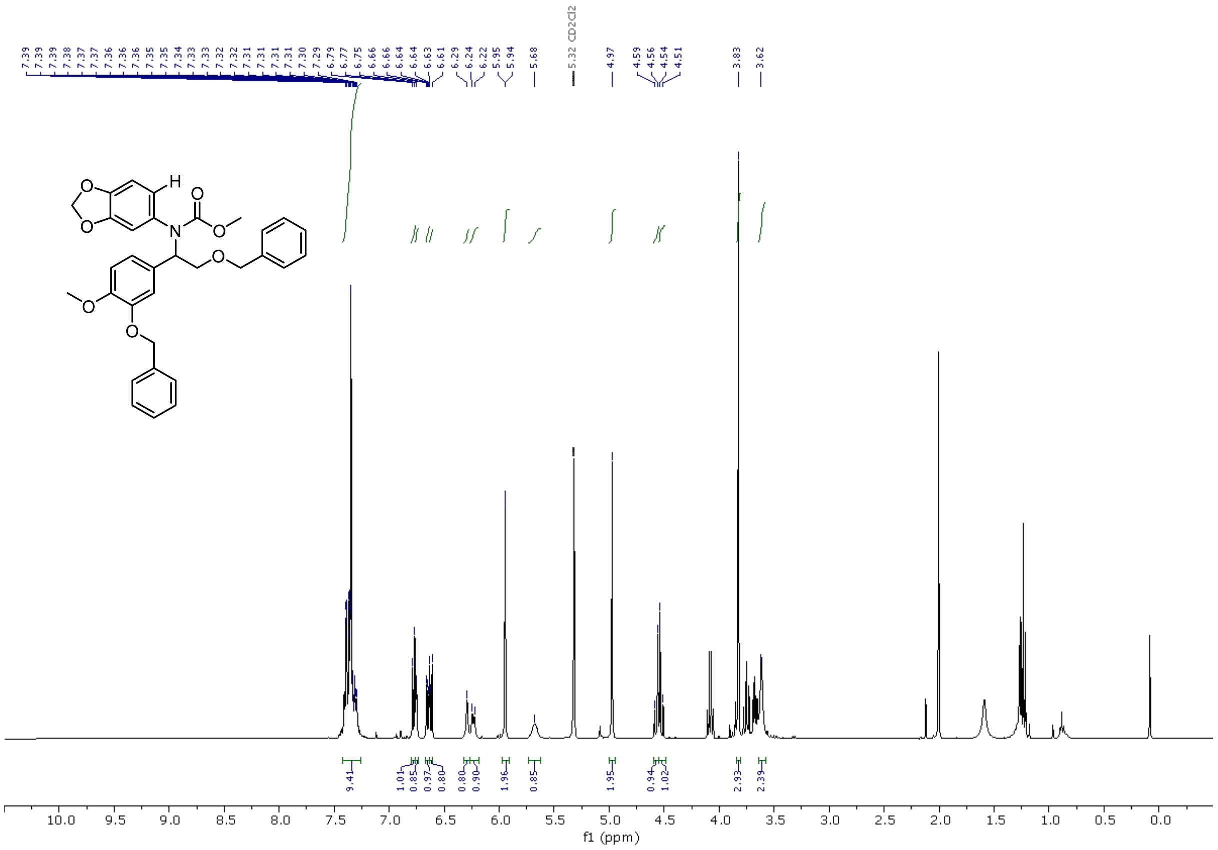

Supplement: Supplementary file 1 — ol4c00386_si_001.pdf [file ol4c00386_si_001.pdf]
